# Supplementary material for: Histidine-rich glycoprotein modulates neutrophils and thrombolysis-associated hemorrhagic transformation
Source: EMBO Mol Med. 2024 Aug 15;16(9):10. doi: 10.1038/s44321-024-00117-y (PMC11393346; doi:10.1038/s44321-024-00117-y)
Supplement: Supplementary file 1 — Table EV1 [file 44321_2024_117_MOESM1_ESM.docx]

**Table EV1. Demographic characteristics of patients with acute ischemic stroke in the discovery cohort.**

|  | **Statistics (n=10)** |  | **Statistics (n=10)** |
| --- | --- | --- | --- |
| **Age**, years | 64.4±11.6 | Anti-platelet, n | 3 |
| **Sex**, female, n | 4 | Anti-coagulation, n | 0 |
| **Risk factors** |  | **Blood pressure** |  |
| Cardiovascular disease, n | 4 | SBP (mmHg) | 166.3±24.9 |
| Hypertension, n | 6 | DBP (mmHg) | 92.6±14.8 |
| Diabetes, n | 2 | **NIHSS score** | 8.6±6.9 |
| Atrial fibrillation, n | 4 | **Time of onset**, hours | 3.2±0.7 |
| Smoking, n | 6 | **Hemorrhagic Transformation** | |
| Drinking, n | 5 | No, n | 8 |
| **Medication** |  | Yes, n | 2 |

Data are shown as means± SEM, SBP = systolic blood pressure, DBP = diastolic blood pressure, NIHSS = National Institute of Health Stroke Scale
