# Supplementary material for: Histidine-rich glycoprotein modulates neutrophils and thrombolysis-associated hemorrhagic transformation
Source: EMBO Mol Med. 2024 Aug 15;16(9):10. doi: 10.1038/s44321-024-00117-y (PMC11393346; doi:10.1038/s44321-024-00117-y)
Supplement: Supplementary file 2 — Table EV2 [file 44321_2024_117_MOESM2_ESM.docx]

**Table EV2. Plasma histidine-rich glycoprotein values of patients in the validation cohort**

|  | **Healthy control (n=53)** | **Stroke patients without tPA treatment (n=42)** | | **Stroke patients with tPA treatment (n=62)** | |
| --- | --- | --- | --- | --- | --- |
|  |  | **3.5h** | **6.5h** | **Before** | **After** |
| HRG values  (μg/ml)  HRG values  (μg/ml) | 75.84  88.70  76.19  73.06  64.03  85.58  71.33  64.72  101.56  97.39  90.79  92.88  65.07  79.32  65.76  87.31  63.68  88.70  83.14  86.25  100.88  86.65  83.09  72.81  97.32  73.21  81.11  73.60  75.98  71.23  94.55  67.68  70.05  74.00  90.21  77.16  74.79  99.30  72.42  83.09  82.70  81.69  86.71  83.26  89.85  81.69  82.32  87.34  91.42  78.55  89.85  78.87  74.79 | 109.67  90.92  99.05  66.13  62.66  79.53  72.26  99.96  81.97  90.24  73.77  69.78  69.25  84.38  94.68  93.67  85.78  83.88  89.02  86.25  82.70  85.07  87.04  74.79  71.63  74.39  102.46  75.10  112.76  77.93  85.77  88.60  79.18  78.55  92.68  86.40  88.60  91.11  85.46  89.54  84.52  82.63 | 98.20  96.33  80.42  77.54  65.70  74.67  104.06  88.22  83.76  95.43  75.26  69.86  66.18  94.02  92.71  94.55  84.46  88.63  91.79  85.86  85.46  79.14  88.23  70.05  72.81  81.11  96.14  71.96  113.70  82.01  91.42  92.68  83.26  81.06  90.17  88.28  88.91  89.22  85.77  90.79  86.09  82.95 | 85.15  93.39  83.98  70.50  80.86  78.20  74.45  70.06  101.55  108.87  86.22  54.83  118.14  61.13  63.11  63.32  71.44  90.60  81.40  92.79  93.23  107.26  80.96  73.51  93.23  98.05  68.69  63.43  59.92  62.55  86.66  80.71  79.72  75.84  111.99  92.18  83.84  65.42  67.85  63.41  79.00  86.95  89.81  77.09  76.45  81.22  81.22  89.81  79.95  86.63  88.22  85.68  83.77  82.81  84.41  82.81  88.54  79.63  79.95  97.22  50.01  113.36 | 91.09  95.88  90.24  77.76  79.31  92.04  86.66  97.69  121.37  99.28  120.10  76.26  112.66  64.42  66.39  72.97  77.58  88.85  86.66  74.38  96.74  106.82  74.38  84.90  97.61  110.32  70.88  64.30  73.95  87.97  87.53  85.58  94.61  75.50  131.80  83.49  62.98  71.33  69.59  66.59  83.13  92.68  74.86  84.41  85.36  91.41  89.50  90.13  95.22  80.59  92.04  96.81  84.09  87.91  91.09  83.45  94.27  91.09  75.82  91.00  53.74  105.91 |
