# Supplementary material for: Histidine-rich glycoprotein modulates neutrophils and thrombolysis-associated hemorrhagic transformation
Source: EMBO Mol Med. 2024 Aug 15;16(9):10. doi: 10.1038/s44321-024-00117-y (PMC11393346; doi:10.1038/s44321-024-00117-y)
Supplement: Supplementary file 3 — Table EV3 [file 44321_2024_117_MOESM3_ESM.docx]

**Table EV3. The Clinical information of individuals from the healthy control group**

| NO. | Age, years | Sex | Risk factors | | | | | |
| --- | --- | --- | --- | --- | --- | --- | --- | --- |
|  |  |  | Cardiovascular disease | Hypertension | Diabetes | Atrial fibrillation | Smoking | Drinking |
| 1  2  3  4  5  6  7  8  9  10  11  12  13  14  15  16  17  18  19  20  21  22  23  24  25  26  27  28  29  30  31  32  33  34  35  36  37  38  39  40  41  42  43  44  45  46  47  48  49  50  51  52  53 | 56  82  72  58  78  56  70  68  65  69  72  70  78  75  52  50  77  70  79  59  69  61  69  54  69  67  66  61  61  64  71  48  79  54  61  62  79  71  69  63  29  71  62  79  65  71  69  70  59  70  70  67  64 | F  M  F  M  F  M  M  M  F  M  F  F  F  M  M  M  M  M  M  M  M  M  F  M  F  M  M  M  M  M  M  M  F  F  M  F  M  M  M  F  M  M  M  F  M  M  M  F  F  M  M  M  M | N  N  N  N  N  N  N  N  Y  N  N  Y  N  N  N  N  Y  N  N  N  N  N  N  N  N  N  N  N  N  N  Y  N  Y  Y  N  Y  N  N  Y  N  N  N  N  N  Y  N  N  Y  Y  N  N  Y  N | N  N  N  N  Y  N  Y  Y  Y  Y  N  Y  Y  Y  N  Y  Y  Y  Y  Y  Y  Y  N  N  Y  N  Y  Y  Y  Y  Y  Y  Y  N  N  N  N  Y  N  Y  Y  Y  Y  Y  Y  Y  N  Y  N  Y  Y  Y  N | N  N  N  N  Y  N  N  N  Y  N  N  N  Y  N  N  N  Y  Y  N  Y  N  N  N  N  Y  N  N  Y  Y  N  Y  Y  N  N  N  N  N  Y  Y  Y  Y  Y  N  N  N  N  N  Y  N  N  Y  Y  N | N  N  Y  N  N  N  Y  N  N  N  N  N  N  N  N  Y  N  N  N  N  N  N  N  N  N  N  N  N  N  N  N  N  N  N  N  N  N  Y  N  N  N  N  N  N  N  Y  N  N  Y  N  N  N  N | N  Y  N  Y  N  N  Y  Y  N  Y  N  N  N  Y  Y  Y  N  Y  N  Y  N  Y  N  N  N  Y  Y  Y  N  Y  N  Y  N  N  N  N  N  N  Y  N  N  Y  Y  N  Y  Y  N  N  N  Y  Y  Y  Y | N  Y  N  Y  N  Y  Y  Y  N  Y  N  N  N  N  Y  Y  N  Y  N  Y  Y  N  N  N  N  Y  N  Y  Y  Y  N  Y  N  N  N  N  N  Y  Y  N  N  Y  Y  N  Y  N  N  N  N  Y  Y  Y  Y |

M = Male, F = Female, Y = Yes, N = No
