# Supplementary material for: Histidine-rich glycoprotein modulates neutrophils and thrombolysis-associated hemorrhagic transformation
Source: EMBO Mol Med. 2024 Aug 15;16(9):10. doi: 10.1038/s44321-024-00117-y (PMC11393346; doi:10.1038/s44321-024-00117-y)
Supplement: Supplementary file 4 — Table EV4 [file 44321_2024_117_MOESM4_ESM.docx]

**Table EV4. Clinical information of ischemic stroke patients without tPA treatment**

| No. | Age, years | Sex | Risk factors | | | | | | NIHSS score | Time of onset, hours | Hemorrhagic Transformation |
| --- | --- | --- | --- | --- | --- | --- | --- | --- | --- | --- | --- |
|  |  |  | Cardiovascular disease | Hypertension | Diabetes | Atrial fibrillation | Smoking | Drinking |  |  |  |
| 1  2  3  4  5  6  7  8  9  10  11  12  13  14  15  16  17  18  19  20  21  22  23  24  25  26  27  28  29  30  31  32  33  34  35  36  37  38  39  40  41  42 | 70  73  68  76  71  83  61  67  72  73  69  68  53  89  68  90  64  68  61  51  58  64  64  93  59  79  45  67  55  69  68  48  51  81  62  79  65  71  73  49  82  91 | F  M  M  M  F  F  F  M  M  M  M  F  F  M  F  F  M  M  M  F  M  M  M  F  M  M  M  M  M  M  M  M  F  M  M  M  M  M  M  F  F  F | N  Y  N  Y  N  Y  N  N  N  N  N  N  Y  N  N  N  N  N  N  N  Y  N  N  N  N  N  N  N  N  Y  N  N  N  N  N  N  N  N  N  N  N  N | Y  Y  Y  Y  Y  Y  Y  N  N  N  Y  Y  N  Y  Y  N  N  Y  N  Y  Y  Y  N  Y  N  Y  N  N  N  Y  Y  Y  N  Y  N  Y  Y  N  Y  Y  N  N | Y  N  Y  Y  Y  Y  N  N  Y  N  Y  N  N  Y  Y  N  N  Y  N  Y  Y  N  N  N  N  N  N  Y  N  N  Y  N  N  N  N  N  N  N  N  N  N  N | N  N  N  N  Y  N  N  N  N  N  N  N  Y  N  N  N  N  N  N  N  Y  N  N  N  N  Y  N  N  N  N  Y  N  N  N  N  N  Y  N  N  N  Y  N | Y  Y  N  Y  N  N  N  Y  N  N  Y  N  Y  Y  N  N  Y  Y  Y  N  Y  N  Y  N  N  Y  N  Y  N  Y  Y  N  N  N  Y  Y  Y  Y  Y  N  N  N | N  Y  Y  Y  N  N  N  Y  Y  Y  Y  N  N  Y  N  N  Y  Y  Y  N  N  Y  Y  N  Y  Y  N  N  N  Y  Y  N  N  N  Y  Y  Y  Y  Y  N  N  N | 3  2  5  2  11  2  4  2  3  5  11  4  18  9  10  12  5  2  5  16  9  3  2  22  1  4  3  4  1  3  2  2  4  2  3  2  4  2  13  7  3  6 | 1  3  3.5  3  4.5  3.5  2.5  3  4  3  3.5  2  4  3  3  3  3.5  4  3  3  3  2.5  2  2  4  4  3.5  4  2  2  4  2  3  4  2  2  2.5  3.5  3  3.5  3  3 | N  N  N  N  Y  N  N  N  N  N  N  N  N  N  N  N  N  N  N  N  N  N  N  N  N  N  N  N  N  N  N  N  N  N  N  N  N  N  N  N  N  N |

M = Male, F = Female, Y = Yes, N = No
