# Supplementary material for: Histidine-rich glycoprotein modulates neutrophils and thrombolysis-associated hemorrhagic transformation
Source: EMBO Mol Med. 2024 Aug 15;16(9):10. doi: 10.1038/s44321-024-00117-y (PMC11393346; doi:10.1038/s44321-024-00117-y)
Supplement: Supplementary file 5 — Table EV5 [file 44321_2024_117_MOESM5_ESM.docx]

**Table EV5. Clinical information of ischemic stroke patients with tPA treatment**

| NO. | Age, years | Sex | Risk factors | | | | | | NIHSS score | Time of onset, hours | Hemorrhagic Transformation |
| --- | --- | --- | --- | --- | --- | --- | --- | --- | --- | --- | --- |
|  |  |  | Cardiovascular disease | Hypertension | Diabetes | Atrial fibrillation | Smoking | Drinking |  |  |  |
| 1  2  3  4  5  6  7  8  9  10  11  12  13  14  15  16  17  18  19  20  21  22  23  24  25  26  27  28  29  30  31  32  33  34  35  36  37  38  39  40  41  42  43  44  45  46  47  48  49  50  51  52  53  54  55  56  57  58  59  60  61  62 | 41  65  64  54  66  82  62  66  83  61  76  71  56  66  63  72  53  70  78  69  69  74  64  70  66  71  61  61  75  72  58  73  59  63  77  83  66  71  42  71  52  54  80  78  60  59  65  80  65  62  56  78  66  69  56  55  70  57  45  56  76  57 | M  M  M  M  F  F  M  F  F  M  M  M  M  F  M  F  M  M  M  M  M  F  M  M  F  M  F  M  F  M  M  M  M  M  M  F  M  F  M  F  M  M  M  M  M  M  M  M  M  F  M  F  M  F  M  M  F  M  M  M  M  M | N  N  N  N  N  Y  Y  Y  Y  N  N  N  N  Y  N  Y  N  N  N  Y  N  N  N  N  N  N  N  N  N  N  N  N  N  Y  N  Y  N  N  N  N  N  N  N  Y  N  N  N  N  N  N  N  N  N  Y  N  N  N  N  Y  N  N  N | Y  Y  N  N  N  Y  Y  Y  Y  N  Y  N  N  Y  Y  Y  N  N  N  N  N  Y  Y  Y  Y  Y  Y  N  N  Y  Y  Y  N  Y  N  Y  Y  Y  Y  N  Y  Y  Y  N  N  Y  N  Y  Y  Y  N  Y  N  N  N  Y  N  Y  N  Y  Y  Y | N  Y  N  N  N  N  N  N  Y  N  Y  N  N  Y  Y  N  N  N  N  Y  N  Y  N  N  N  N  Y  N  N  N  N  N  N  N  Y  N  N  Y  N  N  Y  Y  Y  Y  N  N  N  N  N  N  N  N  N  N  Y  N  N  Y  N  N  Y  N | N  Y  N  N  Y  Y  N  N  N  Y  Y  N  N  Y  N  N  Y  N  N  N  N  N  N  N  N  N  N  N  N  N  N  Y  N  N  N  N  N  N  N  N  N  N  Y  N  N  N  N  N  N  N  N  N  N  N  N  N  Y  N  N  N  Y  N | Y  Y  N  Y  N  Y  Y  N  N  Y  N  Y  Y  N  Y  N  Y  Y  Y  N  N  N  Y  Y  N  Y  N  Y  N  Y  Y  Y  Y  Y  Y  N  Y  N  Y  Y  Y  N  N  N  Y  N  N  N  Y  N  N  N  N  N  N  Y  N  Y  Y  Y  N  Y | Y  Y  N  Y  N  N  Y  N  N  Y  N  Y  Y  N  Y  N  Y  N  N  N  N  N  Y  N  N  Y  N  N  N  Y  Y  Y  Y  N  Y  N  Y  N  Y  N  Y  N  N  N  Y  Y  N  N  Y  N  N  N  N  N  N  Y  N  Y  Y  Y  N  Y | 11  3  8  3  21  19  14  1  2  4  5  18  12  17  4  7  16  3  18  4  10  4  3  9  1  4  4  5  3  5  6  20  1  8  12  11  9  2  0  5  1  0  2  2  3  2  7  8  1  5  1  15  1  1  5  3  14  11  5  10  17  6 | 4  3  2.5  3  2  2.75  3  4.5  2.75  4  2.5  2  4  3  3  2  3  3  3  3  4  3.5  3.5  4  3  3  4  3  3.5  4.5  3  4.5  3  2  3  4  3.5  2.5  4  3.5  3  4  3.5  4  3  3  2.5  3.5  4  4  4  2.5  4.5  3  4  2  3.5  5  2  1.5  2  3 | N  Y  N  N  Y  N  N  N  N  N  N  N  N  N  N  N  N  N  N  N  N  N  N  N  N  N  N  N  N  N  N  N  N  N  N  N  N  N  N  N  N  N  N  N  N  N  N  N  N  N  N  N  N  N  N  N  N  N  N  Y  Y  Y |

M = Male, F = Female, Y = Yes, N = No
