# Supplementary material for: Histidine-rich glycoprotein modulates neutrophils and thrombolysis-associated hemorrhagic transformation
Source: EMBO Mol Med. 2024 Aug 15;16(9):10. doi: 10.1038/s44321-024-00117-y (PMC11393346; doi:10.1038/s44321-024-00117-y)
Supplement: Supplementary file 6 — Table EV6 [file 44321_2024_117_MOESM6_ESM.docx]

**Table EV6. The sequence for siRNA and PCR primers.**

| HRG siRNA | 5ʹ- GUUCUAGACC UGAUCAAUA- 3ʹ |
| --- | --- |
| PCR primer (GAPDH) | F-5′TGG AGA AAC CTG CCA AGT ATGA 3′  R-5′TGG AAG AAT GGG AGT TGC TGT 3′ |
| PCR primer (HRG) | F-5′TGT CTC TTC AGC ACT TCG CA 3′  R-5′GCC CGT TCC ACT CTG AAA GA 3′ |
