# Supplementary material for: Histidine-rich glycoprotein modulates neutrophils and thrombolysis-associated hemorrhagic transformation
Source: EMBO Mol Med. 2024 Aug 15;16(9):10. doi: 10.1038/s44321-024-00117-y (PMC11393346; doi:10.1038/s44321-024-00117-y)
Supplement: Supplementary file 7 — Appendix [file 44321_2024_117_MOESM7_ESM.pdf]

Histidine-rich glycoprotein modulates neutrophils and thrombolysis-associated hemorrhagic transformation

Appendix

Contents

Appendix Figure S1. Variations in HRG levels at different time points after photothrombotic middle cerebral artery occlusion (MCAO). 2

Appendix Figure S2. Neutrophils viability determined at different time points after tPA and/or HRG treatment in vitro. 3

Appendix Figure S3. Effect of tPA on mononuclear cell mortality. 4

Appendix Figure S4. HRG expression decreases following siRNA delivery in vitro and in vivo. 5

Appendix Figure S5. HRG expression in various tissues following tPA treatment. 6

Appendix Figure S6. Verification of purity of in vitro isolated neutrophils and efficacy of in vivo neutrophil depletion. 7

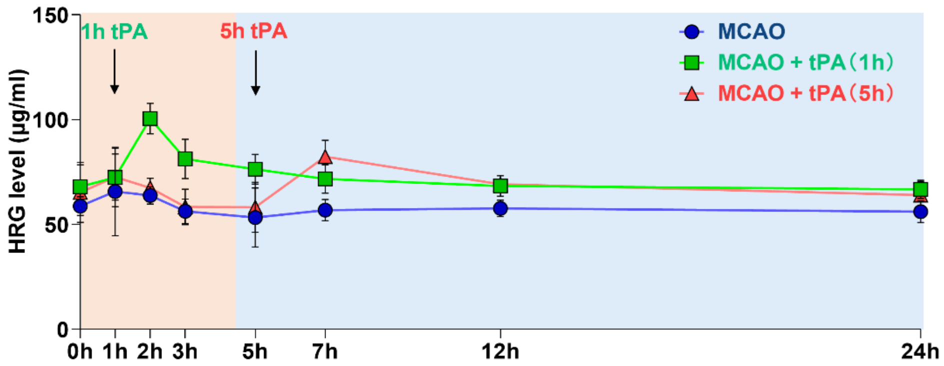

Appendix Figure S1. Variations in HRG levels at different time points after photothrombotic middle cerebral artery occlusion (MCAO).

Blood samples were collected for HRG determination at 1 h, 2 h, 3 h, 5 h, 7 h, 12 h and 24 h after MCAO, and right before MCAO onset (0 h). Mice that underwent early thrombolysis (1h) and delayed thrombolysis (5h) were also included for HRG determination at the indicated times (n = 5-8). Data information: Results are shown as mean ± SEM. Two tailed Kruskal-Wallis H test. Source data are available online for this figure.

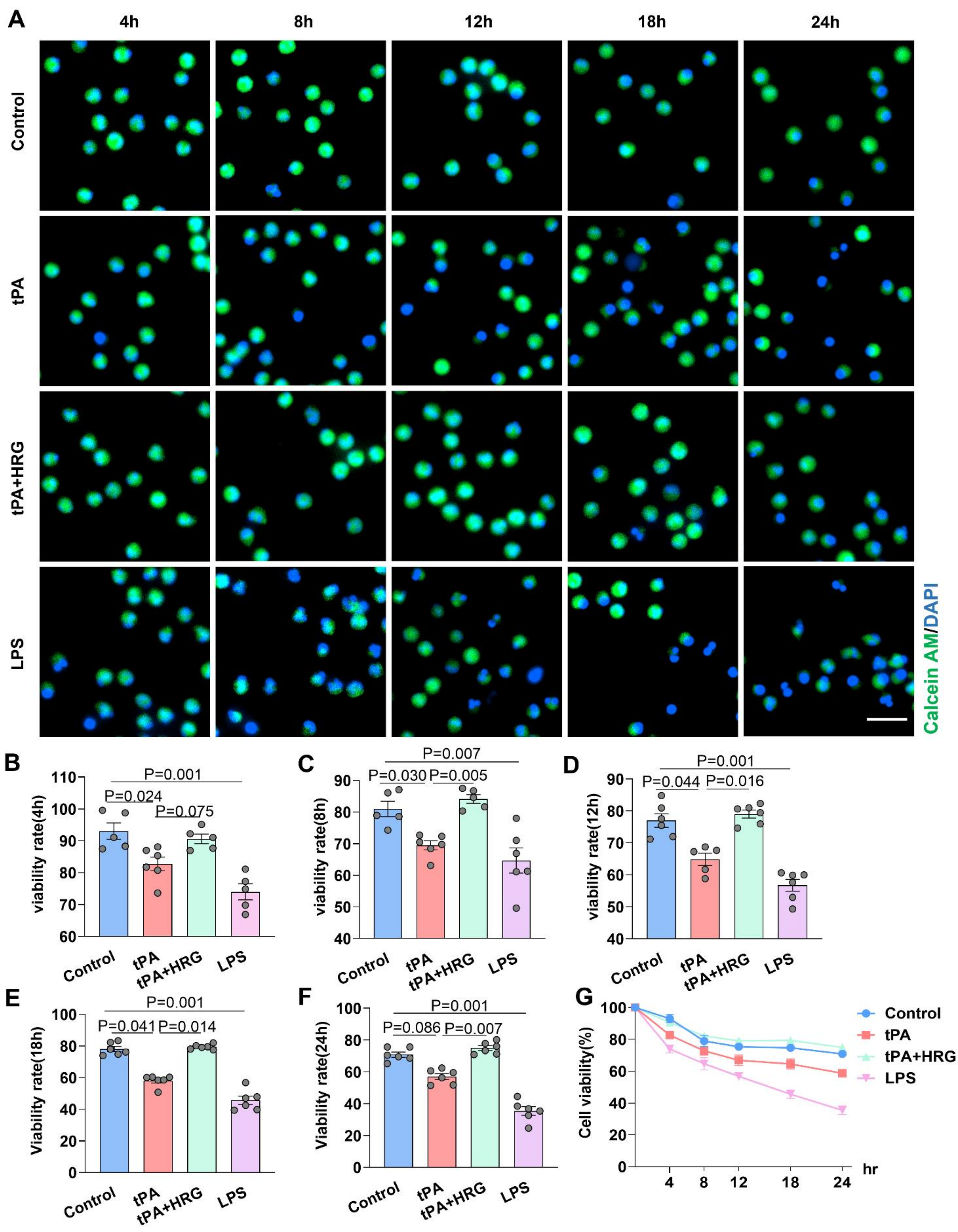

Appendix Figure S2. Neutrophils viability determined at different time points after tPA and/or HRG treatment in vitro.

(A) Neutrophils were labeled with calcein-AM (living cell, green) and Hoechst 33342 (nuclei, blue). tPA and HRG were added to the culture system with LPS (10 µg/ml) added as the positive control. After 4 h, 8 h, 12 h, 18 h and 24 h of culture, the viability of neutrophils was observed and calculated under a fluorescent microscope. Typical results from five independent experiments are shown. Scale bar = 20 µm. **(B-F)**.

Statistical analysis of the cell viability rate at 4 h, 8 h, 12 h, 18 h and 24 h of culture is shown (n = 5-6). **(G)** The line graph summarizes the cell viability at different time points (n = 5-6). Data information: Results are shown as mean ± SEM. Two tailed Kruskal-Wallis H test is used in (B-F). Source data are available online for this figure.

.

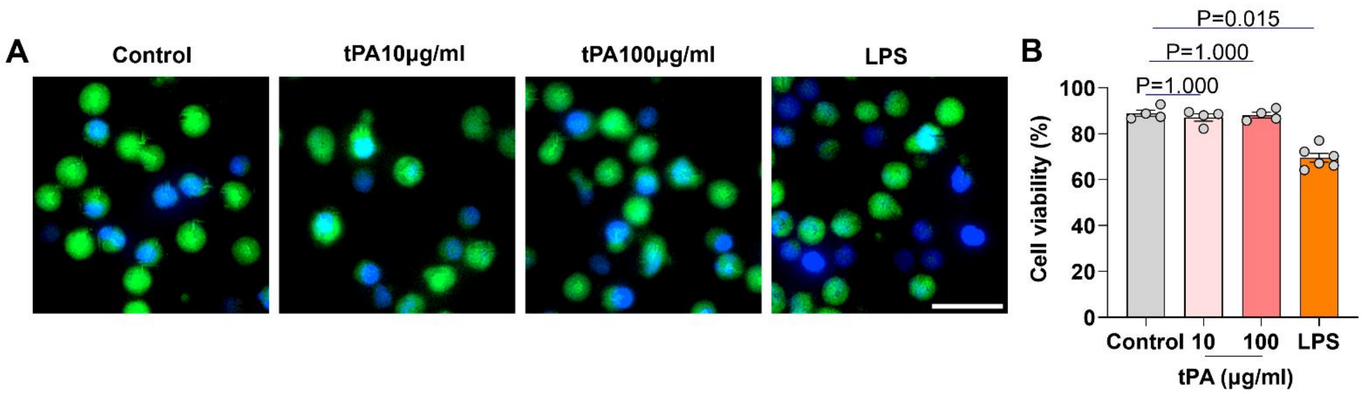

Appendix Figure S3. Effect of tPA on mononuclear cell mortality.

(A) After 18 h of culture, mononuclear cells were labeled with calcein-AM (living cells, green) and Hoechst 33342 (nuclei, blue). The results from five independent experiments are presented. Scale bar = 20 μm. **(B)** Statistical graph showing the cell mortality rate. There was no significant change in monocyte mortality after tPA treatment (n = 6–8). Data information: Results are shown as mean ± SEM. Two tailed Kruskal-Wallis H test is used in (B). Source data are available online for this figure.

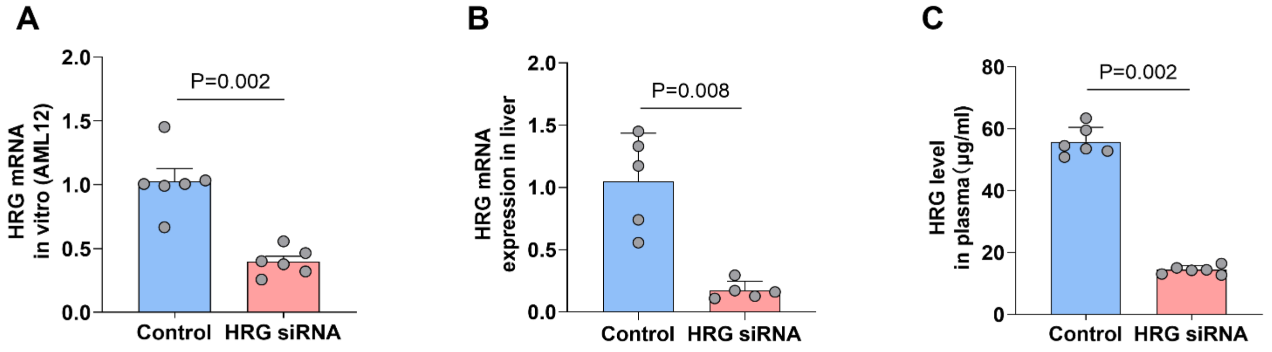

Appendix Figure S4. HRG expression decreases following siRNA delivery in vitro and in vivo.
(A) HRG siRNA was delivered to AML12 hepatocytes in culture, which showed decreased mRNA expression (n=6). **(B)** HRG siRNA was injected into C57BL/6 mice at a dose of 1 mg/kg via the tail vein. HRG mRNA expression in liver was determined 24 hours after the injection, which showed decreased mRNA levels (17±0.07%) compared to the control siRNA group (n=5). **(C)** HRG levels in plasma were determined by ELISA, which showed significant decreases following HRG siRNA treatment (n=6). Data information: Results are shown as mean ± SEM. Two tailed Mann-Whitney U-test is used in (A-C). Source data are available online for this figure.

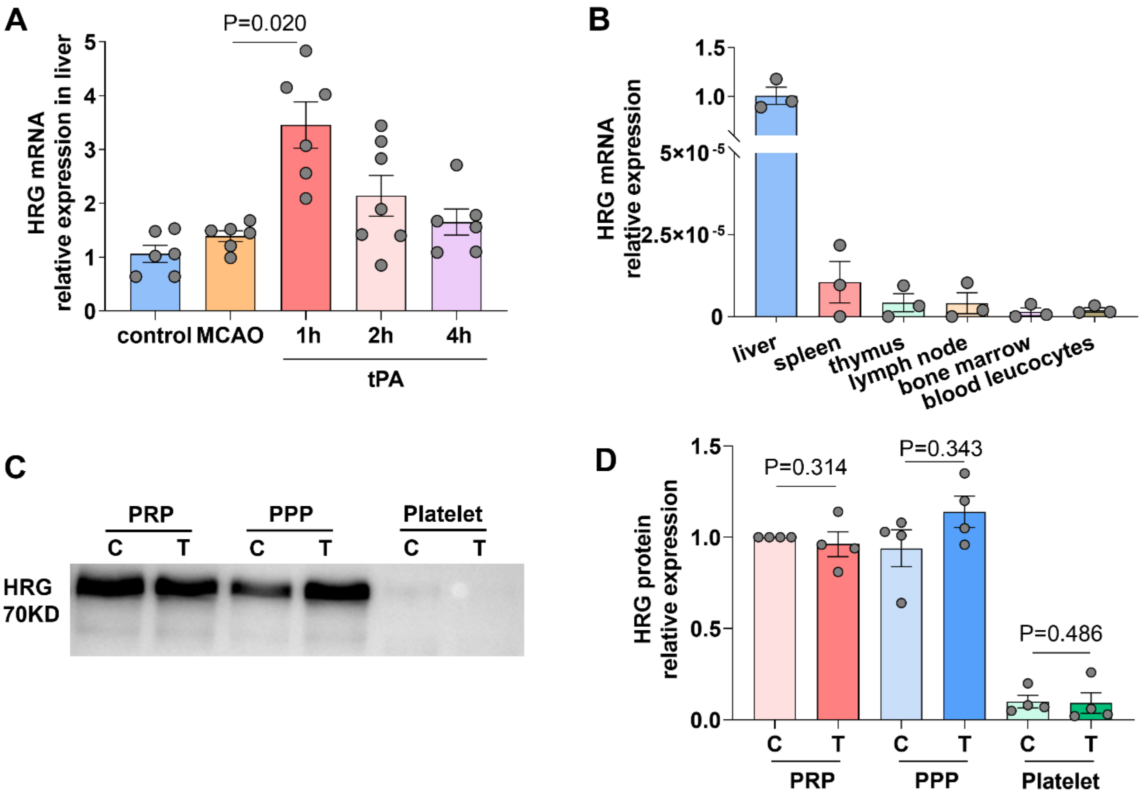

Appendix Figure S5. HRG expression in various tissues following tPA treatment.

(A) HRG mRNA expression in the liver after MCAO induction and tPA administration (n=5-7). **(B)** HRG mRNA expression in the spleen, thymus, lymph node, bone marrow and peripheral blood leukocytes in MCAO mice following tPA treatment (n=3). **(C)** Platelet-rich plasmas (PRP) was collected from human peripheral blood and stimulated with tPA in vitro. Then PRP was centrifuged for platelet-poor plasma (PPP) and platelet separation. HRG protein (70KD) was detected by Western blot analysis. **(D)** Statistical analysis of relative HRG protein expression (n = 4). Data information: Results are shown as mean ± SEM. Two tailed Kruskal-Wallis H test is used in (A, D). Source data are available online for this figure.

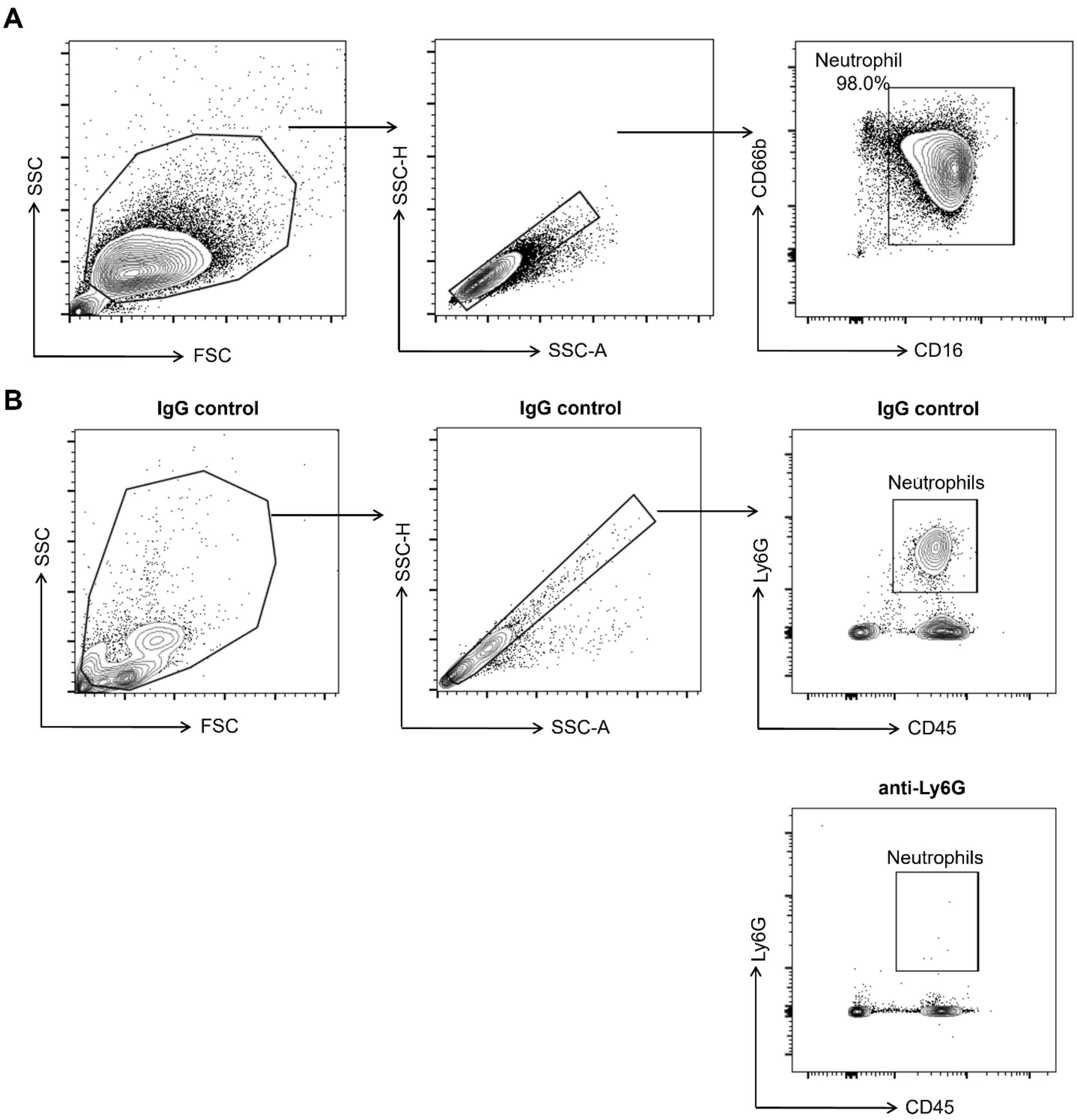

Appendix Figure S6. Verification of purity of in vitro isolated neutrophils and efficacy of in vivo neutrophil depletion.

(A) The gating strategy of neutrophils isolated from peripheral blood and determination of their purity by flow cytometry. **(B)** The gating strategy for neutrophil identification after control IgG and anti-Ly6G IgG injection in vivo. Source data are available online for this figure.
