# Supplementary figures and images for: Histidine-rich glycoprotein modulates neutrophils and thrombolysis-associated hemorrhagic transformation (part 1 of 2)
Source: EMBO Mol Med. 2024 Aug 15;16(9):10. doi: 10.1038/s44321-024-00117-y (PMC11393346; doi:10.1038/s44321-024-00117-y)

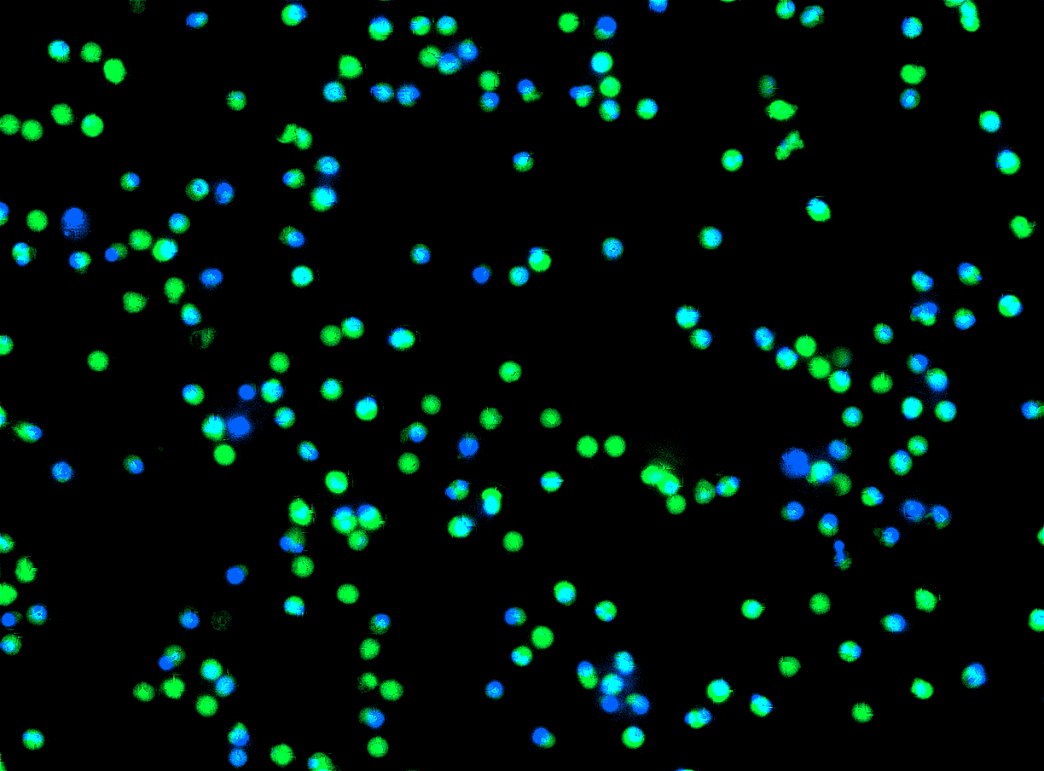

Supplement: Supplementary file 9 — Source data Fig. 3 [file 44321_2024_117_MOESM9_ESM.zip › Figure 3/3A/Control.tif]

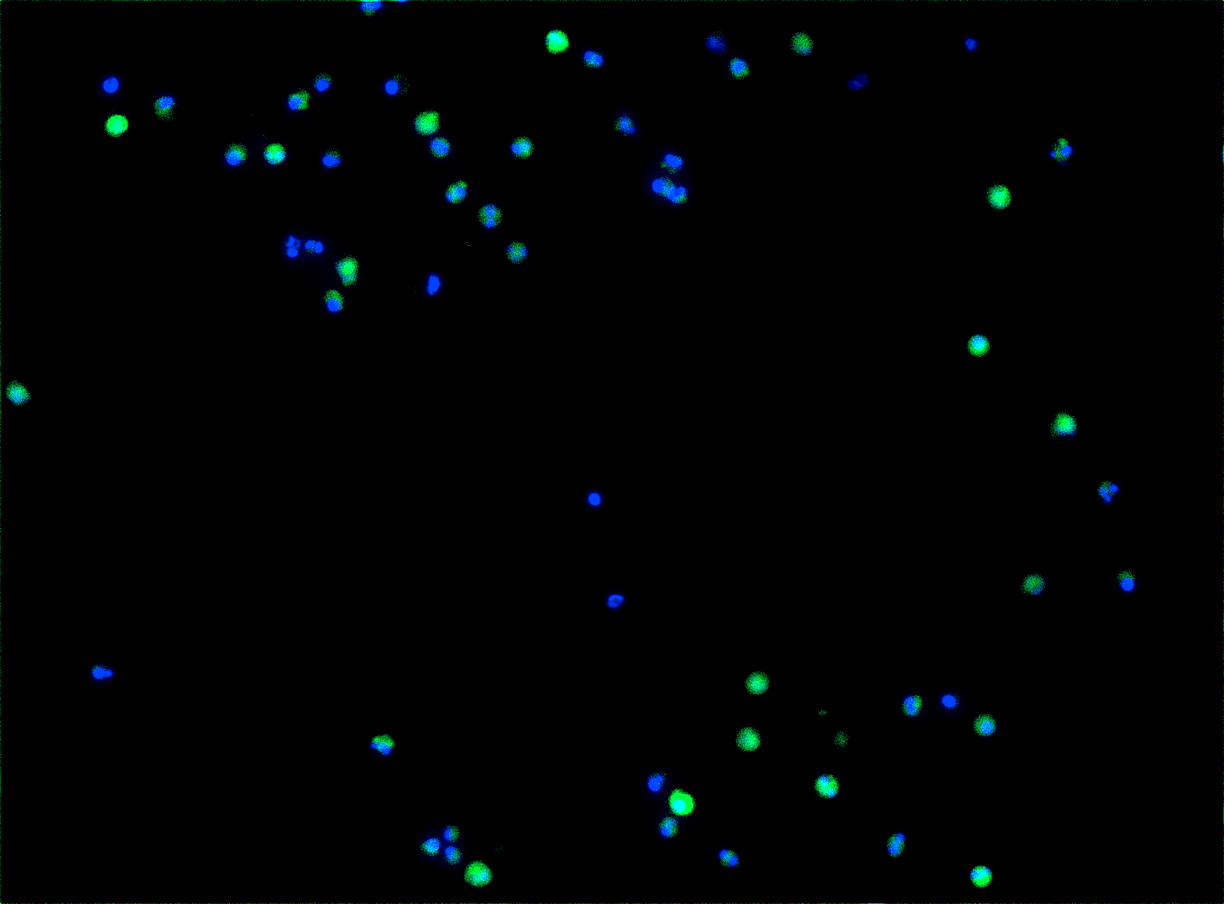

Supplement: Supplementary file 9 — Source data Fig. 3 [file 44321_2024_117_MOESM9_ESM.zip › Figure 3/3A/LPS.tif]

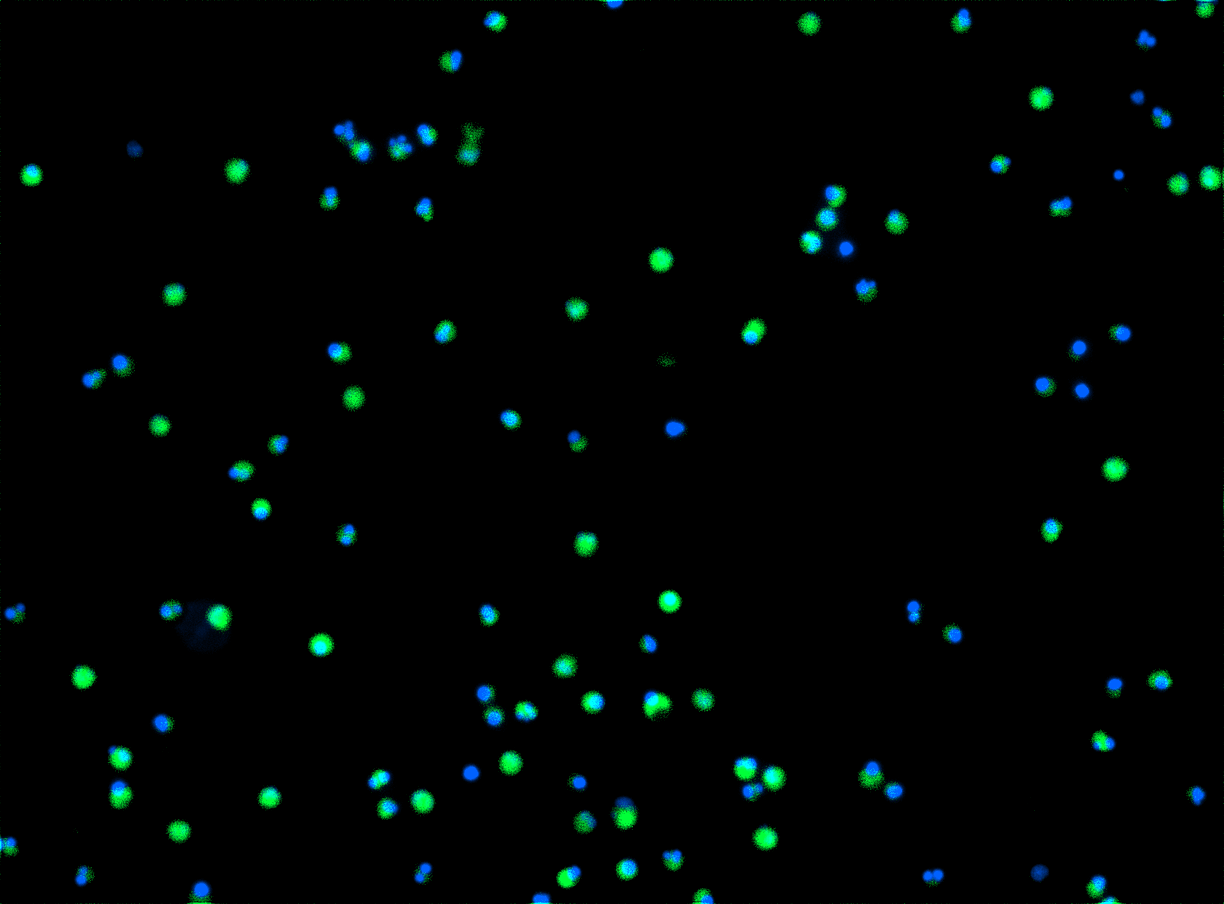

Supplement: Supplementary file 9 — Source data Fig. 3 [file 44321_2024_117_MOESM9_ESM.zip › Figure 3/3A/tPA_100μg_ml.tif]

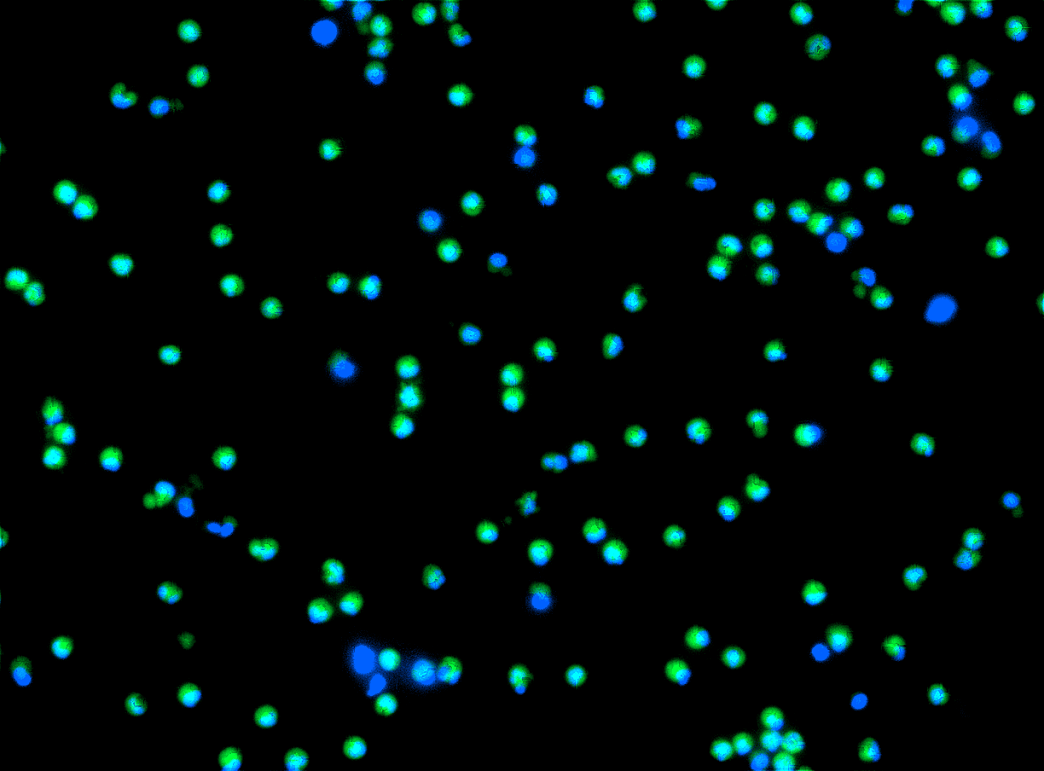

Supplement: Supplementary file 9 — Source data Fig. 3 [file 44321_2024_117_MOESM9_ESM.zip › Figure 3/3A/tPA_10μg_ml.tif]

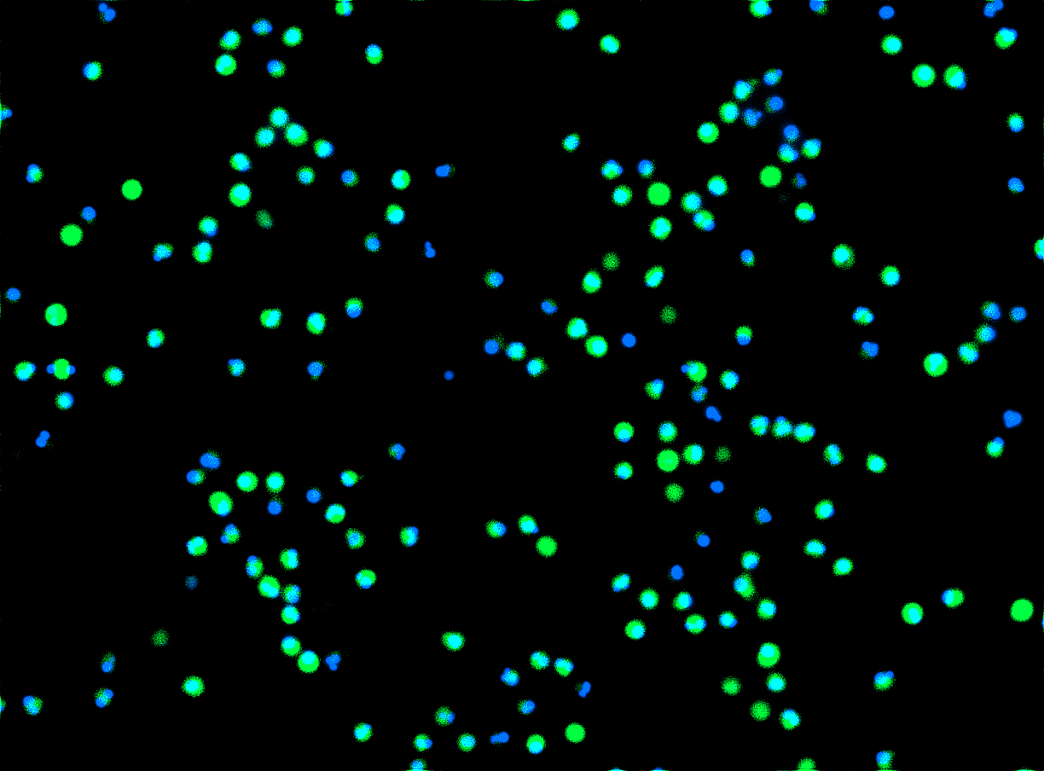

Supplement: Supplementary file 9 — Source data Fig. 3 [file 44321_2024_117_MOESM9_ESM.zip › Figure 3/3A/tPA_2.5μg_ml.tif]

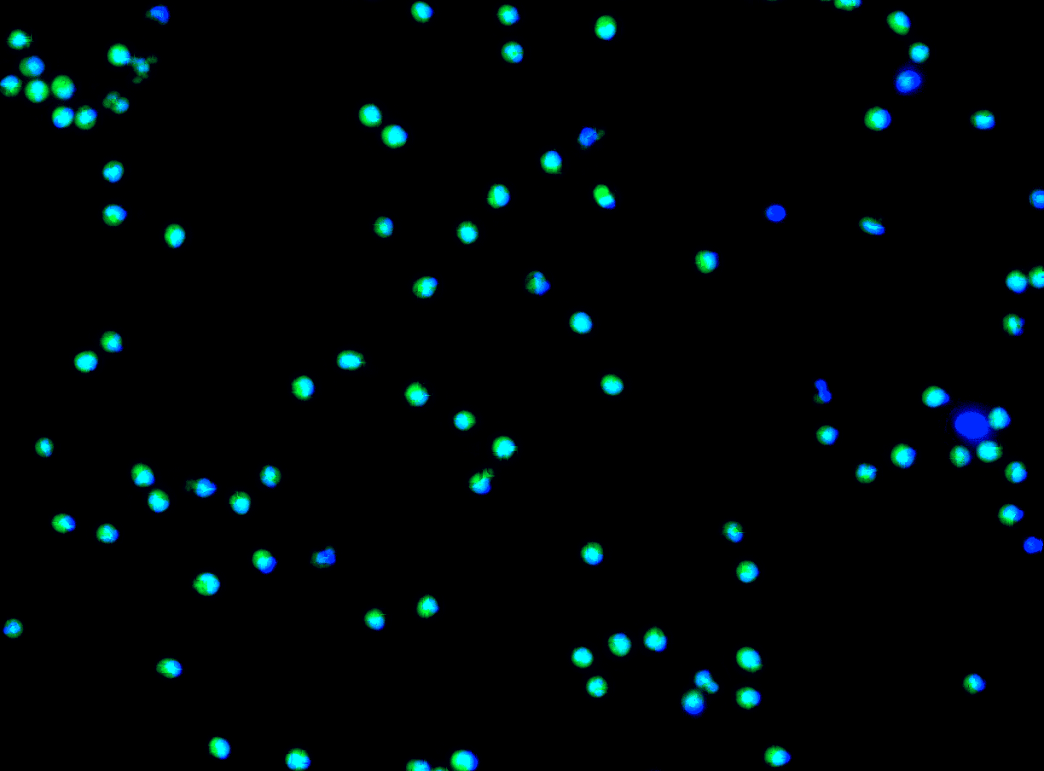

Supplement: Supplementary file 9 — Source data Fig. 3 [file 44321_2024_117_MOESM9_ESM.zip › Figure 3/3A/tPA_20μg_ml.tif]

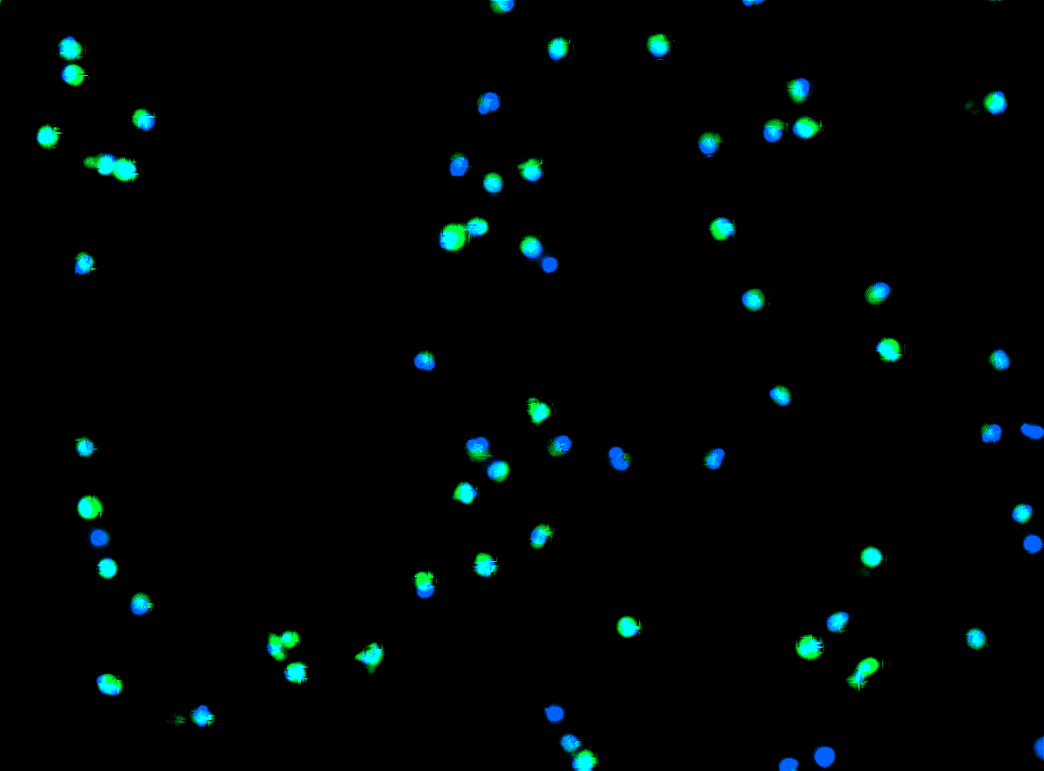

Supplement: Supplementary file 9 — Source data Fig. 3 [file 44321_2024_117_MOESM9_ESM.zip › Figure 3/3A/tPA_40μg_ml.tif]

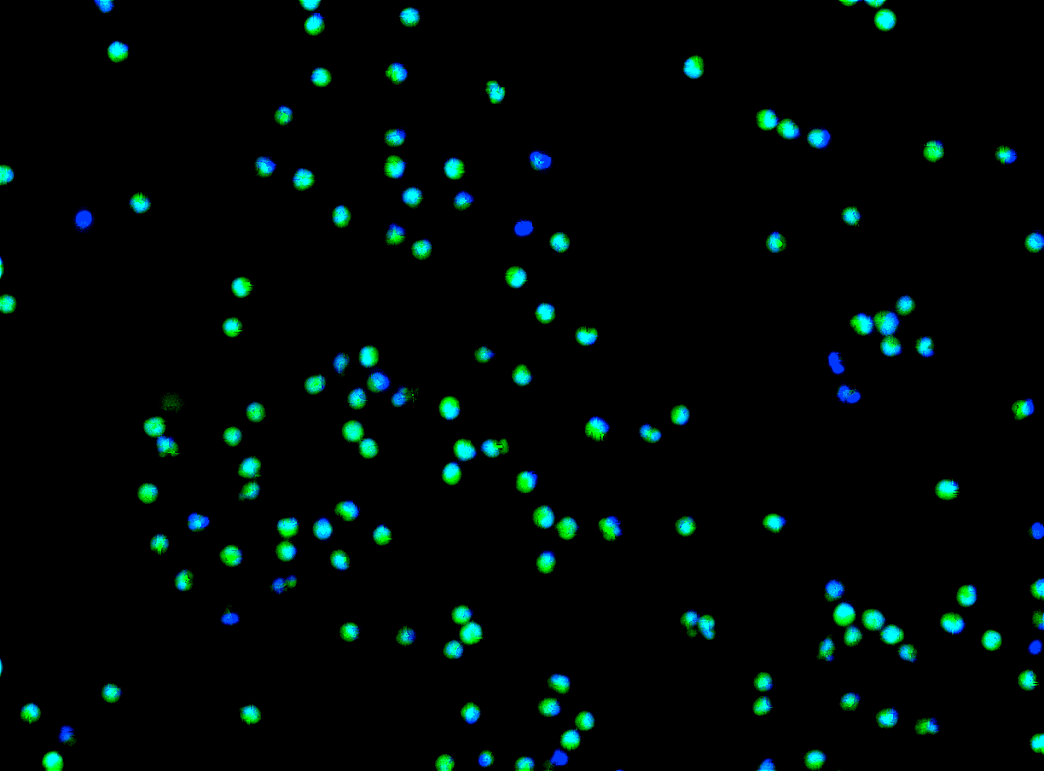

Supplement: Supplementary file 9 — Source data Fig. 3 [file 44321_2024_117_MOESM9_ESM.zip › Figure 3/3A/tPA_5μg_ml.tif]

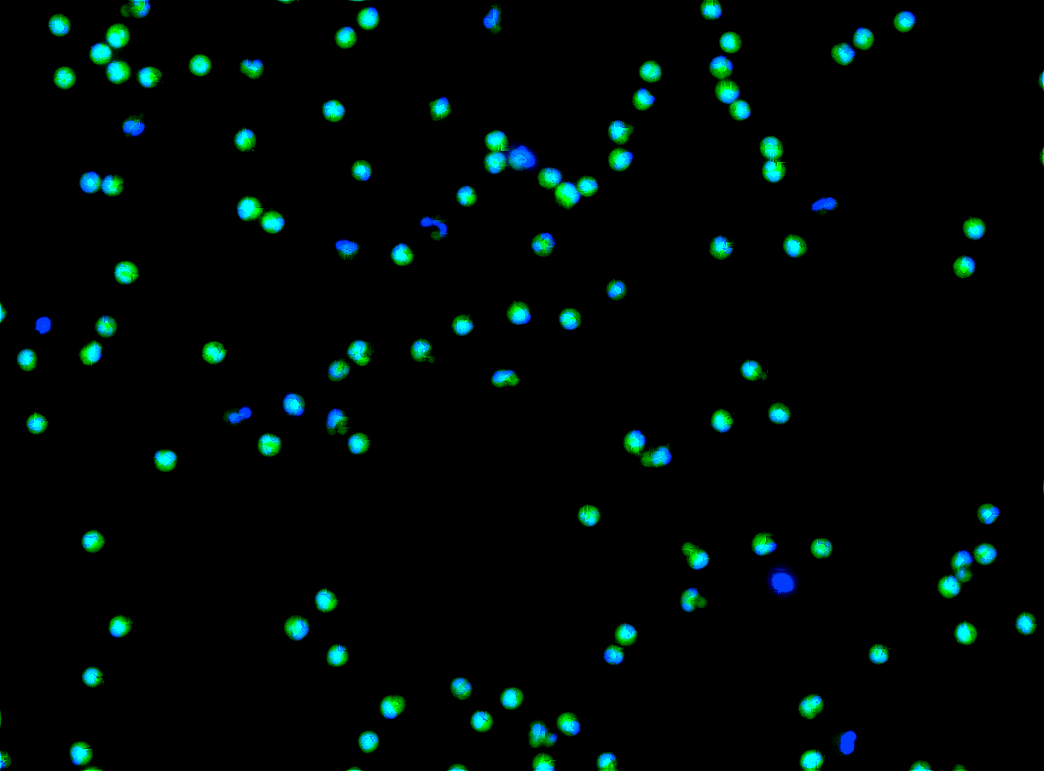

Supplement: Supplementary file 9 — Source data Fig. 3 [file 44321_2024_117_MOESM9_ESM.zip › Figure 3/3D/HRG_0.00_μM.tif]

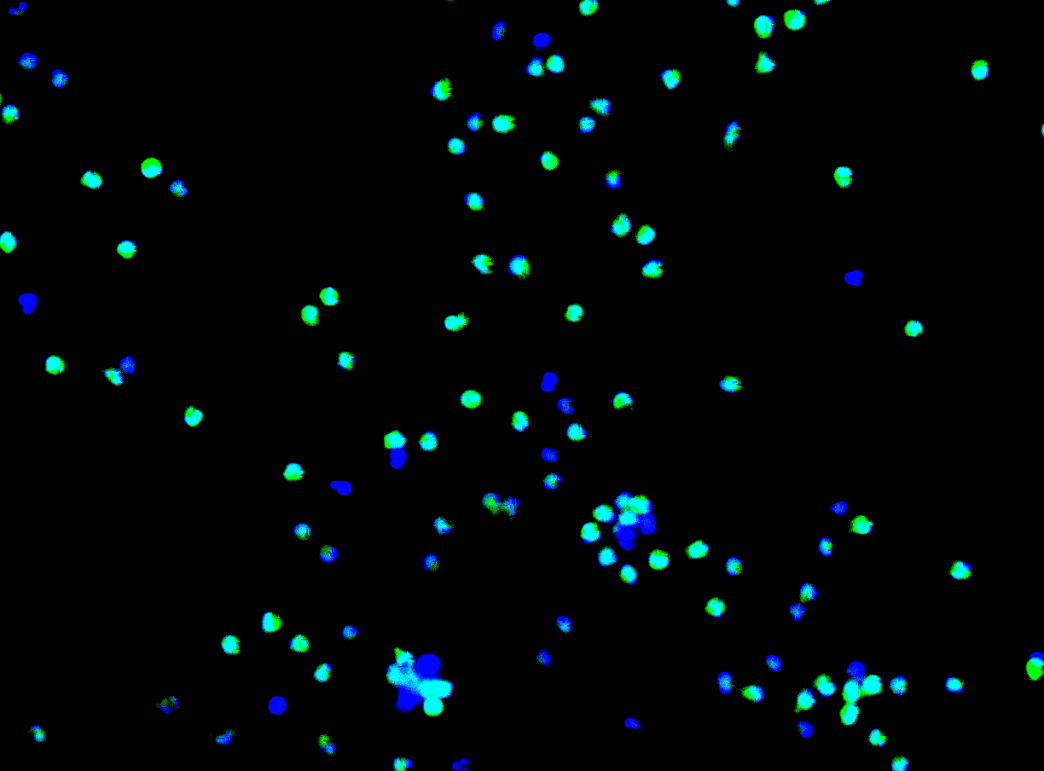

Supplement: Supplementary file 9 — Source data Fig. 3 [file 44321_2024_117_MOESM9_ESM.zip › Figure 3/3D/HRG_0.05_μM.tif]

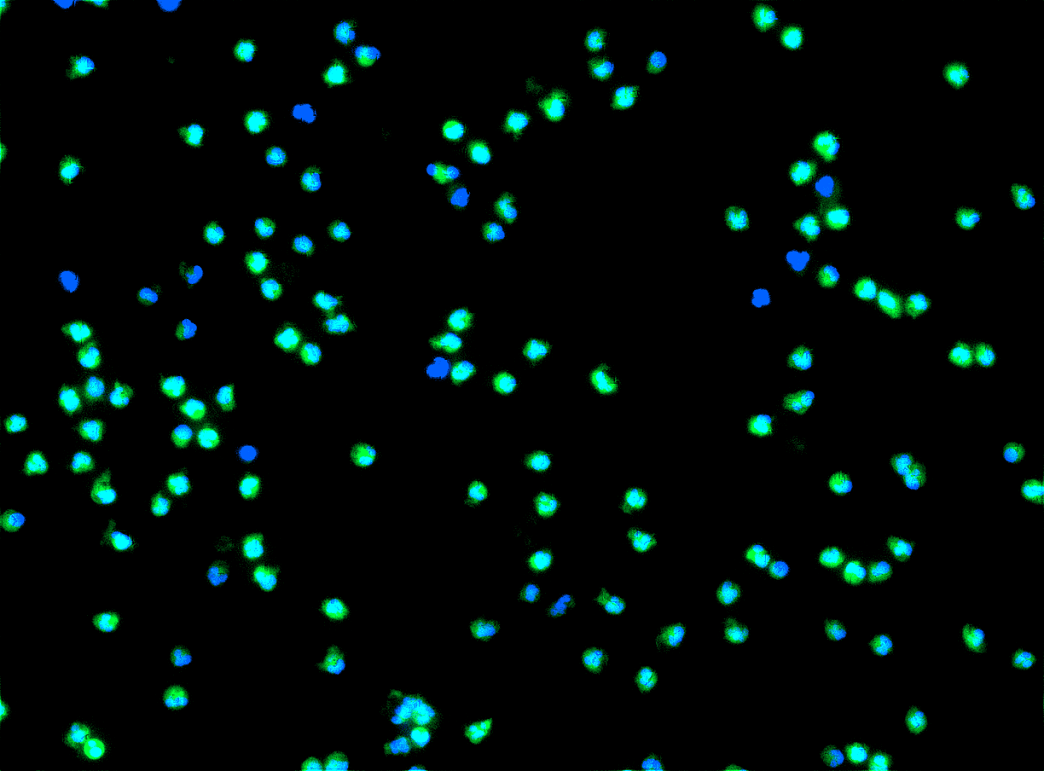

Supplement: Supplementary file 9 — Source data Fig. 3 [file 44321_2024_117_MOESM9_ESM.zip › Figure 3/3D/HRG_0.10_μM.tif]

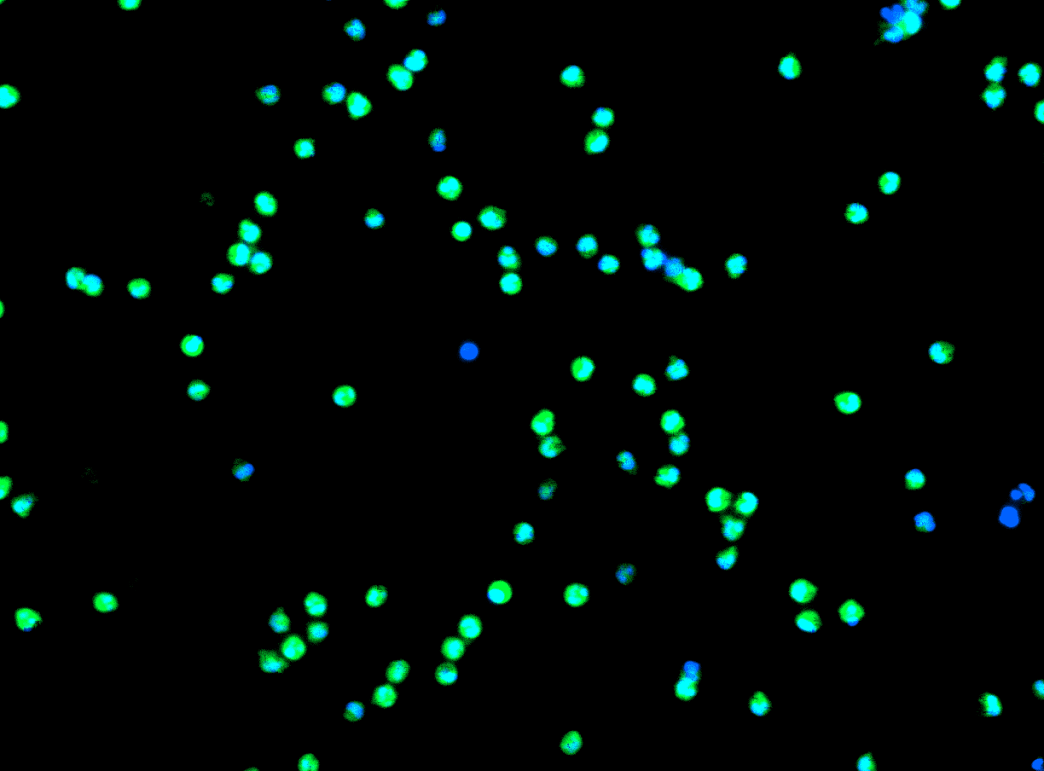

Supplement: Supplementary file 9 — Source data Fig. 3 [file 44321_2024_117_MOESM9_ESM.zip › Figure 3/3D/HRG_0.25_μM.tif]

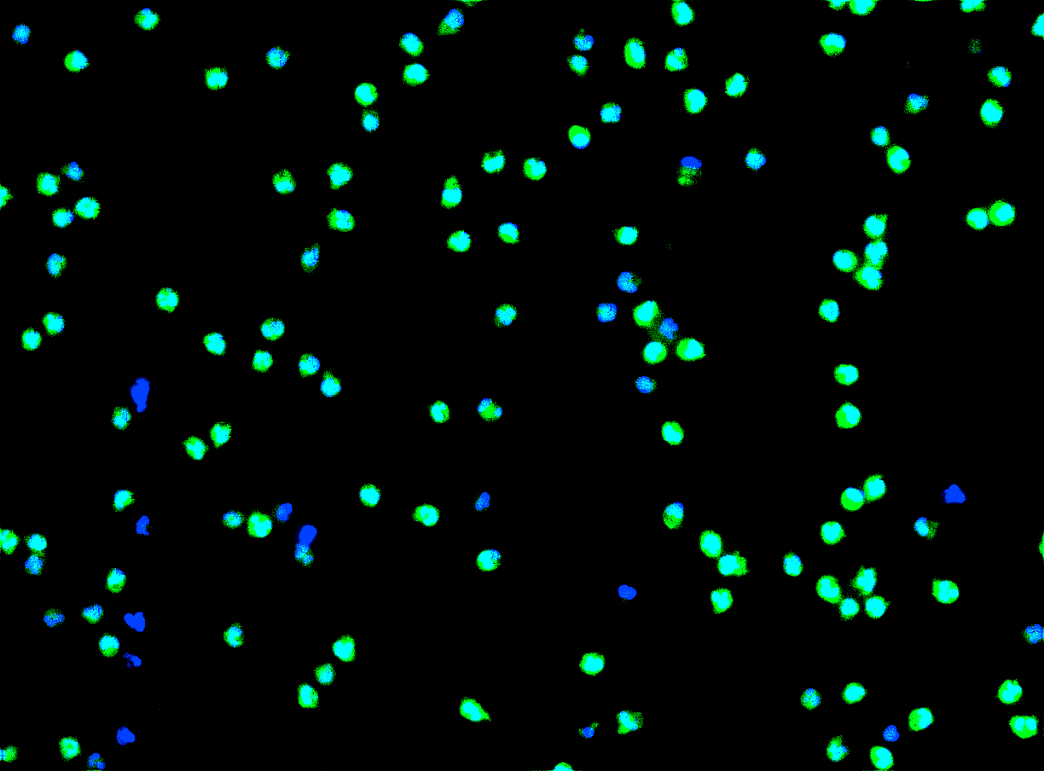

Supplement: Supplementary file 9 — Source data Fig. 3 [file 44321_2024_117_MOESM9_ESM.zip › Figure 3/3D/HRG_0.50_μM.tif]

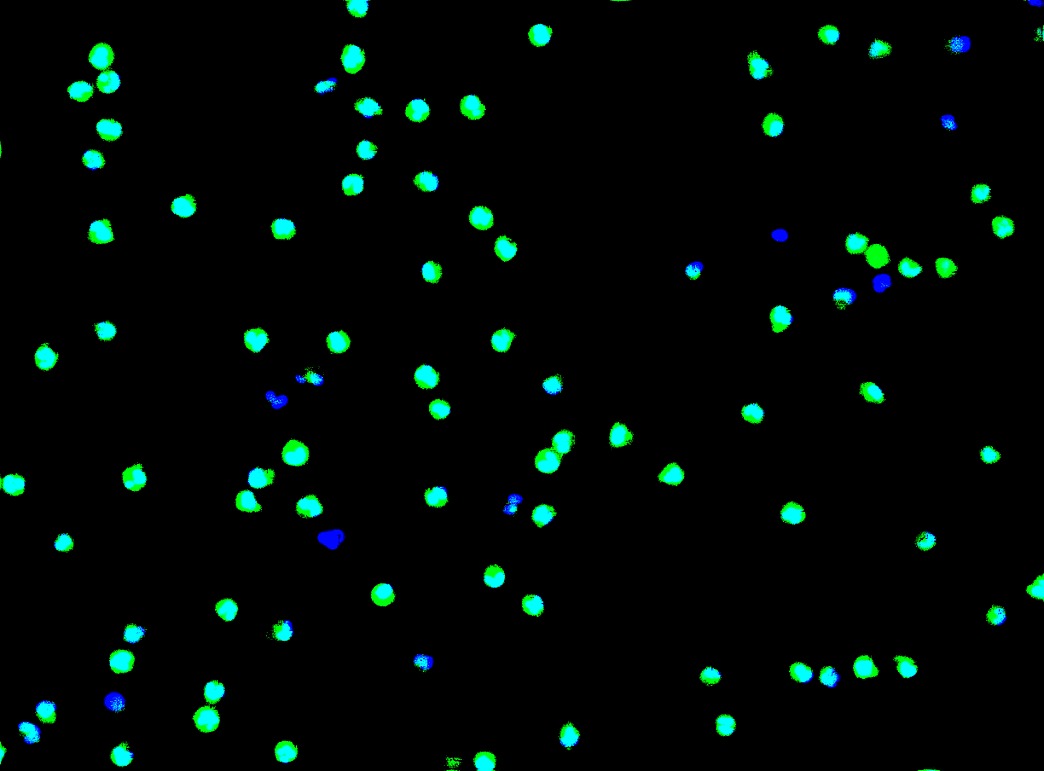

Supplement: Supplementary file 9 — Source data Fig. 3 [file 44321_2024_117_MOESM9_ESM.zip › Figure 3/3D/HRG_1.00_μM.tif]

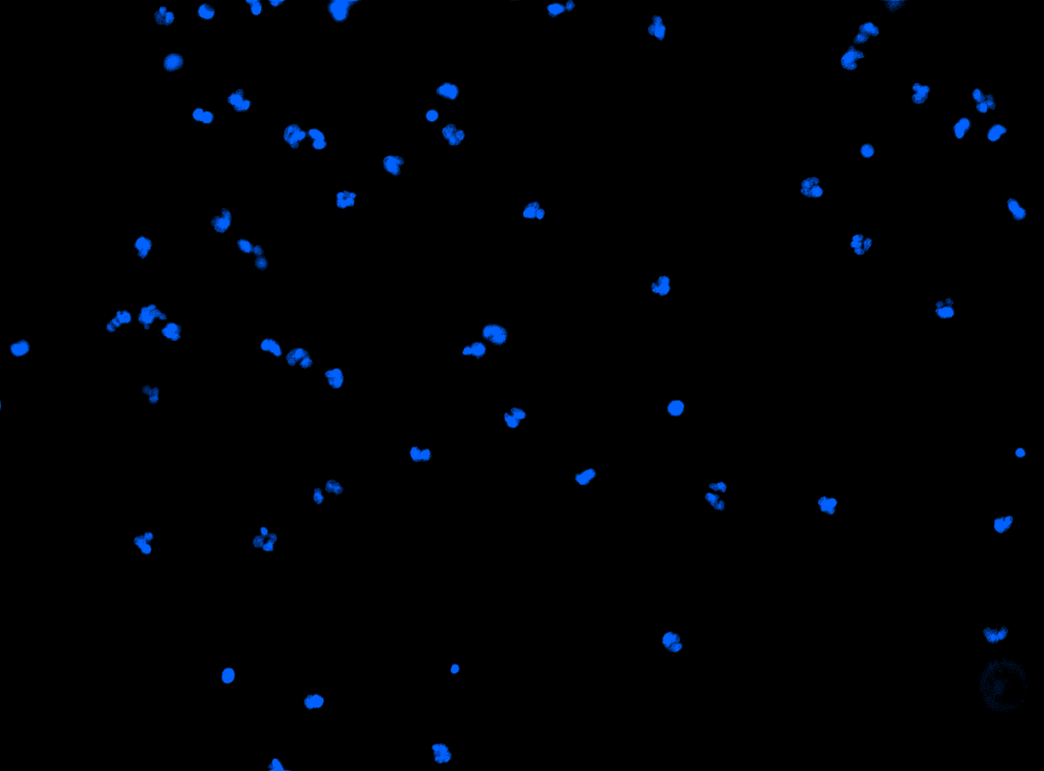

Supplement: Supplementary file 10 — Source data Fig. 4 [file 44321_2024_117_MOESM10_ESM.zip › Figure 4/4A/Control_DAPI.tif]

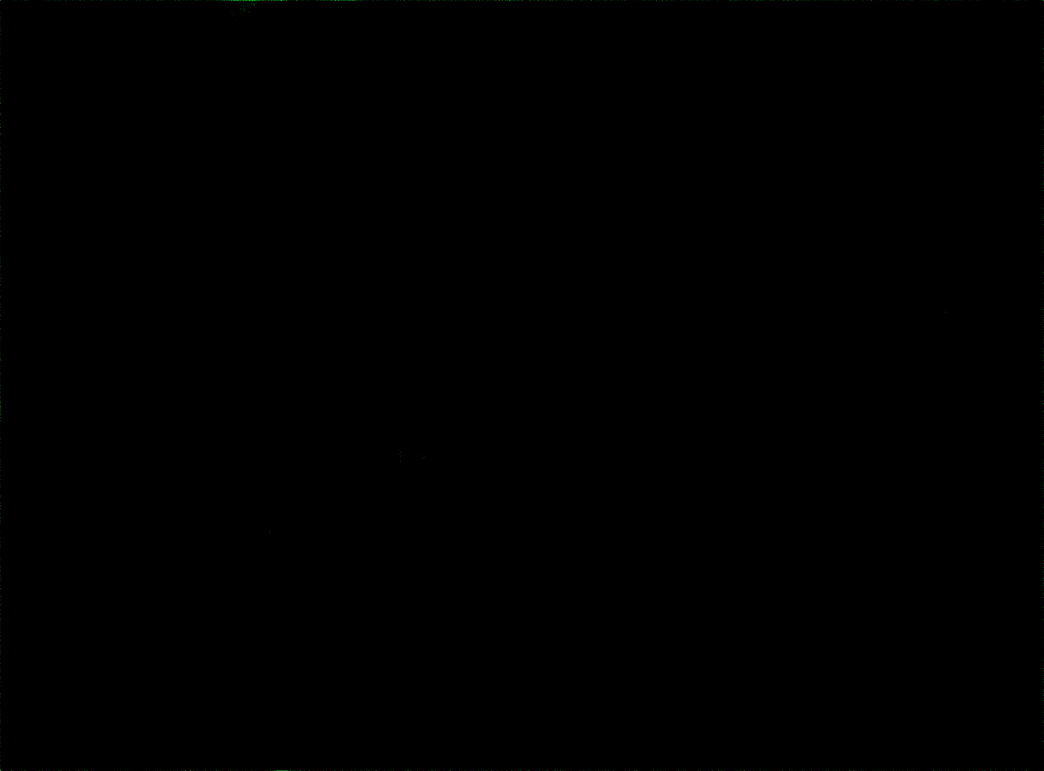

Supplement: Supplementary file 10 — Source data Fig. 4 [file 44321_2024_117_MOESM10_ESM.zip › Figure 4/4A/Control_H3-Cit.tif]

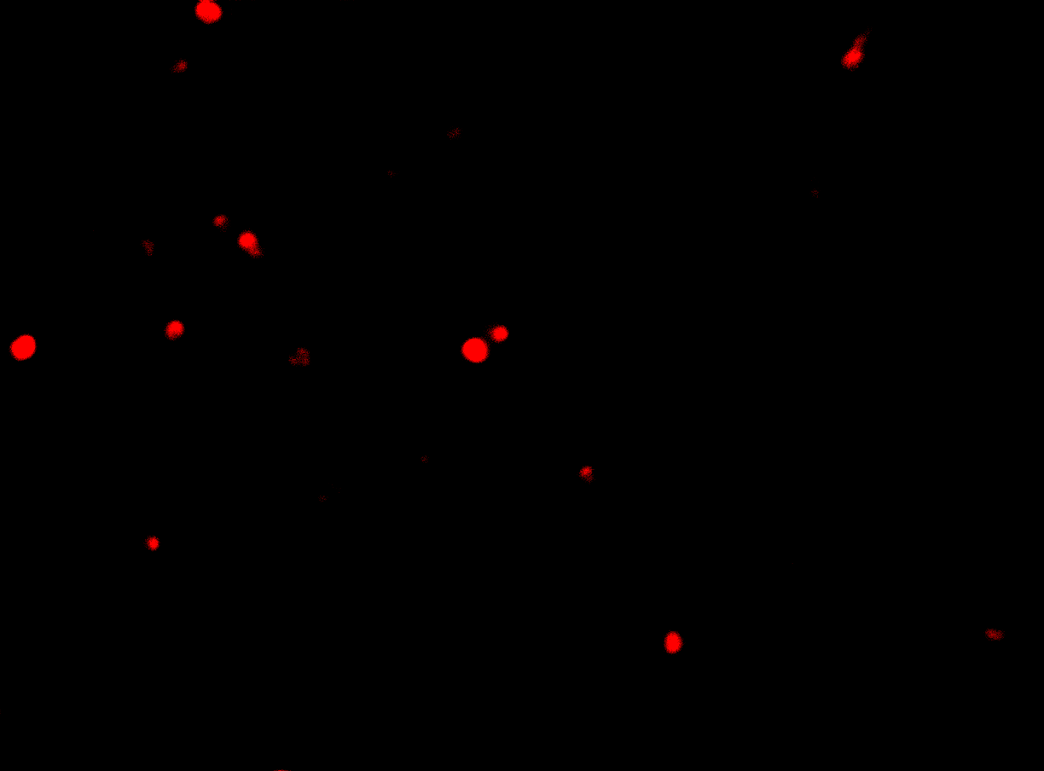

Supplement: Supplementary file 10 — Source data Fig. 4 [file 44321_2024_117_MOESM10_ESM.zip › Figure 4/4A/Control_MPO.tif]

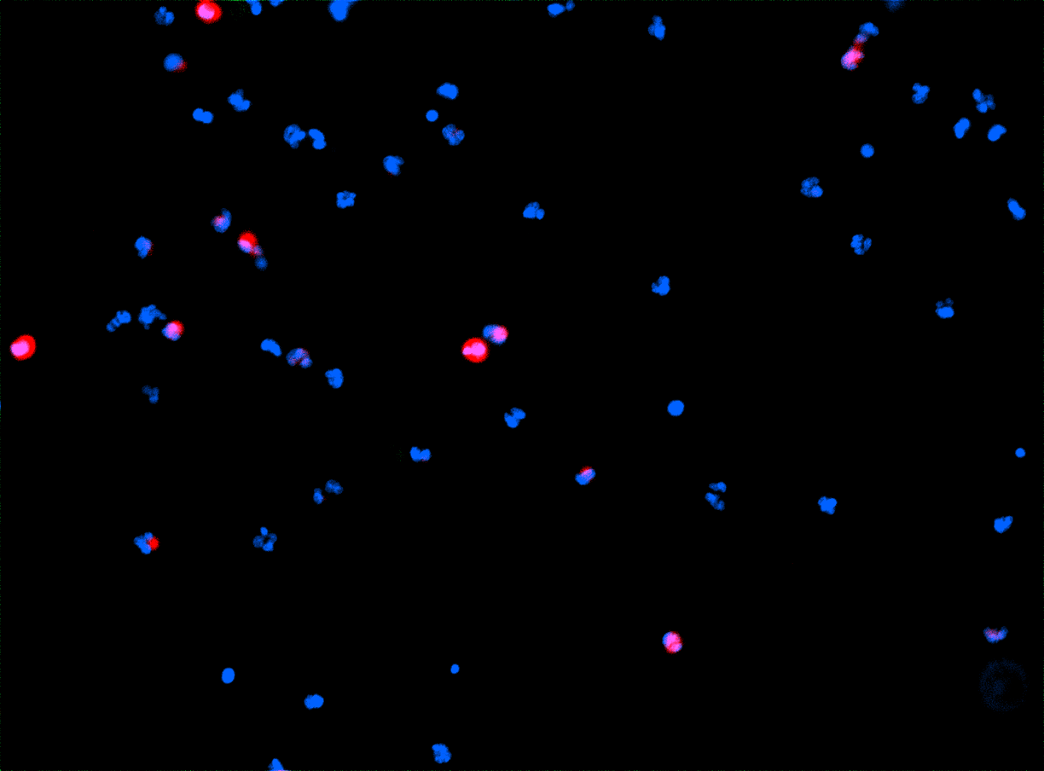

Supplement: Supplementary file 10 — Source data Fig. 4 [file 44321_2024_117_MOESM10_ESM.zip › Figure 4/4A/Control_Merge.tif]

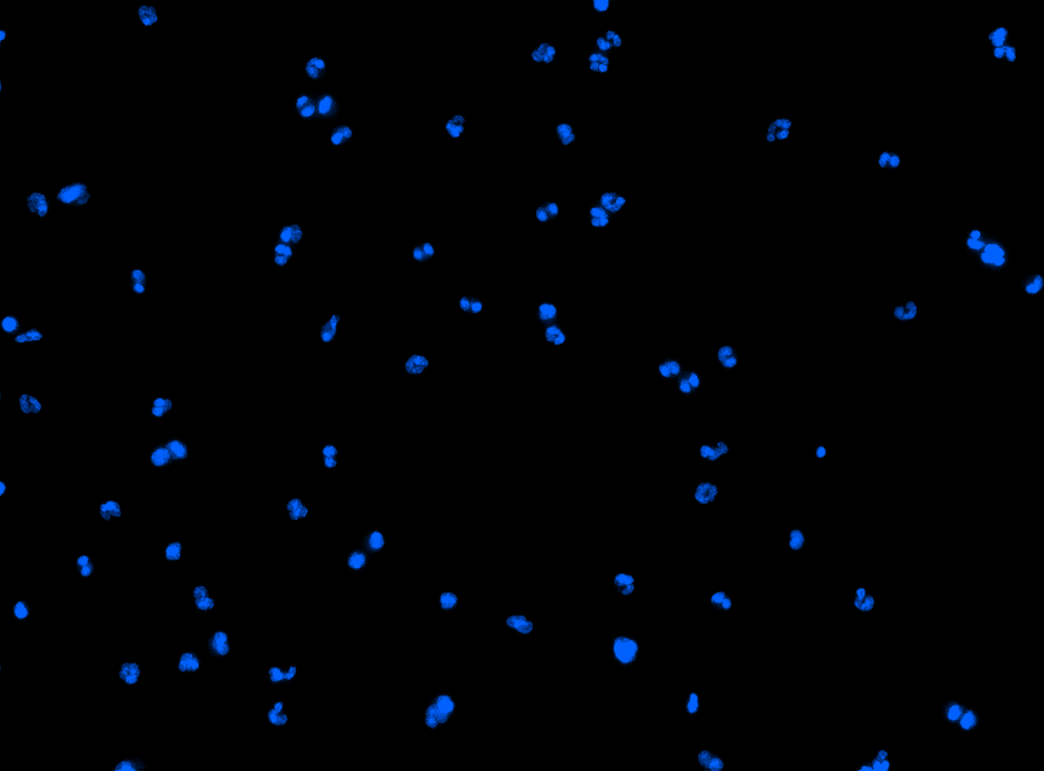

Supplement: Supplementary file 10 — Source data Fig. 4 [file 44321_2024_117_MOESM10_ESM.zip › Figure 4/4A/LPS_DAPI.tif]

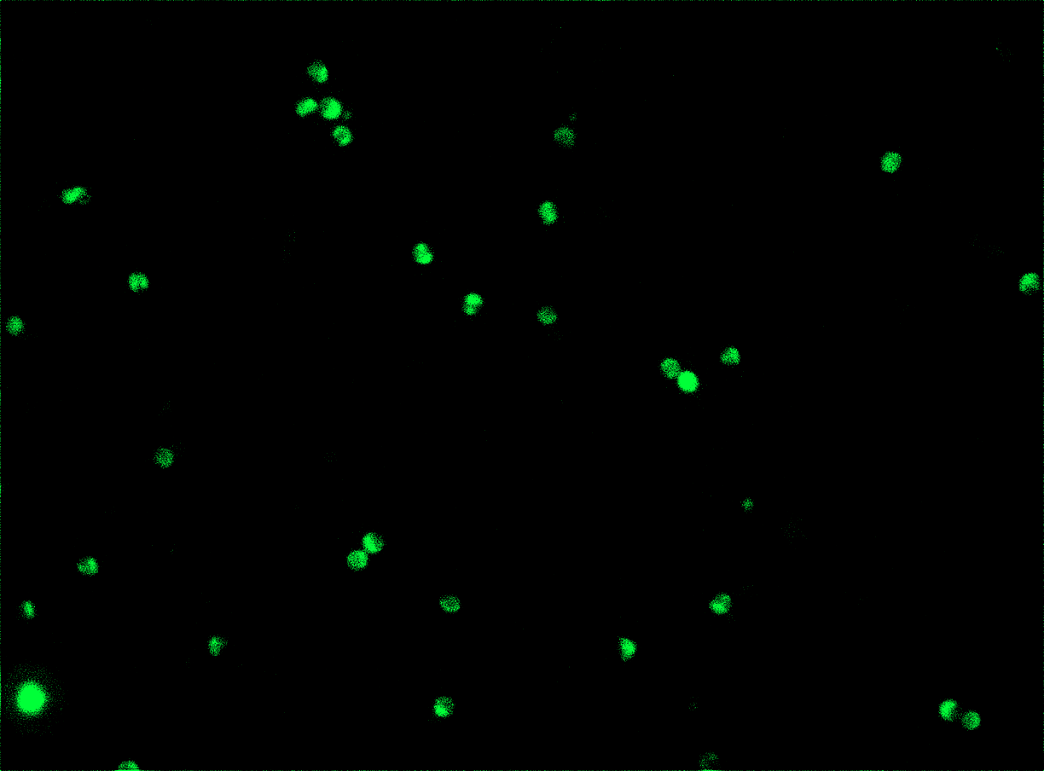

Supplement: Supplementary file 10 — Source data Fig. 4 [file 44321_2024_117_MOESM10_ESM.zip › Figure 4/4A/LPS_H3-Cit.tif]

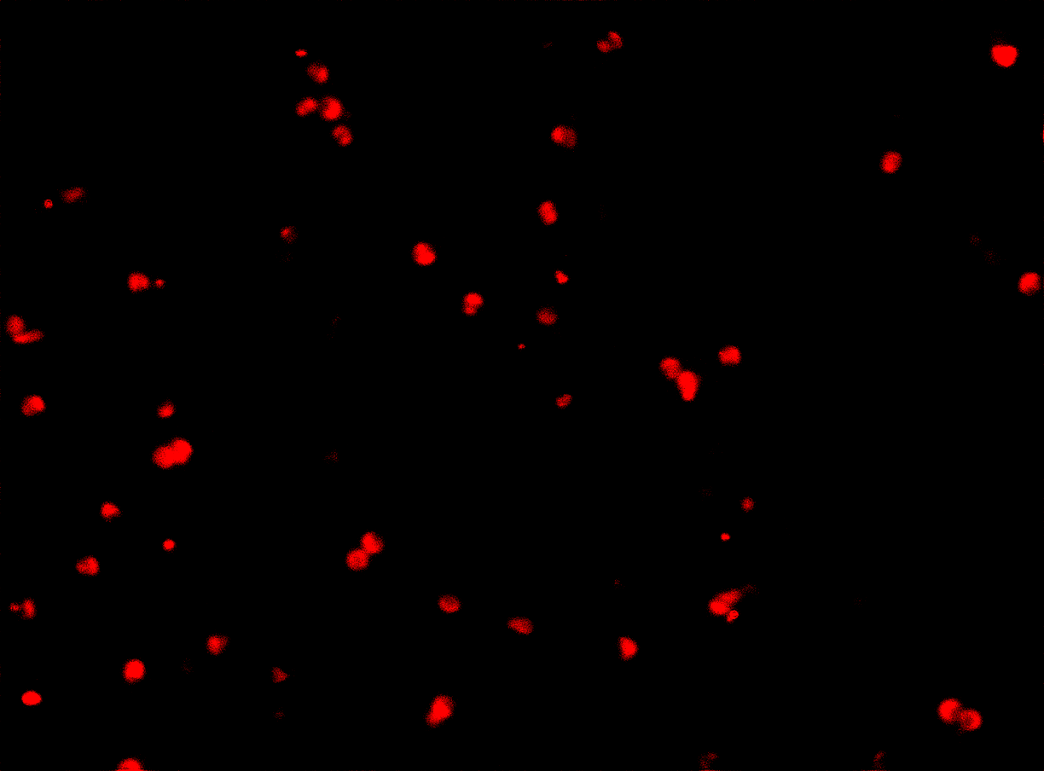

Supplement: Supplementary file 10 — Source data Fig. 4 [file 44321_2024_117_MOESM10_ESM.zip › Figure 4/4A/LPS_MPO.tif]

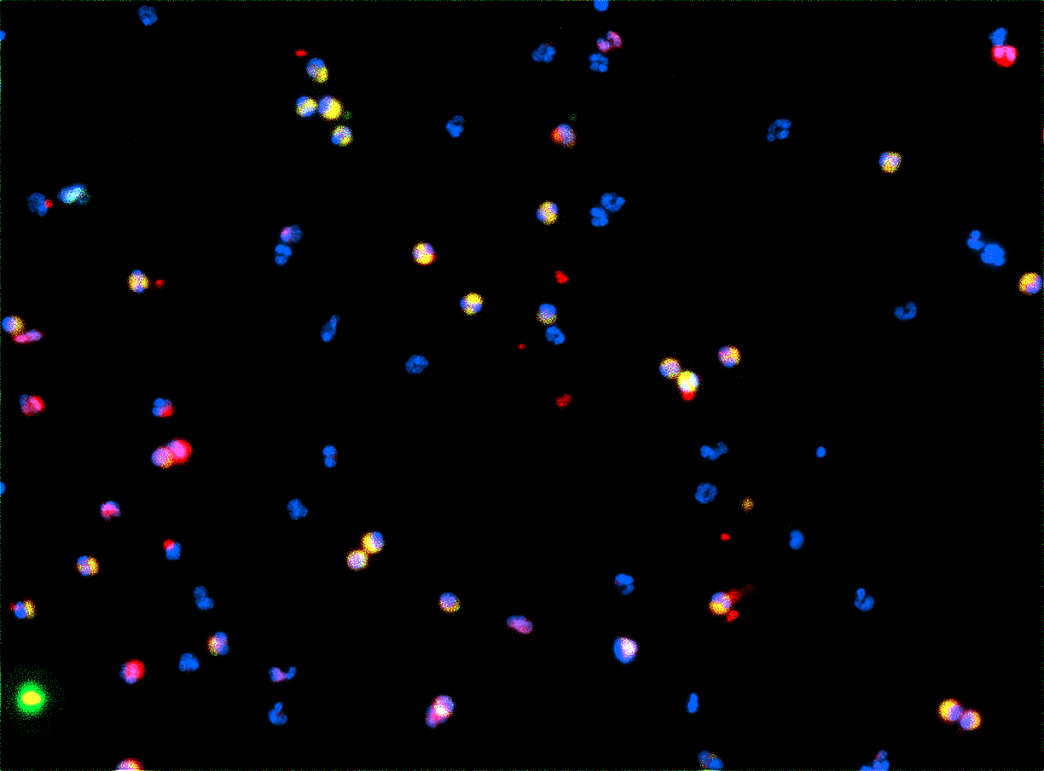

Supplement: Supplementary file 10 — Source data Fig. 4 [file 44321_2024_117_MOESM10_ESM.zip › Figure 4/4A/LPS_Merge.tif]

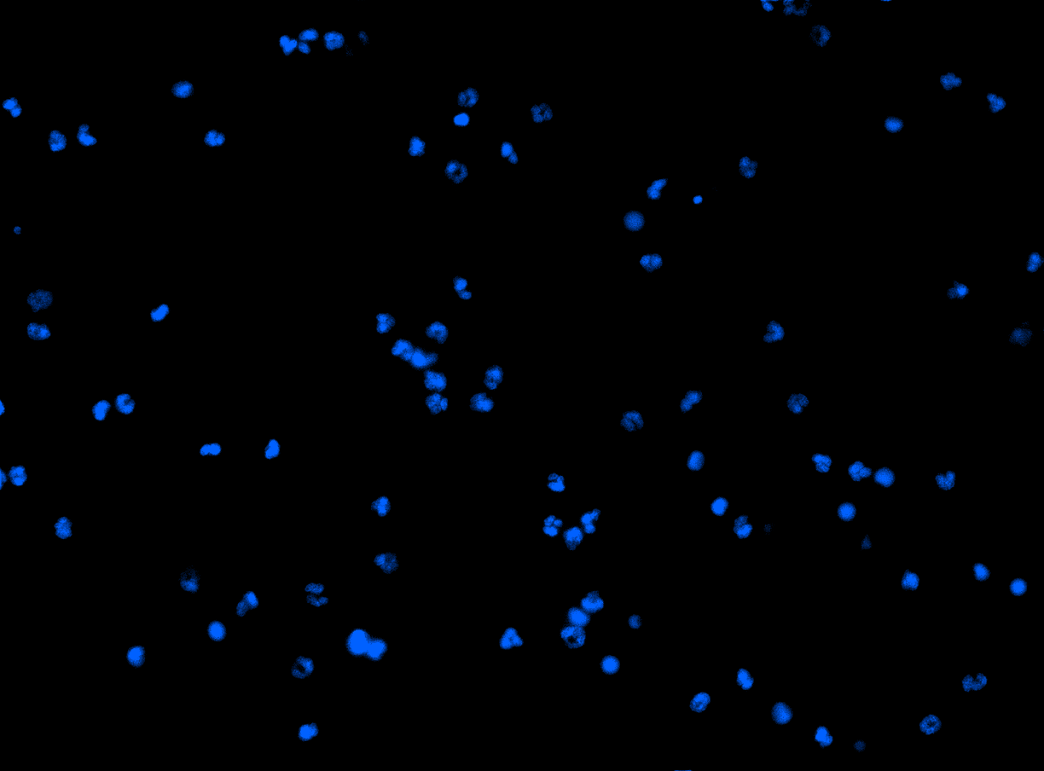

Supplement: Supplementary file 10 — Source data Fig. 4 [file 44321_2024_117_MOESM10_ESM.zip › Figure 4/4A/tPA+HRG_DAPI.tif]

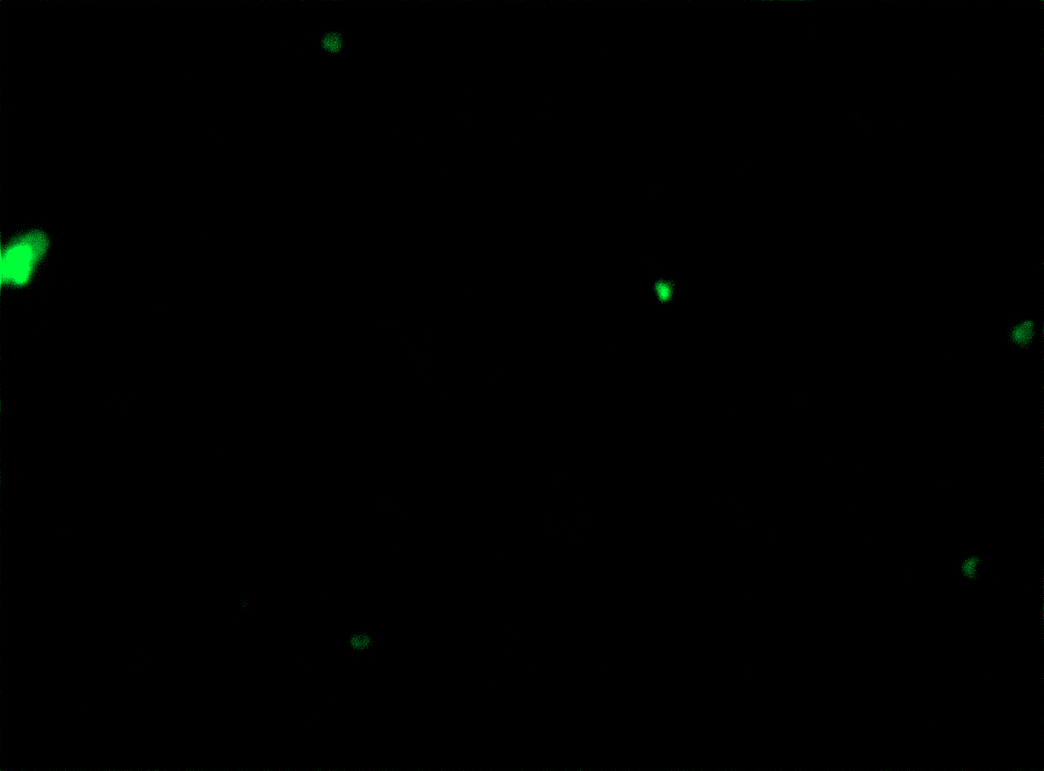

Supplement: Supplementary file 10 — Source data Fig. 4 [file 44321_2024_117_MOESM10_ESM.zip › Figure 4/4A/tPA+HRG_H3-Cit.tif]

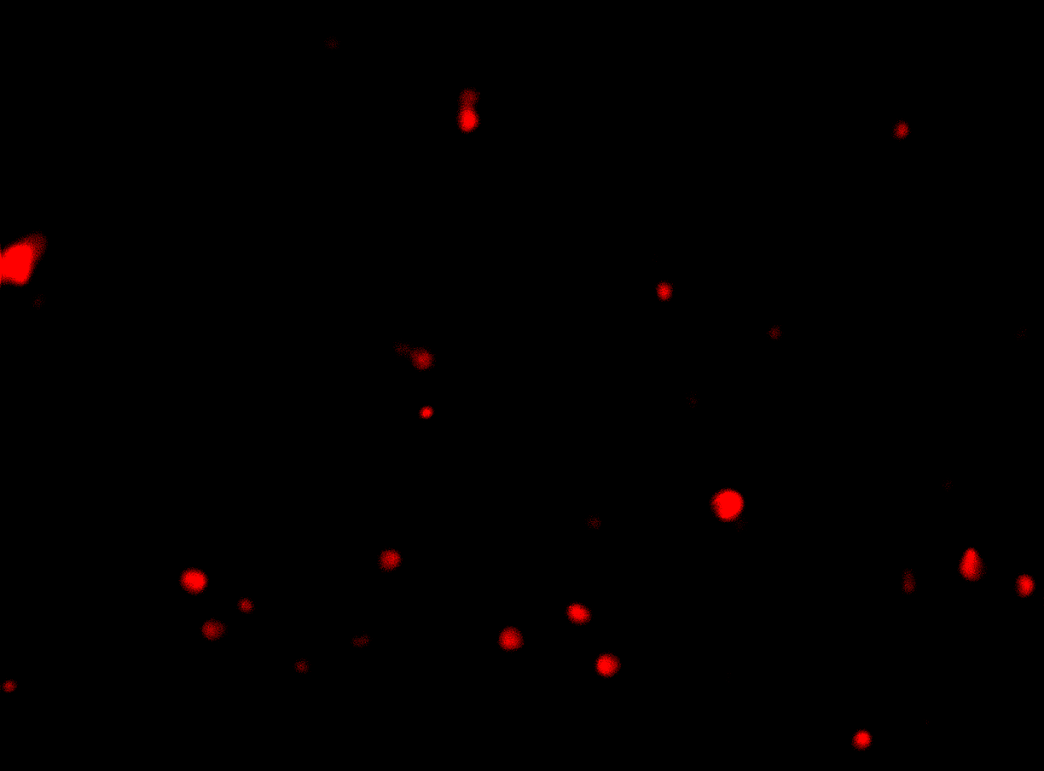

Supplement: Supplementary file 10 — Source data Fig. 4 [file 44321_2024_117_MOESM10_ESM.zip › Figure 4/4A/tPA+HRG_MPO.tif]

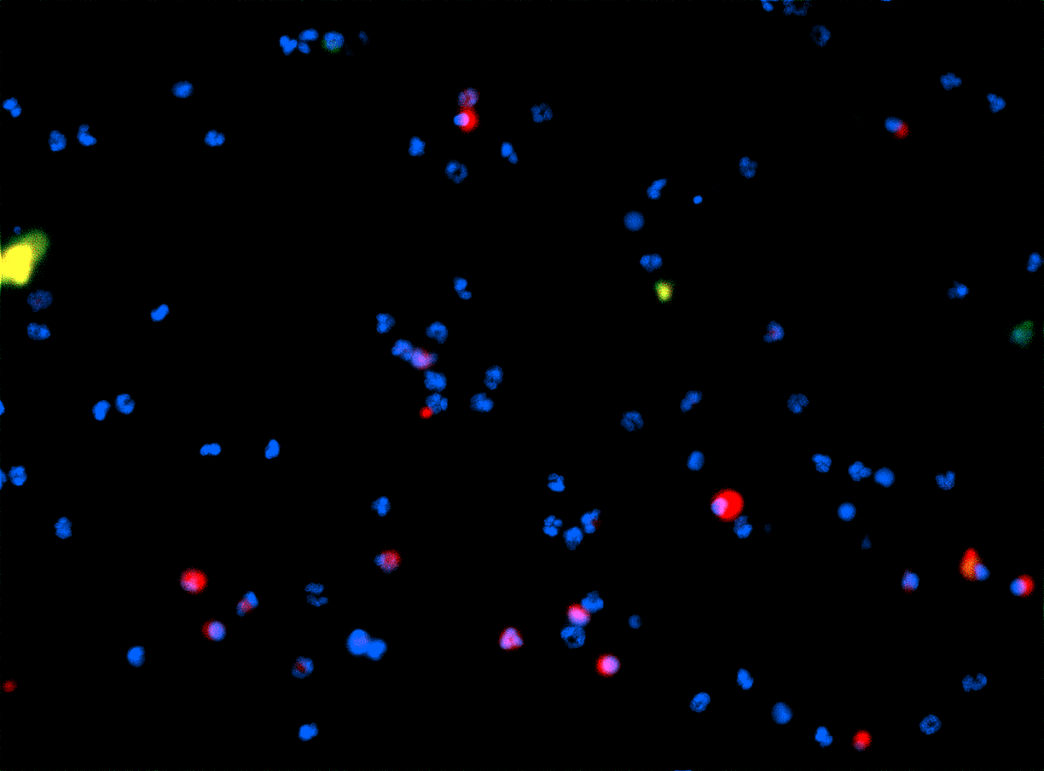

Supplement: Supplementary file 10 — Source data Fig. 4 [file 44321_2024_117_MOESM10_ESM.zip › Figure 4/4A/tPA+HRG_Merge.tif]

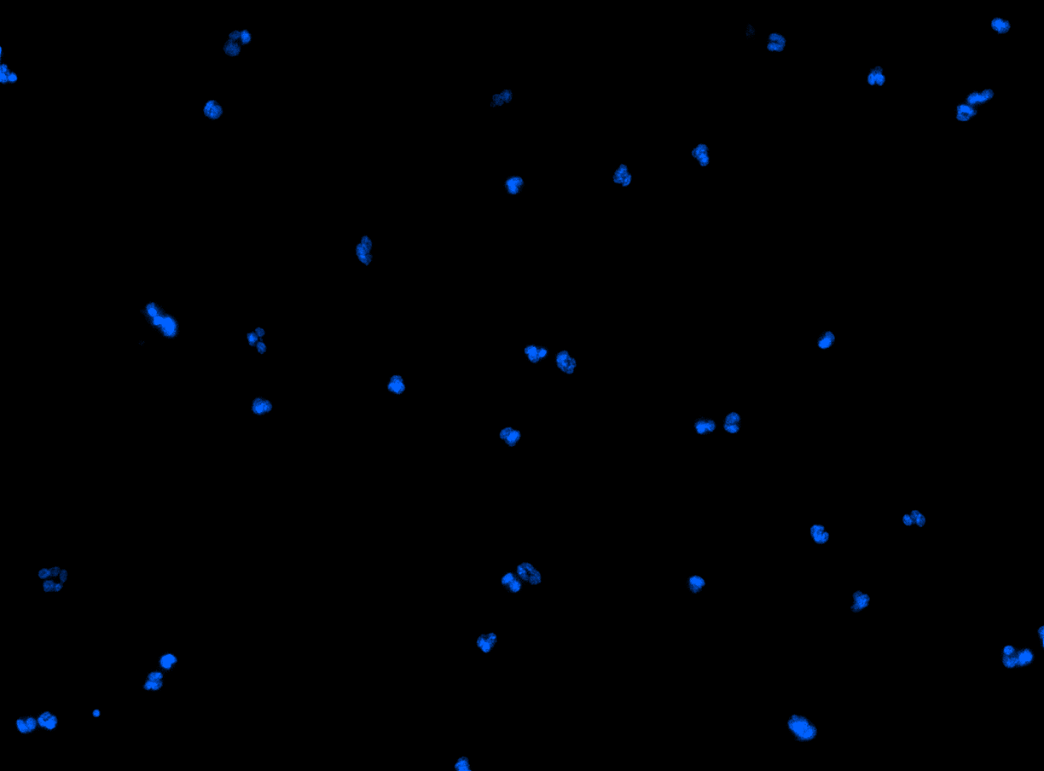

Supplement: Supplementary file 10 — Source data Fig. 4 [file 44321_2024_117_MOESM10_ESM.zip › Figure 4/4A/tPA_DAPI.tif]

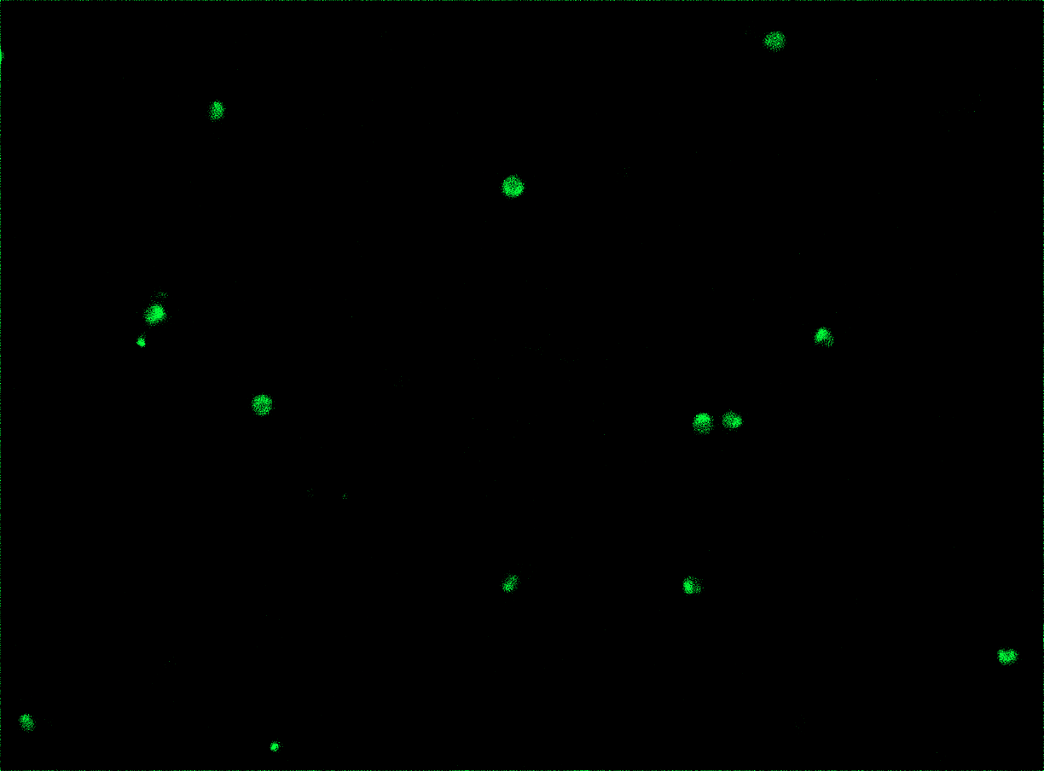

Supplement: Supplementary file 10 — Source data Fig. 4 [file 44321_2024_117_MOESM10_ESM.zip › Figure 4/4A/tPA_H3-Cit.tif]

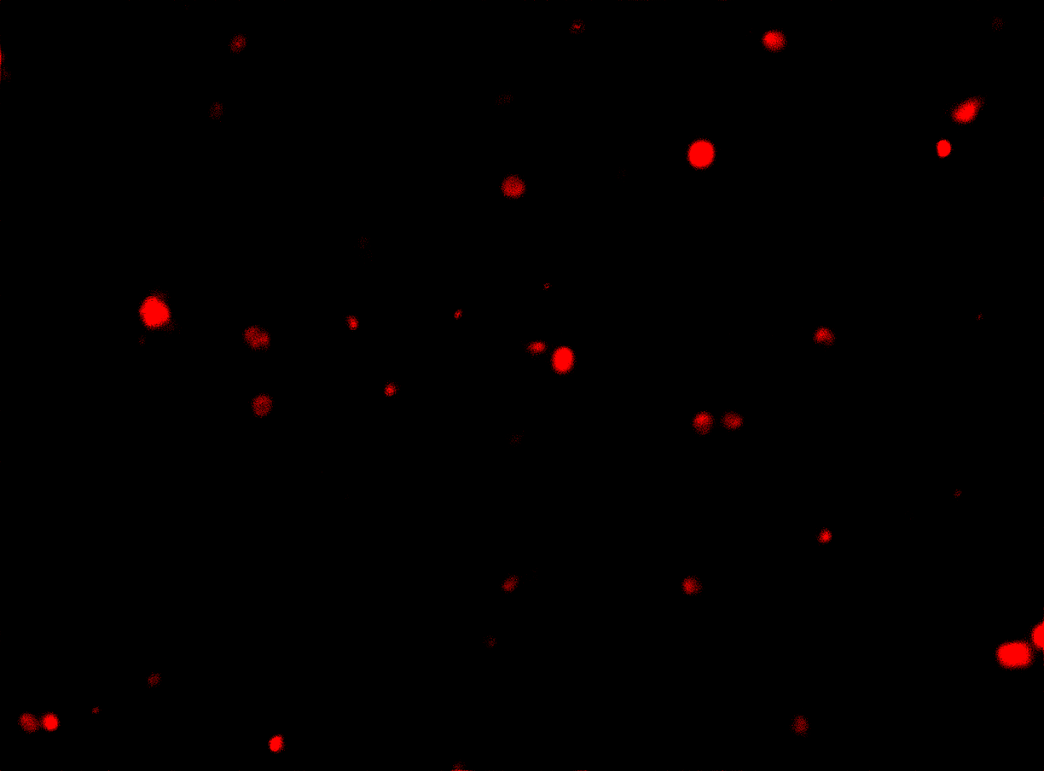

Supplement: Supplementary file 10 — Source data Fig. 4 [file 44321_2024_117_MOESM10_ESM.zip › Figure 4/4A/tPA_MPO.tif]

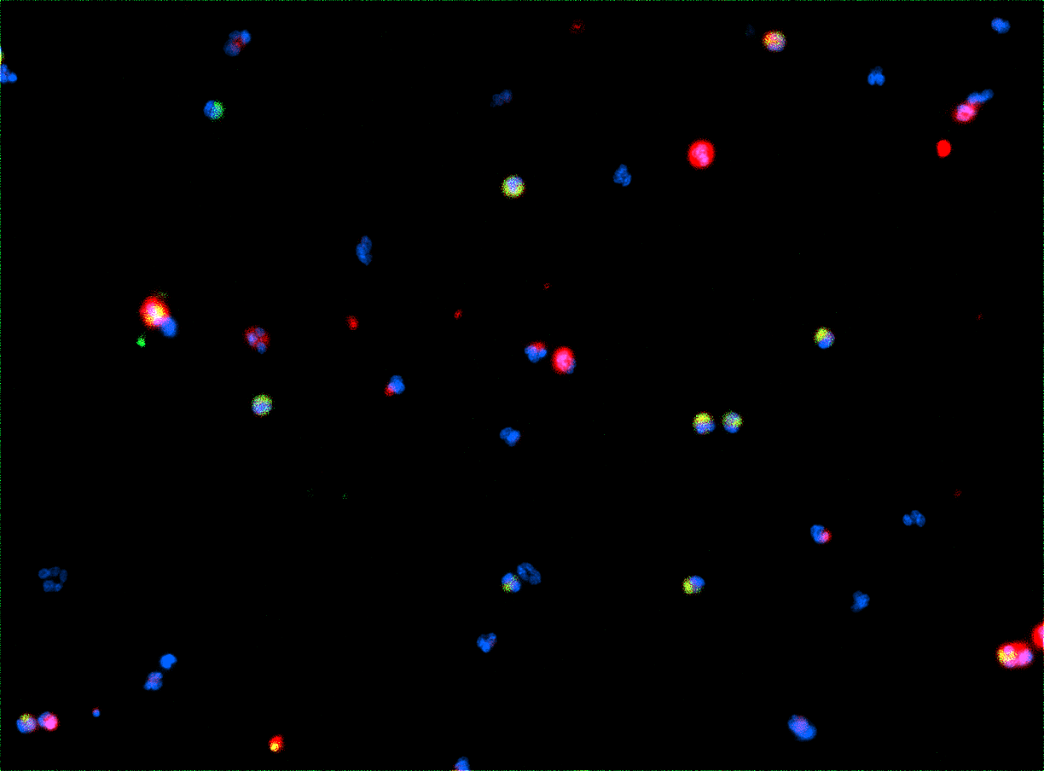

Supplement: Supplementary file 10 — Source data Fig. 4 [file 44321_2024_117_MOESM10_ESM.zip › Figure 4/4A/tPA_Merge.tif]

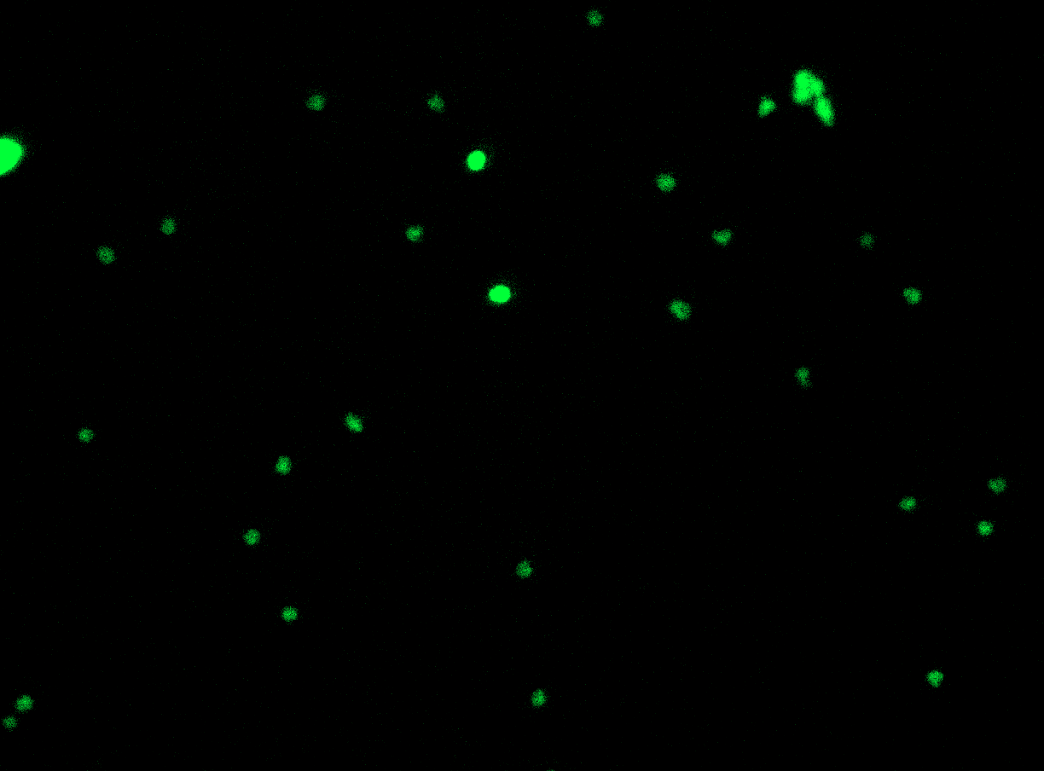

Supplement: Supplementary file 11 — Source data Fig. 5 [file 44321_2024_117_MOESM11_ESM.zip › Figure 5/5A/Control_F-actin.tif]

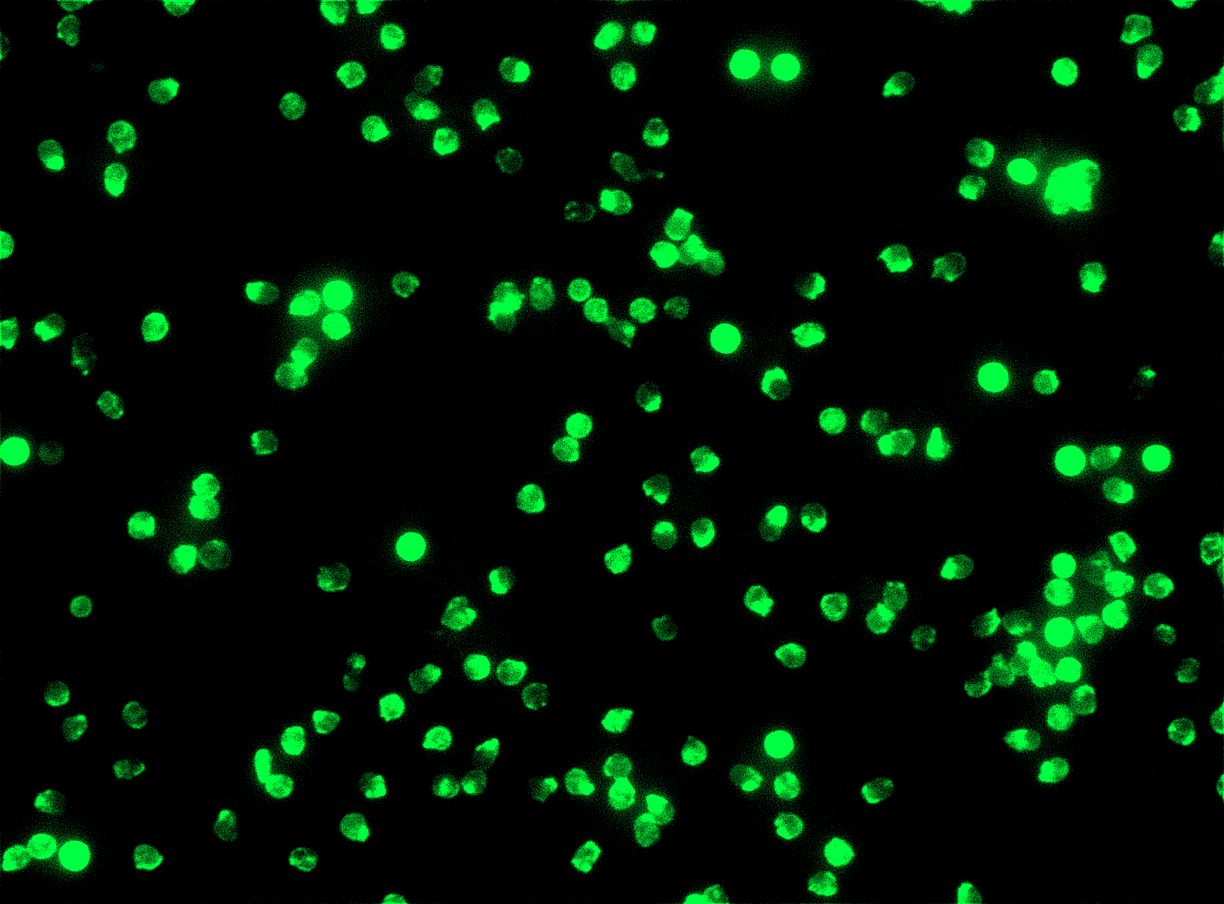

Supplement: Supplementary file 11 — Source data Fig. 5 [file 44321_2024_117_MOESM11_ESM.zip › Figure 5/5A/LPS_F-actin.tif]

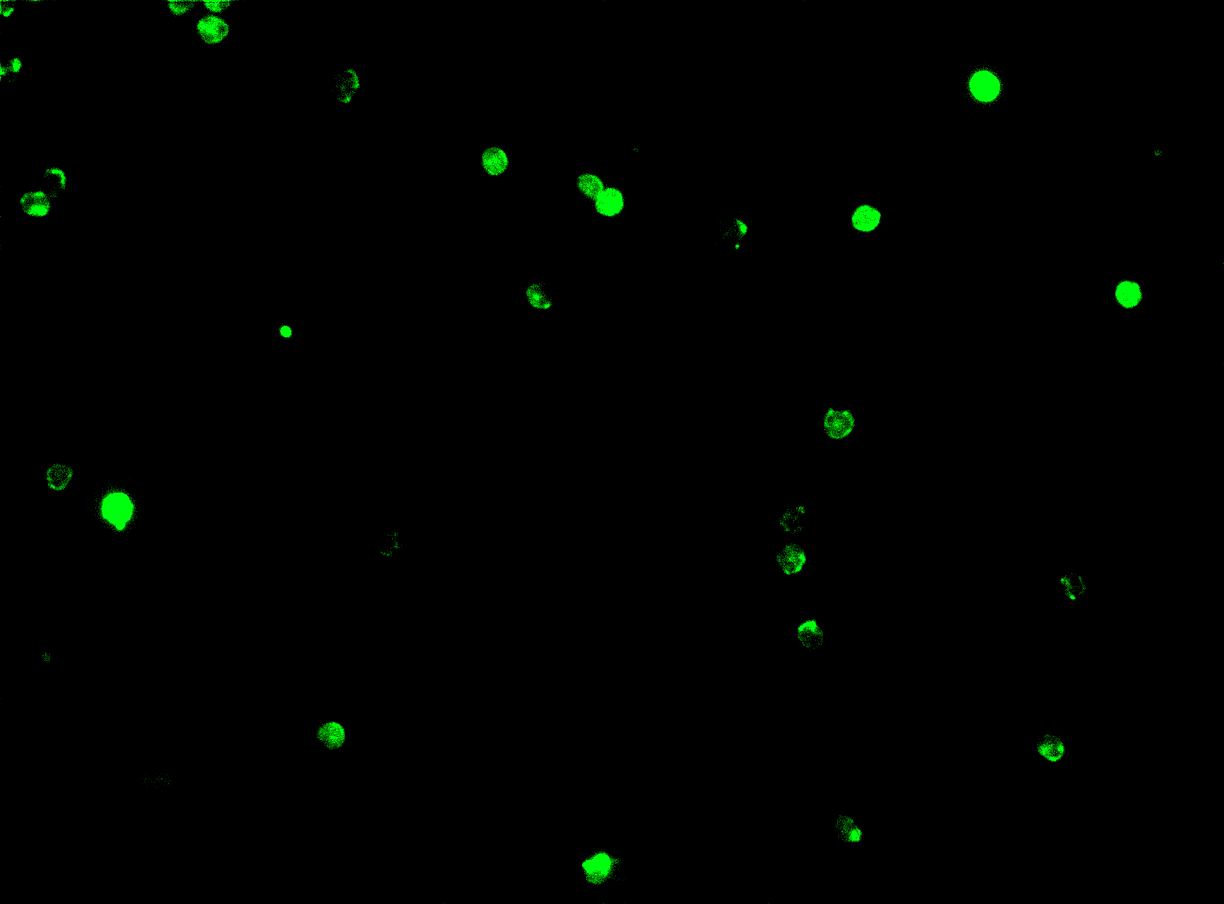

Supplement: Supplementary file 11 — Source data Fig. 5 [file 44321_2024_117_MOESM11_ESM.zip › Figure 5/5A/tPA+HRG_F-actin.tif]

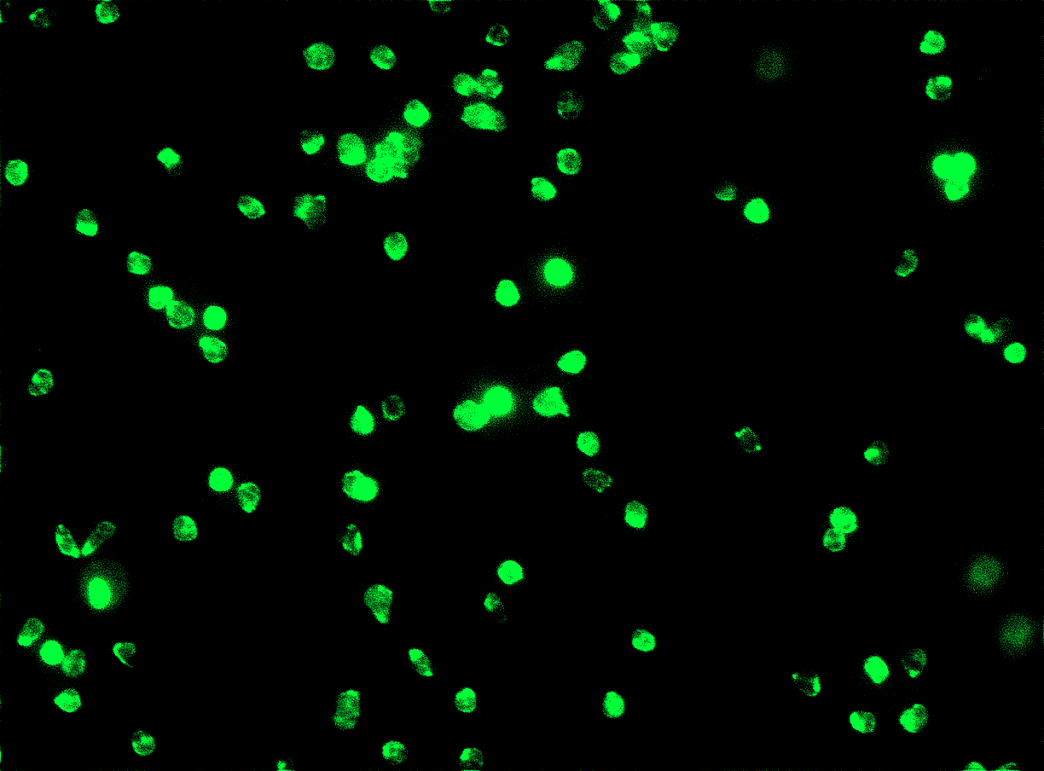

Supplement: Supplementary file 11 — Source data Fig. 5 [file 44321_2024_117_MOESM11_ESM.zip › Figure 5/5A/tPA_F-actin.tif]

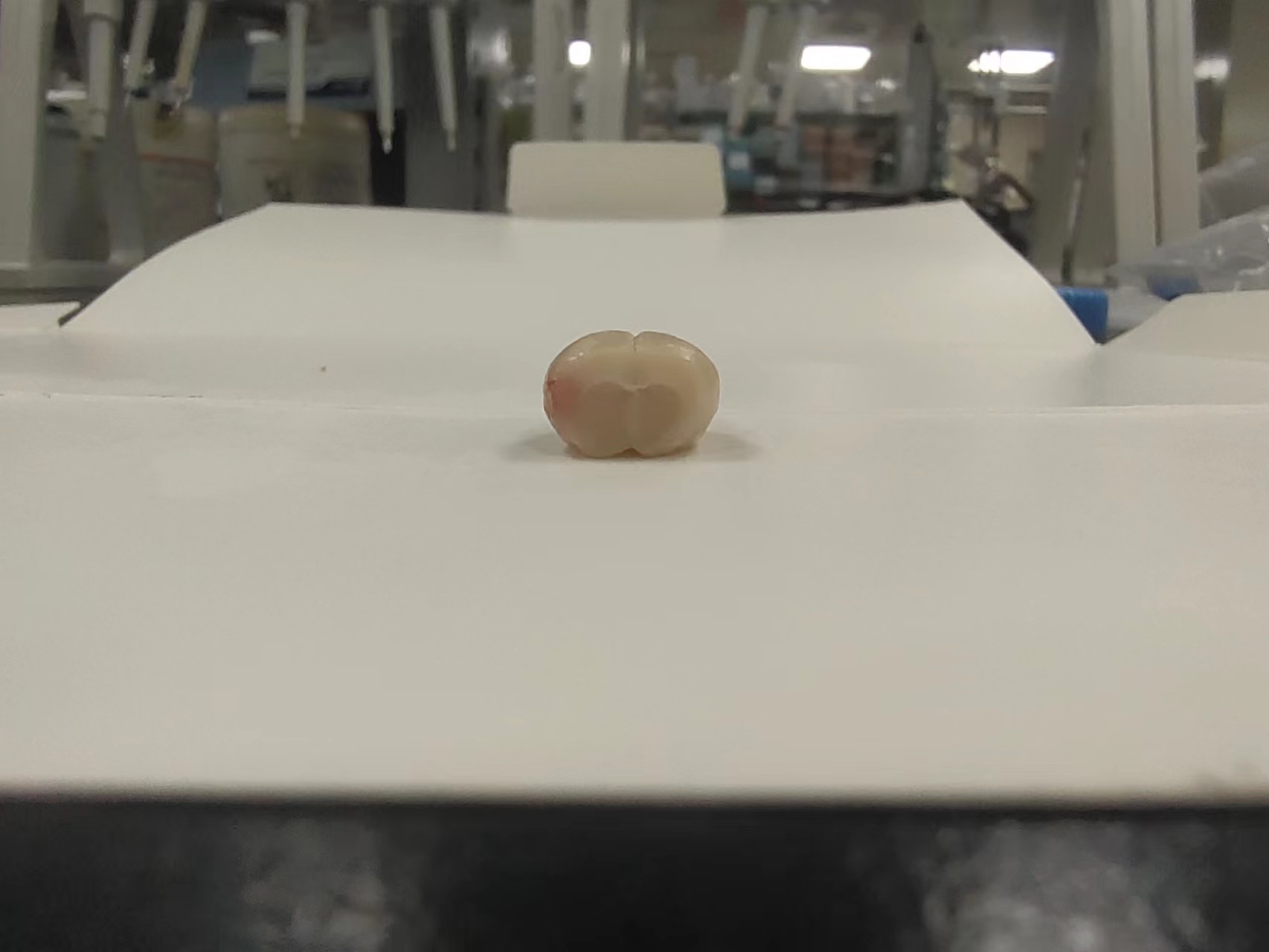

Supplement: Supplementary file 12 — Source data Fig. 6 [file 44321_2024_117_MOESM12_ESM.zip › Figure 6/6E/1h_tPA_coronal.jpg]

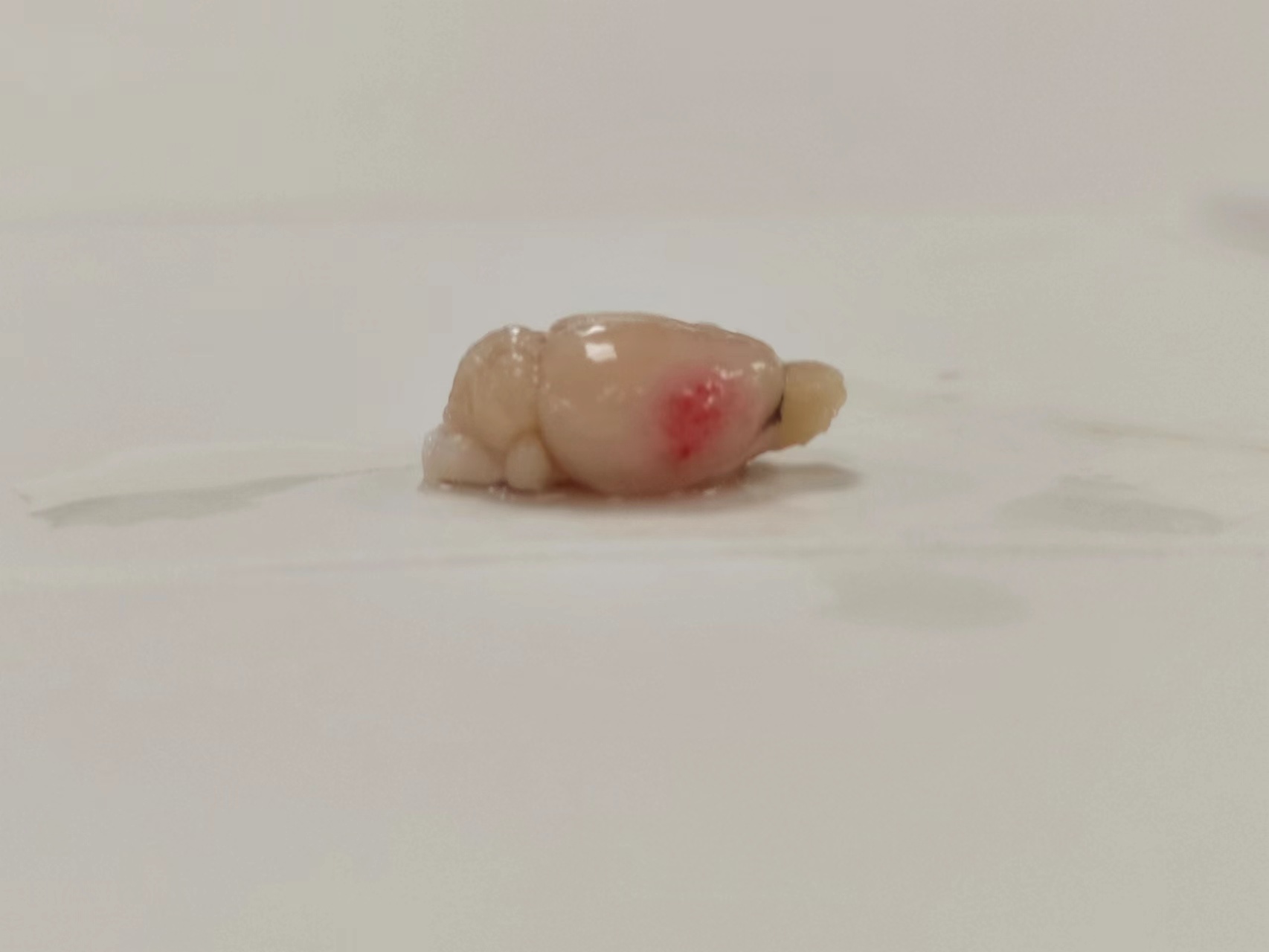

Supplement: Supplementary file 12 — Source data Fig. 6 [file 44321_2024_117_MOESM12_ESM.zip › Figure 6/6E/1h_tPA_dorsal.jpg]

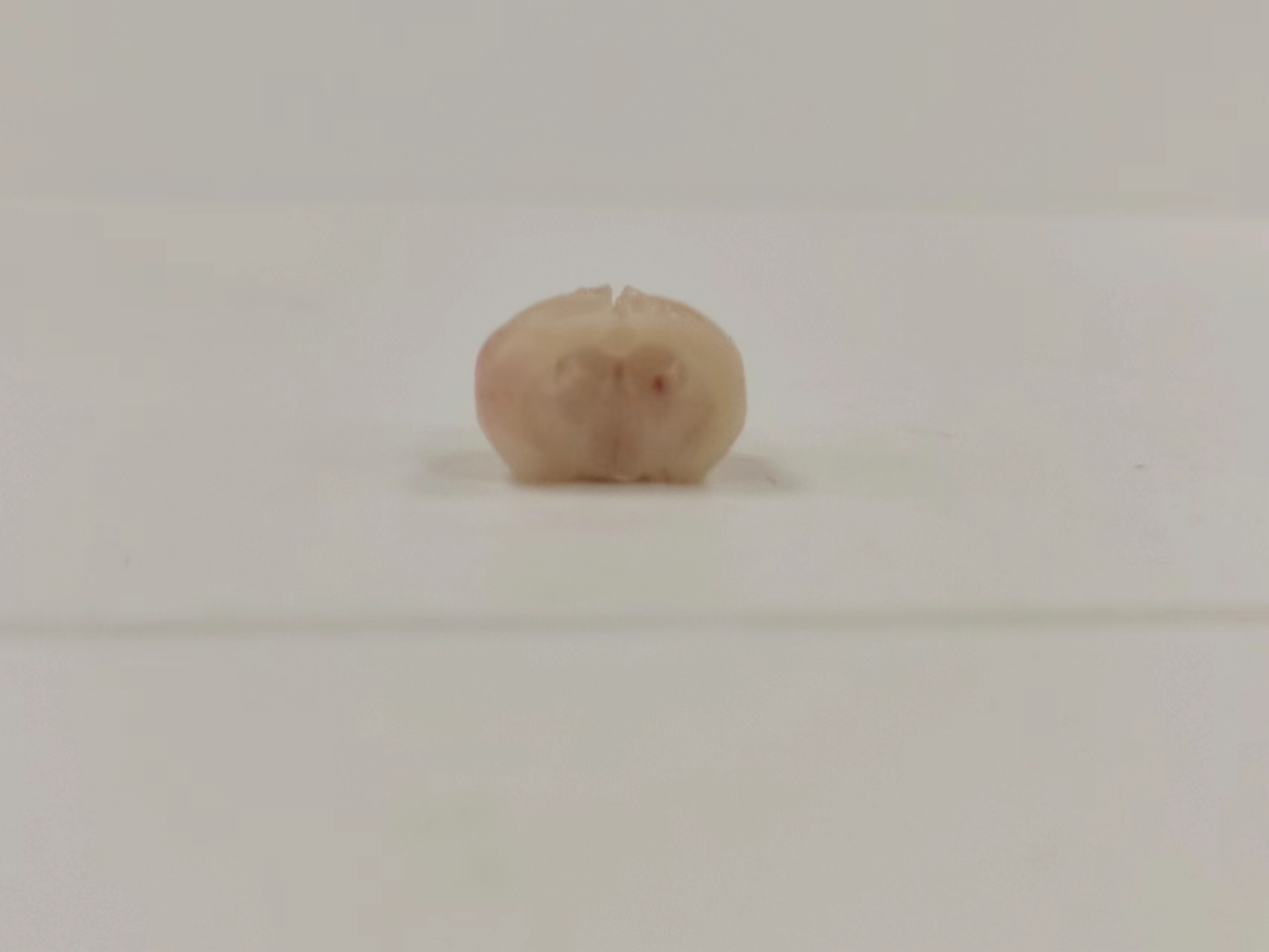

Supplement: Supplementary file 12 — Source data Fig. 6 [file 44321_2024_117_MOESM12_ESM.zip › Figure 6/6E/5h_tPA+HRG_coronal.jpg]

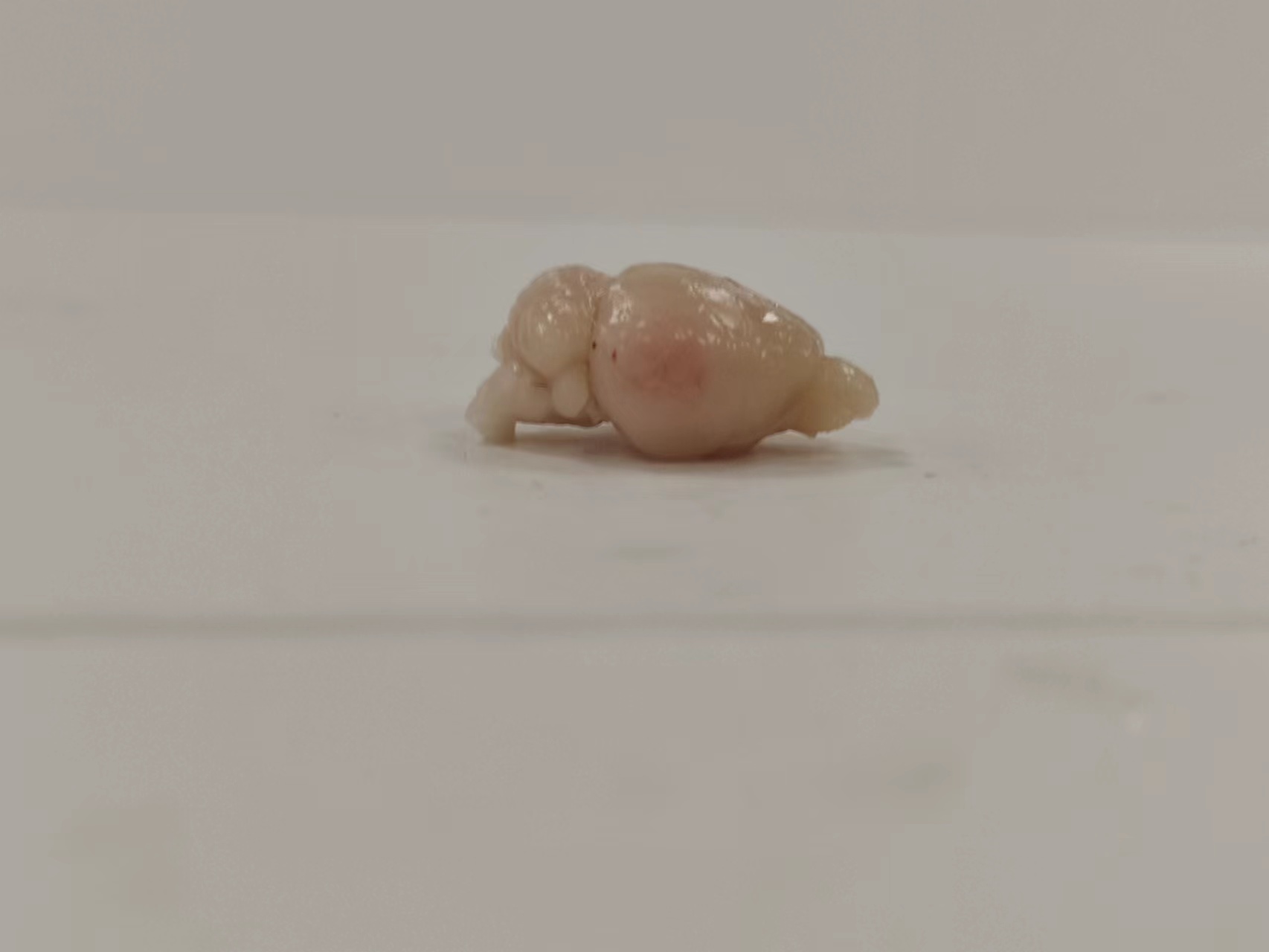

Supplement: Supplementary file 12 — Source data Fig. 6 [file 44321_2024_117_MOESM12_ESM.zip › Figure 6/6E/5h_tPA+HRG_dorsal.jpg]

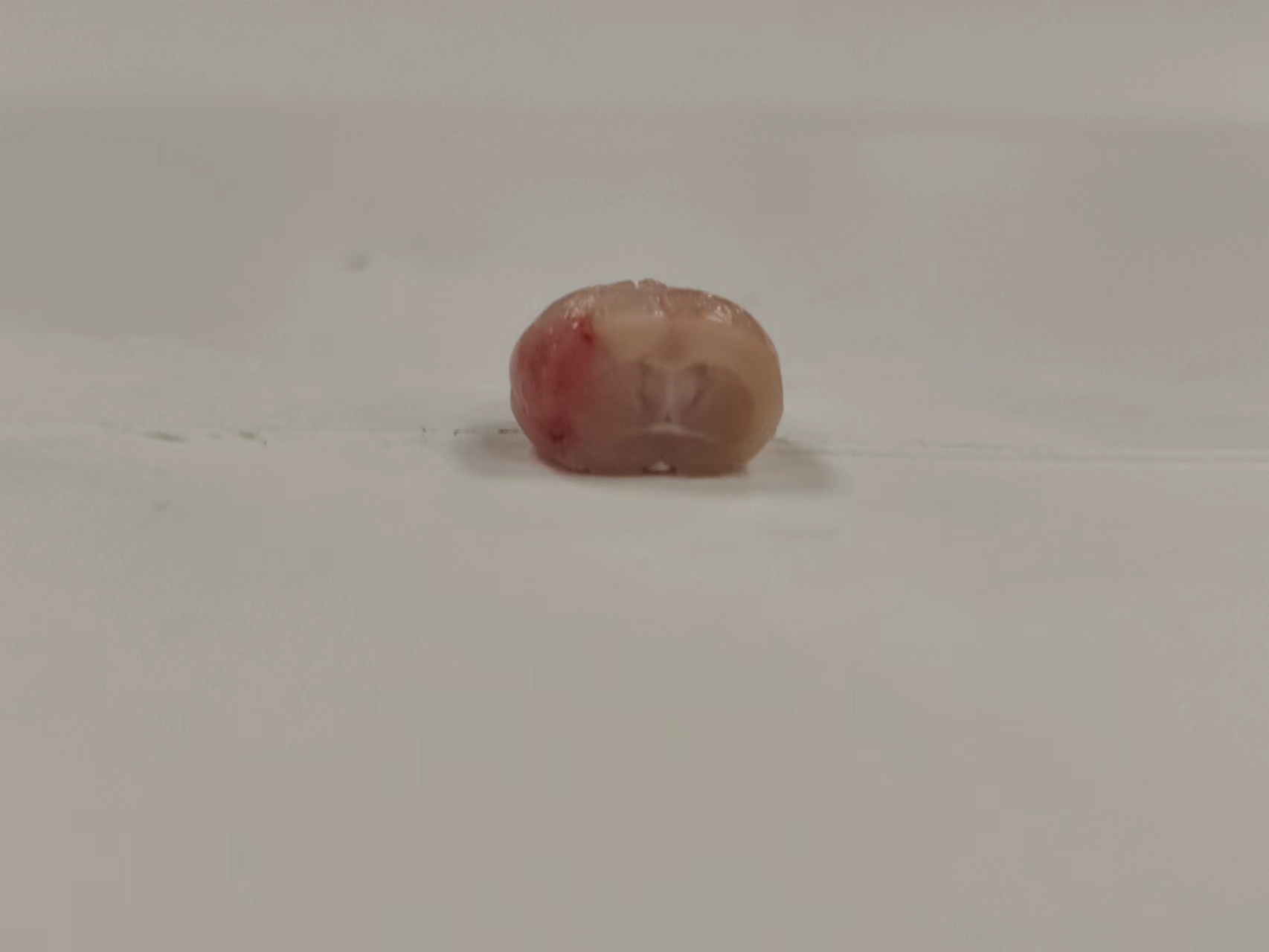

Supplement: Supplementary file 12 — Source data Fig. 6 [file 44321_2024_117_MOESM12_ESM.zip › Figure 6/6E/5h_tPA_coronal.jpg]

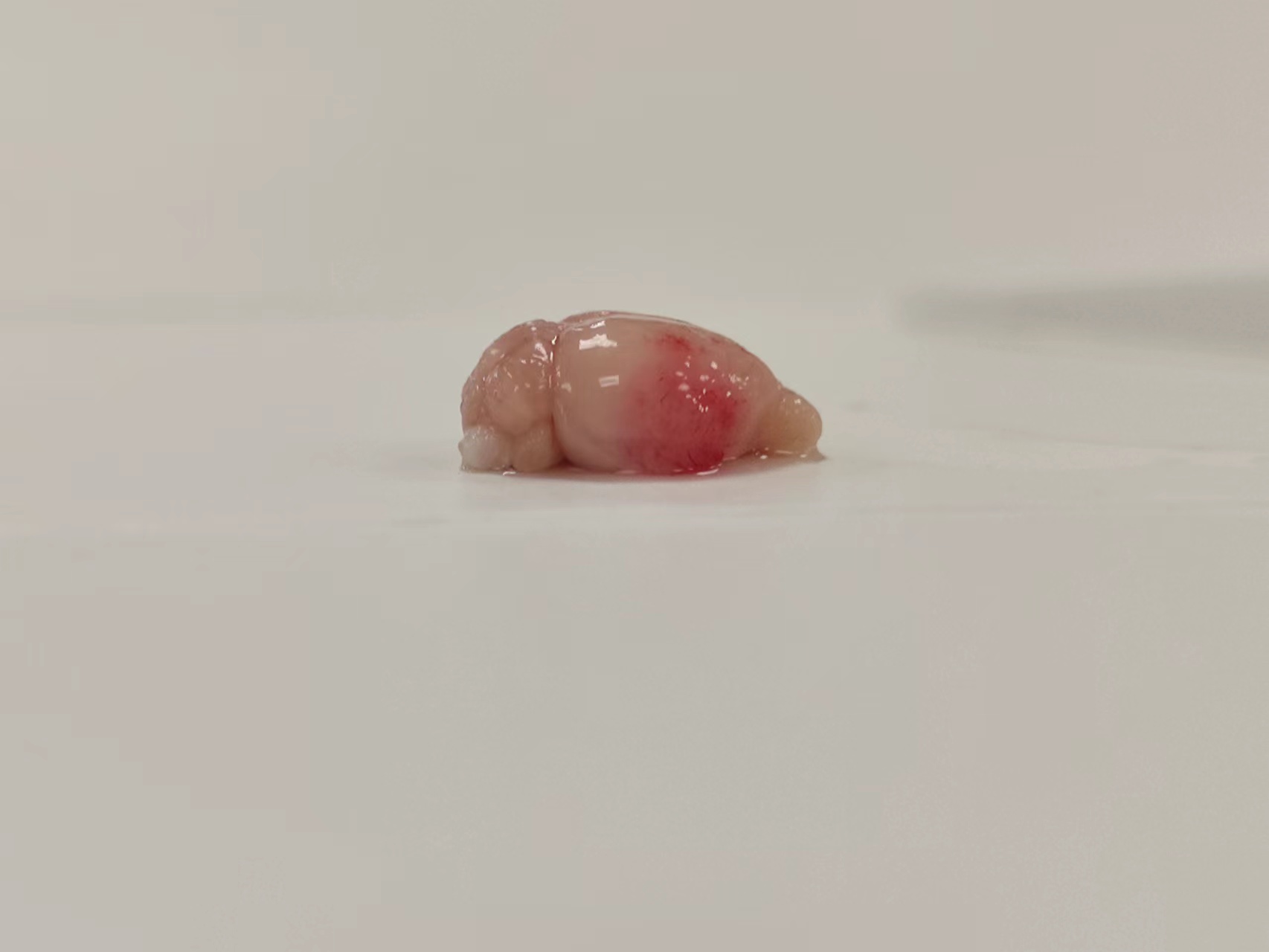

Supplement: Supplementary file 12 — Source data Fig. 6 [file 44321_2024_117_MOESM12_ESM.zip › Figure 6/6E/5h_tPA_dorsal.jpg]

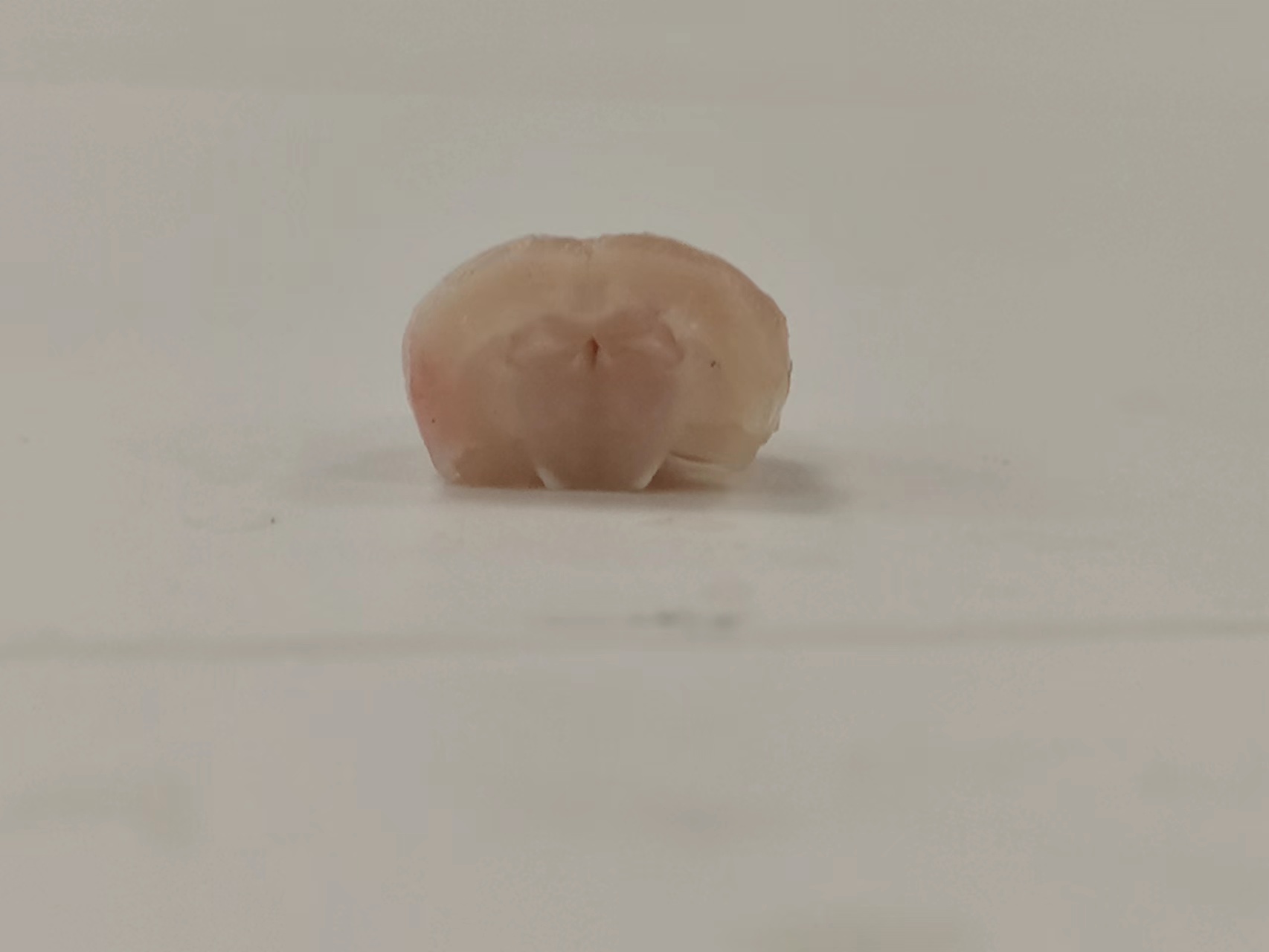

Supplement: Supplementary file 12 — Source data Fig. 6 [file 44321_2024_117_MOESM12_ESM.zip › Figure 6/6E/Vehicle_coronal.jpg]

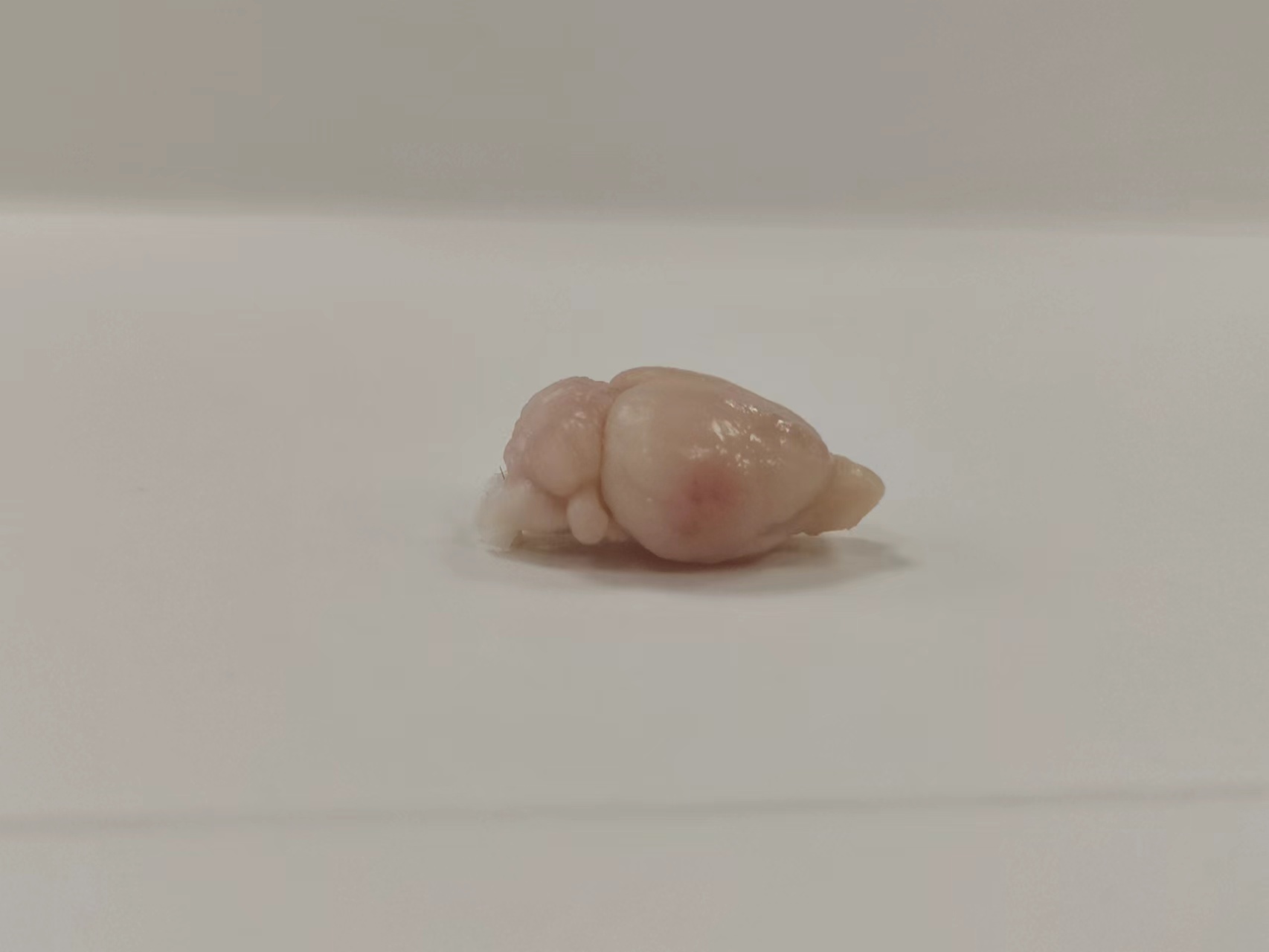

Supplement: Supplementary file 12 — Source data Fig. 6 [file 44321_2024_117_MOESM12_ESM.zip › Figure 6/6E/Vehicle_dorsal.jpg]

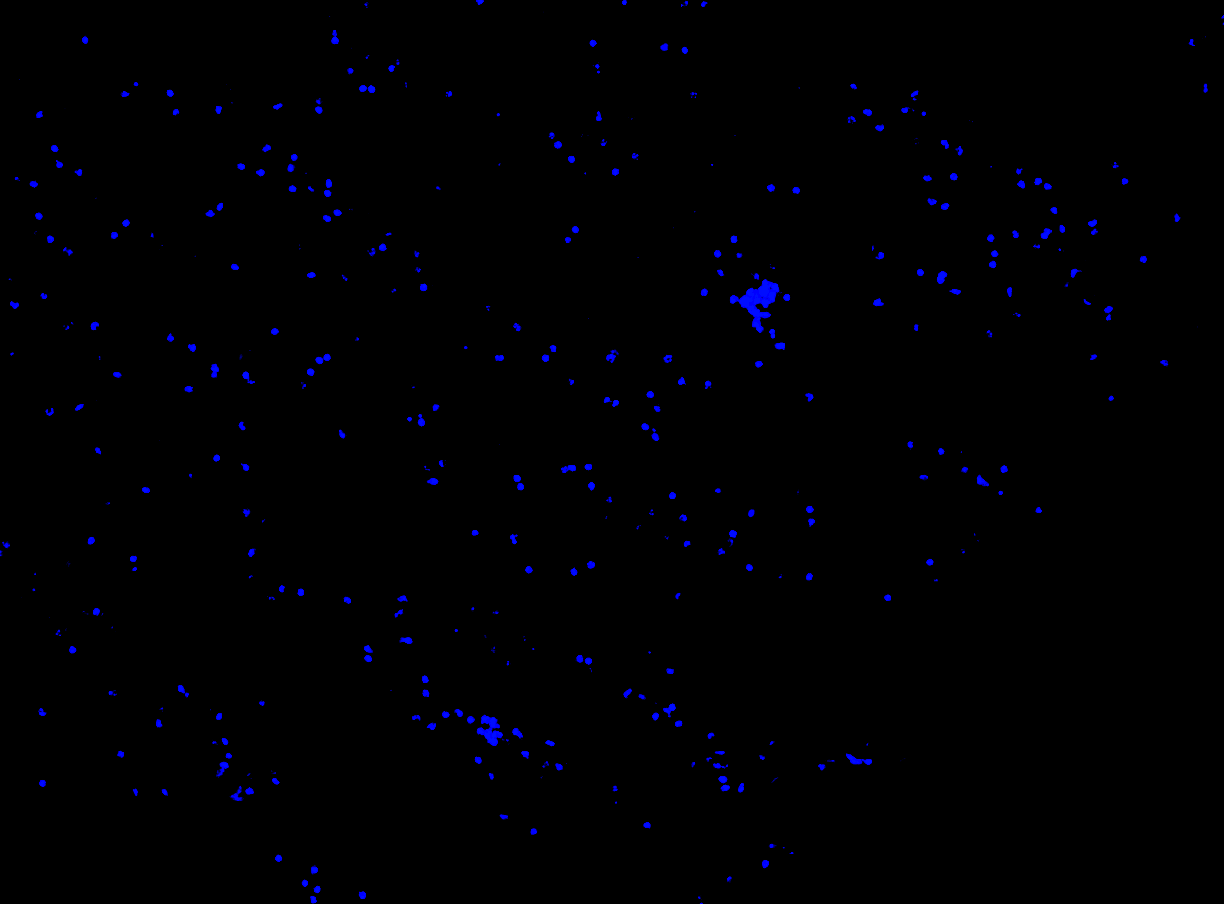

Supplement: Supplementary file 12 — Source data Fig. 6 [file 44321_2024_117_MOESM12_ESM.zip › Figure 6/6G/1h tPA_DAPI.tif]

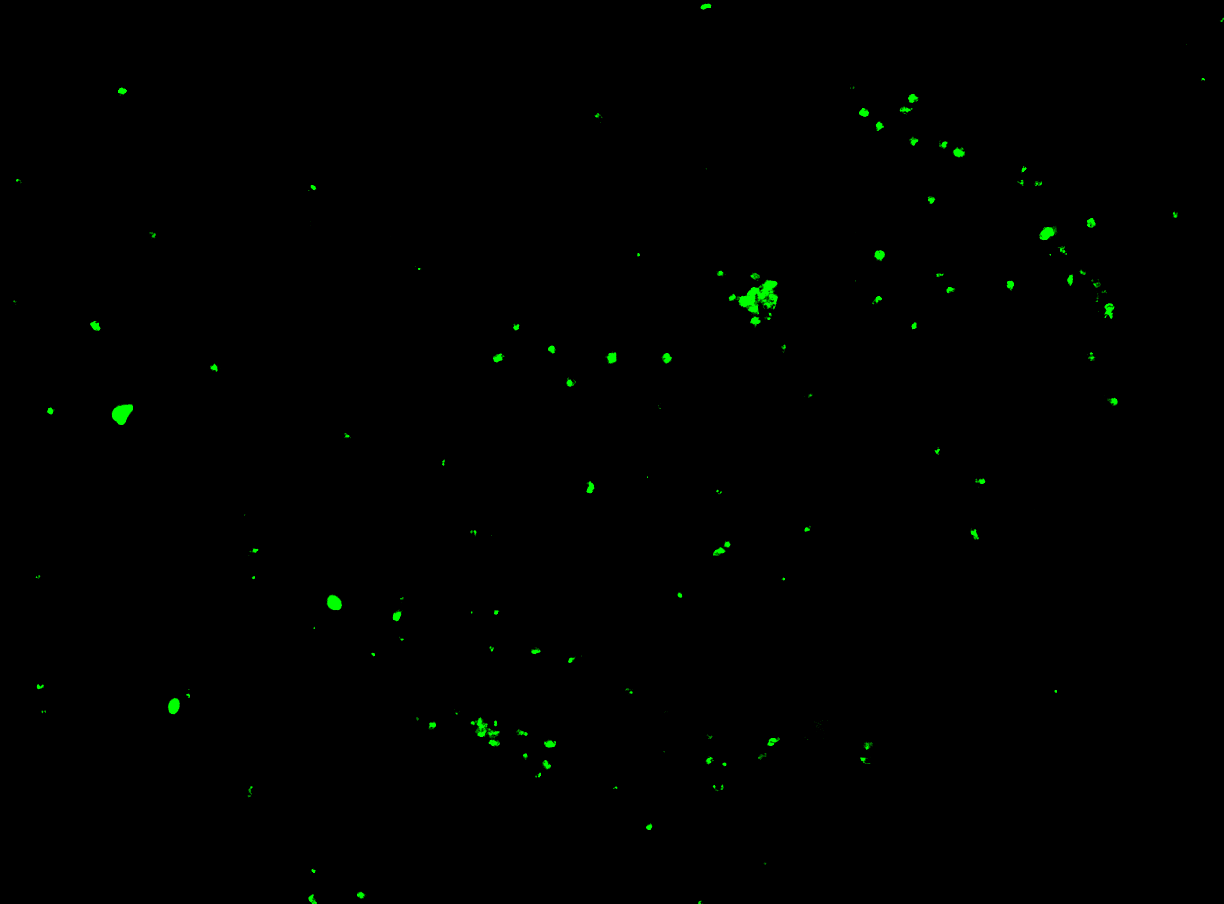

Supplement: Supplementary file 12 — Source data Fig. 6 [file 44321_2024_117_MOESM12_ESM.zip › Figure 6/6G/1h tPA_Ly6G.tif]

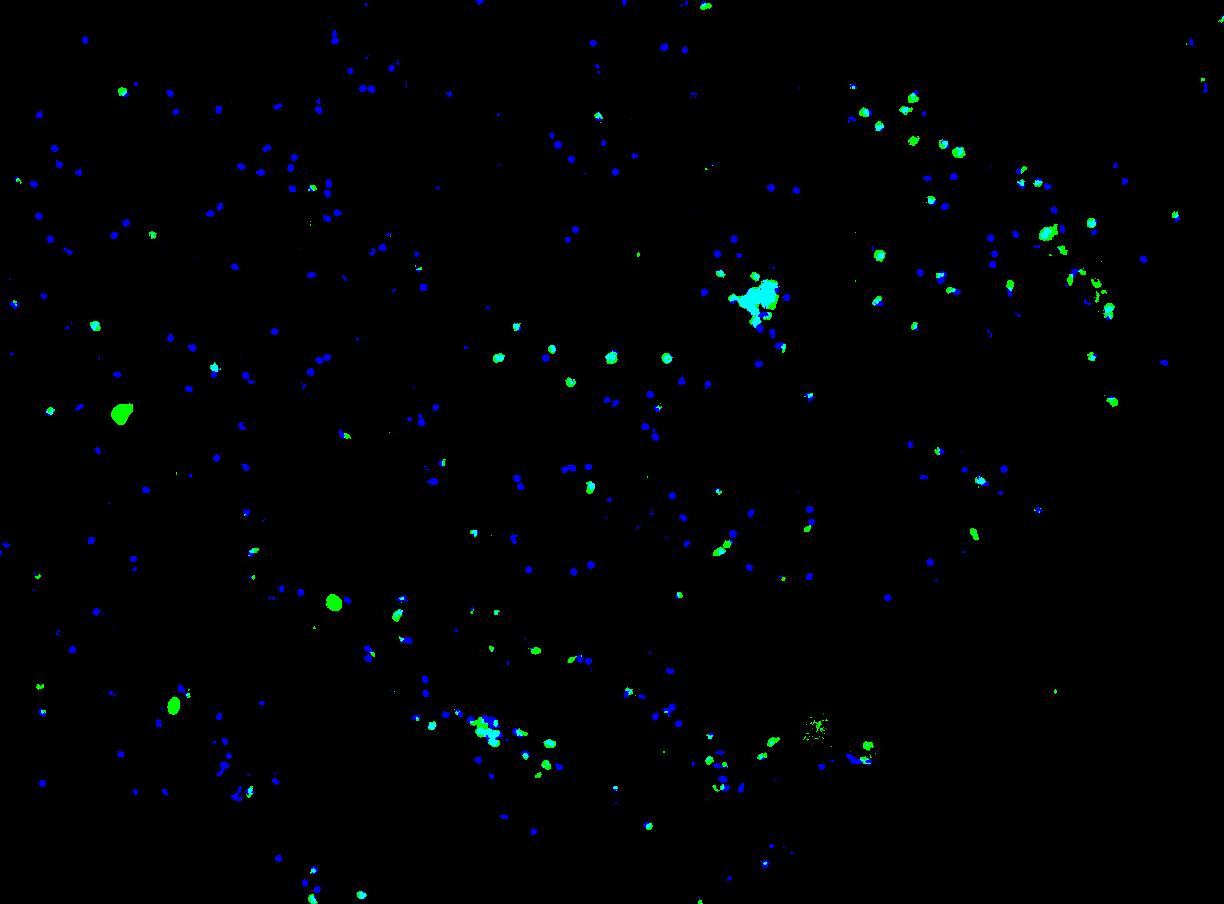

Supplement: Supplementary file 12 — Source data Fig. 6 [file 44321_2024_117_MOESM12_ESM.zip › Figure 6/6G/1h tPA_Merge.tif]

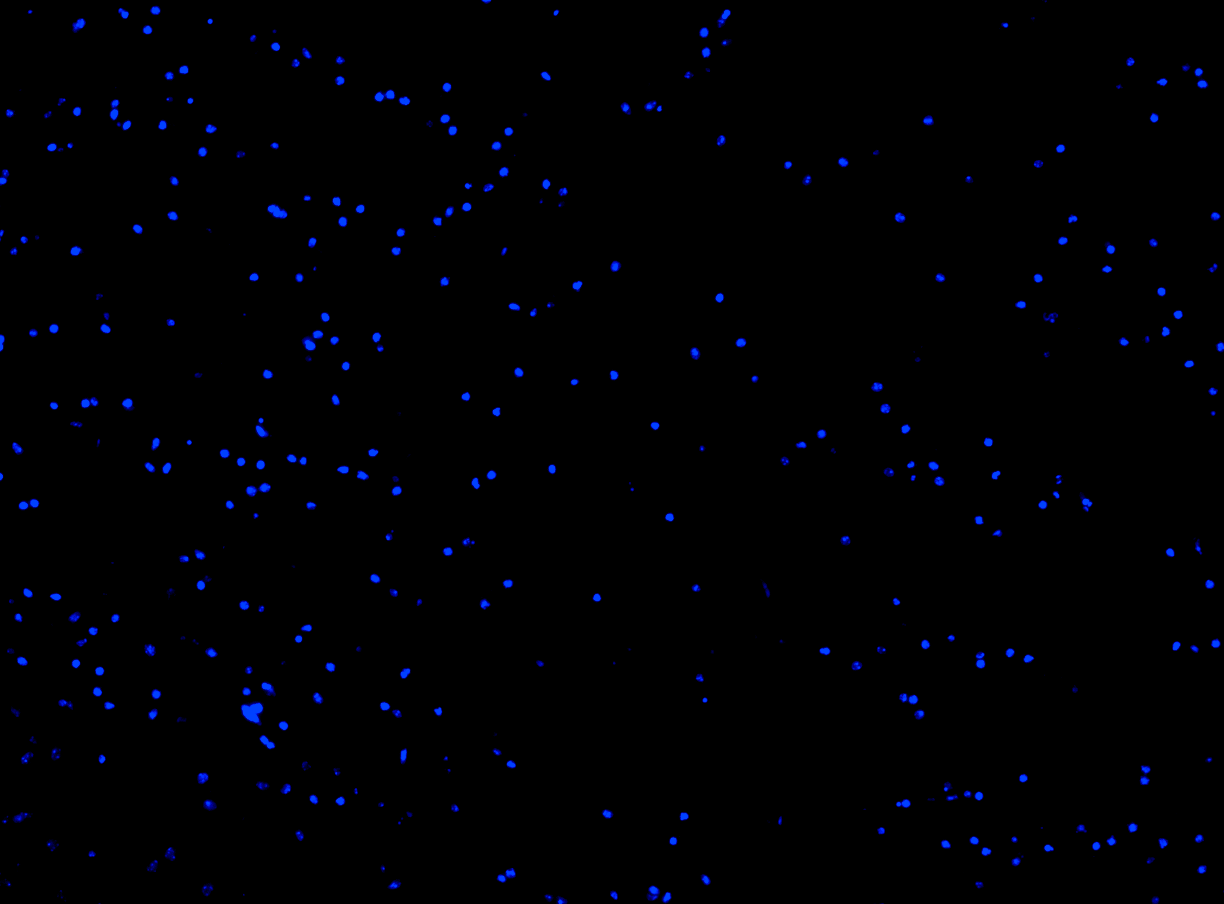

Supplement: Supplementary file 12 — Source data Fig. 6 [file 44321_2024_117_MOESM12_ESM.zip › Figure 6/6G/5h tPA+HRG_DAPI.tif]

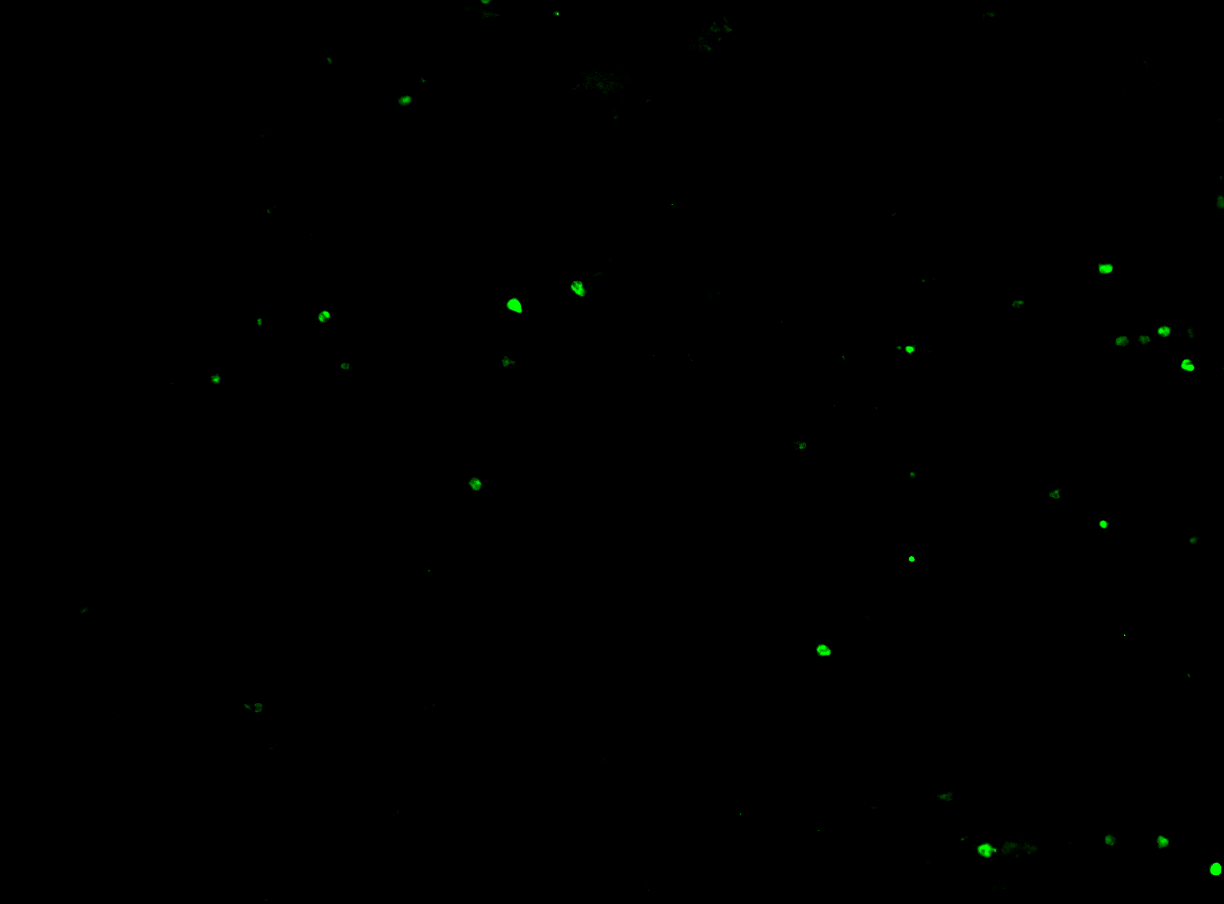

Supplement: Supplementary file 12 — Source data Fig. 6 [file 44321_2024_117_MOESM12_ESM.zip › Figure 6/6G/5h tPA+HRG_Ly6G.tif]

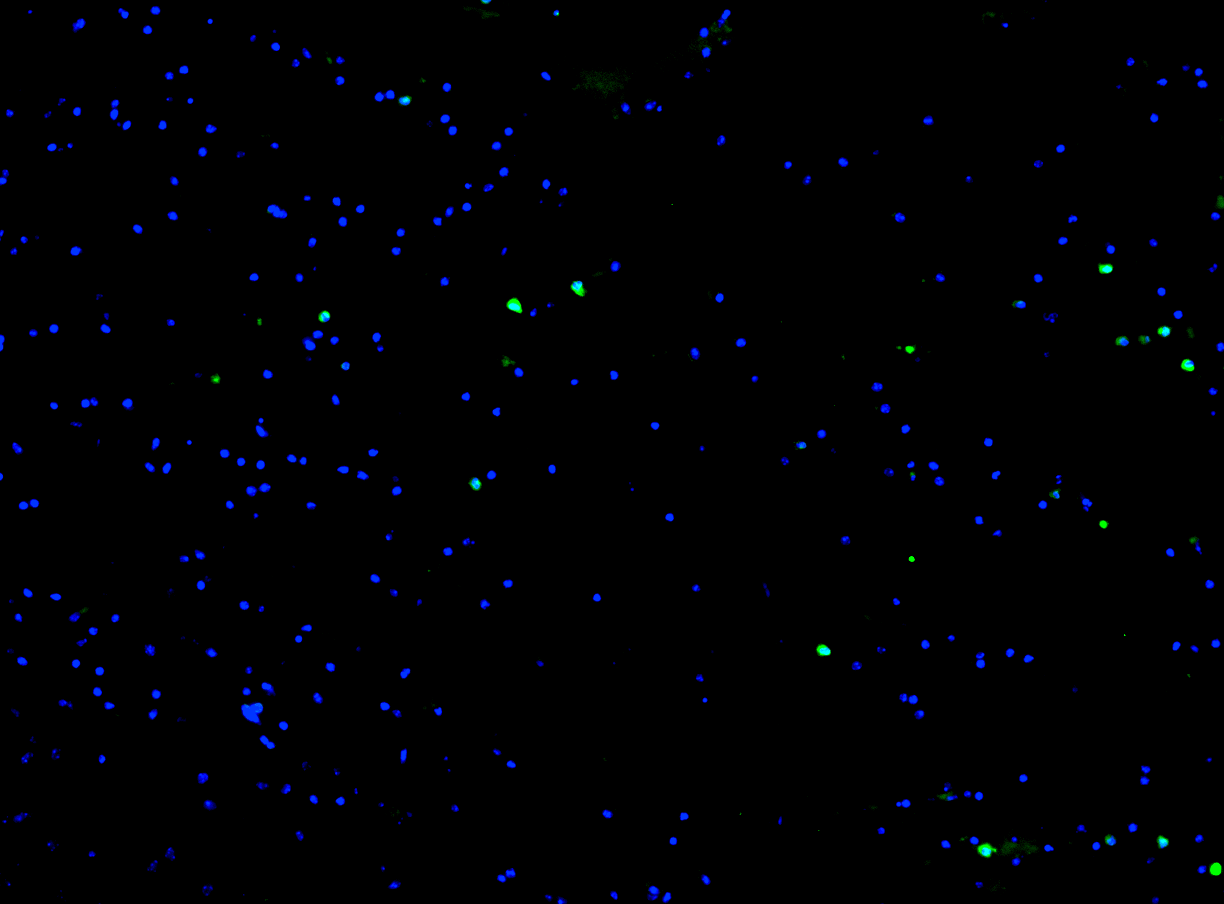

Supplement: Supplementary file 12 — Source data Fig. 6 [file 44321_2024_117_MOESM12_ESM.zip › Figure 6/6G/5h tPA+HRG_Merge.tif]

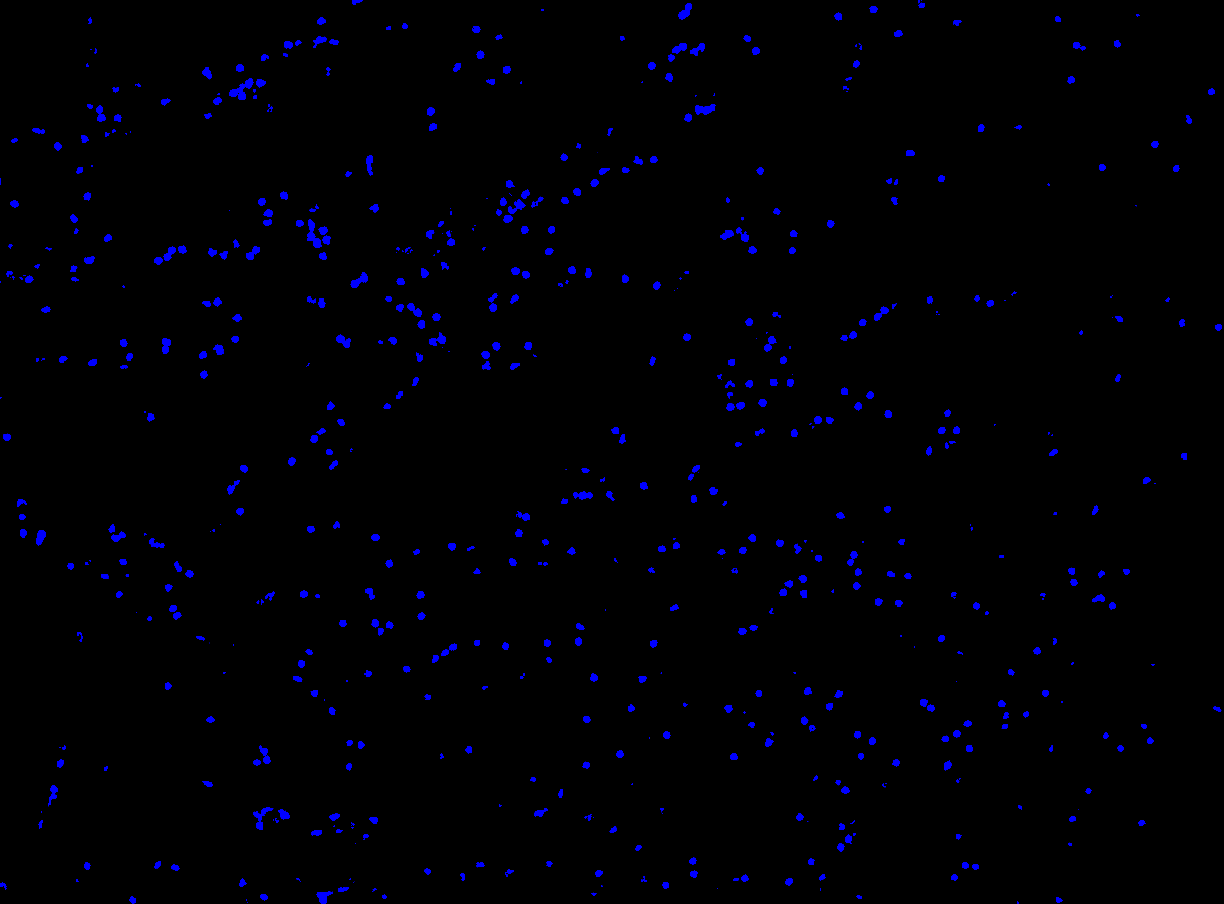

Supplement: Supplementary file 12 — Source data Fig. 6 [file 44321_2024_117_MOESM12_ESM.zip › Figure 6/6G/5h tPA_DAPI.tif]

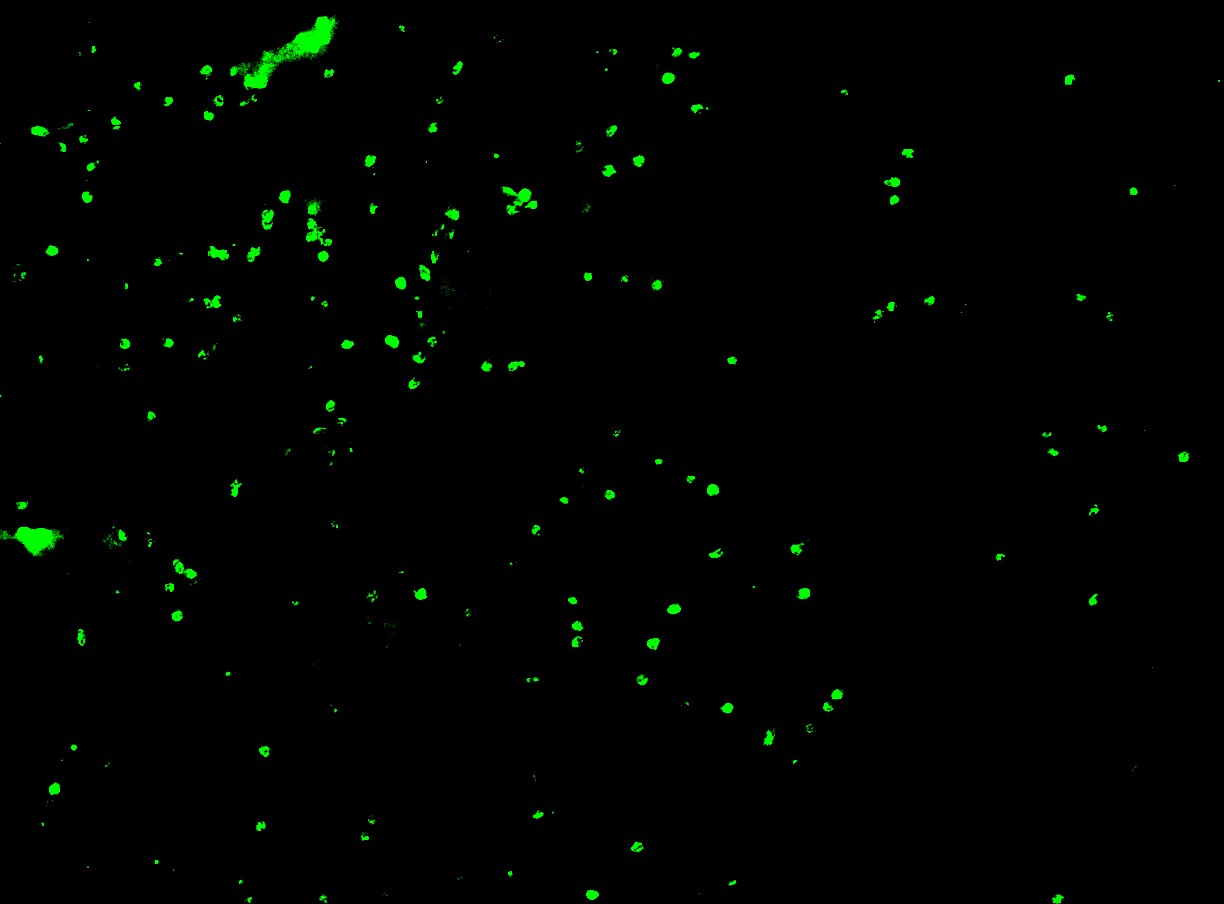

Supplement: Supplementary file 12 — Source data Fig. 6 [file 44321_2024_117_MOESM12_ESM.zip › Figure 6/6G/5h tPA_Ly6G.tif]

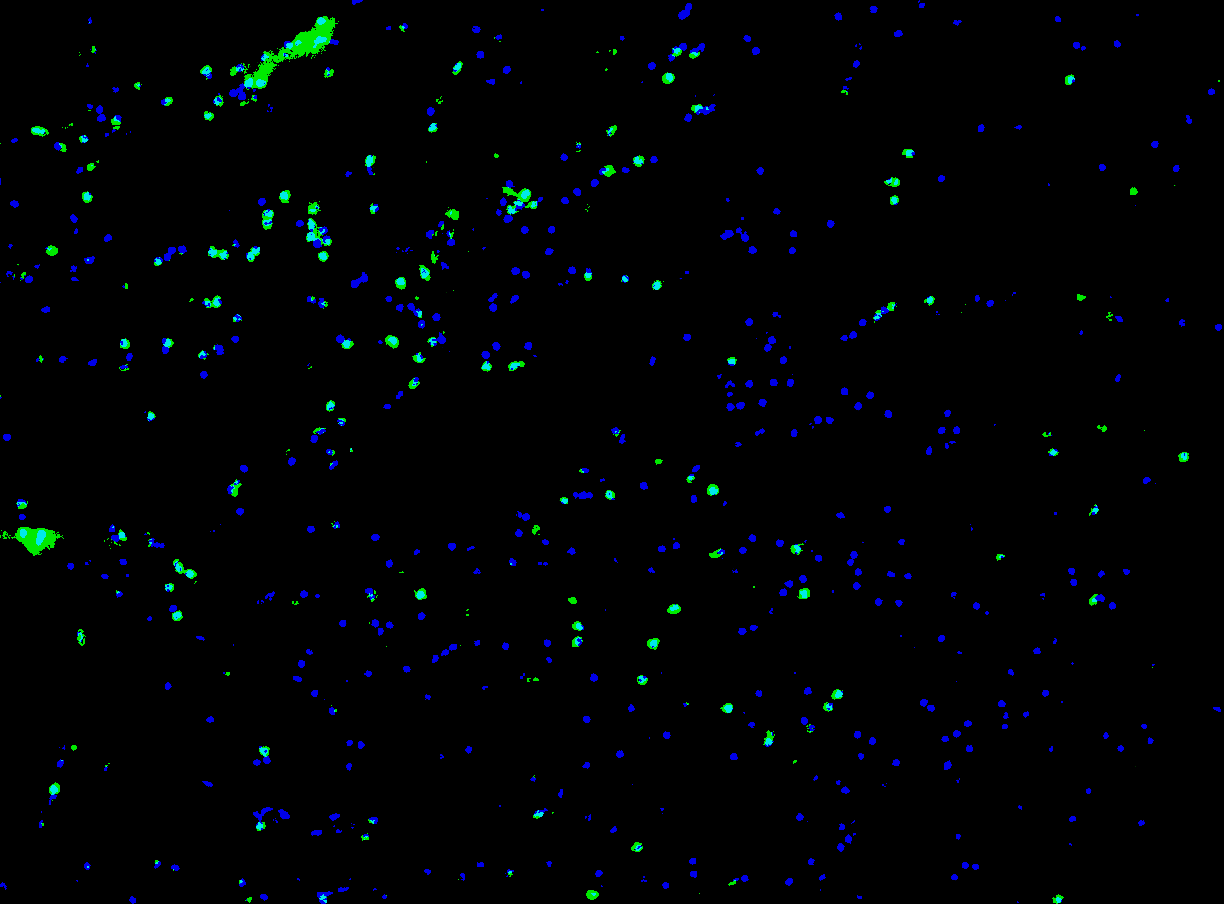

Supplement: Supplementary file 12 — Source data Fig. 6 [file 44321_2024_117_MOESM12_ESM.zip › Figure 6/6G/5h tPA_Merge.tif]

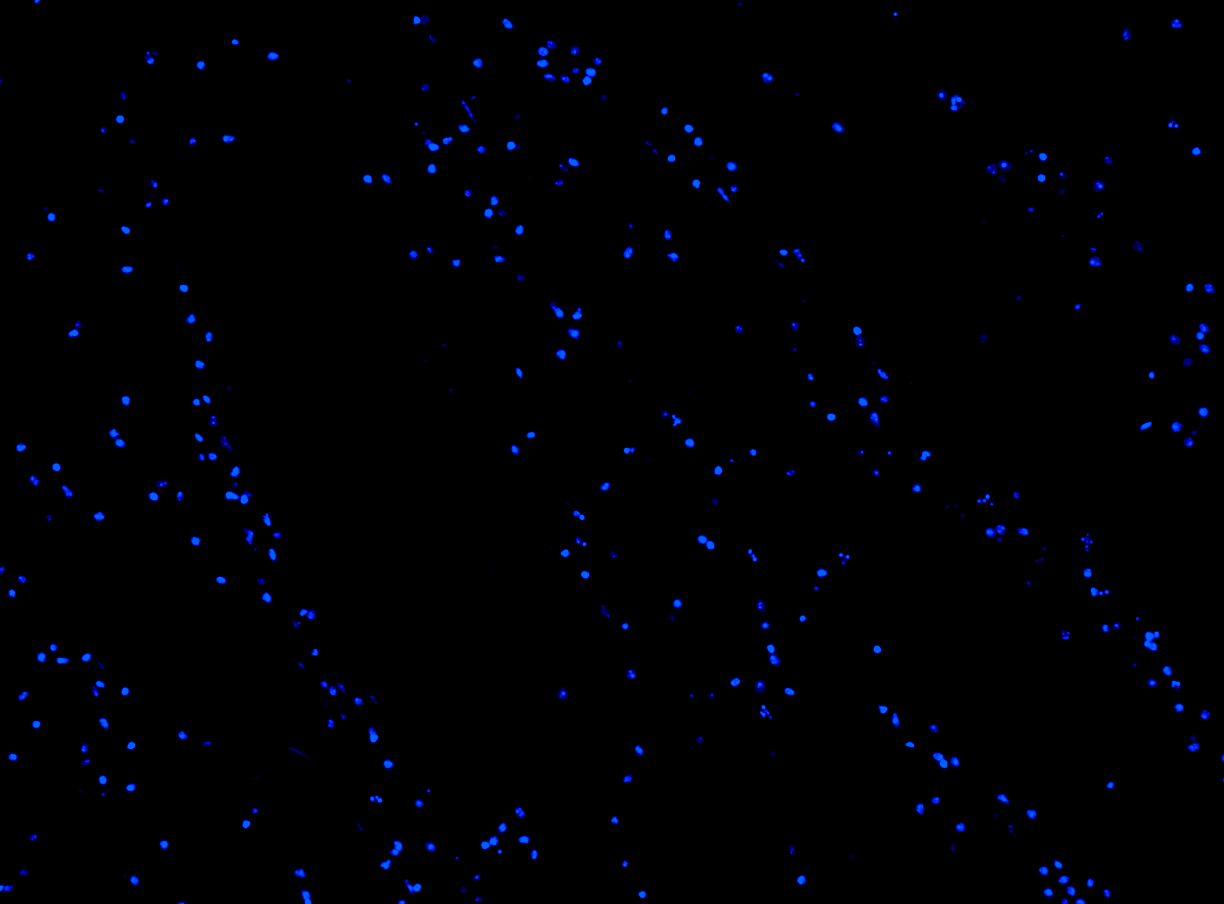

Supplement: Supplementary file 12 — Source data Fig. 6 [file 44321_2024_117_MOESM12_ESM.zip › Figure 6/6G/Vehicle_DAPI.tif]

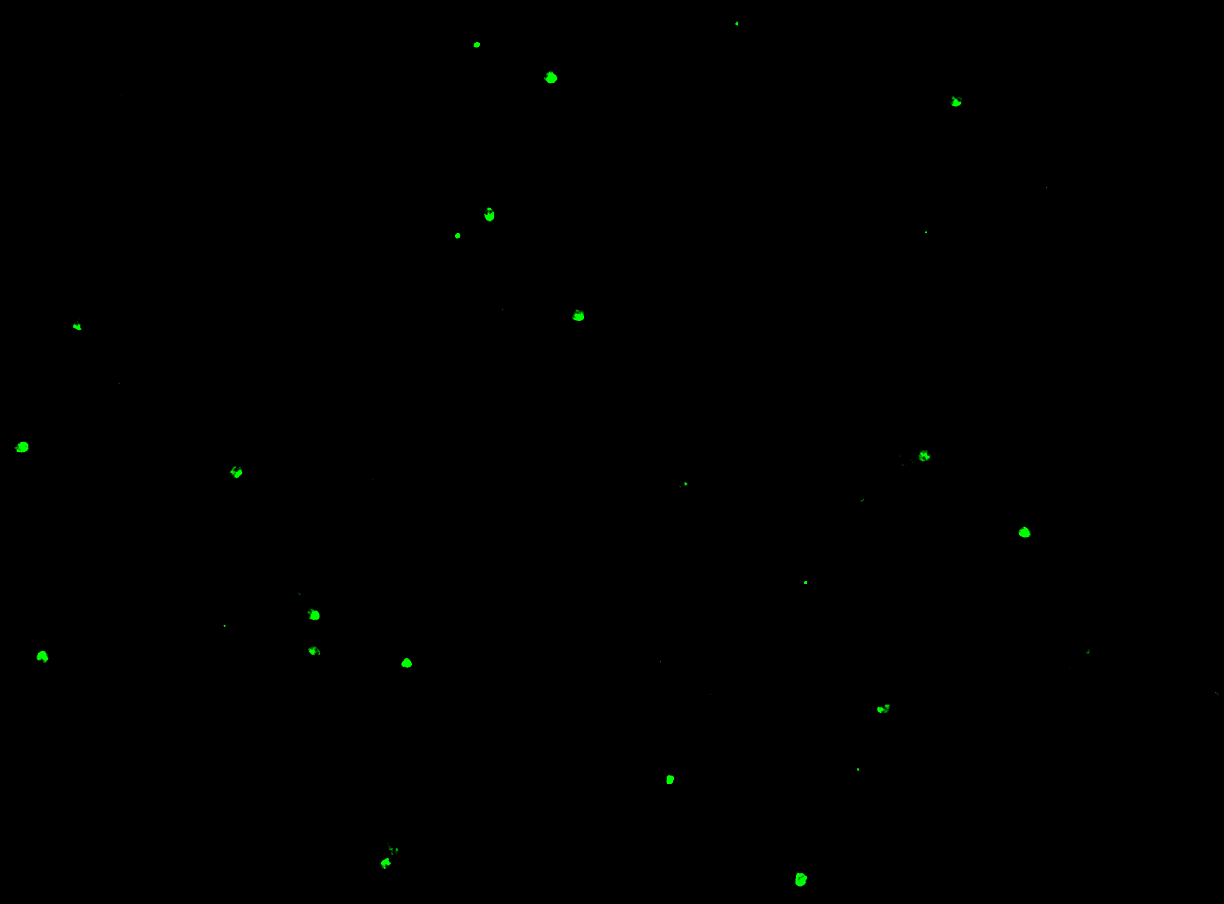

Supplement: Supplementary file 12 — Source data Fig. 6 [file 44321_2024_117_MOESM12_ESM.zip › Figure 6/6G/Vehicle_Ly6G.tif]

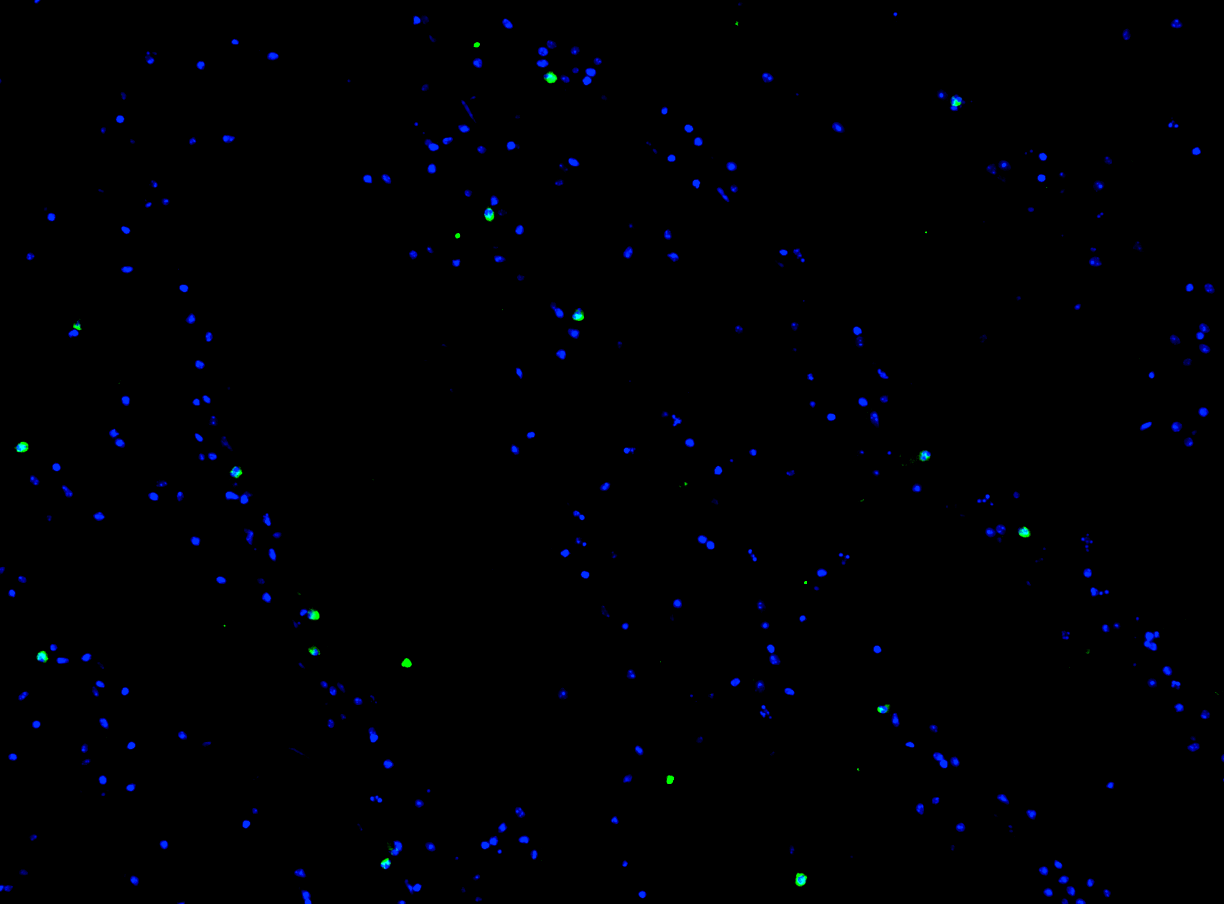

Supplement: Supplementary file 12 — Source data Fig. 6 [file 44321_2024_117_MOESM12_ESM.zip › Figure 6/6G/Vehicle_Merge.tif]

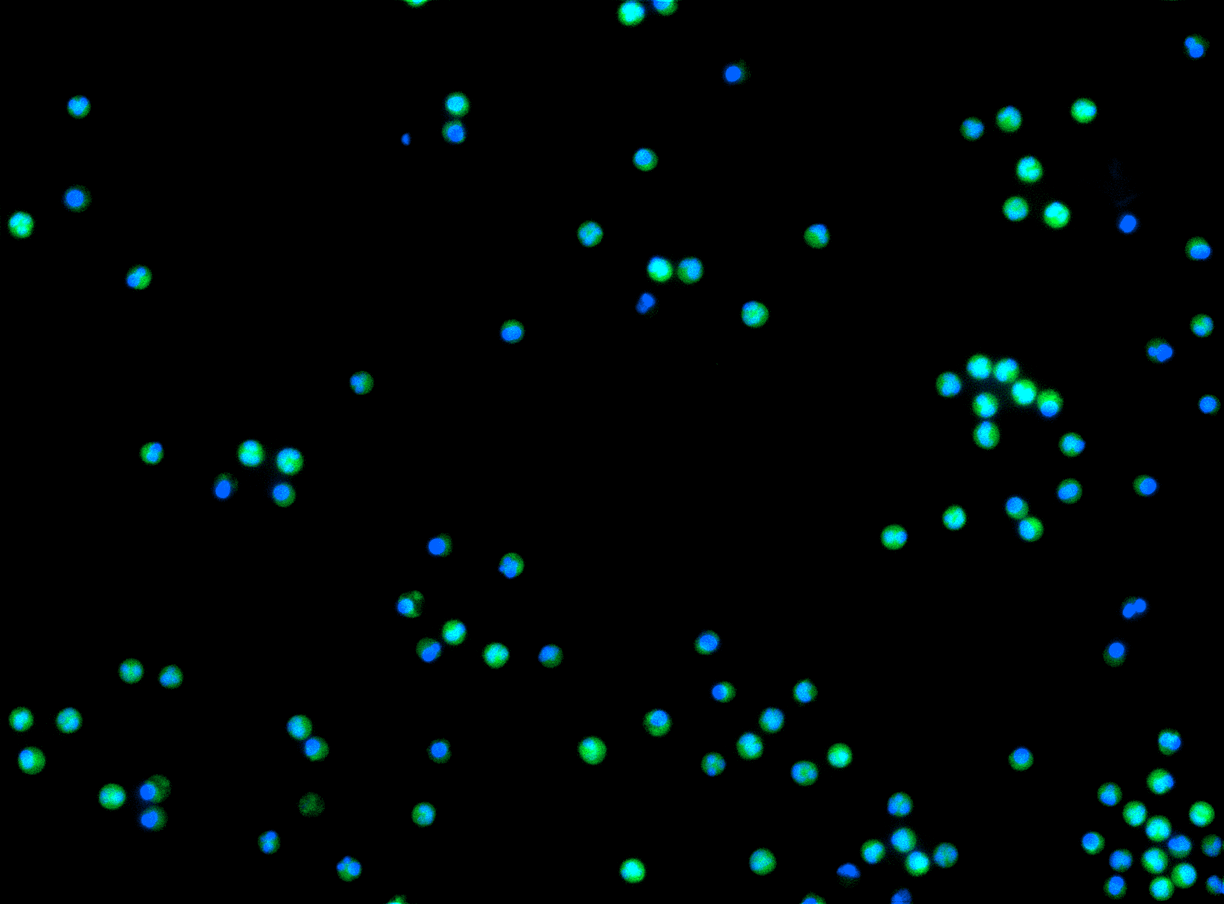

Supplement: Supplementary file 13 — Source Data for EV and Appendix figures [file 44321_2024_117_MOESM13_ESM.zip › Source Data for Expanded View and Appendix 5-23 f/Appendix Figures Source Data/Figure S3/Figure S3A/Control_12h.tif]

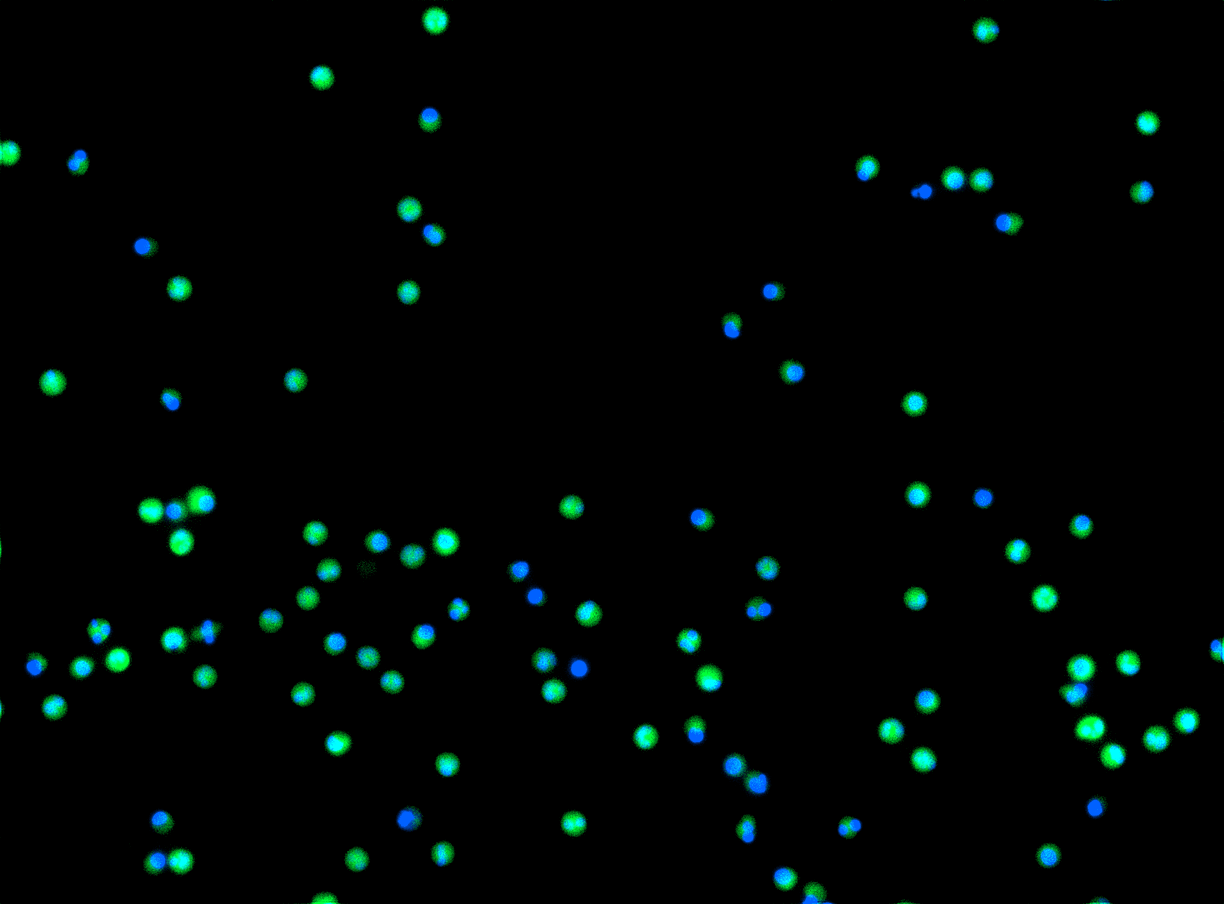

Supplement: Supplementary file 13 — Source Data for EV and Appendix figures [file 44321_2024_117_MOESM13_ESM.zip › Source Data for Expanded View and Appendix 5-23 f/Appendix Figures Source Data/Figure S3/Figure S3A/Control_18h.tif]

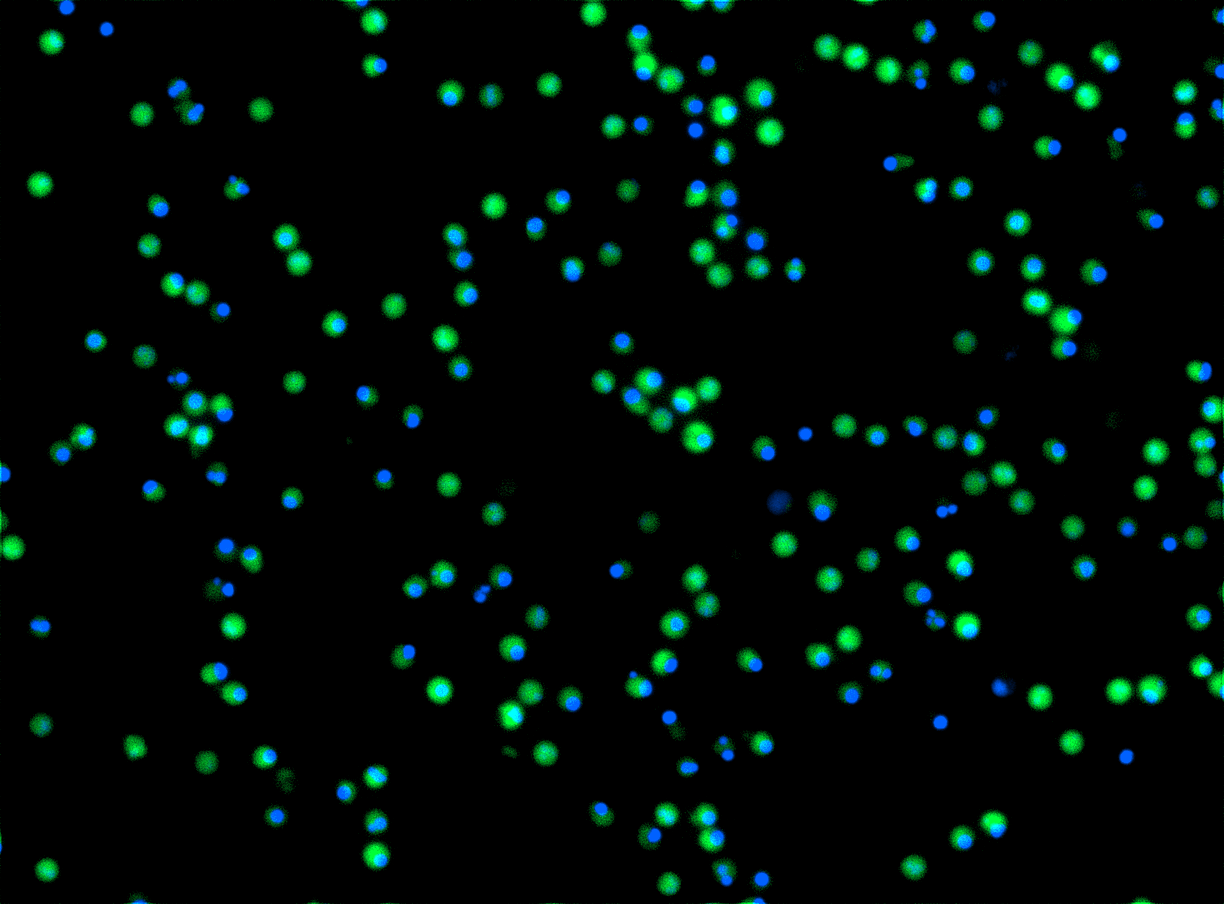

Supplement: Supplementary file 13 — Source Data for EV and Appendix figures [file 44321_2024_117_MOESM13_ESM.zip › Source Data for Expanded View and Appendix 5-23 f/Appendix Figures Source Data/Figure S3/Figure S3A/Control_24h.tif]

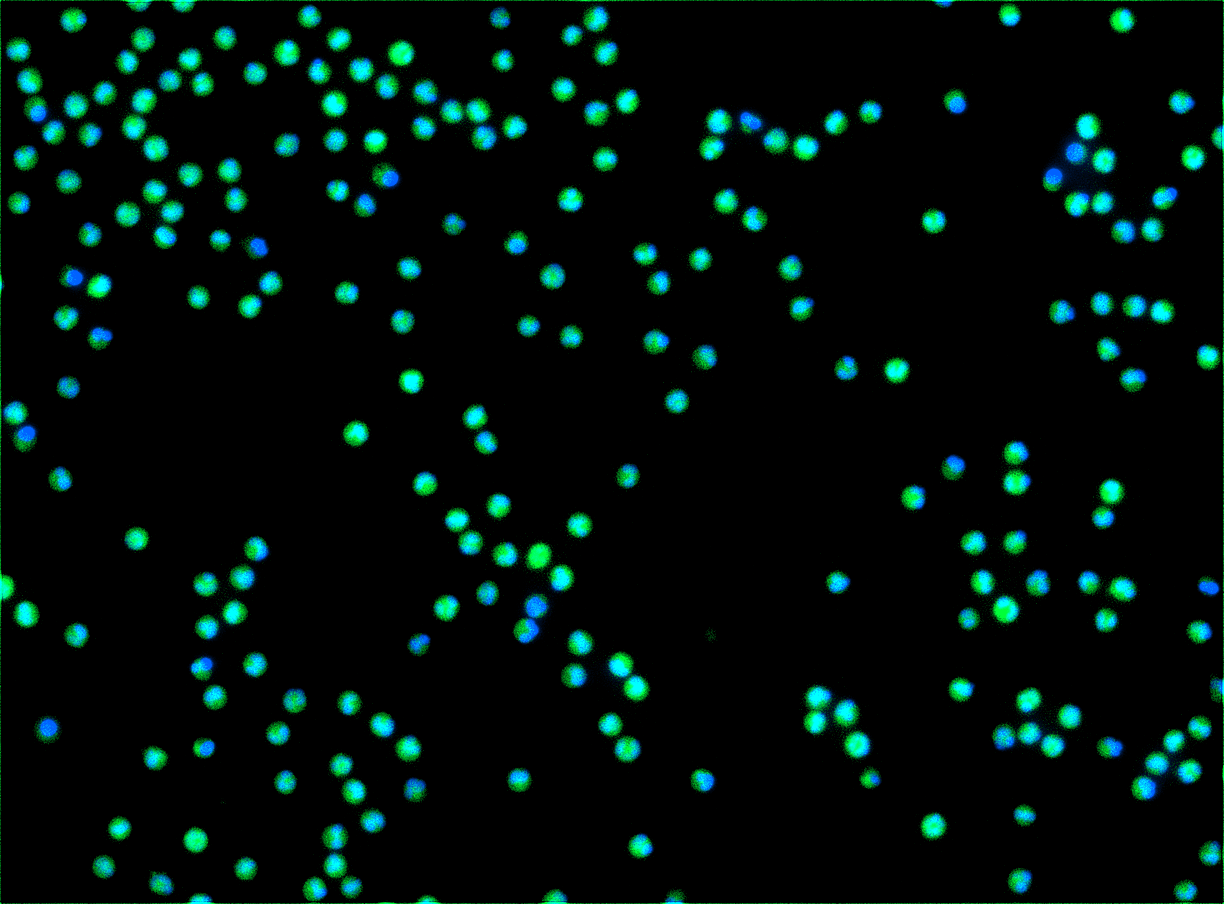

Supplement: Supplementary file 13 — Source Data for EV and Appendix figures [file 44321_2024_117_MOESM13_ESM.zip › Source Data for Expanded View and Appendix 5-23 f/Appendix Figures Source Data/Figure S3/Figure S3A/Control_4h.tif]

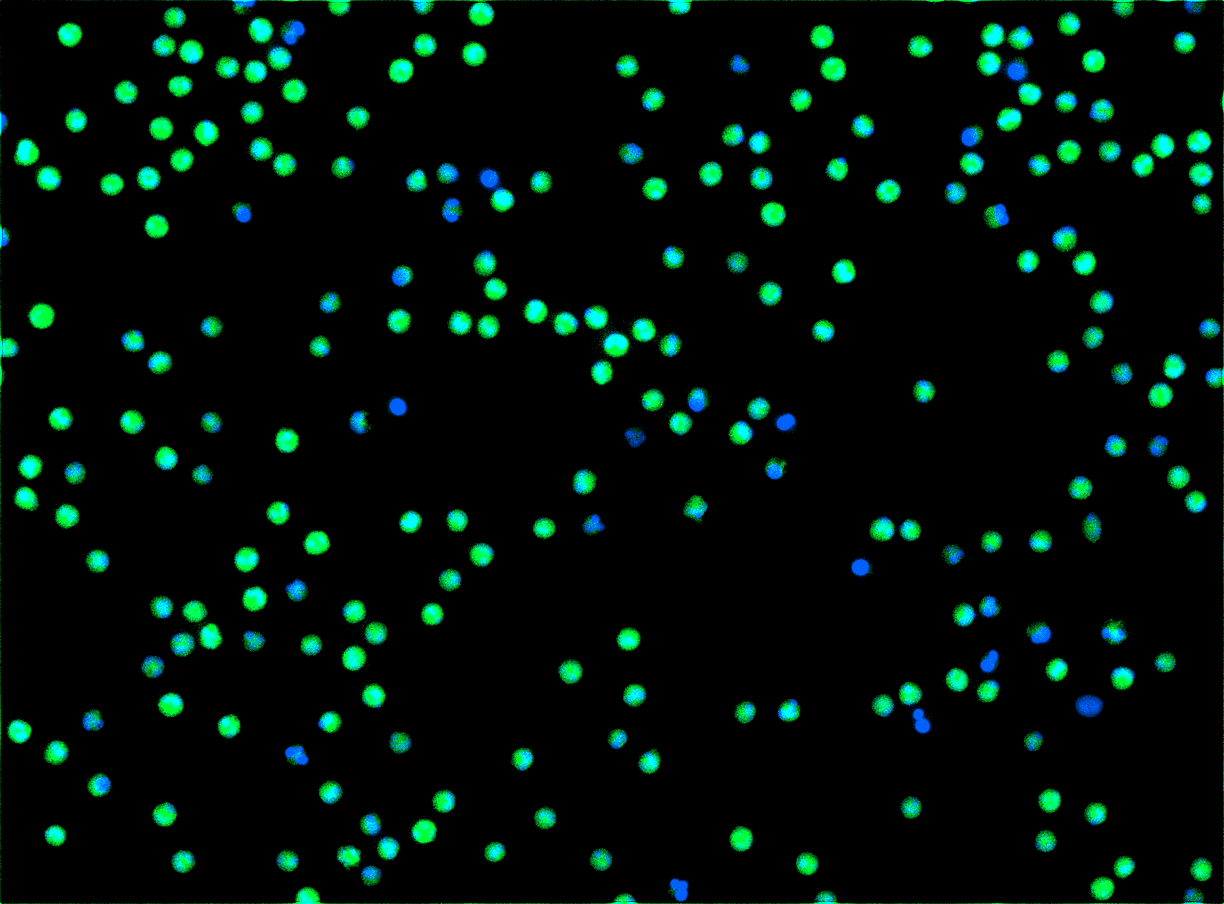

Supplement: Supplementary file 13 — Source Data for EV and Appendix figures [file 44321_2024_117_MOESM13_ESM.zip › Source Data for Expanded View and Appendix 5-23 f/Appendix Figures Source Data/Figure S3/Figure S3A/Control_8h.tif]

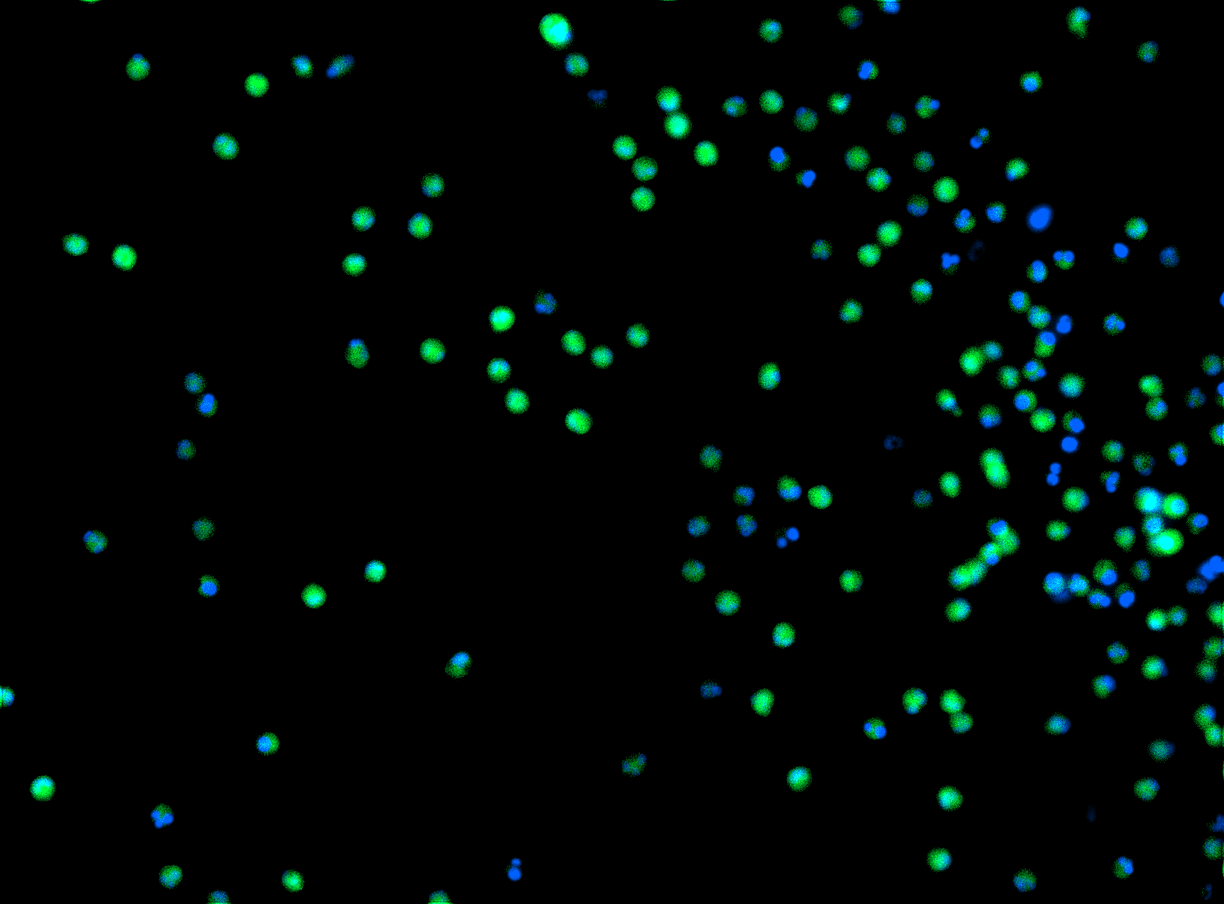

Supplement: Supplementary file 13 — Source Data for EV and Appendix figures [file 44321_2024_117_MOESM13_ESM.zip › Source Data for Expanded View and Appendix 5-23 f/Appendix Figures Source Data/Figure S3/Figure S3A/LPS_12h.tif]

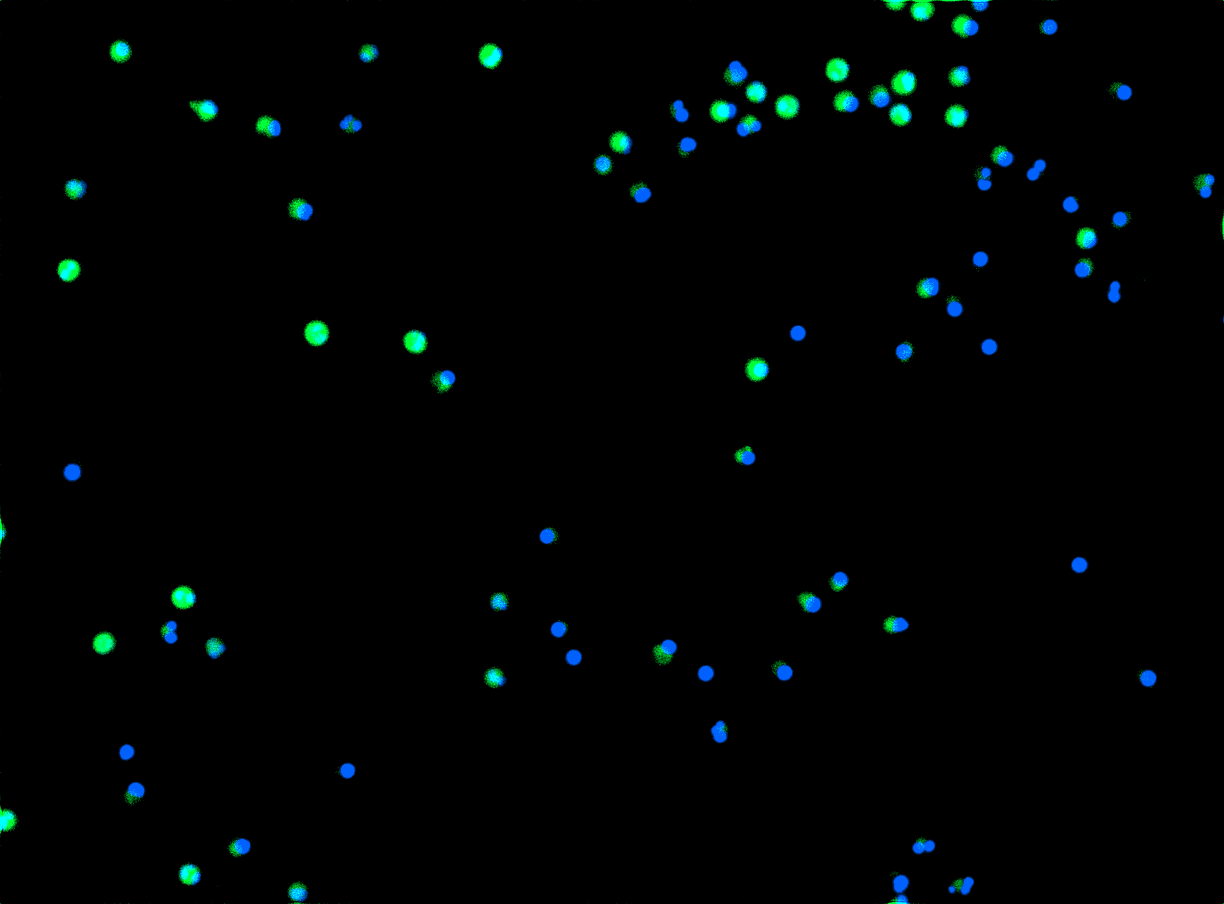

Supplement: Supplementary file 13 — Source Data for EV and Appendix figures [file 44321_2024_117_MOESM13_ESM.zip › Source Data for Expanded View and Appendix 5-23 f/Appendix Figures Source Data/Figure S3/Figure S3A/LPS_18h.tif]

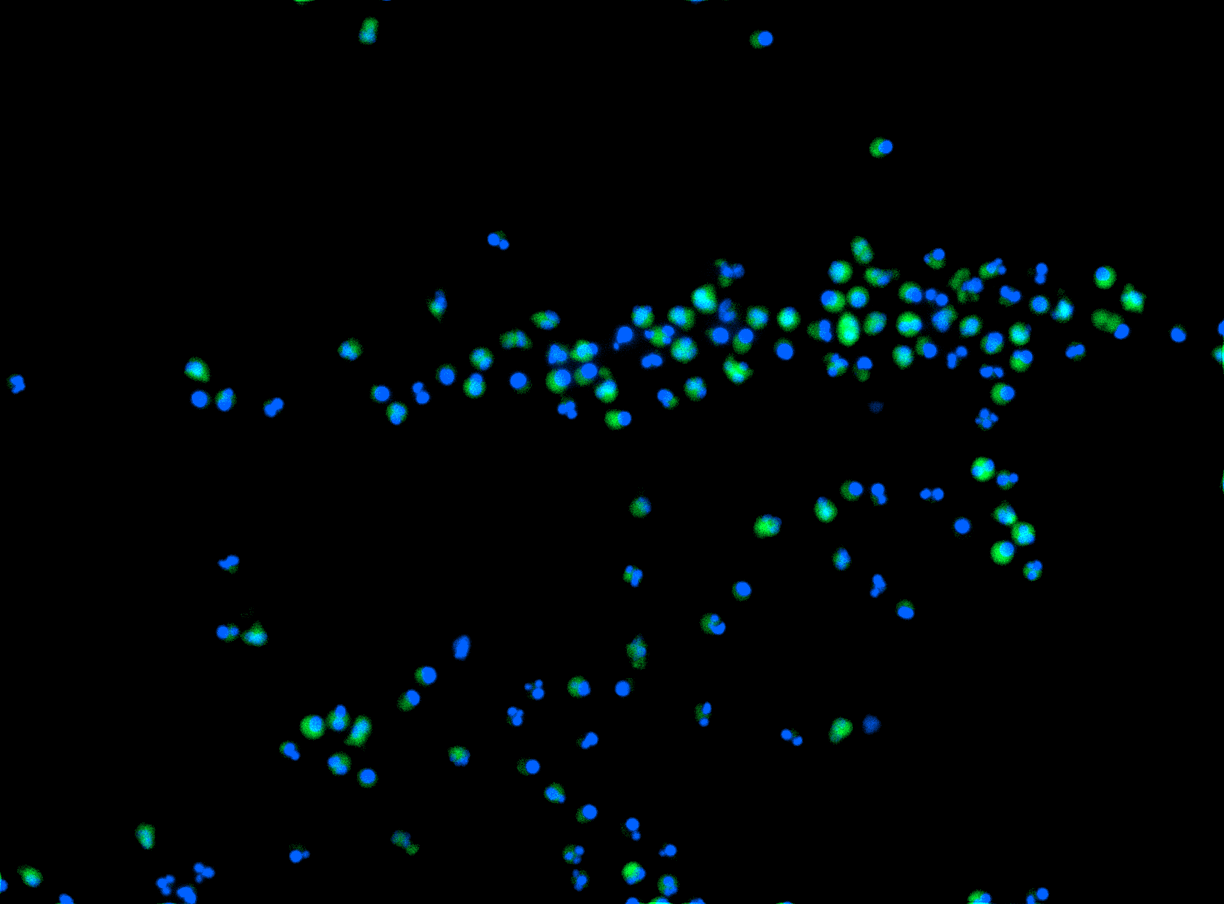

Supplement: Supplementary file 13 — Source Data for EV and Appendix figures [file 44321_2024_117_MOESM13_ESM.zip › Source Data for Expanded View and Appendix 5-23 f/Appendix Figures Source Data/Figure S3/Figure S3A/LPS_24h.tif]

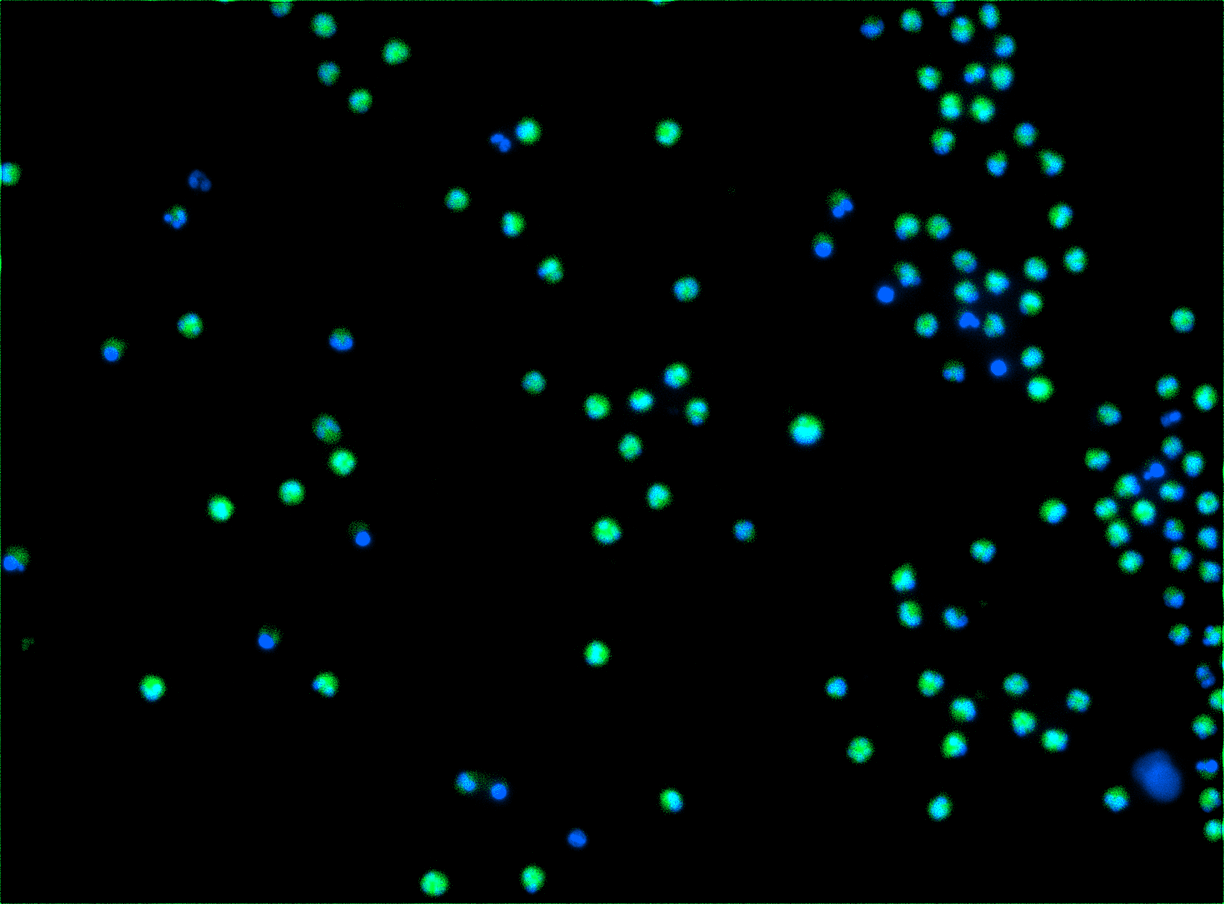

Supplement: Supplementary file 13 — Source Data for EV and Appendix figures [file 44321_2024_117_MOESM13_ESM.zip › Source Data for Expanded View and Appendix 5-23 f/Appendix Figures Source Data/Figure S3/Figure S3A/LPS_4h.tif]

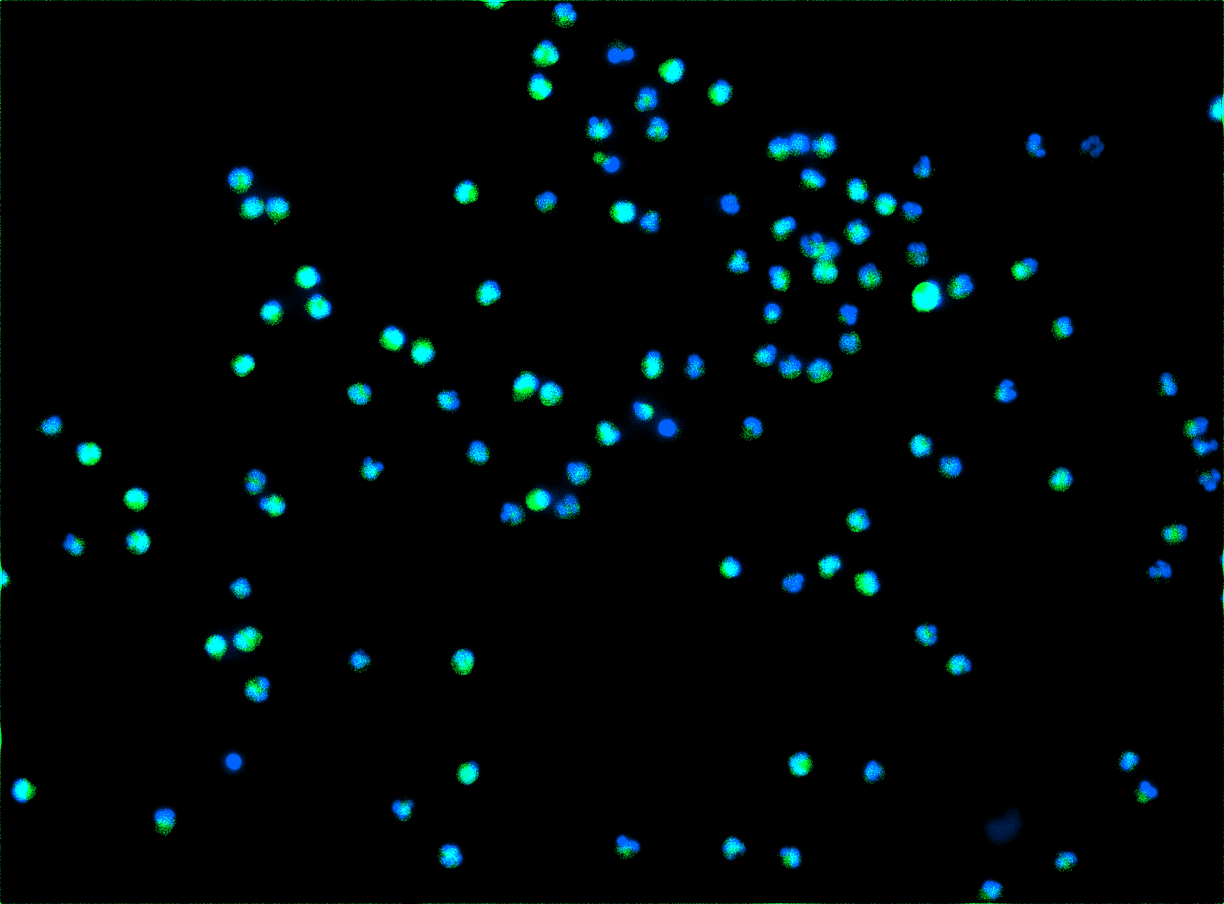

Supplement: Supplementary file 13 — Source Data for EV and Appendix figures [file 44321_2024_117_MOESM13_ESM.zip › Source Data for Expanded View and Appendix 5-23 f/Appendix Figures Source Data/Figure S3/Figure S3A/LPS_8h.tif]

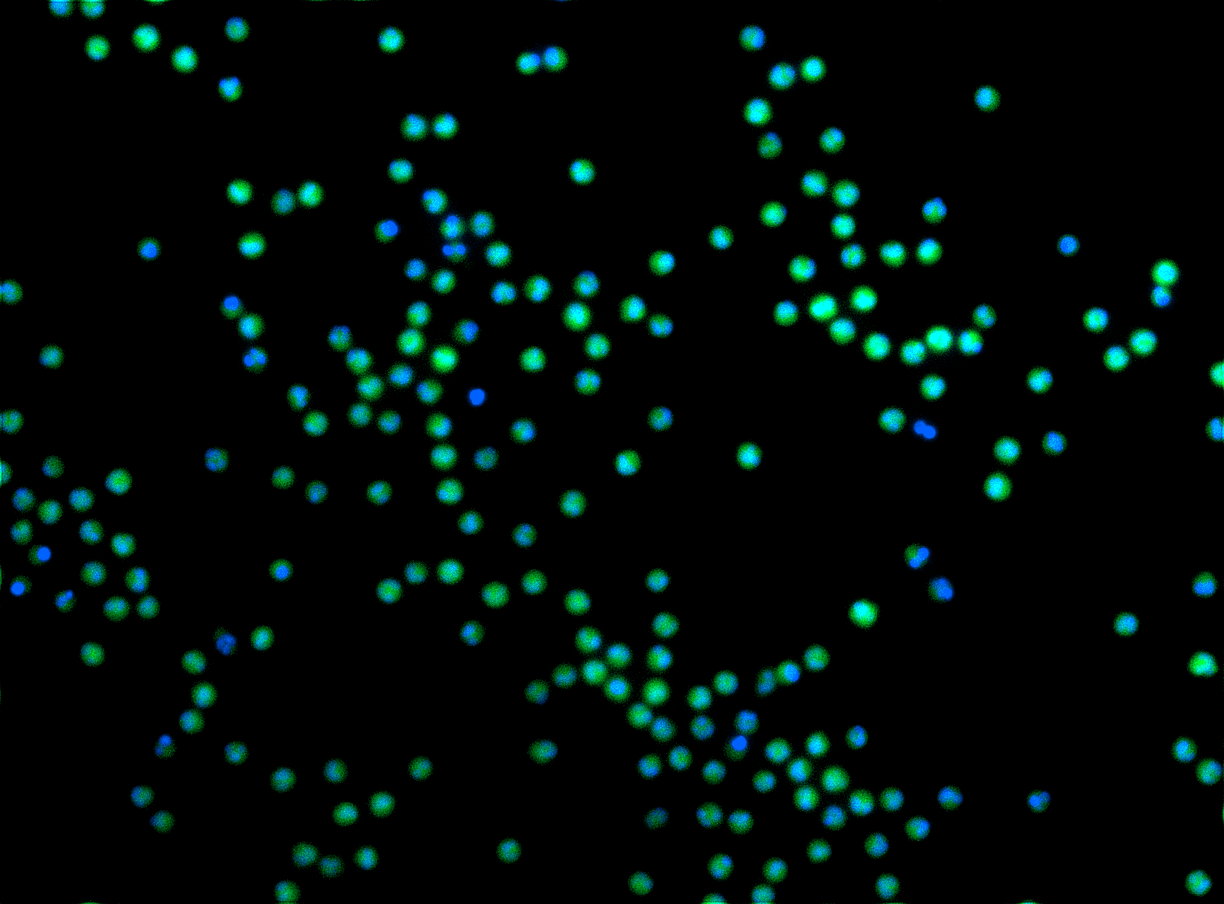

Supplement: Supplementary file 13 — Source Data for EV and Appendix figures [file 44321_2024_117_MOESM13_ESM.zip › Source Data for Expanded View and Appendix 5-23 f/Appendix Figures Source Data/Figure S3/Figure S3A/tPA+HRG_12h.tif]

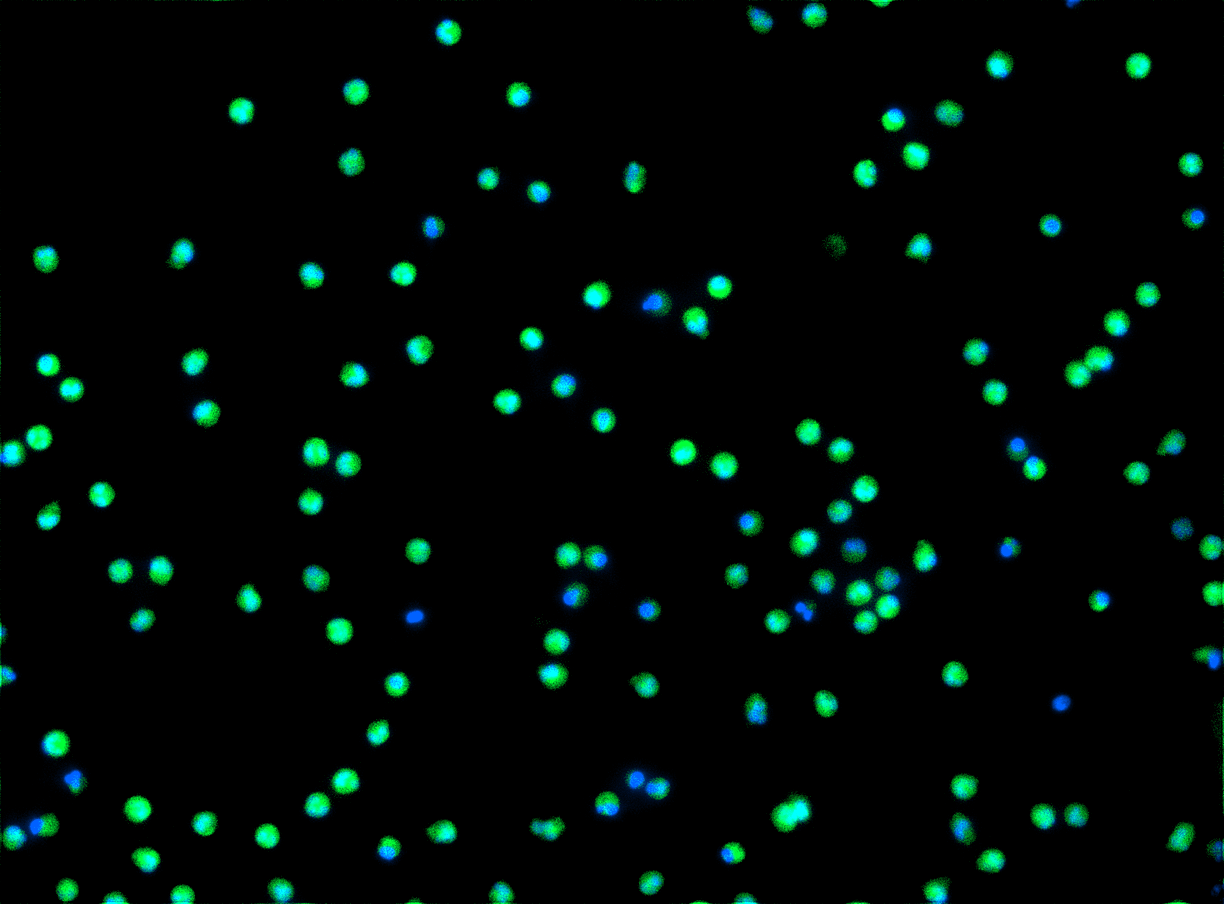

Supplement: Supplementary file 13 — Source Data for EV and Appendix figures [file 44321_2024_117_MOESM13_ESM.zip › Source Data for Expanded View and Appendix 5-23 f/Appendix Figures Source Data/Figure S3/Figure S3A/tPA+HRG_18h.tif]

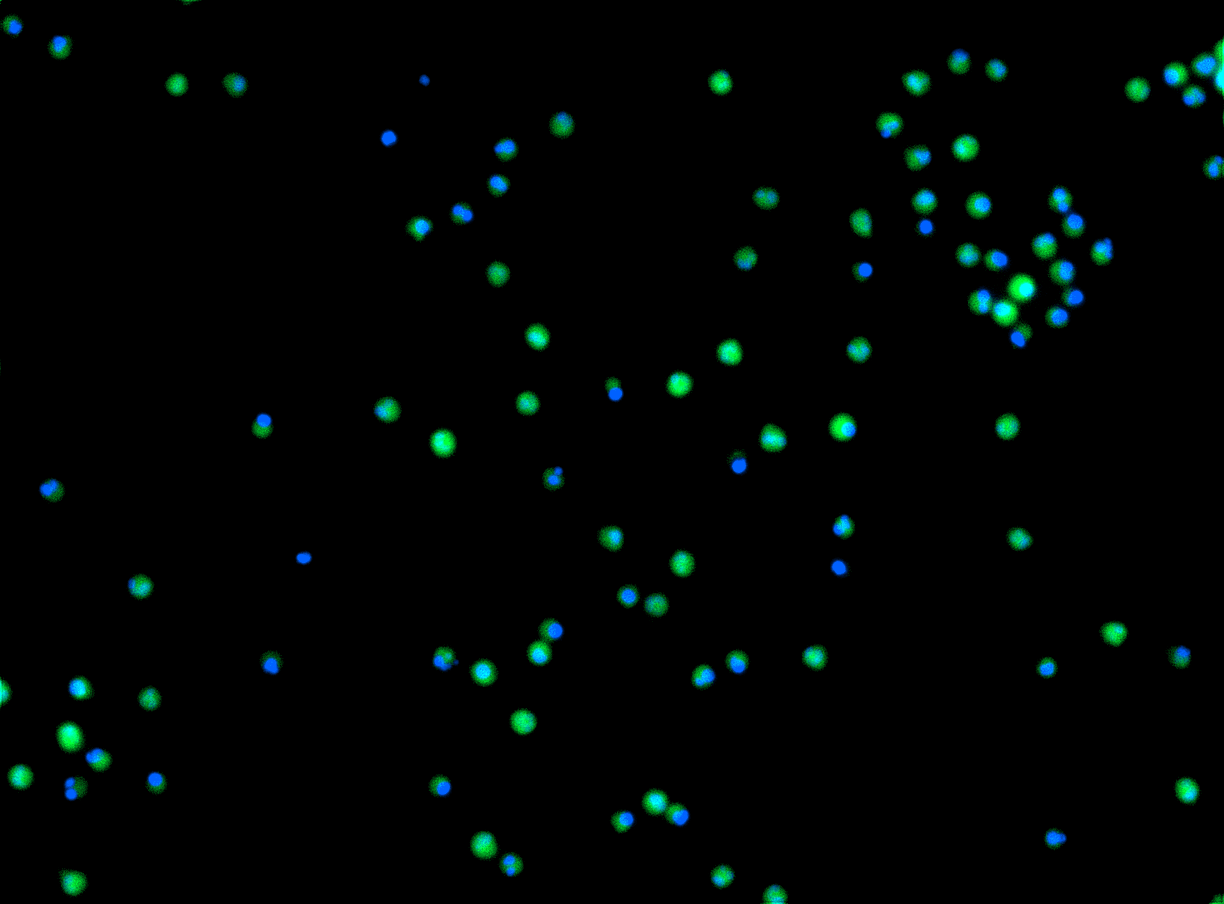

Supplement: Supplementary file 13 — Source Data for EV and Appendix figures [file 44321_2024_117_MOESM13_ESM.zip › Source Data for Expanded View and Appendix 5-23 f/Appendix Figures Source Data/Figure S3/Figure S3A/tPA+HRG_24h.tif]

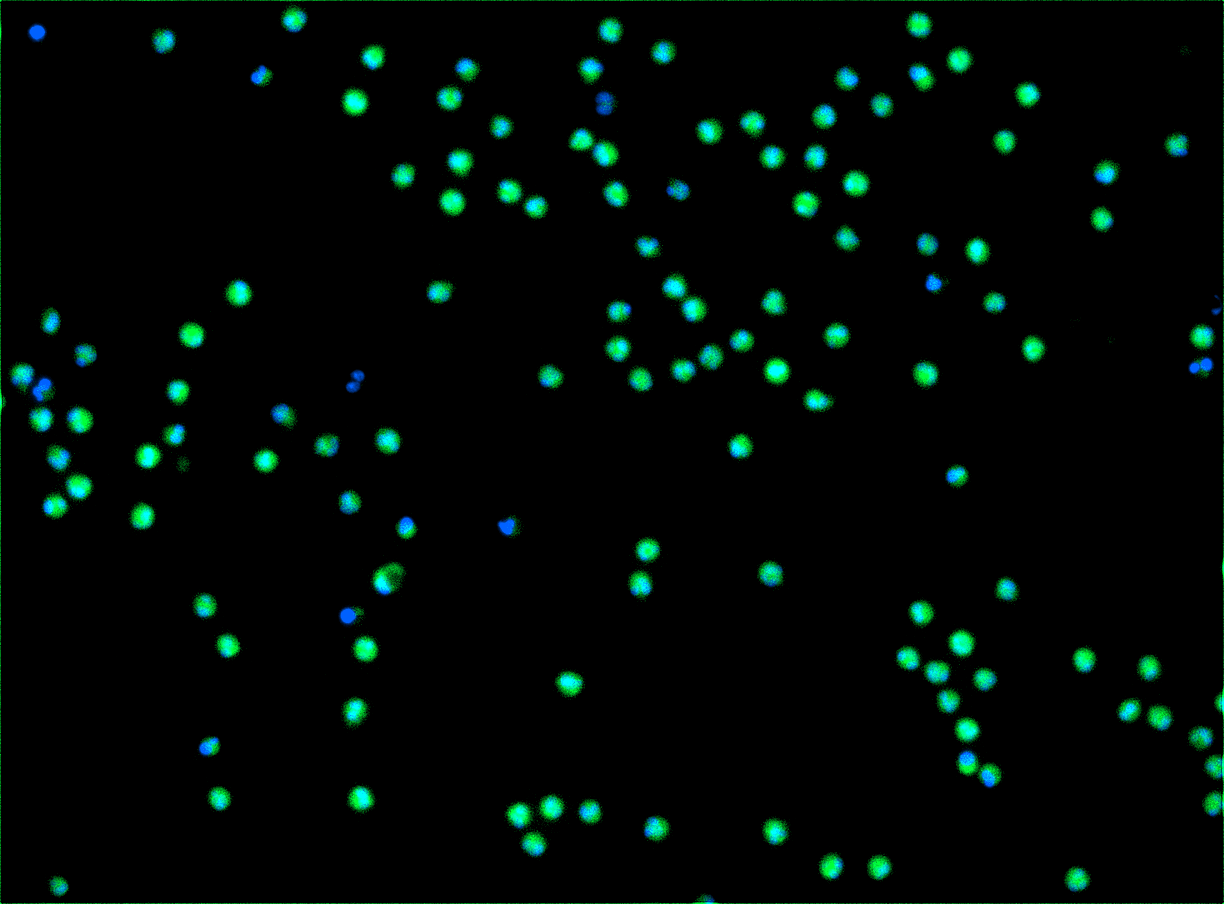

Supplement: Supplementary file 13 — Source Data for EV and Appendix figures [file 44321_2024_117_MOESM13_ESM.zip › Source Data for Expanded View and Appendix 5-23 f/Appendix Figures Source Data/Figure S3/Figure S3A/tPA+HRG_4h.tif]

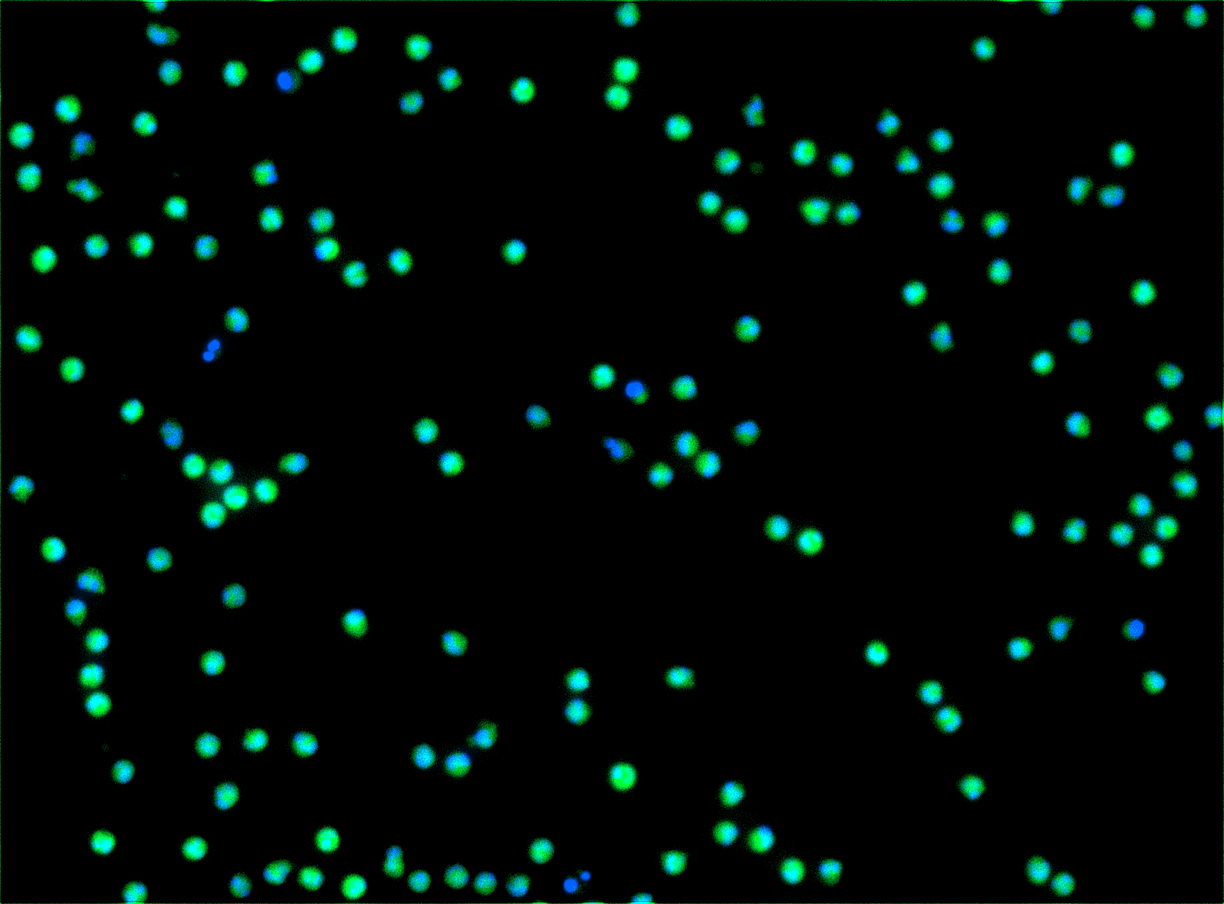

Supplement: Supplementary file 13 — Source Data for EV and Appendix figures [file 44321_2024_117_MOESM13_ESM.zip › Source Data for Expanded View and Appendix 5-23 f/Appendix Figures Source Data/Figure S3/Figure S3A/tPA+HRG_8h.tif]

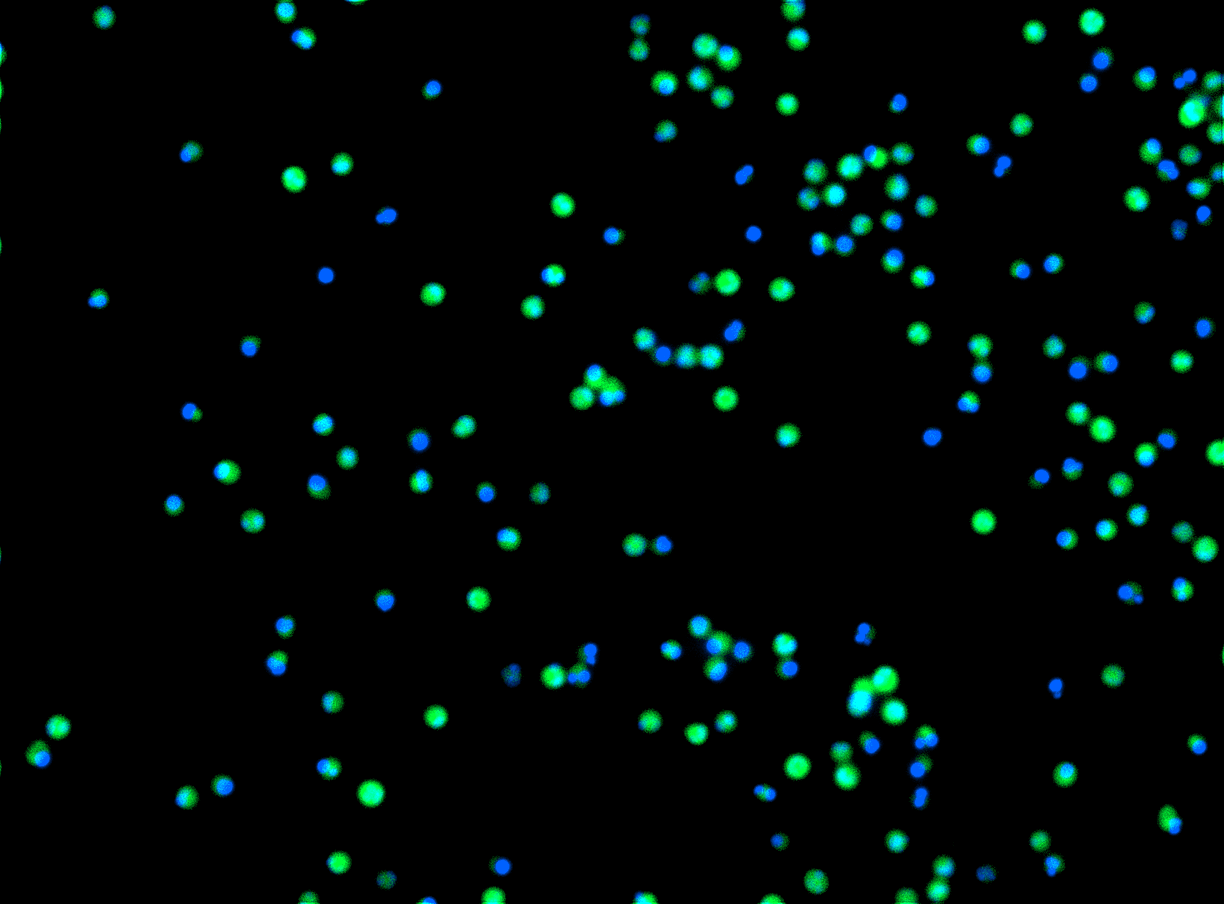

Supplement: Supplementary file 13 — Source Data for EV and Appendix figures [file 44321_2024_117_MOESM13_ESM.zip › Source Data for Expanded View and Appendix 5-23 f/Appendix Figures Source Data/Figure S3/Figure S3A/tPA_12h.tif]

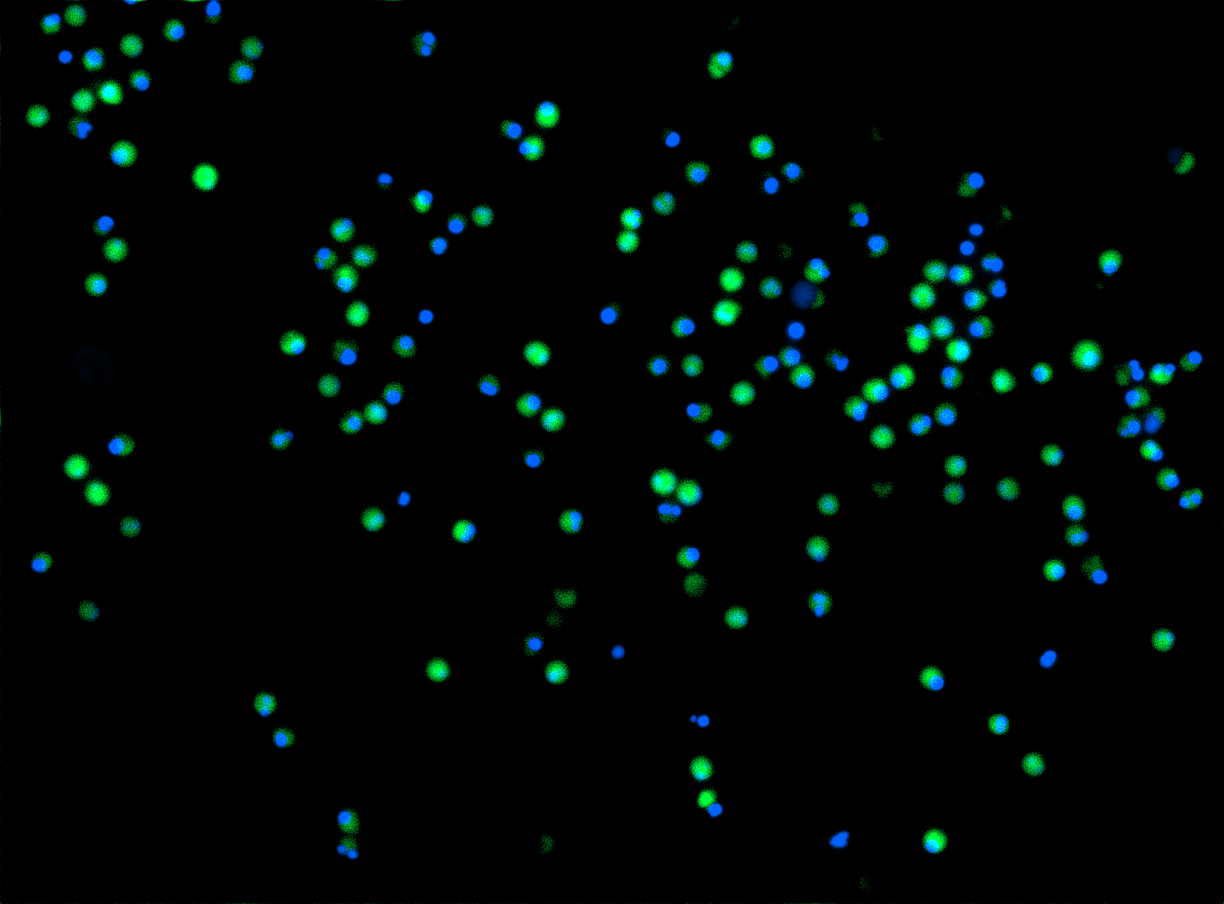

Supplement: Supplementary file 13 — Source Data for EV and Appendix figures [file 44321_2024_117_MOESM13_ESM.zip › Source Data for Expanded View and Appendix 5-23 f/Appendix Figures Source Data/Figure S3/Figure S3A/tPA_18h.tif]

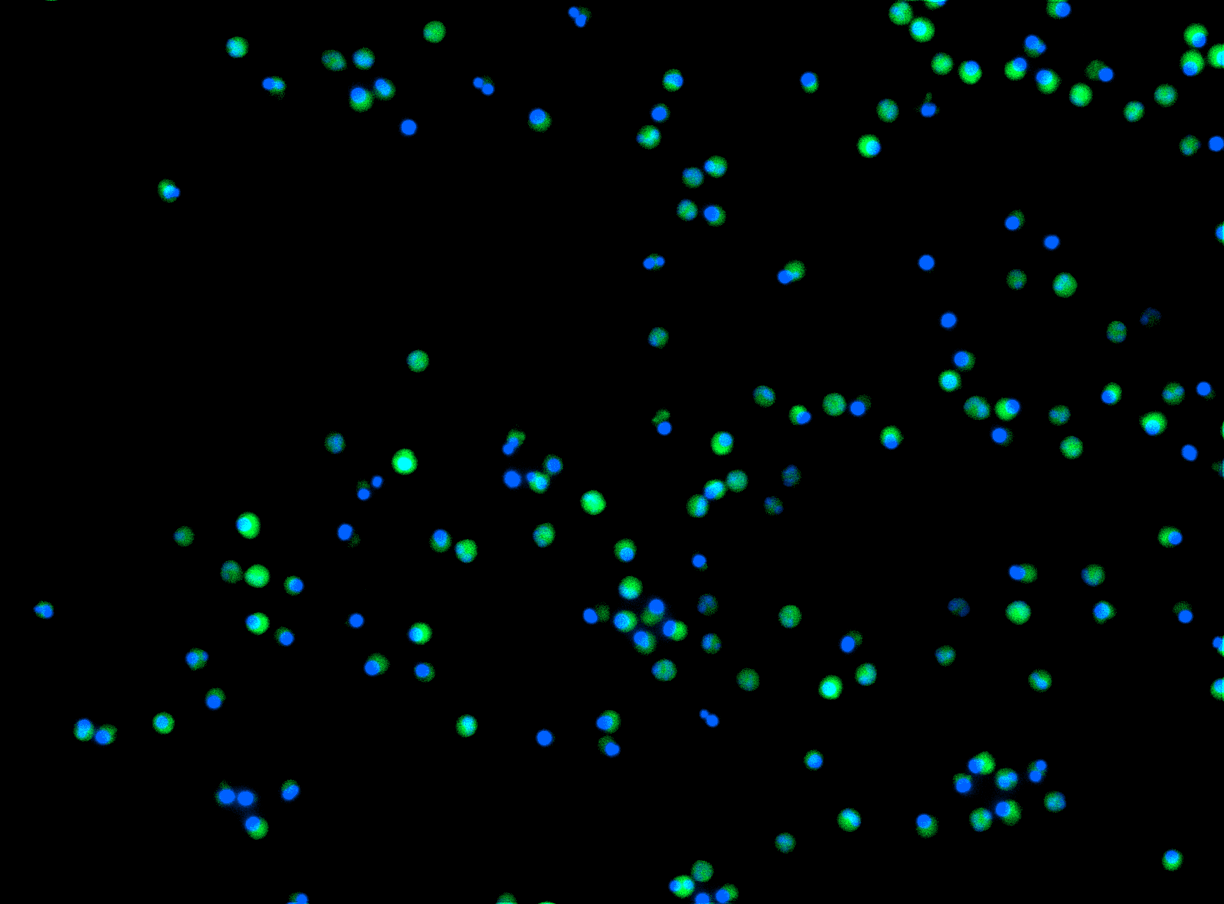

Supplement: Supplementary file 13 — Source Data for EV and Appendix figures [file 44321_2024_117_MOESM13_ESM.zip › Source Data for Expanded View and Appendix 5-23 f/Appendix Figures Source Data/Figure S3/Figure S3A/tPA_24h.tif]

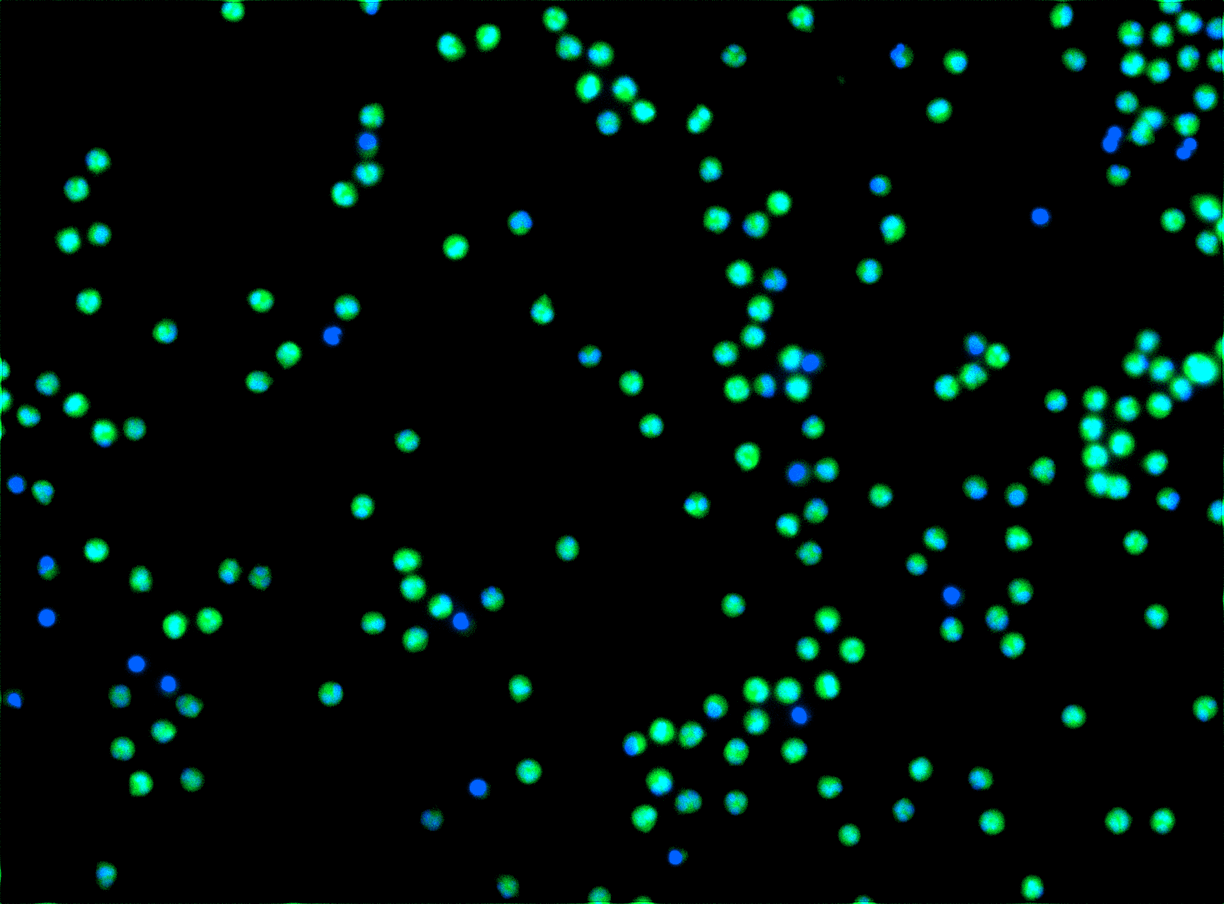

Supplement: Supplementary file 13 — Source Data for EV and Appendix figures [file 44321_2024_117_MOESM13_ESM.zip › Source Data for Expanded View and Appendix 5-23 f/Appendix Figures Source Data/Figure S3/Figure S3A/tPA_4h.tif]

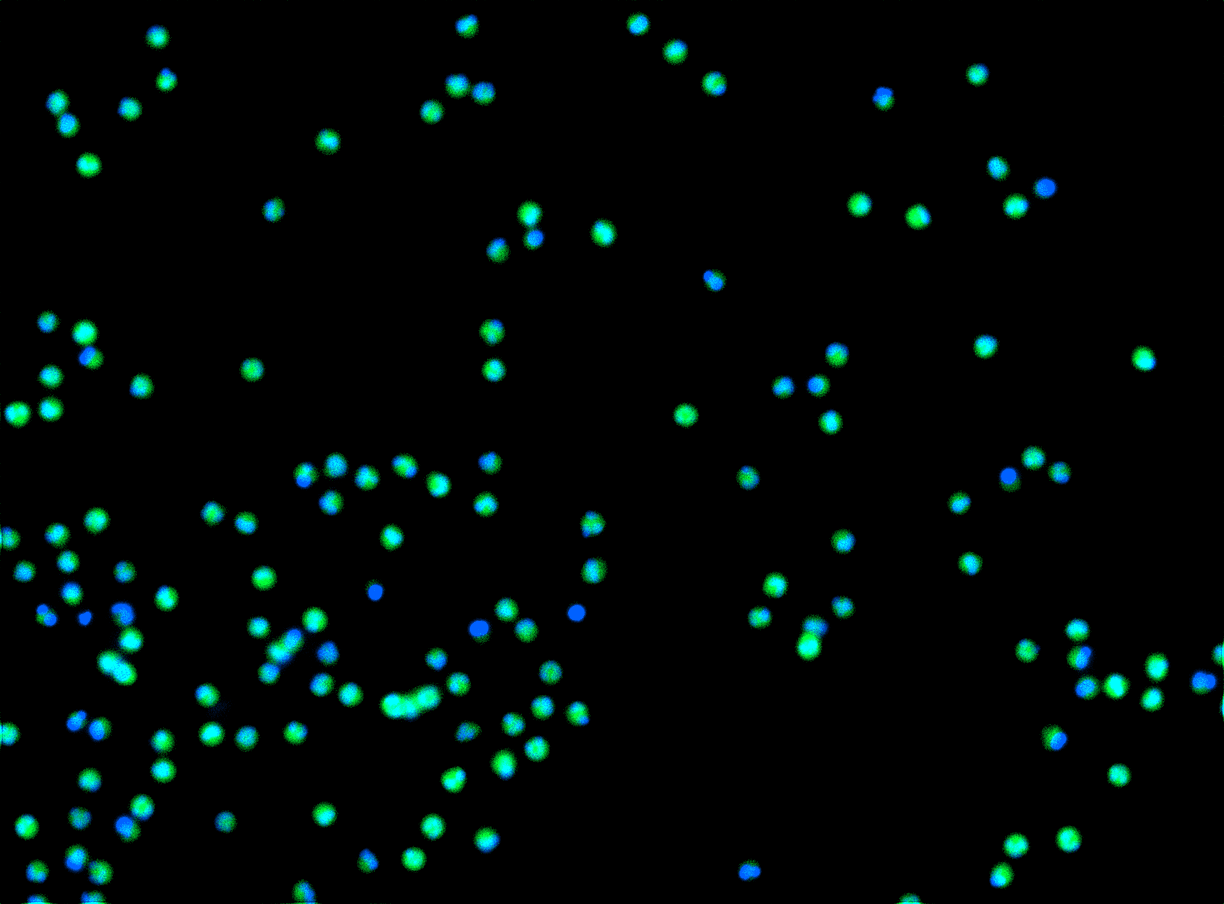

Supplement: Supplementary file 13 — Source Data for EV and Appendix figures [file 44321_2024_117_MOESM13_ESM.zip › Source Data for Expanded View and Appendix 5-23 f/Appendix Figures Source Data/Figure S3/Figure S3A/tPA_8h.tif]

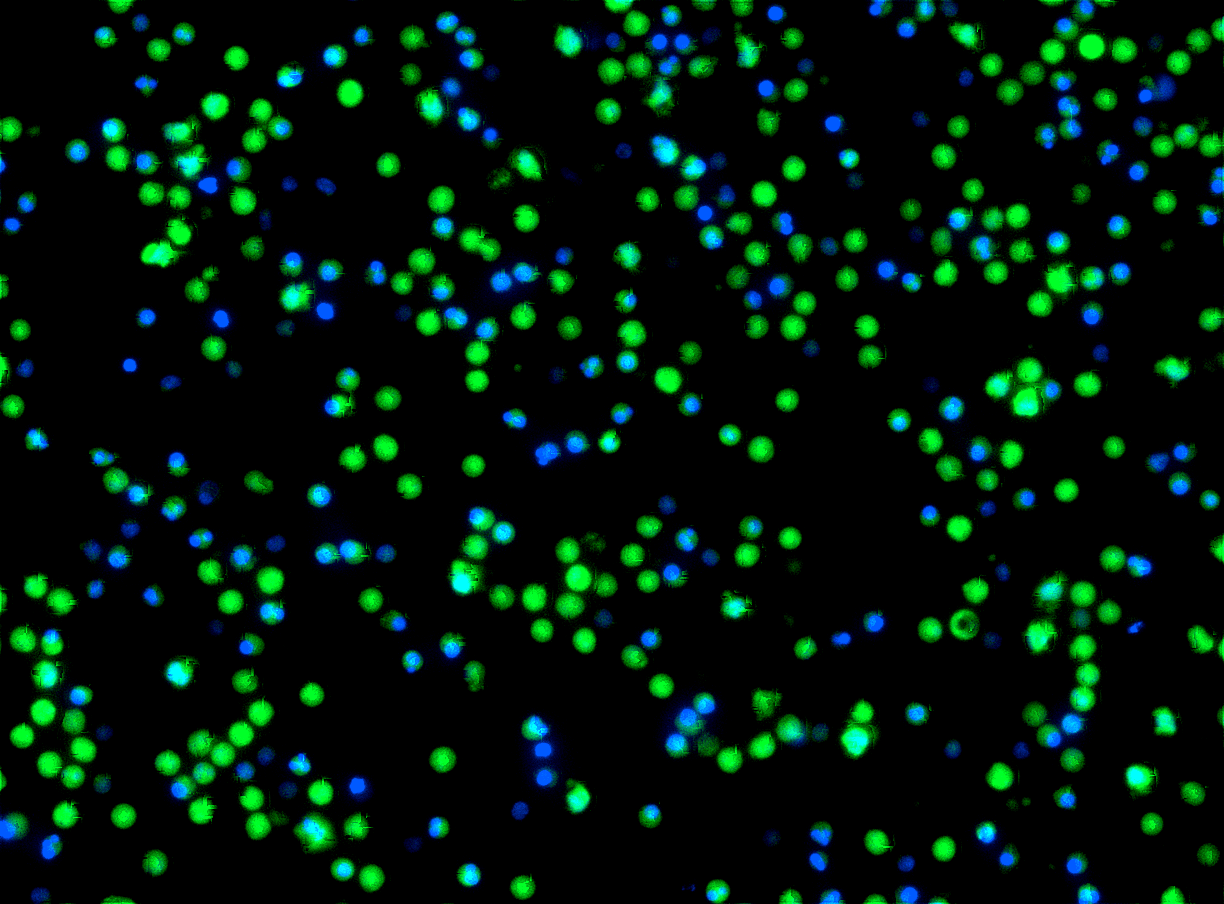

Supplement: Supplementary file 13 — Source Data for EV and Appendix figures [file 44321_2024_117_MOESM13_ESM.zip › Source Data for Expanded View and Appendix 5-23 f/Appendix Figures Source Data/Figure S4/Figure S4A/Control.tif]

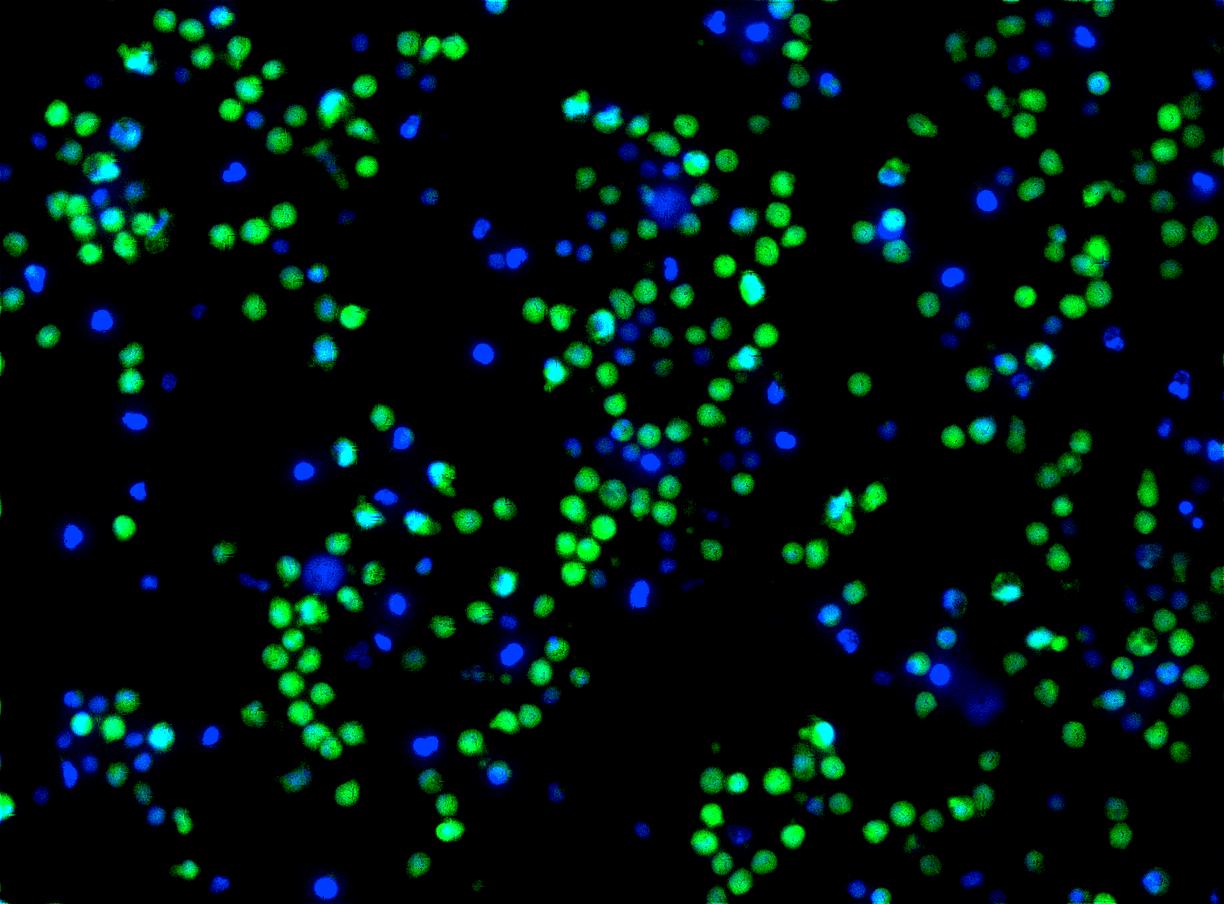

Supplement: Supplementary file 13 — Source Data for EV and Appendix figures [file 44321_2024_117_MOESM13_ESM.zip › Source Data for Expanded View and Appendix 5-23 f/Appendix Figures Source Data/Figure S4/Figure S4A/LPS.tif]

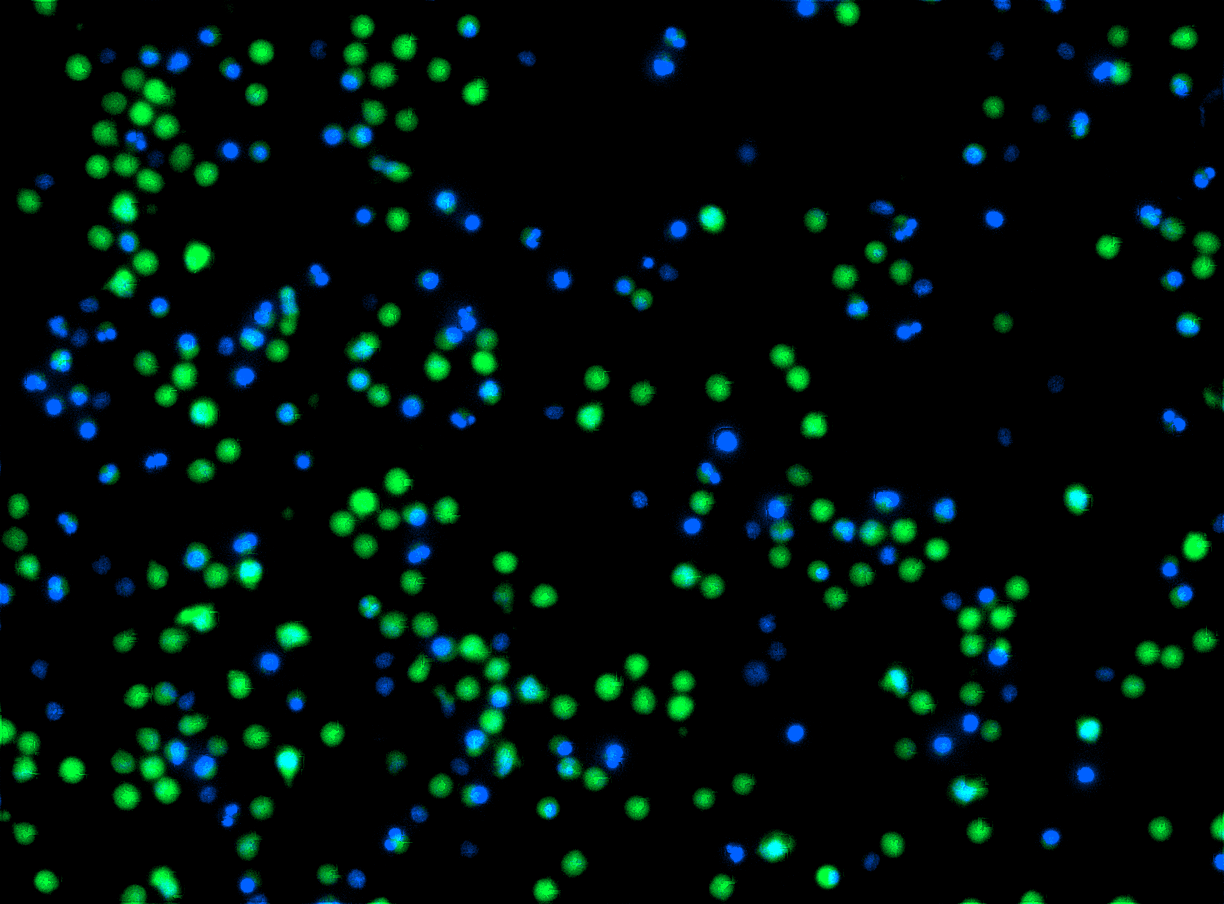

Supplement: Supplementary file 13 — Source Data for EV and Appendix figures [file 44321_2024_117_MOESM13_ESM.zip › Source Data for Expanded View and Appendix 5-23 f/Appendix Figures Source Data/Figure S4/Figure S4A/tPA100μg_ml.tif]

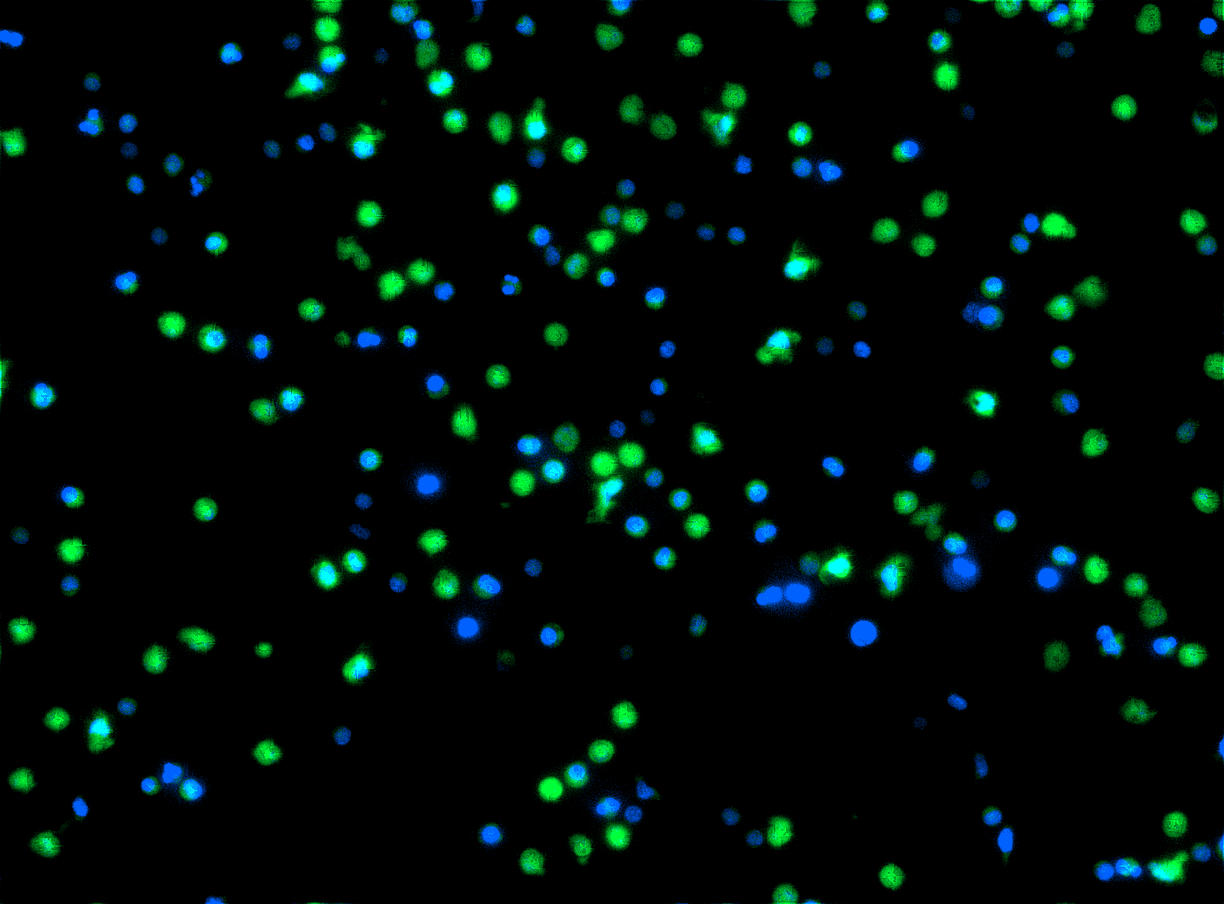

Supplement: Supplementary file 13 — Source Data for EV and Appendix figures [file 44321_2024_117_MOESM13_ESM.zip › Source Data for Expanded View and Appendix 5-23 f/Appendix Figures Source Data/Figure S4/Figure S4A/tPA10μg_ml.tif]

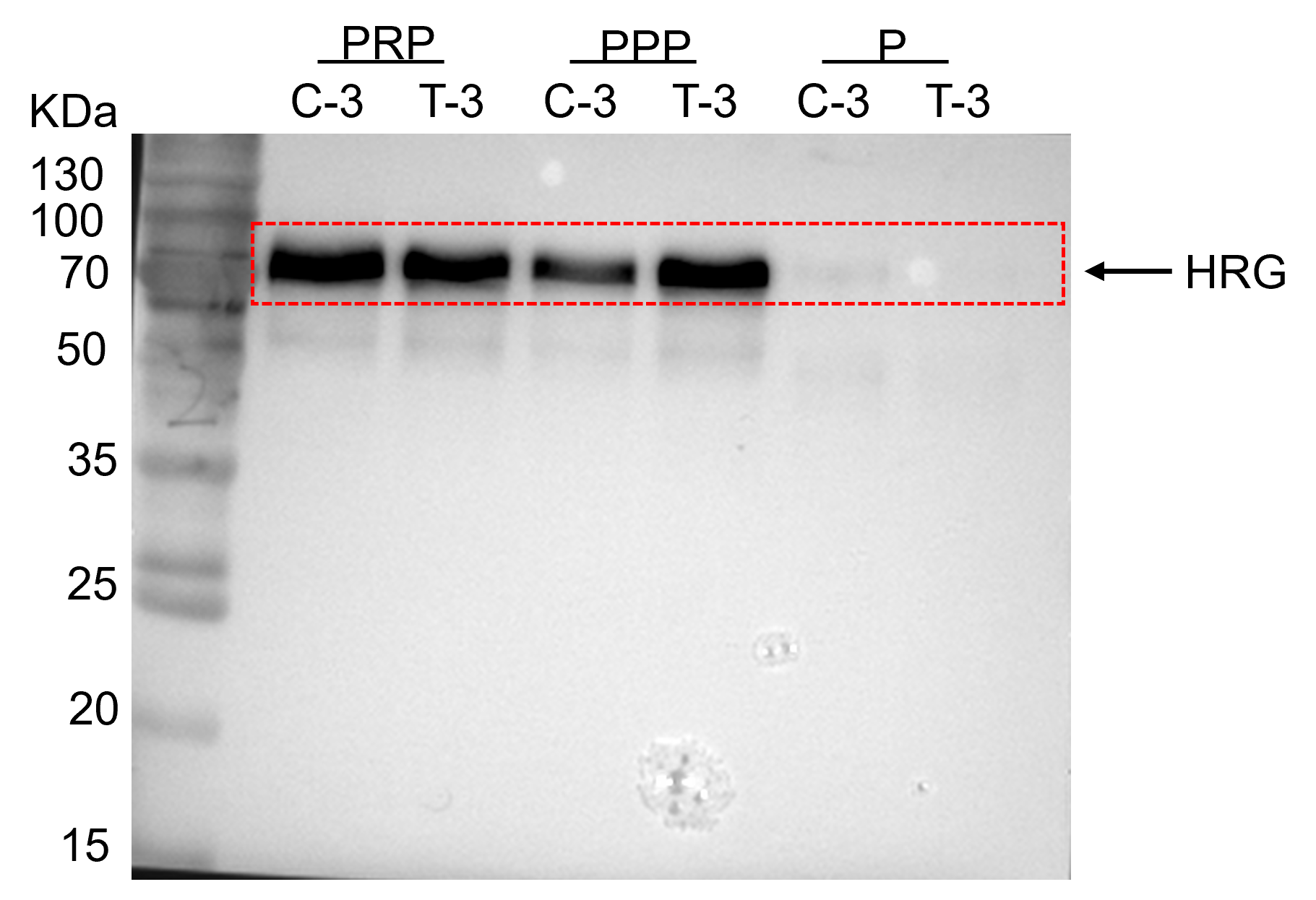

Supplement: Supplementary file 13 — Source Data for EV and Appendix figures [file 44321_2024_117_MOESM13_ESM.zip › Source Data for Expanded View and Appendix 5-23 f/Appendix Figures Source Data/Figure S6/Figure S6C/WB1 for FigS6C.tif]

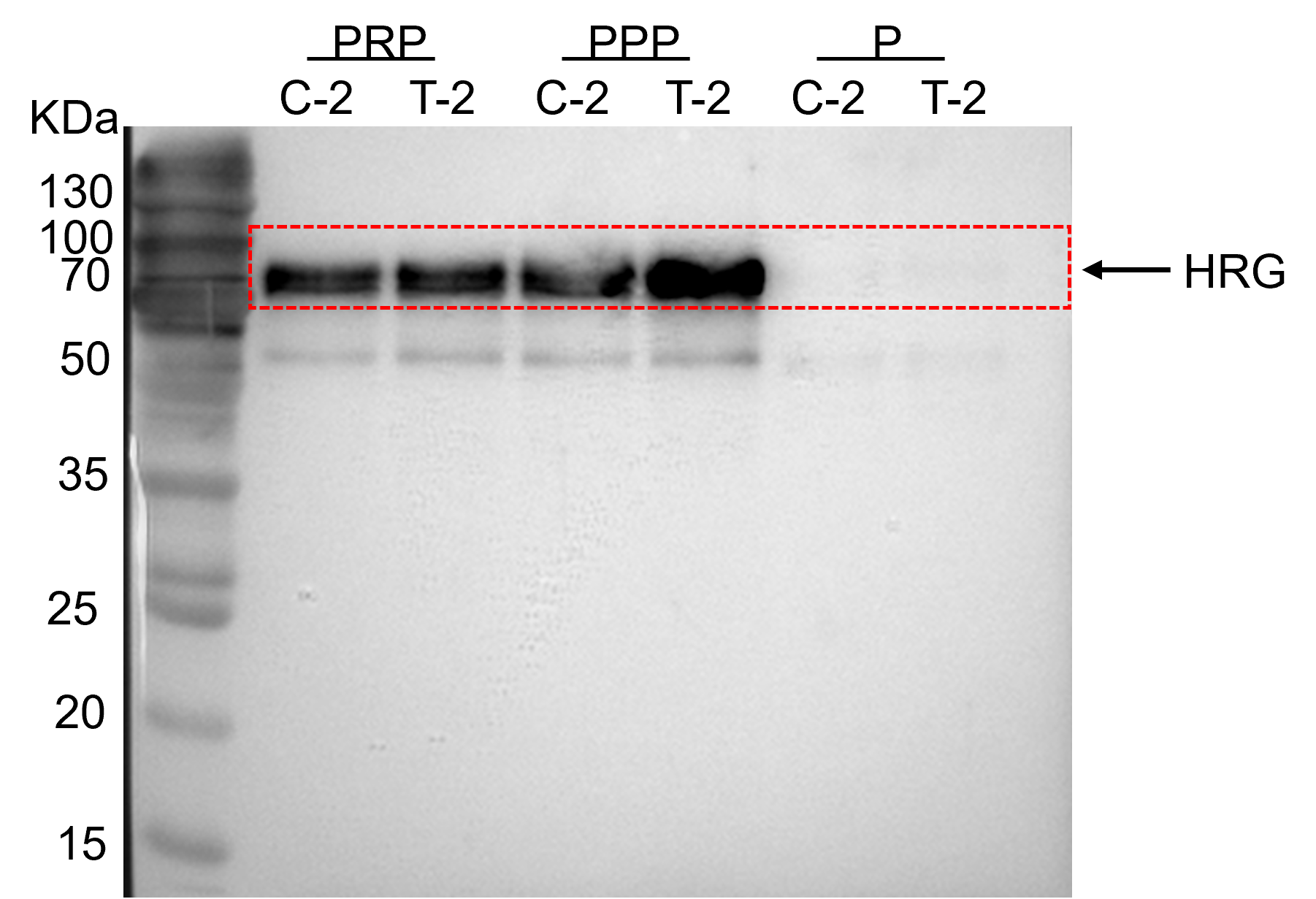

Supplement: Supplementary file 13 — Source Data for EV and Appendix figures [file 44321_2024_117_MOESM13_ESM.zip › Source Data for Expanded View and Appendix 5-23 f/Appendix Figures Source Data/Figure S6/Figure S6C/WB2.tif]

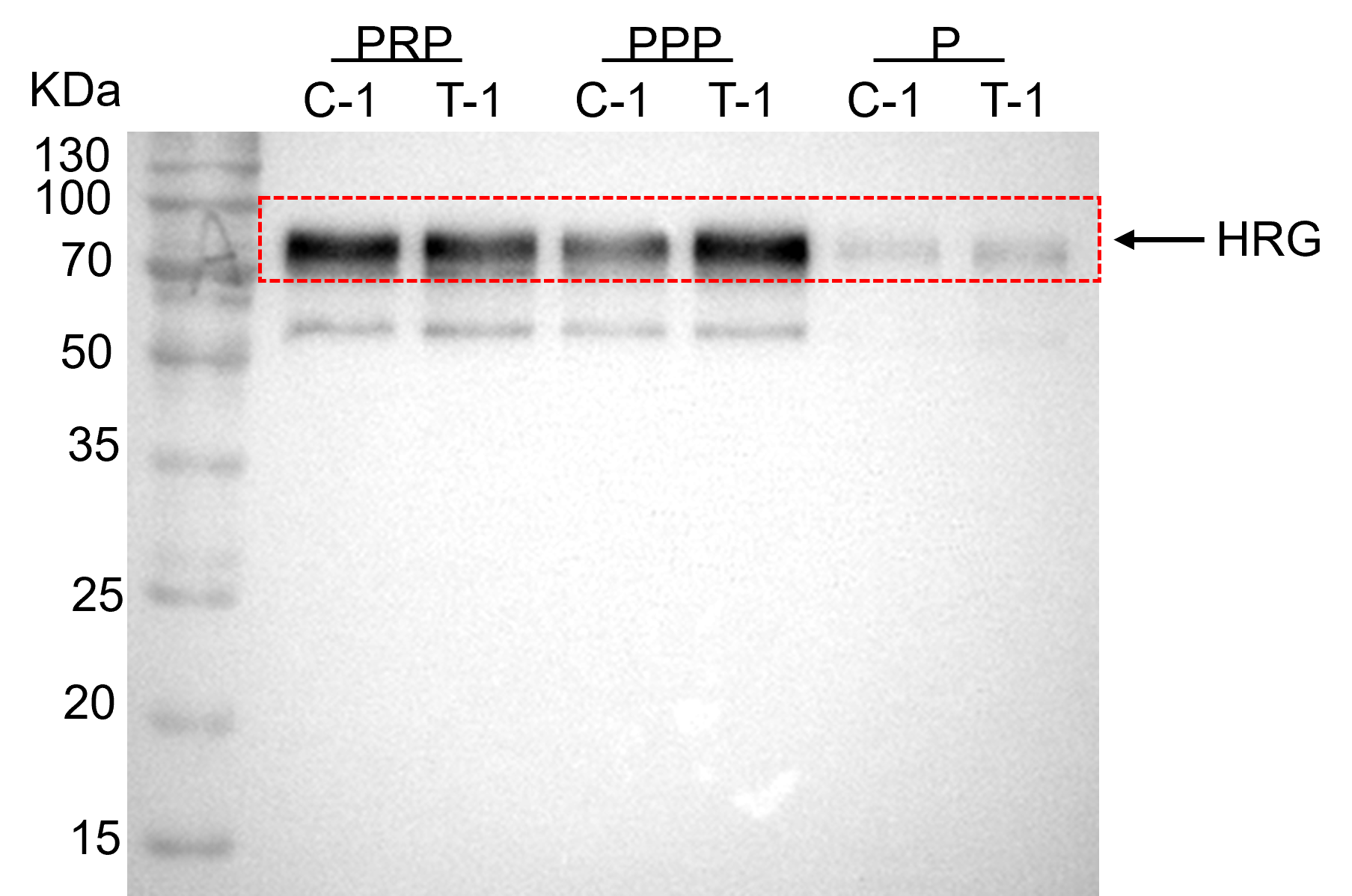

Supplement: Supplementary file 13 — Source Data for EV and Appendix figures [file 44321_2024_117_MOESM13_ESM.zip › Source Data for Expanded View and Appendix 5-23 f/Appendix Figures Source Data/Figure S6/Figure S6C/WB3.tif]

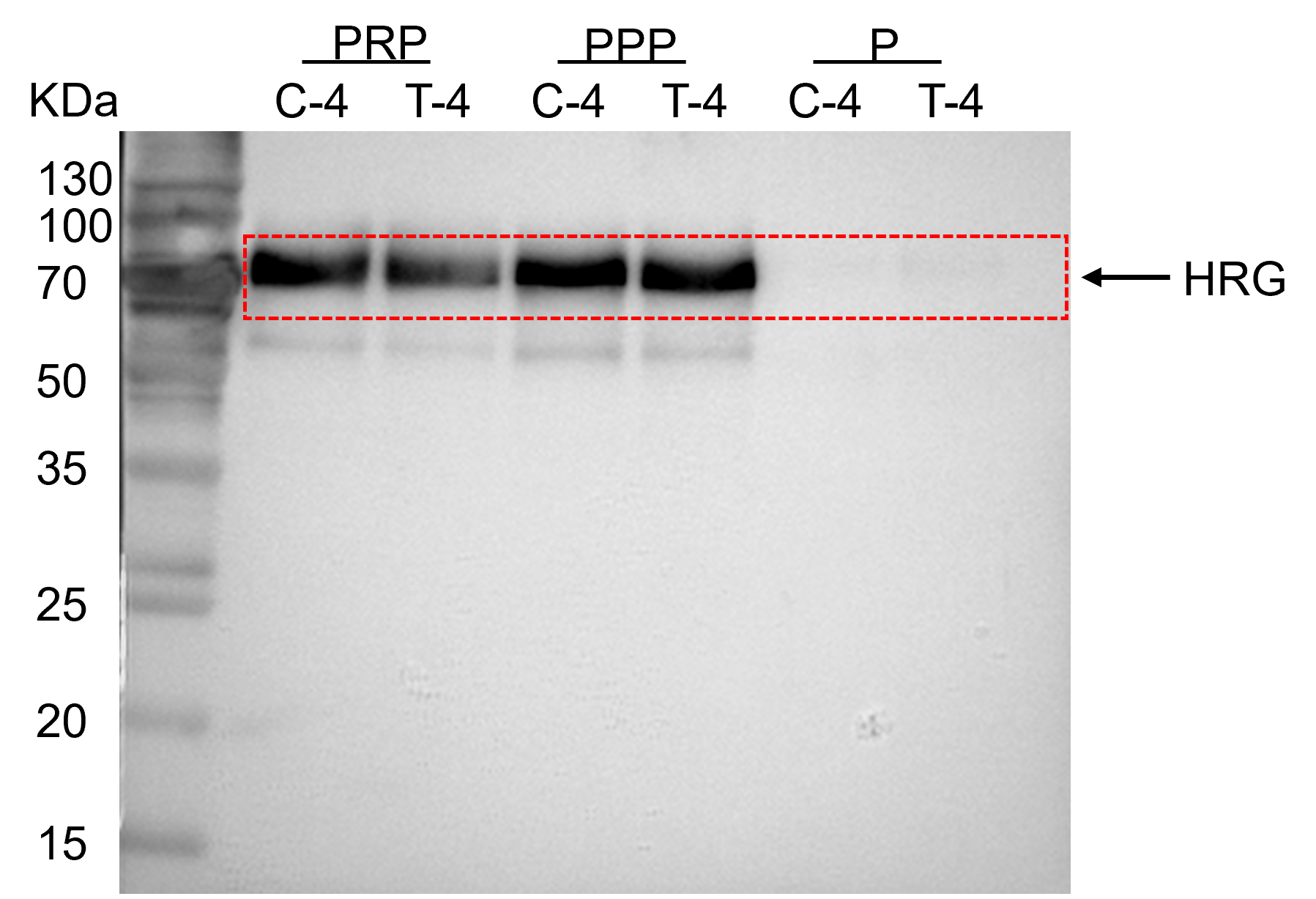

Supplement: Supplementary file 13 — Source Data for EV and Appendix figures [file 44321_2024_117_MOESM13_ESM.zip › Source Data for Expanded View and Appendix 5-23 f/Appendix Figures Source Data/Figure S6/Figure S6C/WB4.tif]

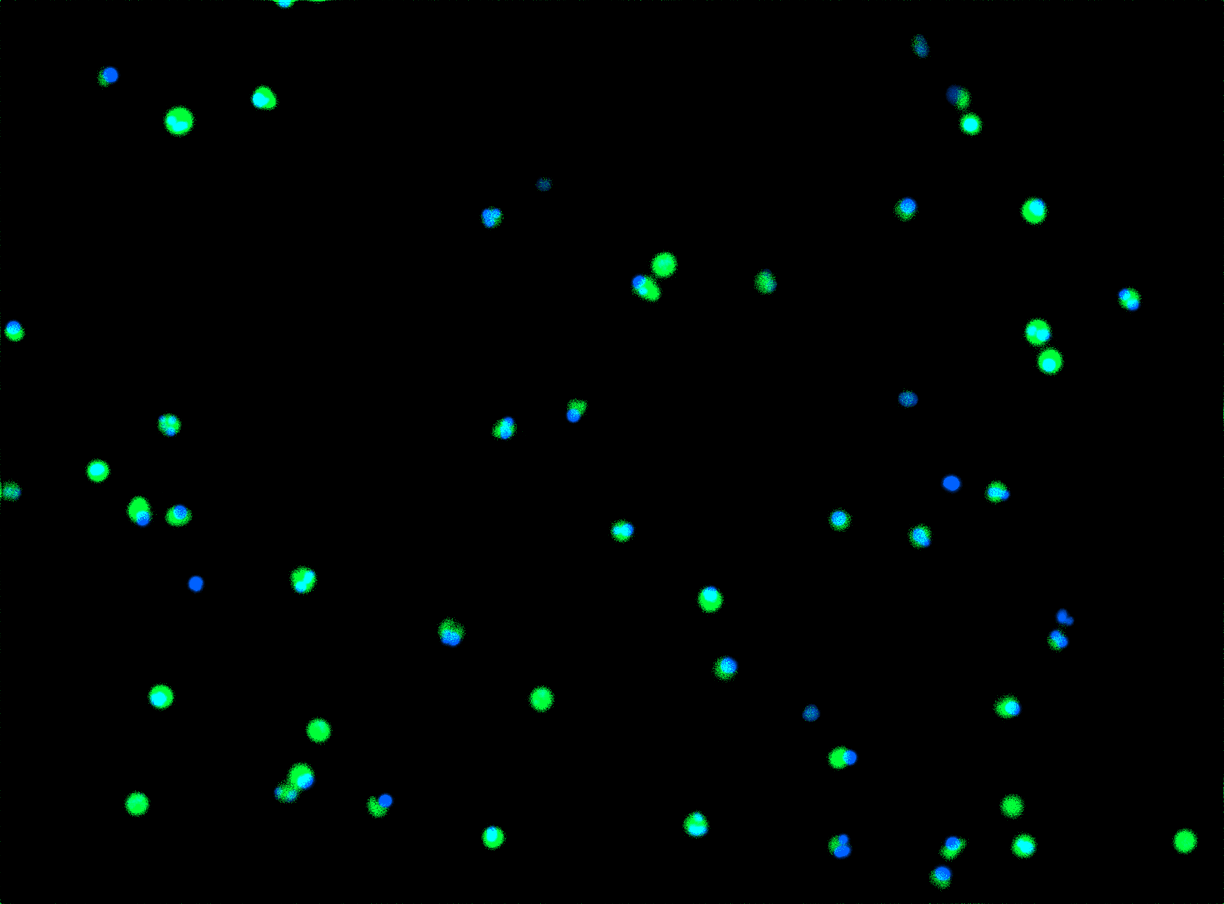

Supplement: Supplementary file 13 — Source Data for EV and Appendix figures [file 44321_2024_117_MOESM13_ESM.zip › Source Data for Expanded View and Appendix 5-23 f/Figure EV2/EV2A/Control.tif]

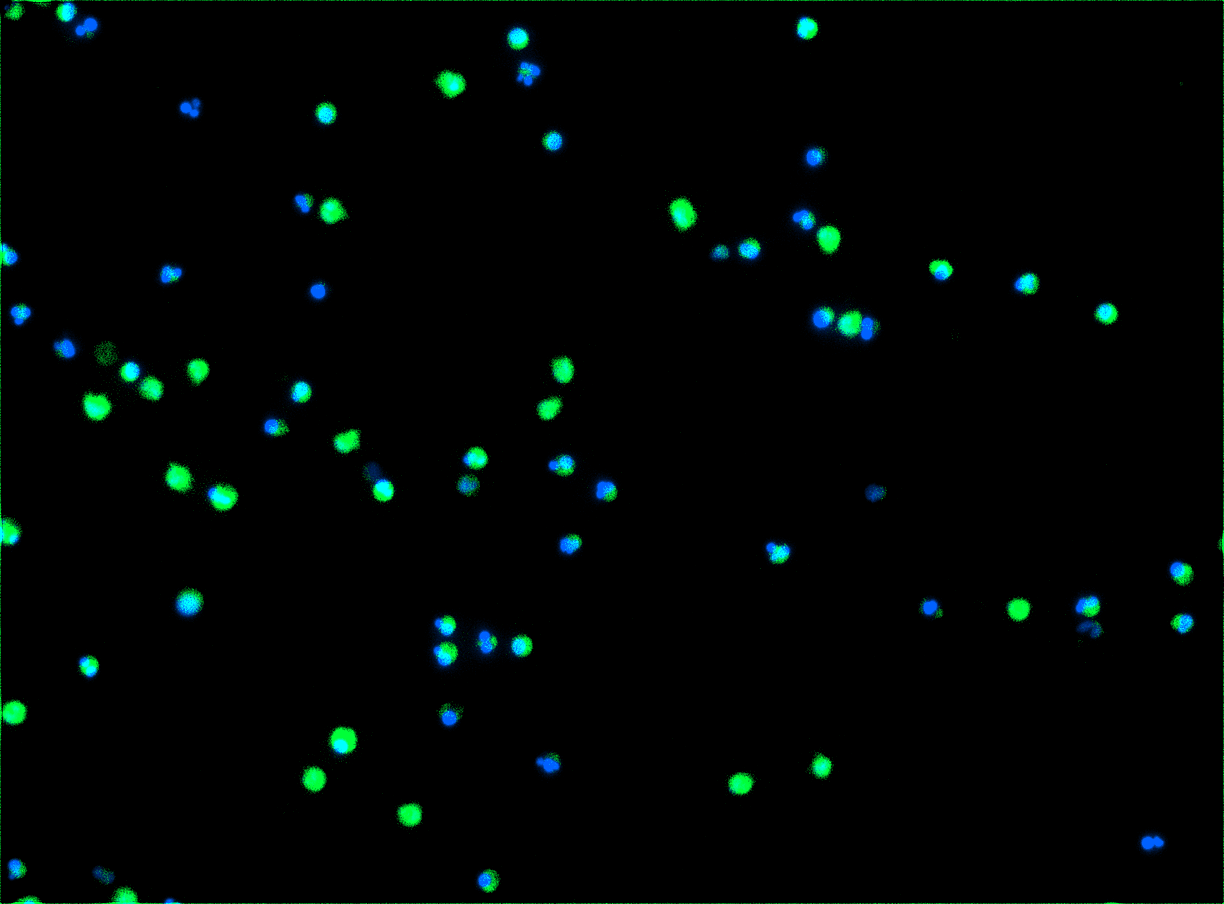

Supplement: Supplementary file 13 — Source Data for EV and Appendix figures [file 44321_2024_117_MOESM13_ESM.zip › Source Data for Expanded View and Appendix 5-23 f/Figure EV2/EV2A/tPA+HRG_CLEC1A Ab.tif]

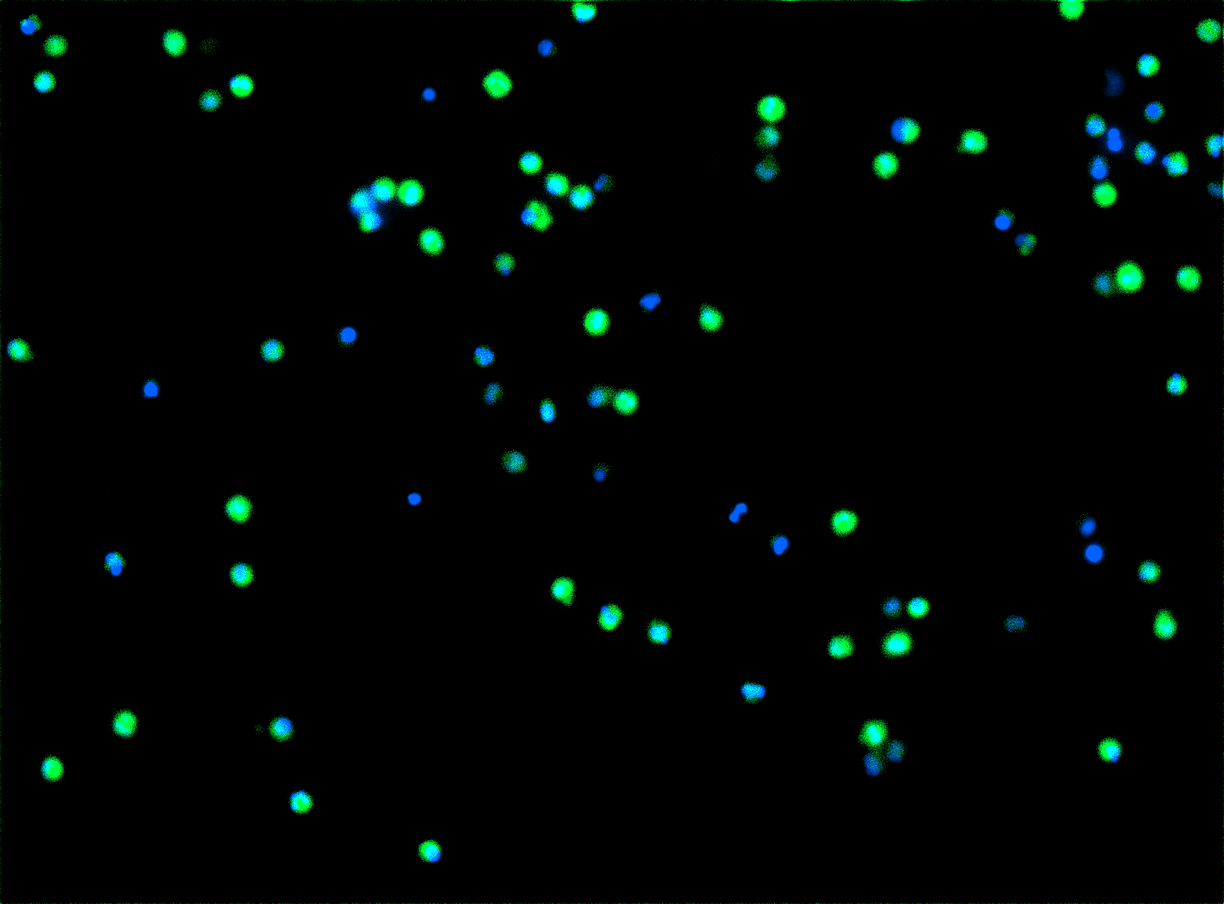

Supplement: Supplementary file 13 — Source Data for EV and Appendix figures [file 44321_2024_117_MOESM13_ESM.zip › Source Data for Expanded View and Appendix 5-23 f/Figure EV2/EV2A/tPA+HRG_CLEC1B Ab.tif]

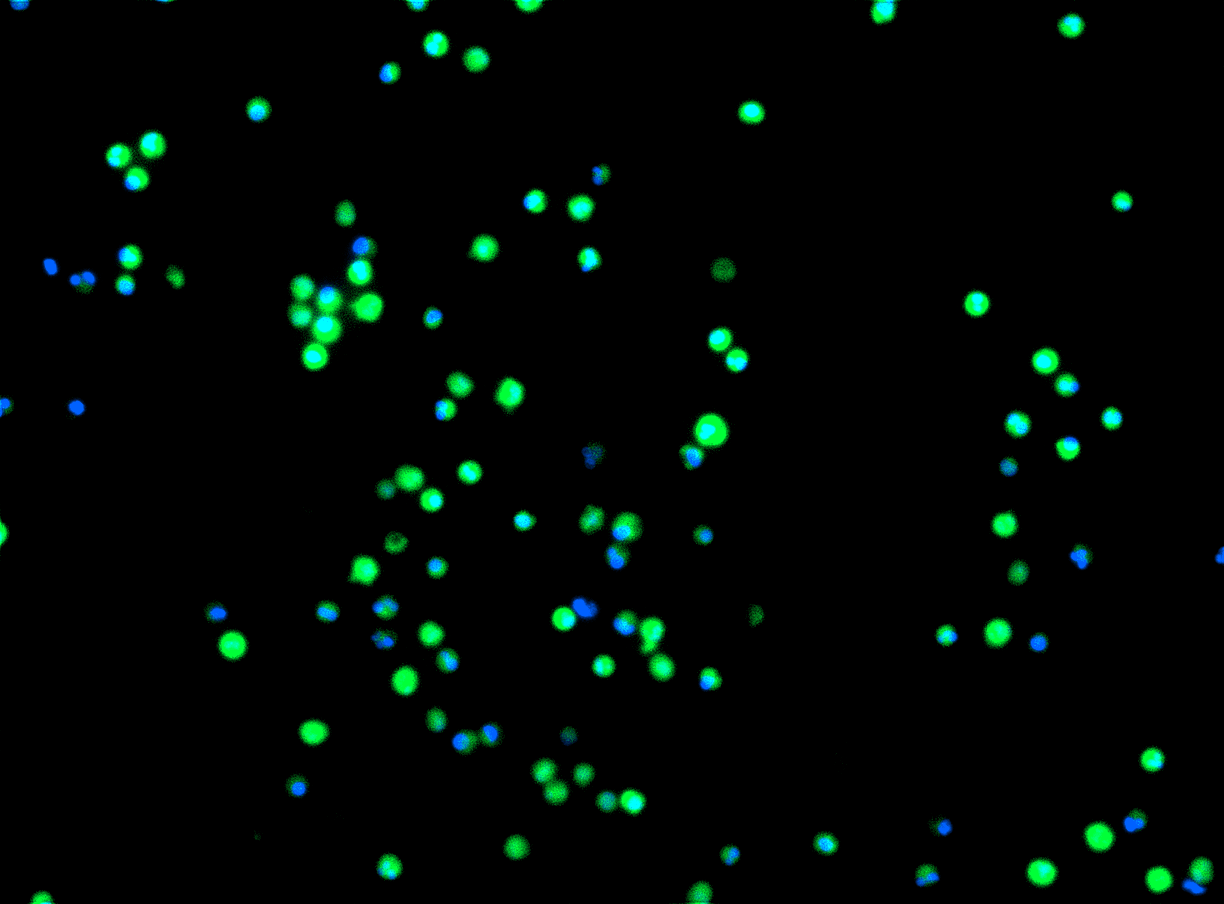

Supplement: Supplementary file 13 — Source Data for EV and Appendix figures [file 44321_2024_117_MOESM13_ESM.zip › Source Data for Expanded View and Appendix 5-23 f/Figure EV2/EV2A/tPA+HRG_Control Ab.tif]

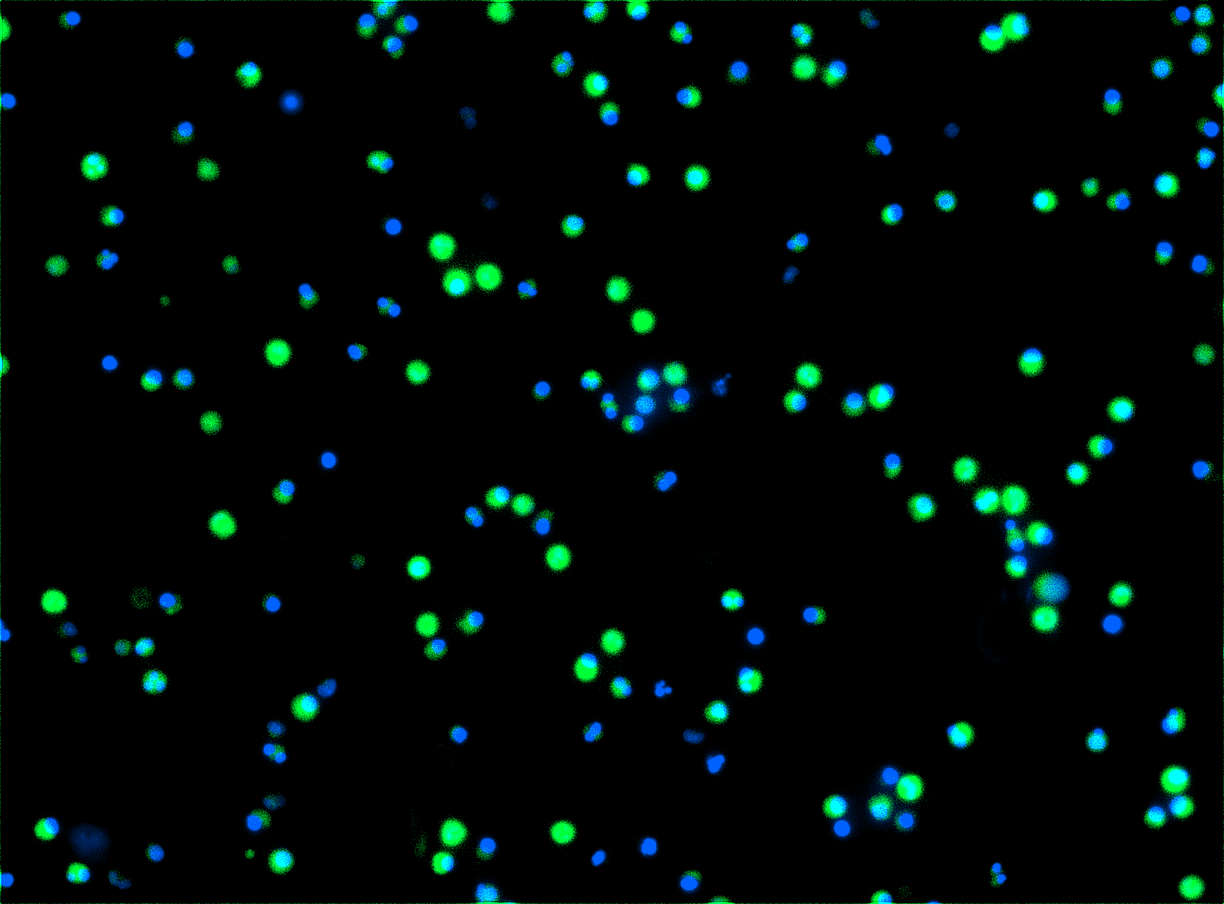

Supplement: Supplementary file 13 — Source Data for EV and Appendix figures [file 44321_2024_117_MOESM13_ESM.zip › Source Data for Expanded View and Appendix 5-23 f/Figure EV2/EV2A/tPA.tif]

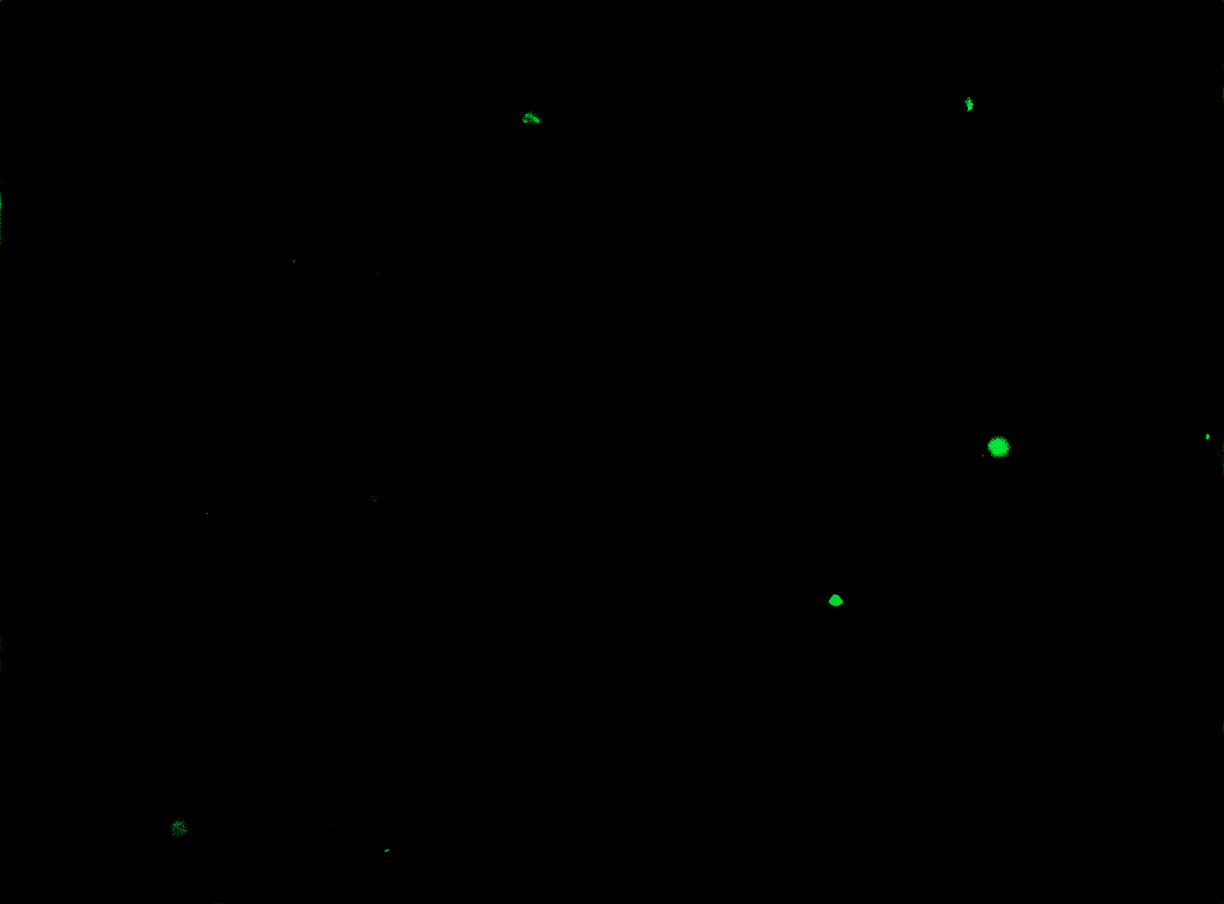

Supplement: Supplementary file 13 — Source Data for EV and Appendix figures [file 44321_2024_117_MOESM13_ESM.zip › Source Data for Expanded View and Appendix 5-23 f/Figure EV2/EV2C/Control_H3-Cit.tif]

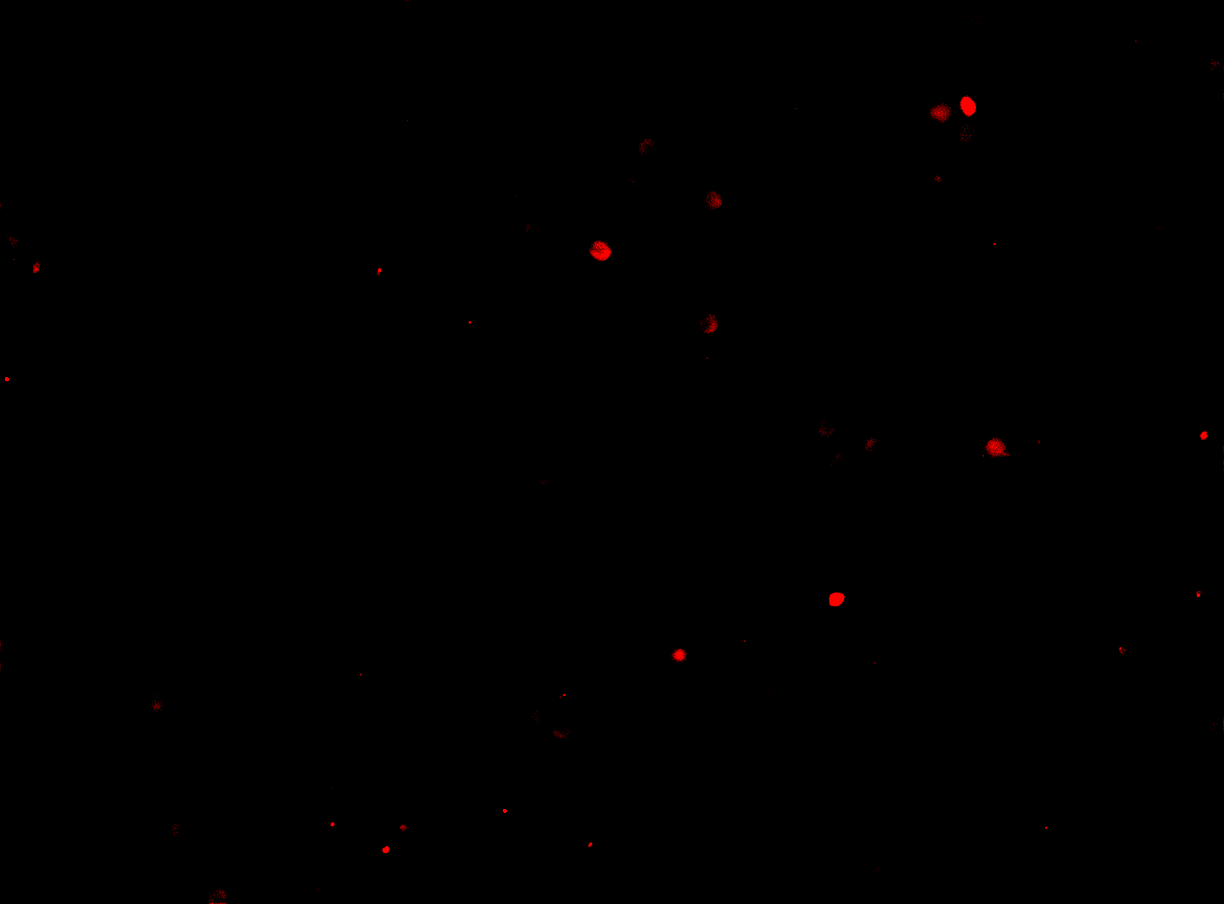

Supplement: Supplementary file 13 — Source Data for EV and Appendix figures [file 44321_2024_117_MOESM13_ESM.zip › Source Data for Expanded View and Appendix 5-23 f/Figure EV2/EV2C/Control_MPO.tif]

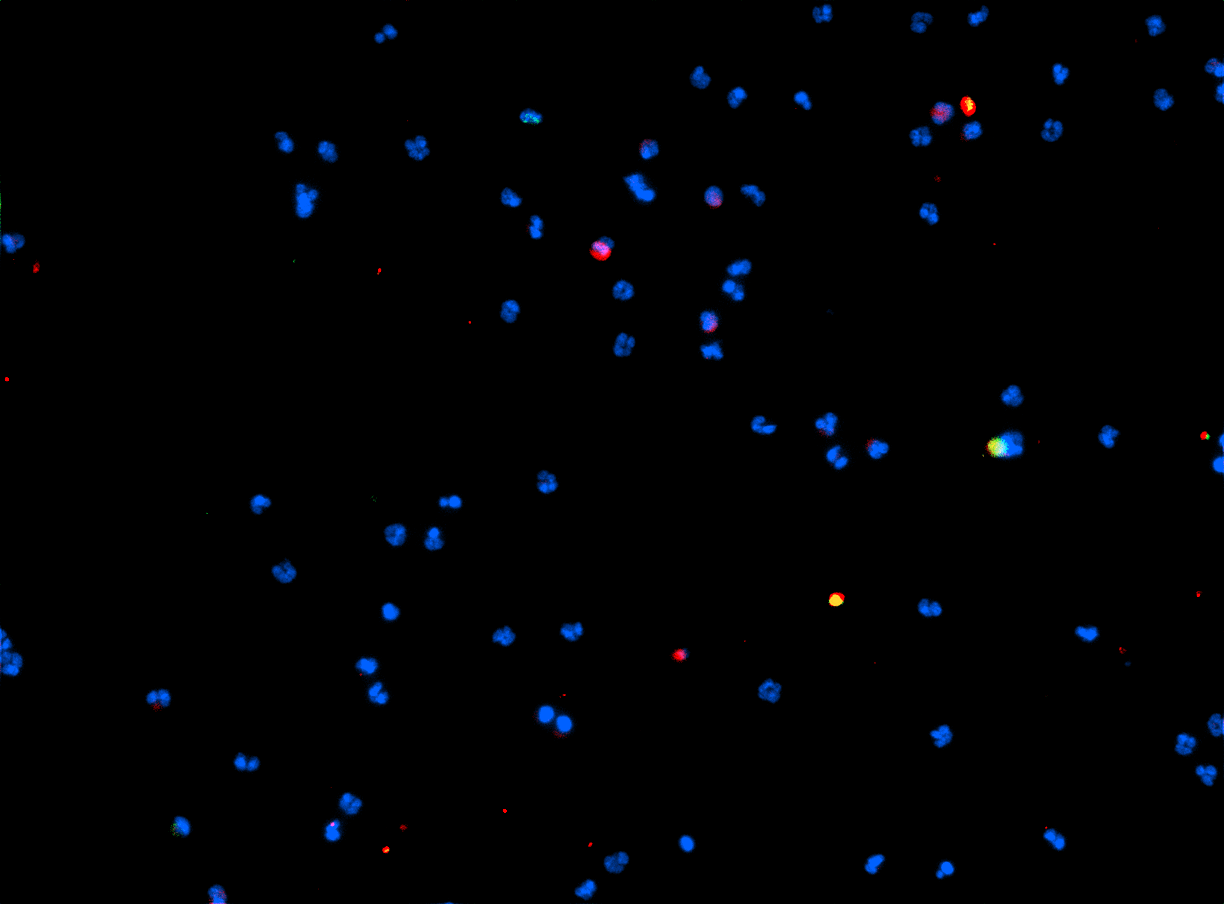

Supplement: Supplementary file 13 — Source Data for EV and Appendix figures [file 44321_2024_117_MOESM13_ESM.zip › Source Data for Expanded View and Appendix 5-23 f/Figure EV2/EV2C/Control_Merge.tif]

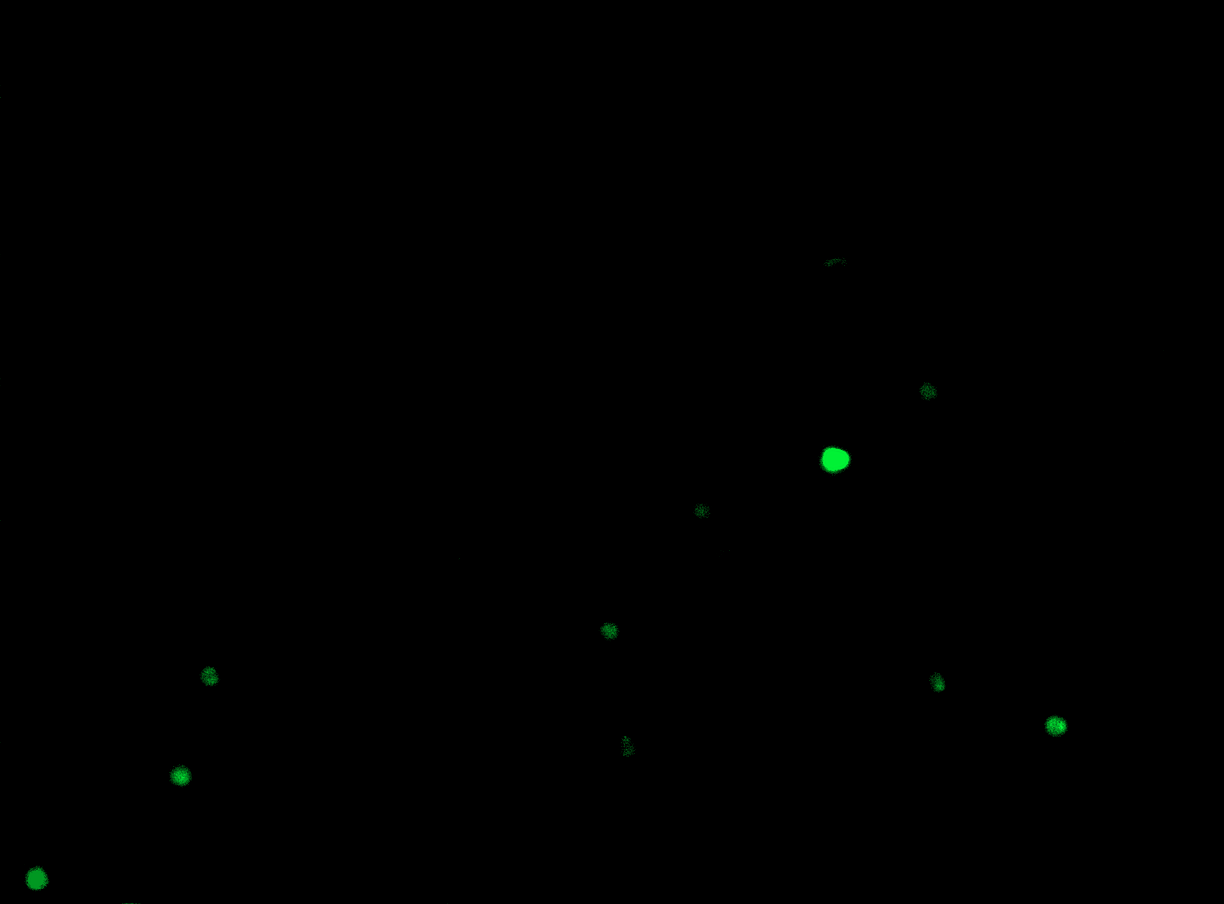

Supplement: Supplementary file 13 — Source Data for EV and Appendix figures [file 44321_2024_117_MOESM13_ESM.zip › Source Data for Expanded View and Appendix 5-23 f/Figure EV2/EV2C/tPA+HRG+CLEC1A Ab_H3-Cit.tif]

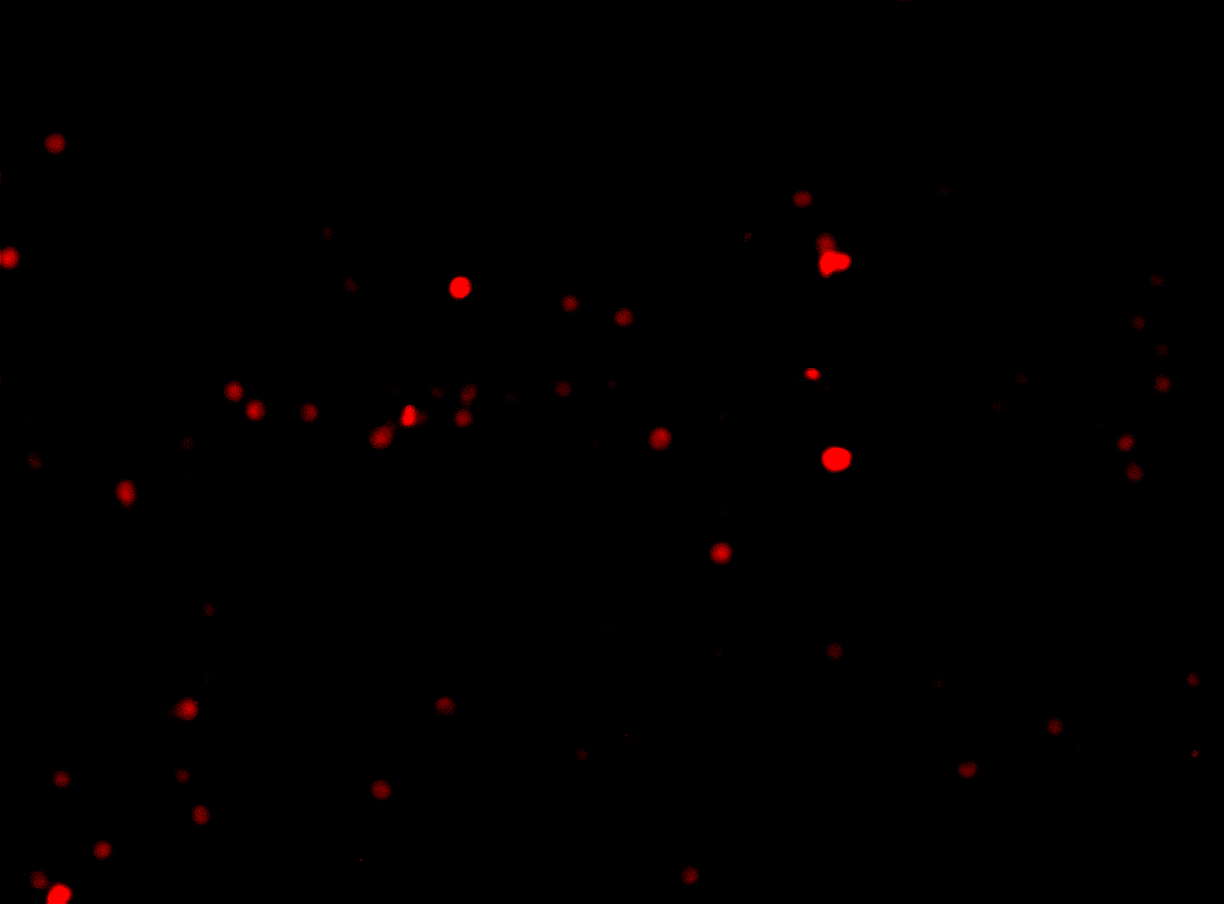

Supplement: Supplementary file 13 — Source Data for EV and Appendix figures [file 44321_2024_117_MOESM13_ESM.zip › Source Data for Expanded View and Appendix 5-23 f/Figure EV2/EV2C/tPA+HRG+CLEC1A Ab_MPO.tif]

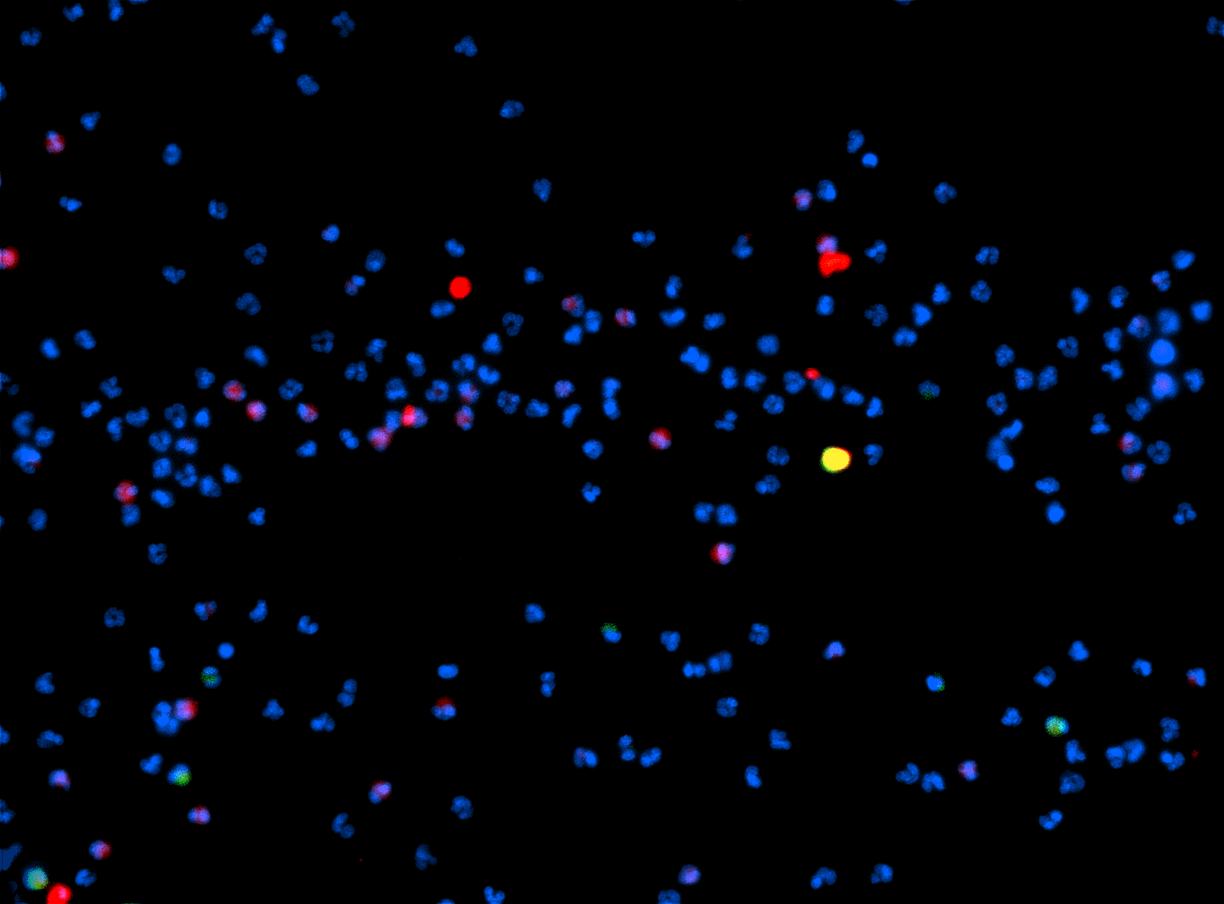

Supplement: Supplementary file 13 — Source Data for EV and Appendix figures [file 44321_2024_117_MOESM13_ESM.zip › Source Data for Expanded View and Appendix 5-23 f/Figure EV2/EV2C/tPA+HRG+CLEC1A Ab_Merge.tif]

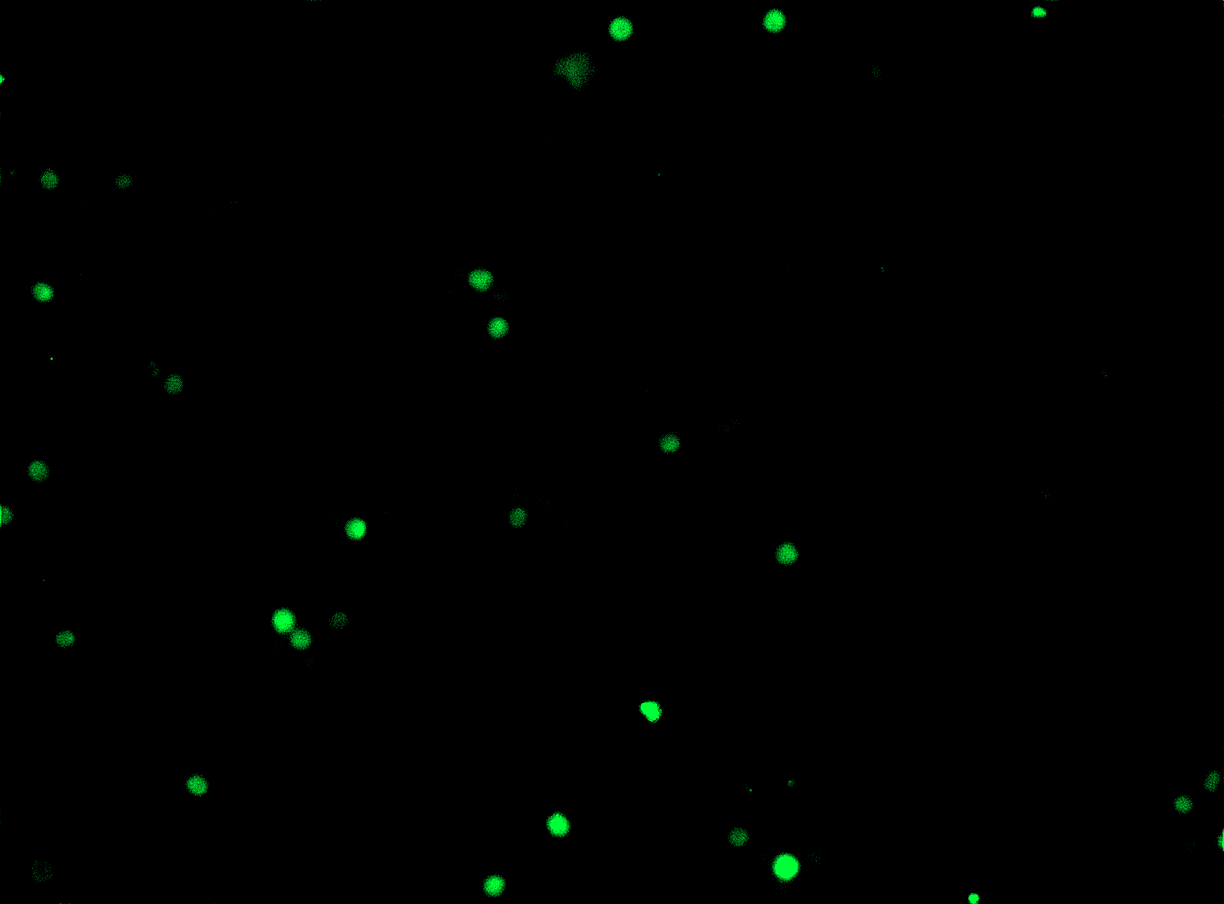

Supplement: Supplementary file 13 — Source Data for EV and Appendix figures [file 44321_2024_117_MOESM13_ESM.zip › Source Data for Expanded View and Appendix 5-23 f/Figure EV2/EV2C/tPA+HRG+CLEC1B Ab_H3-Cit.tif]

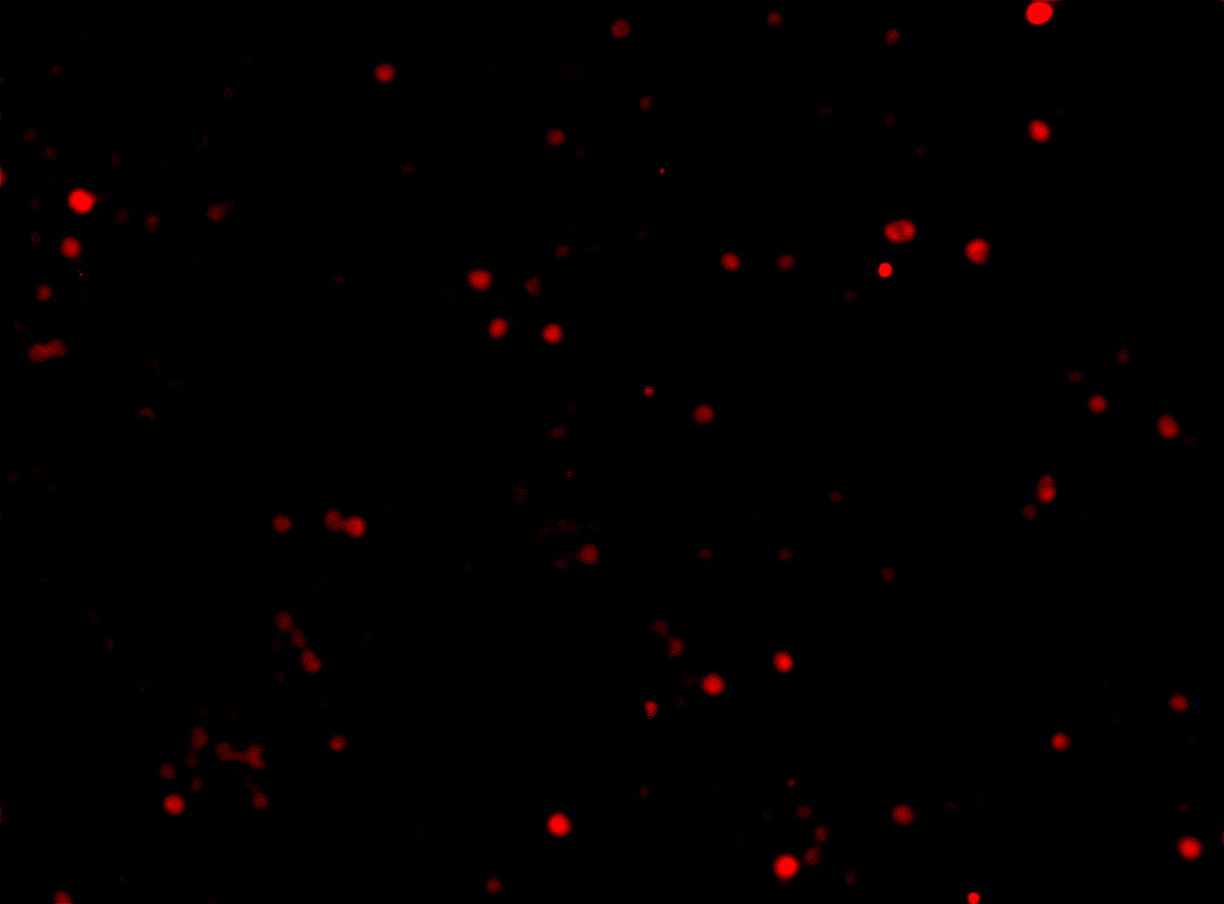

Supplement: Supplementary file 13 — Source Data for EV and Appendix figures [file 44321_2024_117_MOESM13_ESM.zip › Source Data for Expanded View and Appendix 5-23 f/Figure EV2/EV2C/tPA+HRG+CLEC1B Ab_MPO.tif]

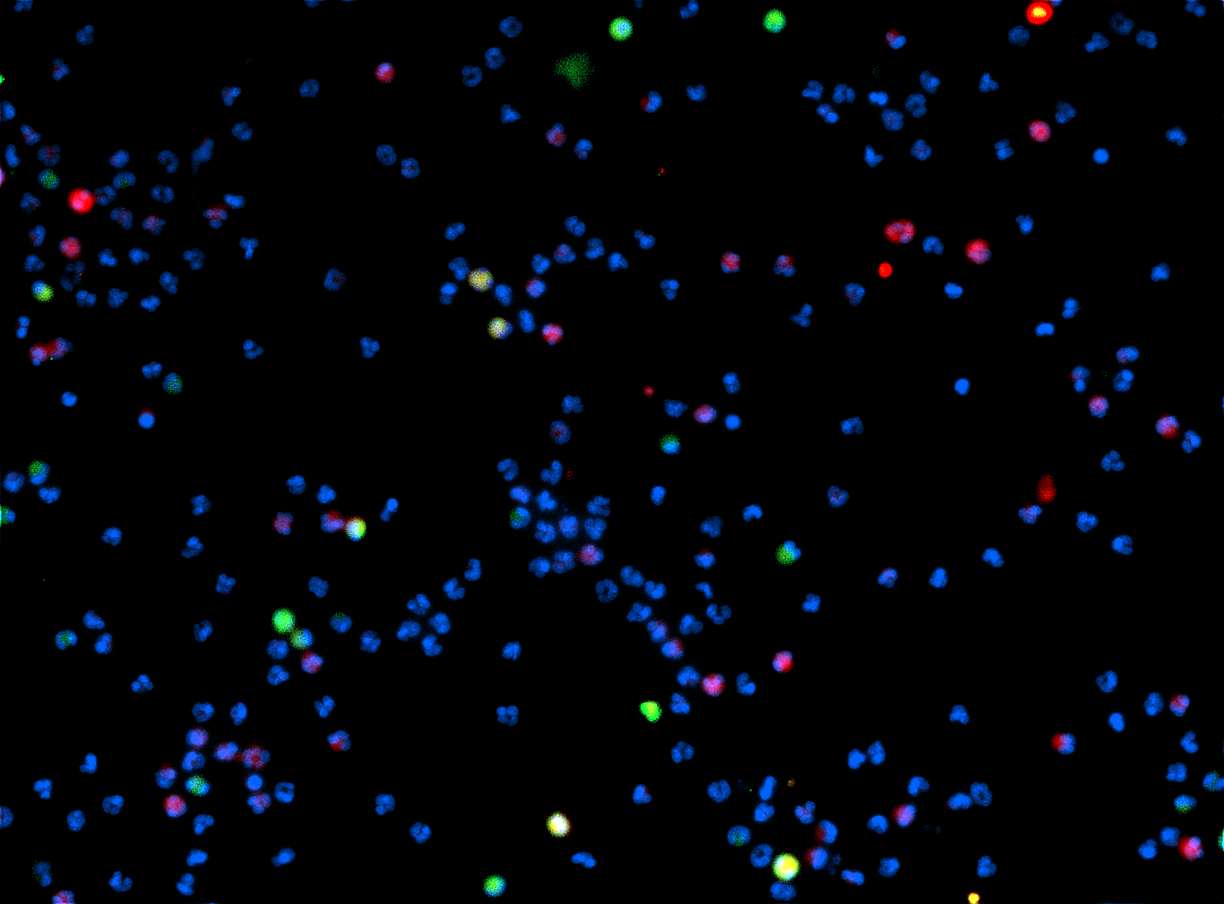

Supplement: Supplementary file 13 — Source Data for EV and Appendix figures [file 44321_2024_117_MOESM13_ESM.zip › Source Data for Expanded View and Appendix 5-23 f/Figure EV2/EV2C/tPA+HRG+CLEC1B Ab_Merge.tif]

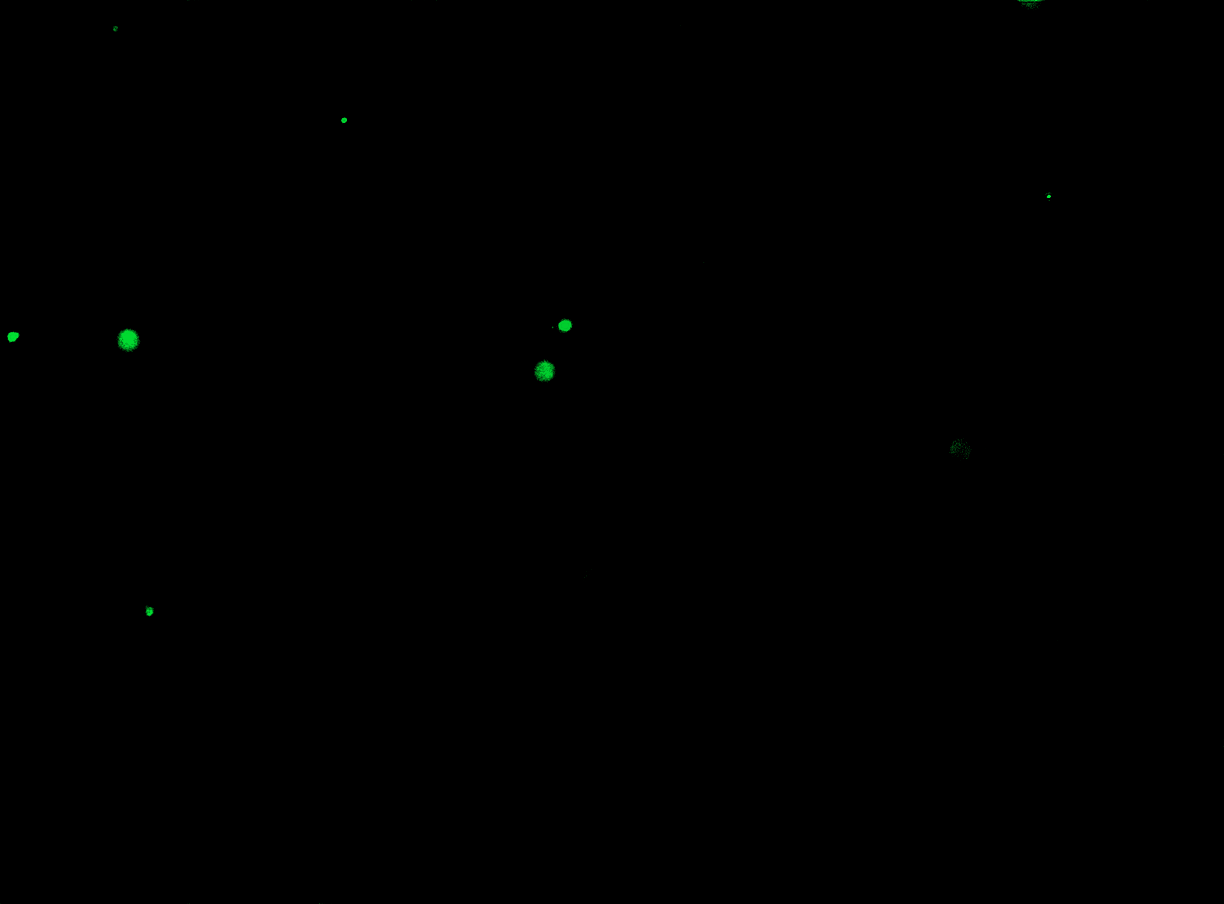

Supplement: Supplementary file 13 — Source Data for EV and Appendix figures [file 44321_2024_117_MOESM13_ESM.zip › Source Data for Expanded View and Appendix 5-23 f/Figure EV2/EV2C/tPA+HRG_H3-Cit.tif]

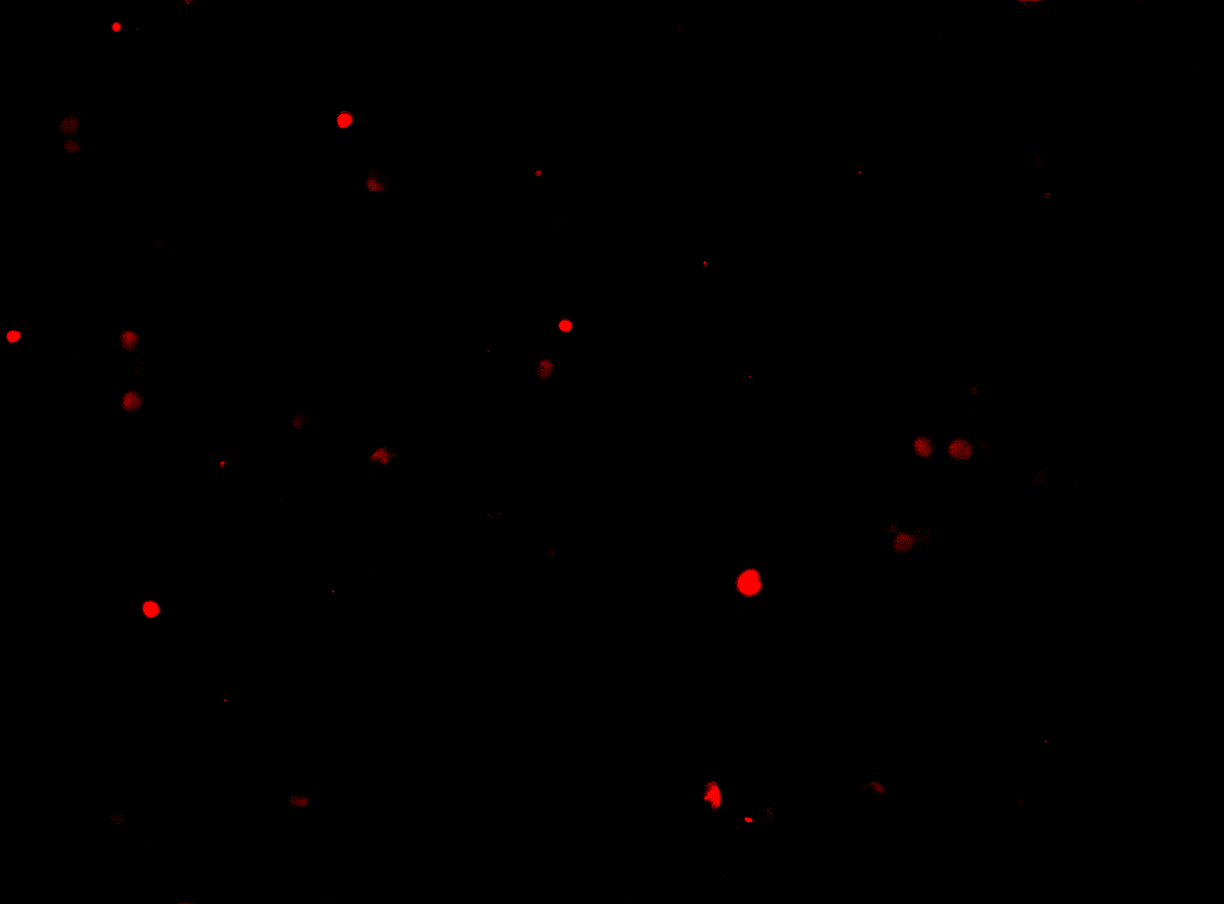

Supplement: Supplementary file 13 — Source Data for EV and Appendix figures [file 44321_2024_117_MOESM13_ESM.zip › Source Data for Expanded View and Appendix 5-23 f/Figure EV2/EV2C/tPA+HRG_MPO.tif]

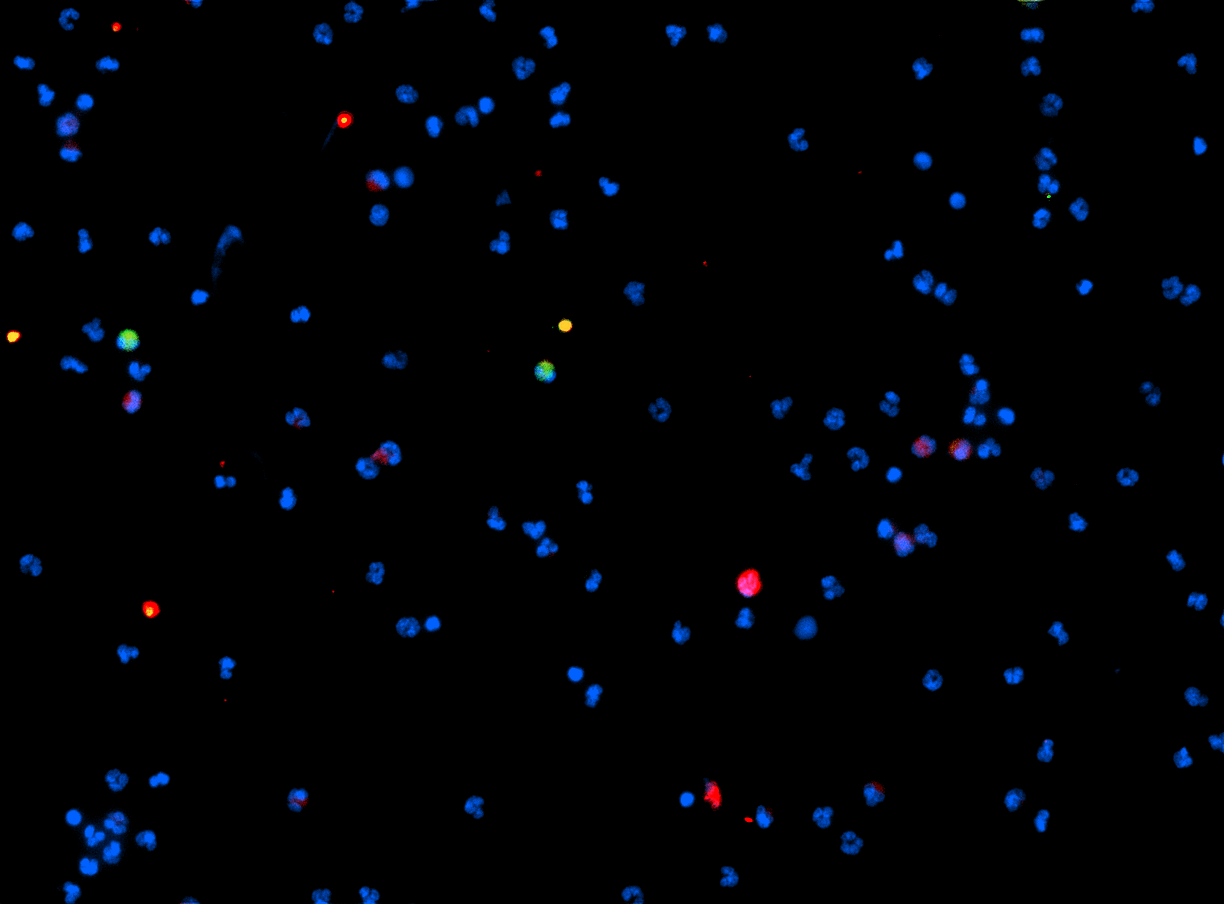

Supplement: Supplementary file 13 — Source Data for EV and Appendix figures [file 44321_2024_117_MOESM13_ESM.zip › Source Data for Expanded View and Appendix 5-23 f/Figure EV2/EV2C/tPA+HRG_Merge.tif]

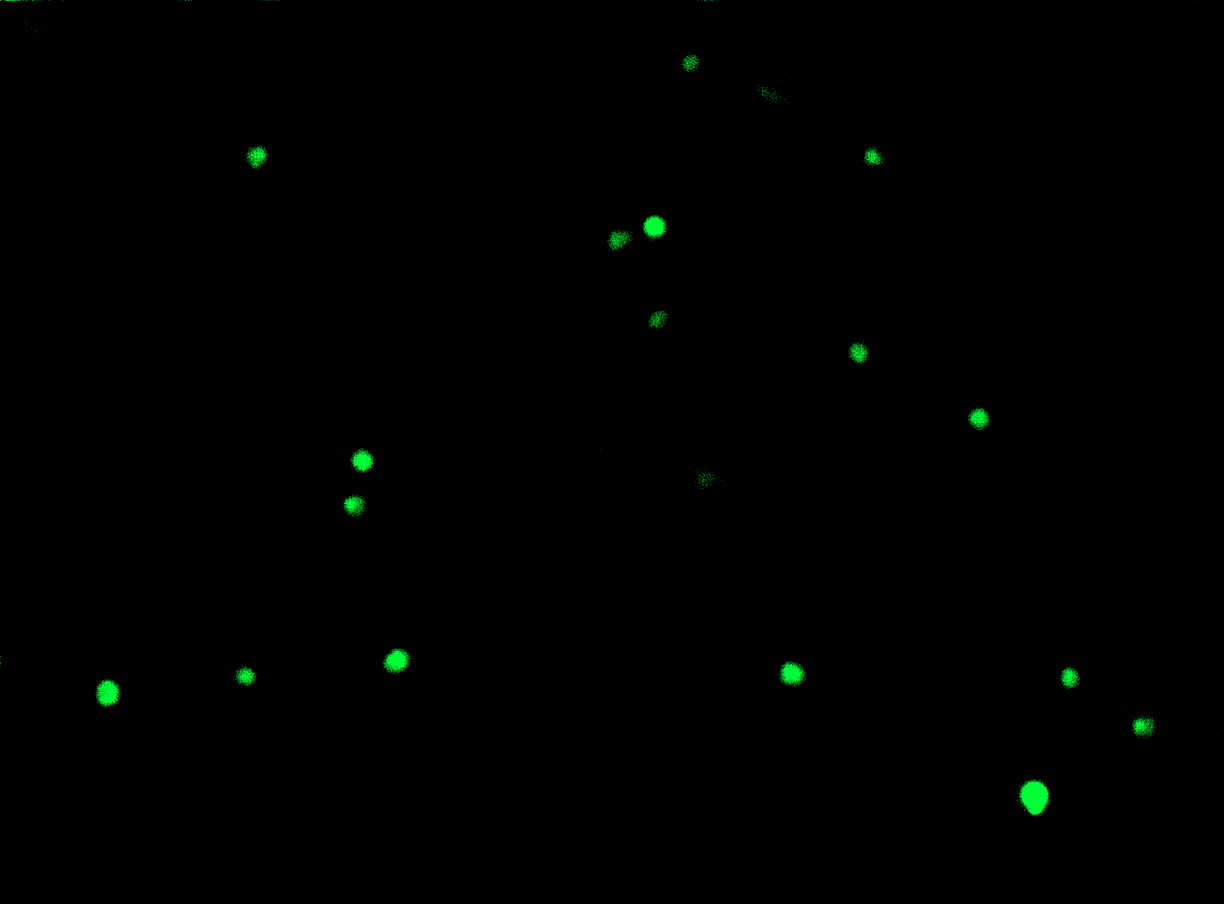

Supplement: Supplementary file 13 — Source Data for EV and Appendix figures [file 44321_2024_117_MOESM13_ESM.zip › Source Data for Expanded View and Appendix 5-23 f/Figure EV2/EV2C/tPA_H3-Cit.tif]
