# Supplementary figures and images for: Histidine-rich glycoprotein modulates neutrophils and thrombolysis-associated hemorrhagic transformation (part 2 of 2)
Source: EMBO Mol Med. 2024 Aug 15;16(9):10. doi: 10.1038/s44321-024-00117-y (PMC11393346; doi:10.1038/s44321-024-00117-y)

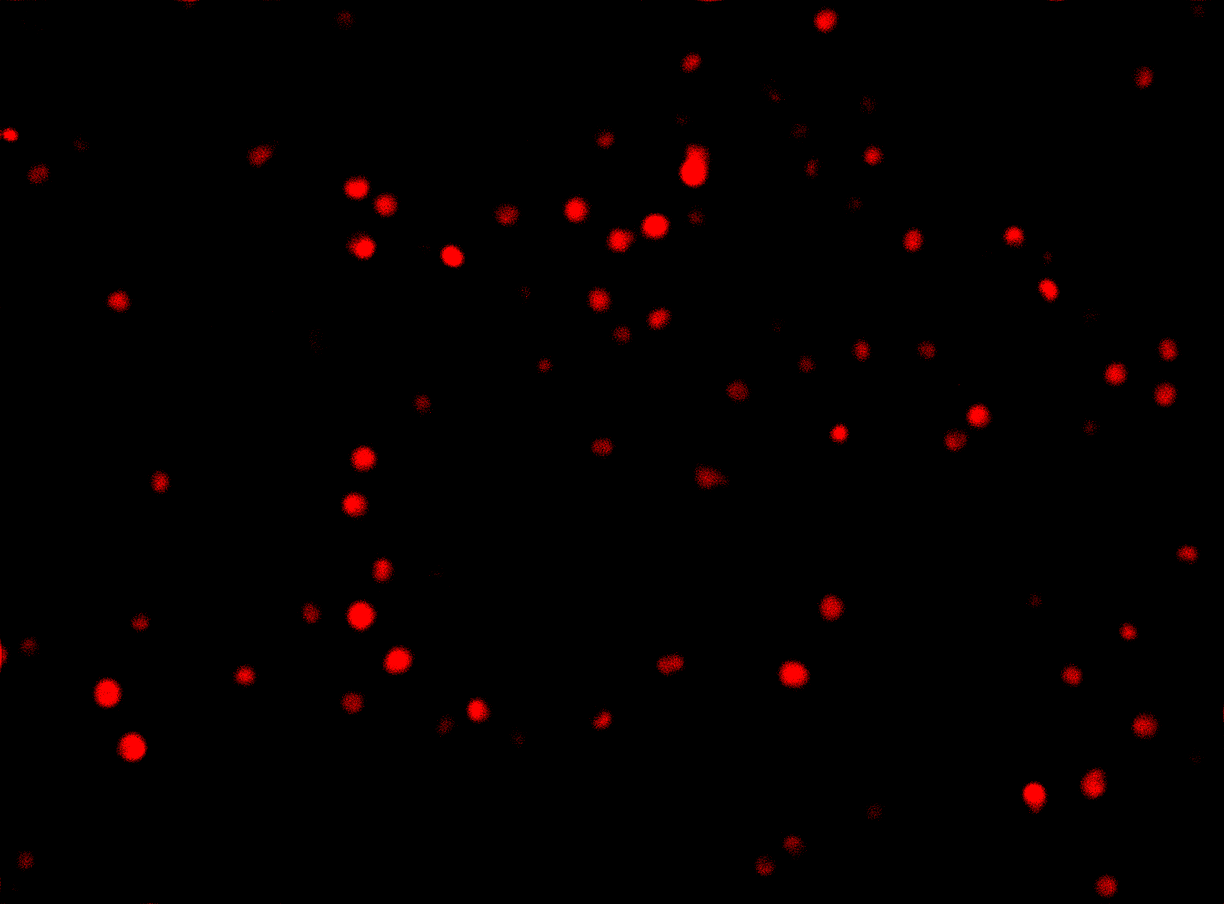

Supplement: Supplementary file 13 — Source Data for EV and Appendix figures [file 44321_2024_117_MOESM13_ESM.zip › Source Data for Expanded View and Appendix 5-23 f/Figure EV2/EV2C/tPA_MPO.tif]

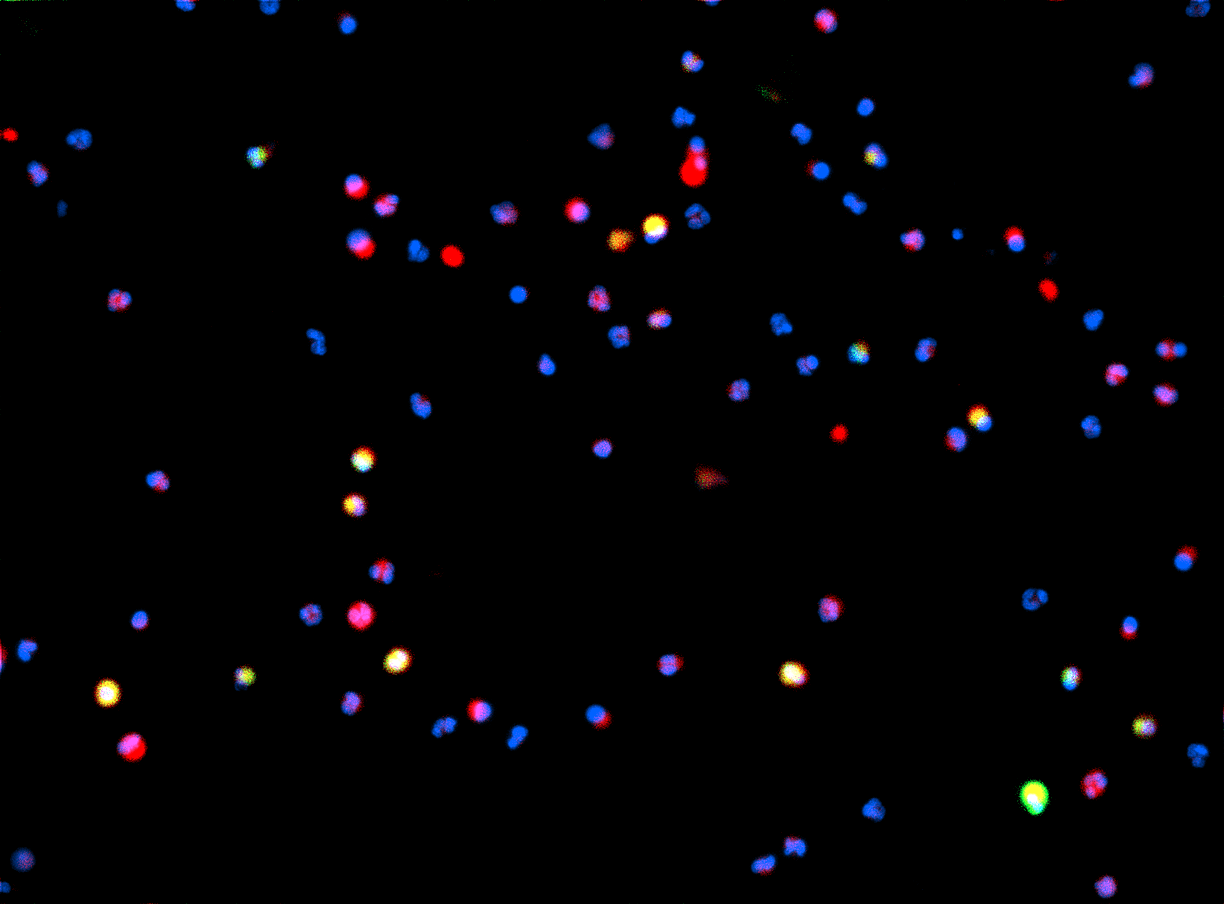

Supplement: Supplementary file 13 — Source Data for EV and Appendix figures [file 44321_2024_117_MOESM13_ESM.zip › Source Data for Expanded View and Appendix 5-23 f/Figure EV2/EV2C/tPA_Merge.tif]

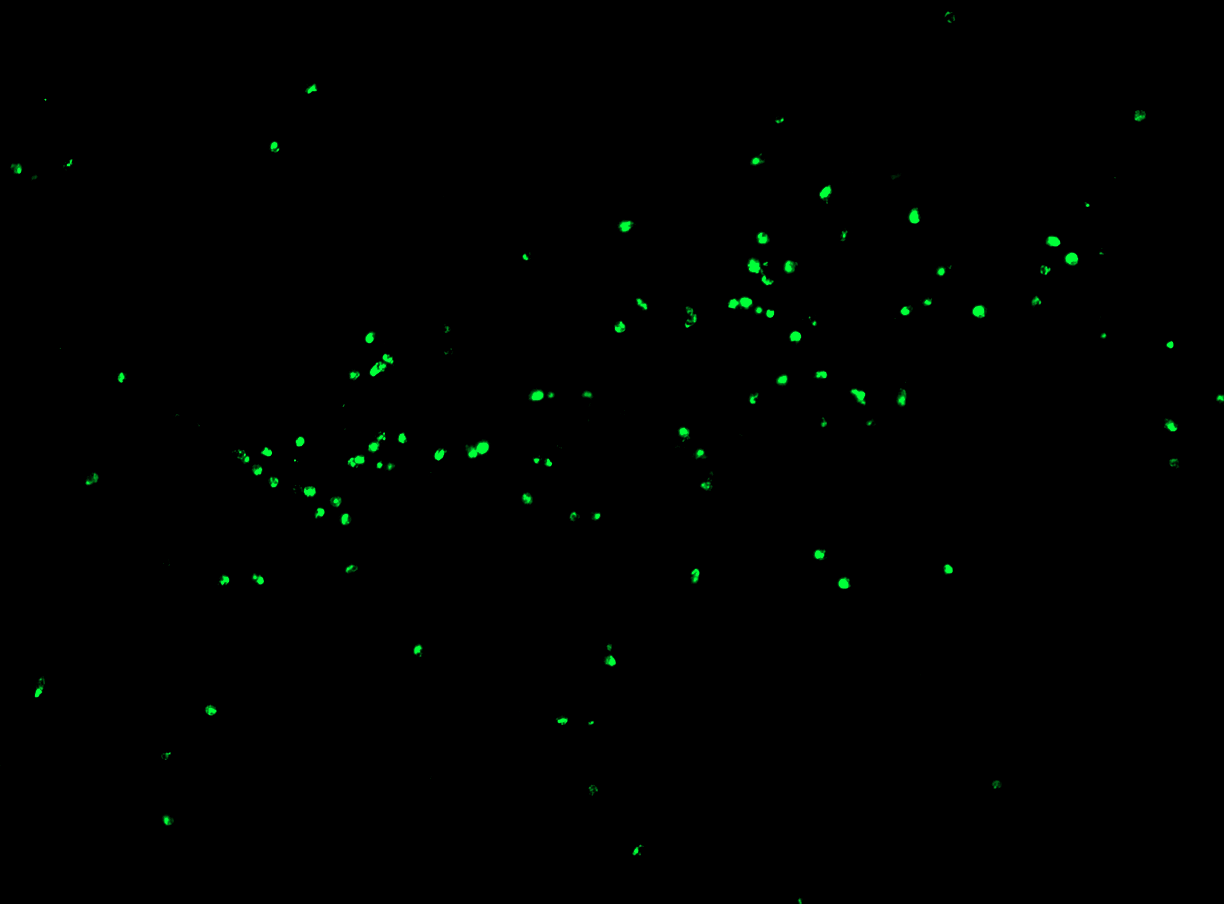

Supplement: Supplementary file 13 — Source Data for EV and Appendix figures [file 44321_2024_117_MOESM13_ESM.zip › Source Data for Expanded View and Appendix 5-23 f/Figure EV3/EV3A/5h tPA+HRG siRNA_Ly6G.tif]

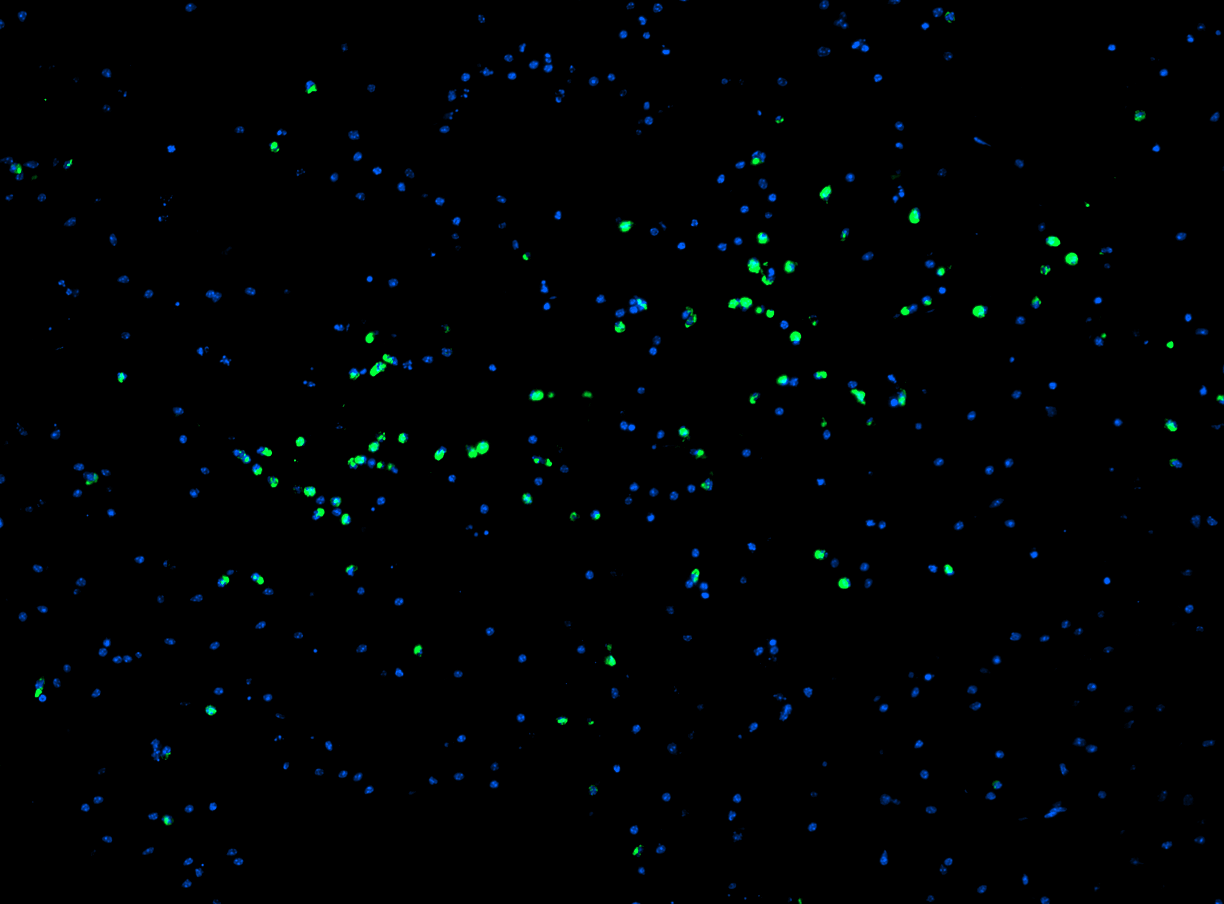

Supplement: Supplementary file 13 — Source Data for EV and Appendix figures [file 44321_2024_117_MOESM13_ESM.zip › Source Data for Expanded View and Appendix 5-23 f/Figure EV3/EV3A/5h tPA+HRG siRNA_Merge.tif]

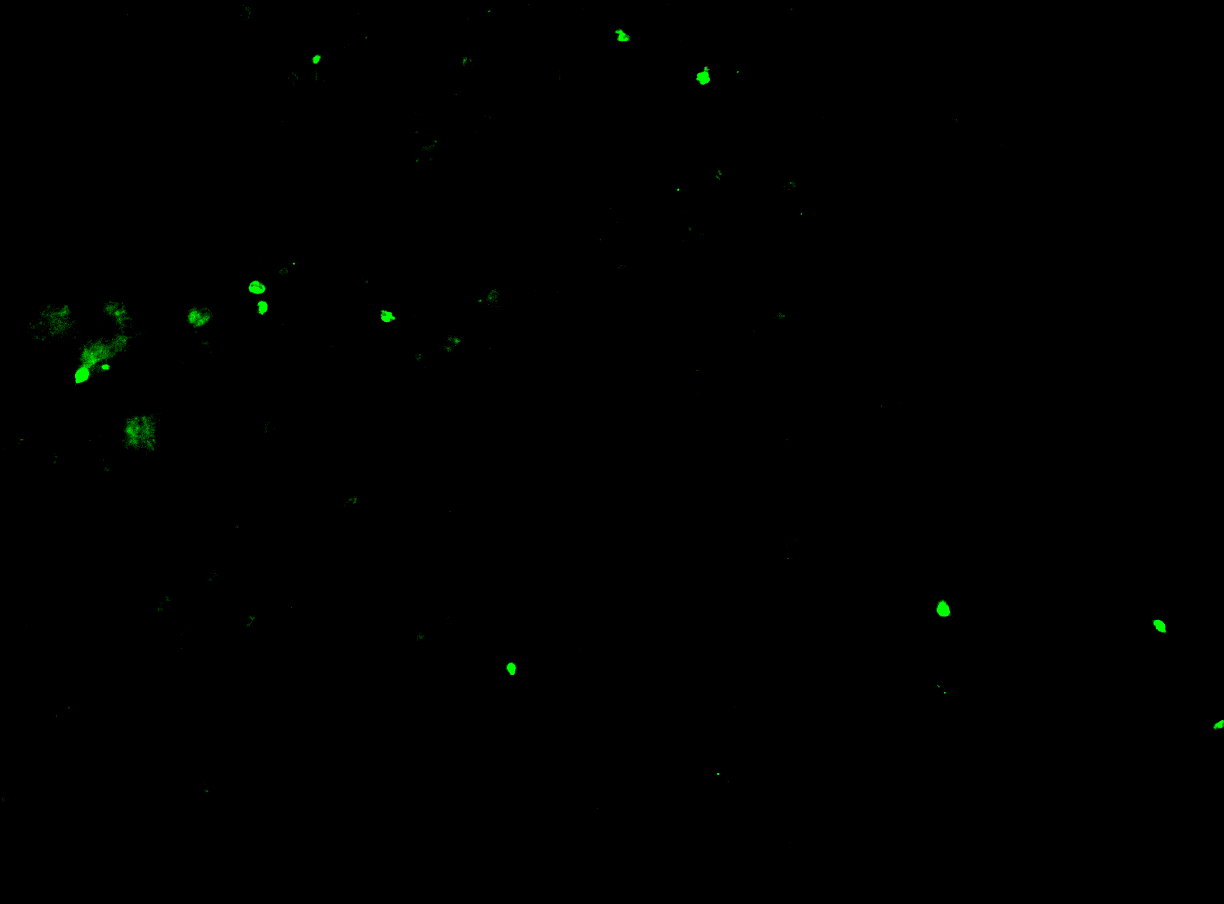

Supplement: Supplementary file 13 — Source Data for EV and Appendix figures [file 44321_2024_117_MOESM13_ESM.zip › Source Data for Expanded View and Appendix 5-23 f/Figure EV3/EV3A/5h tPA+HRG_Ly6G.tif]

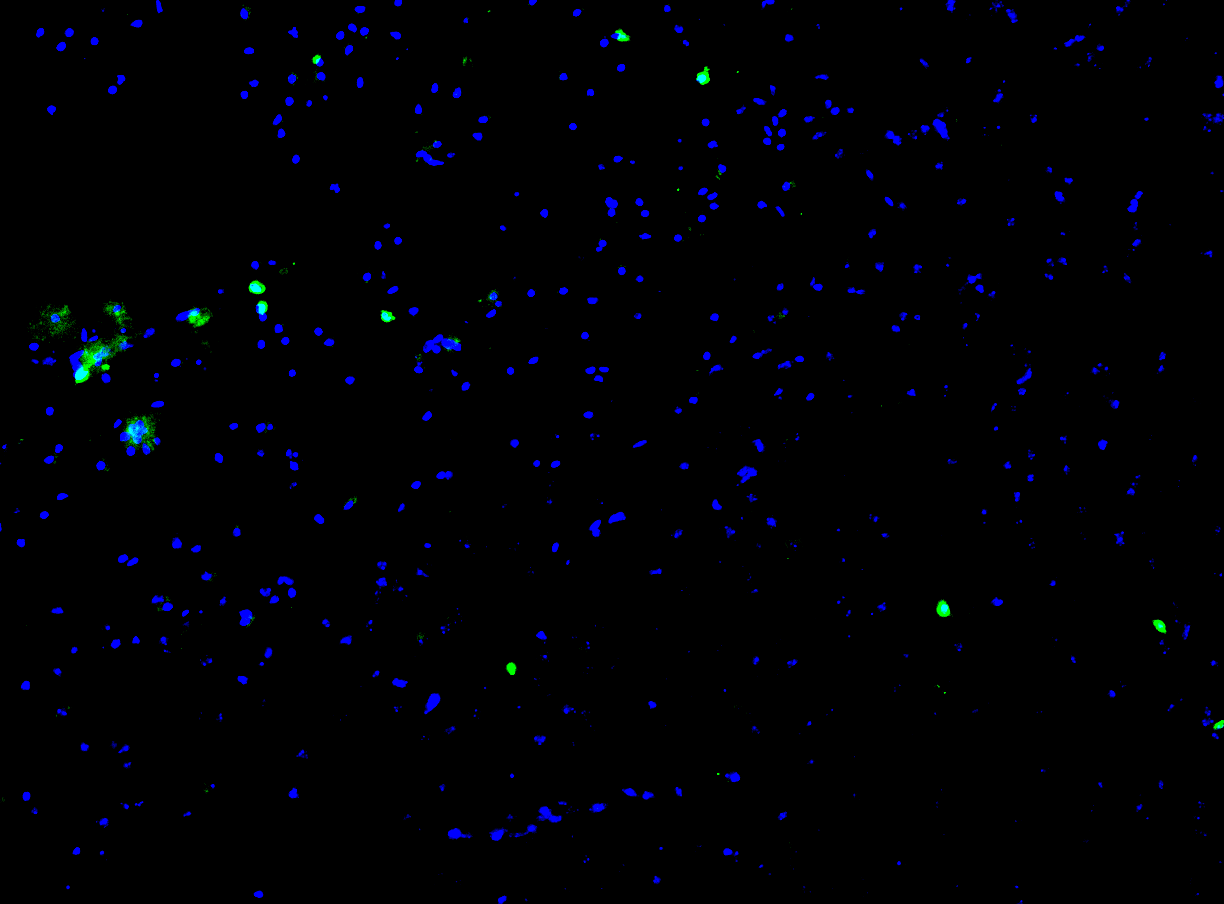

Supplement: Supplementary file 13 — Source Data for EV and Appendix figures [file 44321_2024_117_MOESM13_ESM.zip › Source Data for Expanded View and Appendix 5-23 f/Figure EV3/EV3A/5h tPA+HRG_Merge.tif]

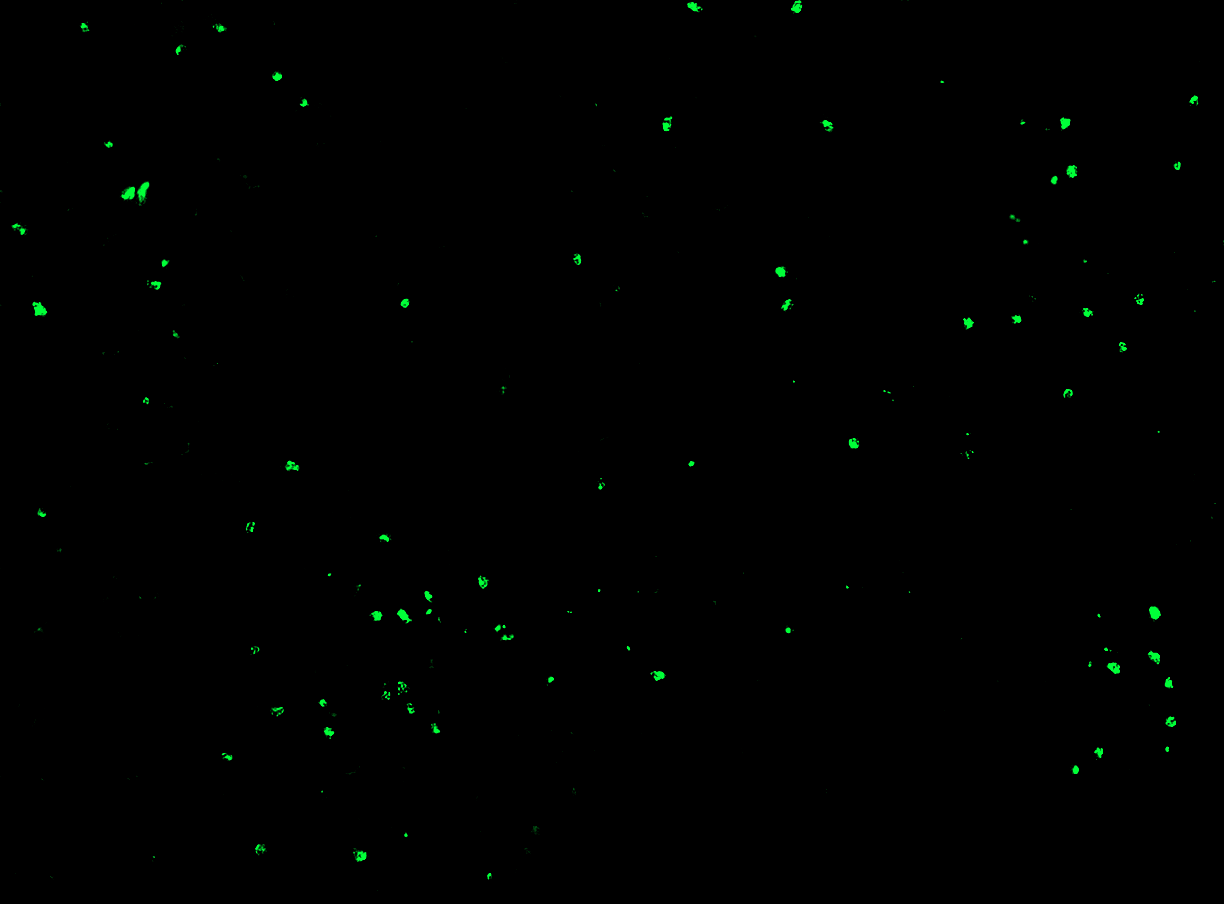

Supplement: Supplementary file 13 — Source Data for EV and Appendix figures [file 44321_2024_117_MOESM13_ESM.zip › Source Data for Expanded View and Appendix 5-23 f/Figure EV3/EV3A/5h tPA_Ly6G.tif]

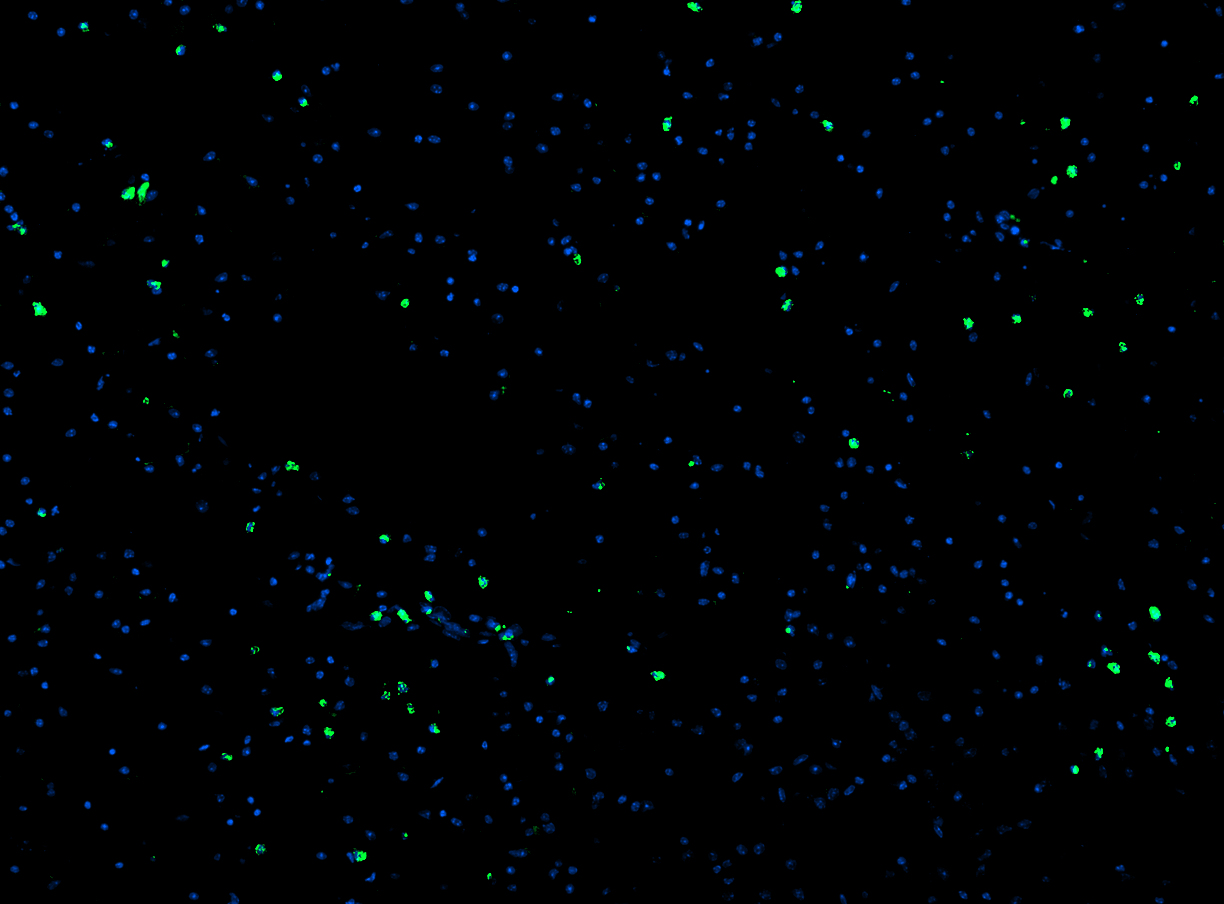

Supplement: Supplementary file 13 — Source Data for EV and Appendix figures [file 44321_2024_117_MOESM13_ESM.zip › Source Data for Expanded View and Appendix 5-23 f/Figure EV3/EV3A/5h tPA_Merge.tif]

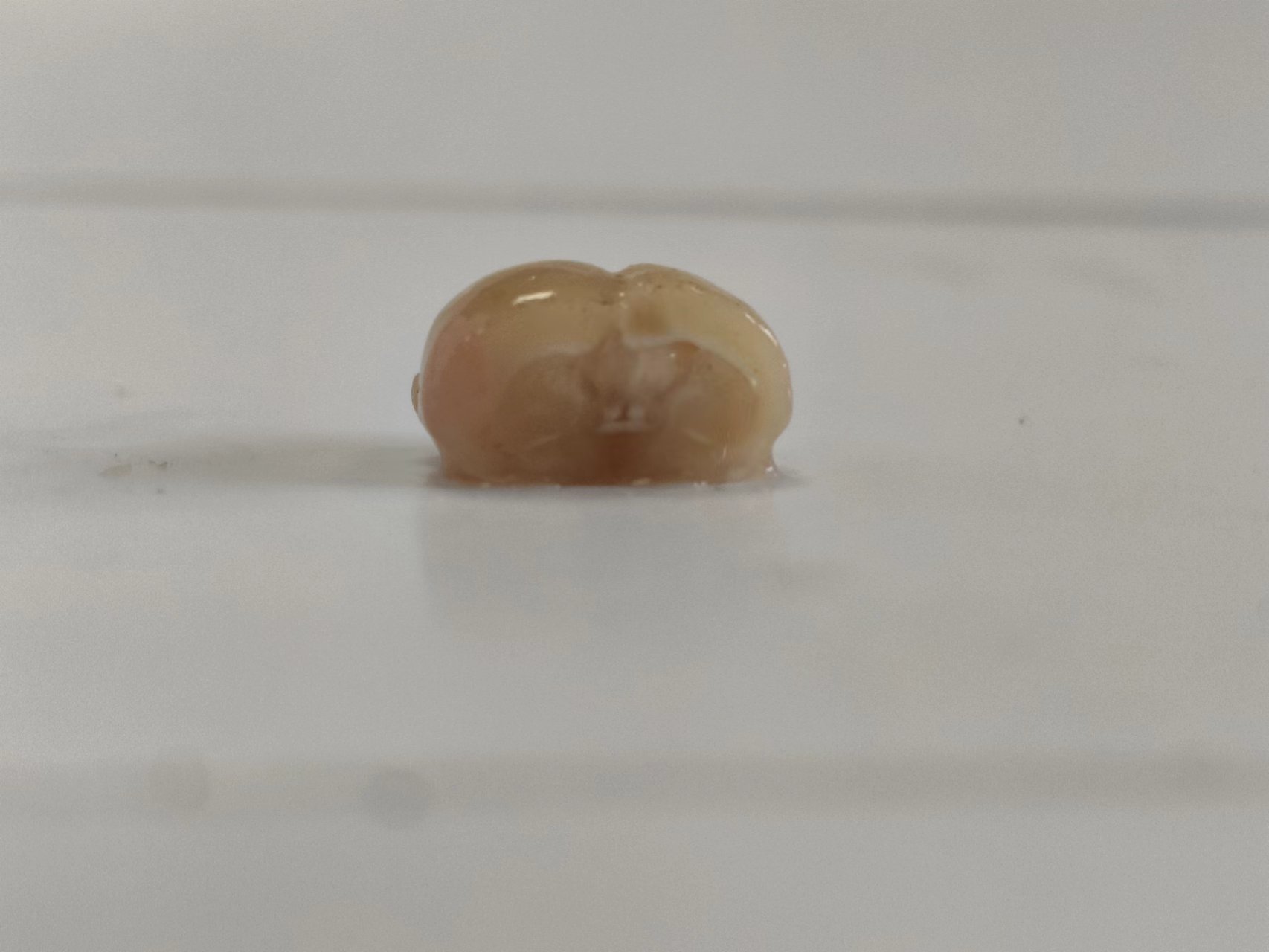

Supplement: Supplementary file 13 — Source Data for EV and Appendix figures [file 44321_2024_117_MOESM13_ESM.zip › Source Data for Expanded View and Appendix 5-23 f/Figure EV3/EV3F/5h tPA+HRG siRNA+Dnase I_coronal.jpg]

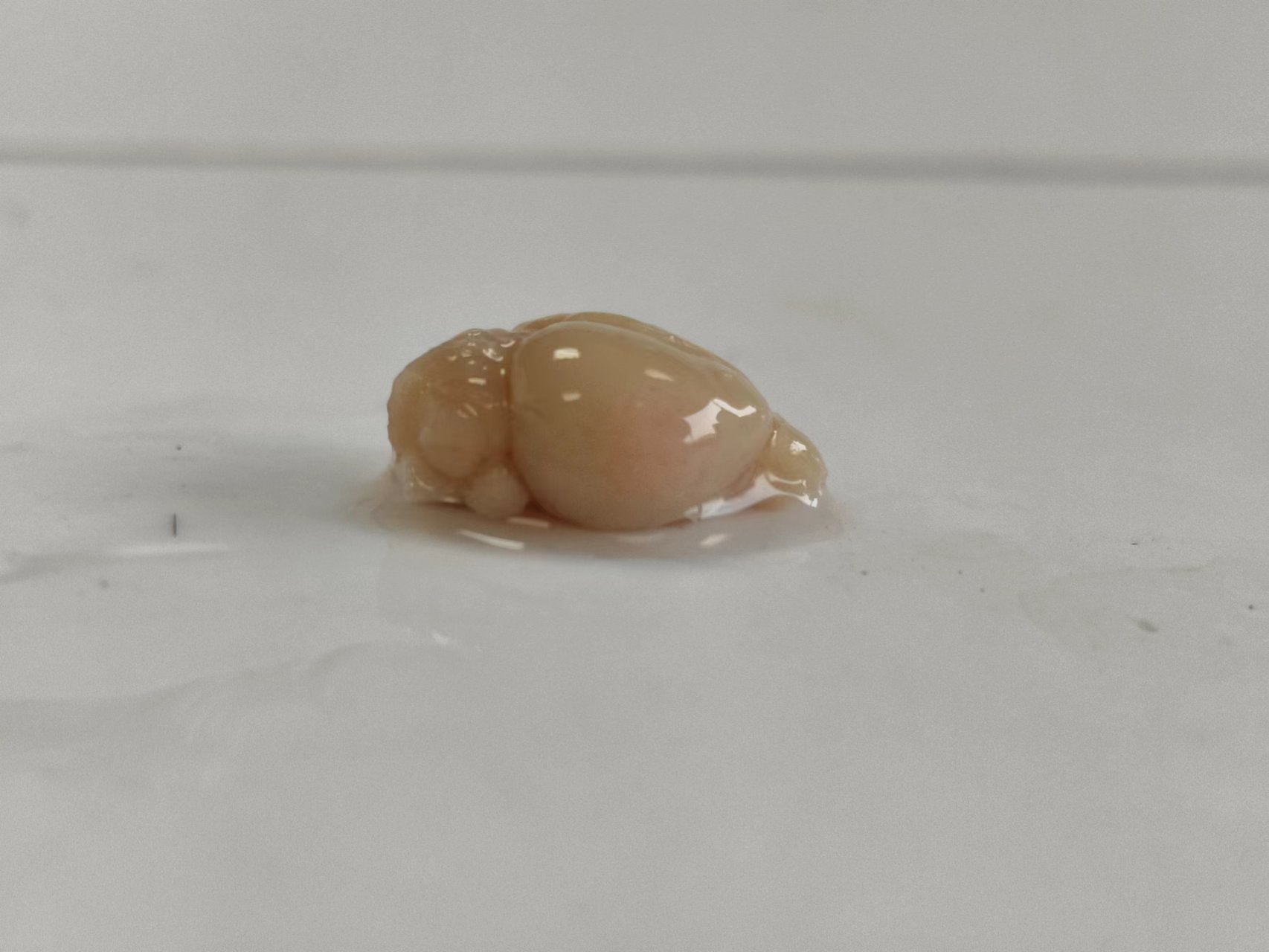

Supplement: Supplementary file 13 — Source Data for EV and Appendix figures [file 44321_2024_117_MOESM13_ESM.zip › Source Data for Expanded View and Appendix 5-23 f/Figure EV3/EV3F/5h tPA+HRG siRNA+Dnase I_dorsal.jpg]

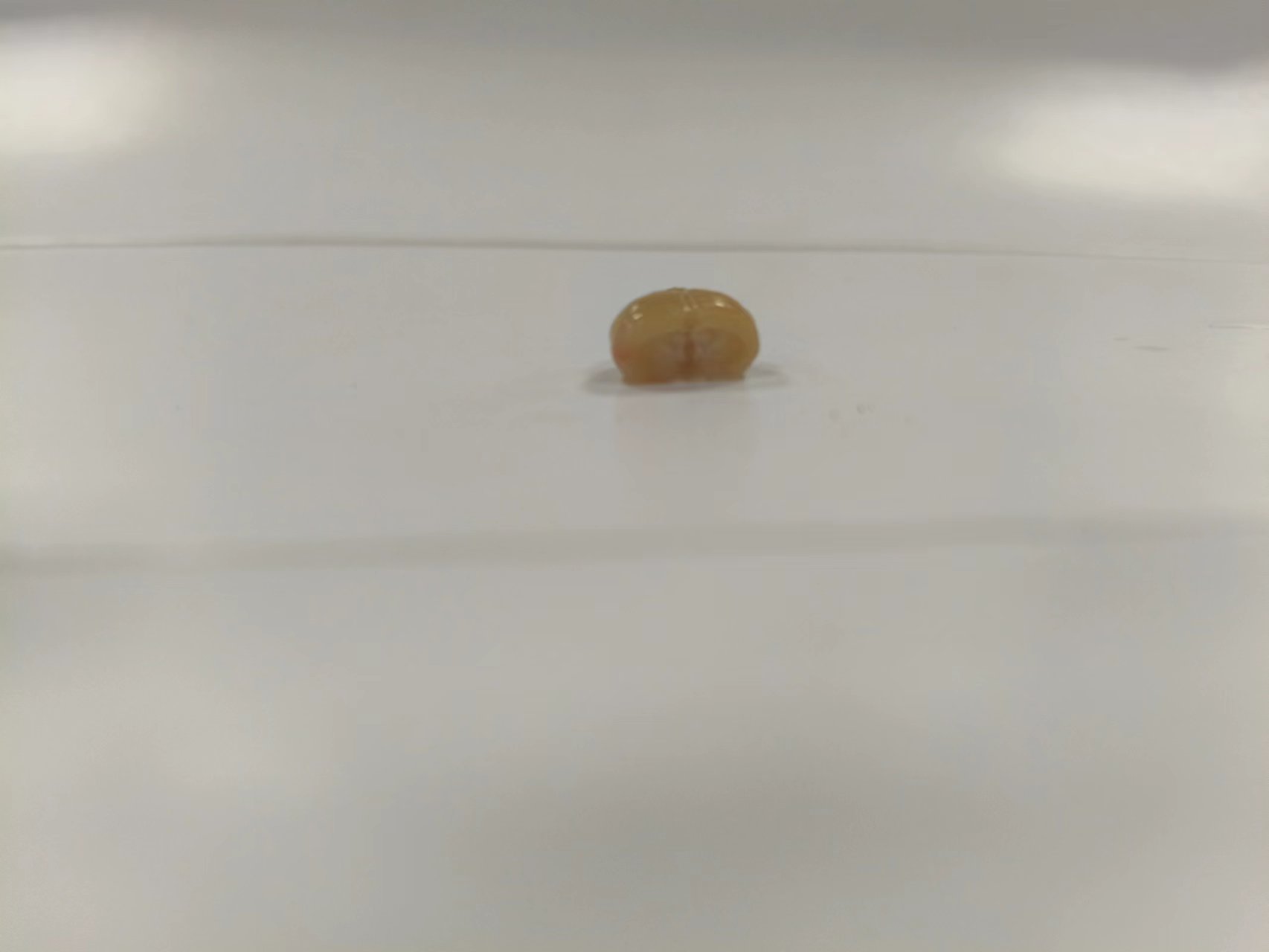

Supplement: Supplementary file 13 — Source Data for EV and Appendix figures [file 44321_2024_117_MOESM13_ESM.zip › Source Data for Expanded View and Appendix 5-23 f/Figure EV3/EV3F/5h tPA+HRG siRNA+anti-Ly6G_coronal.jpg]

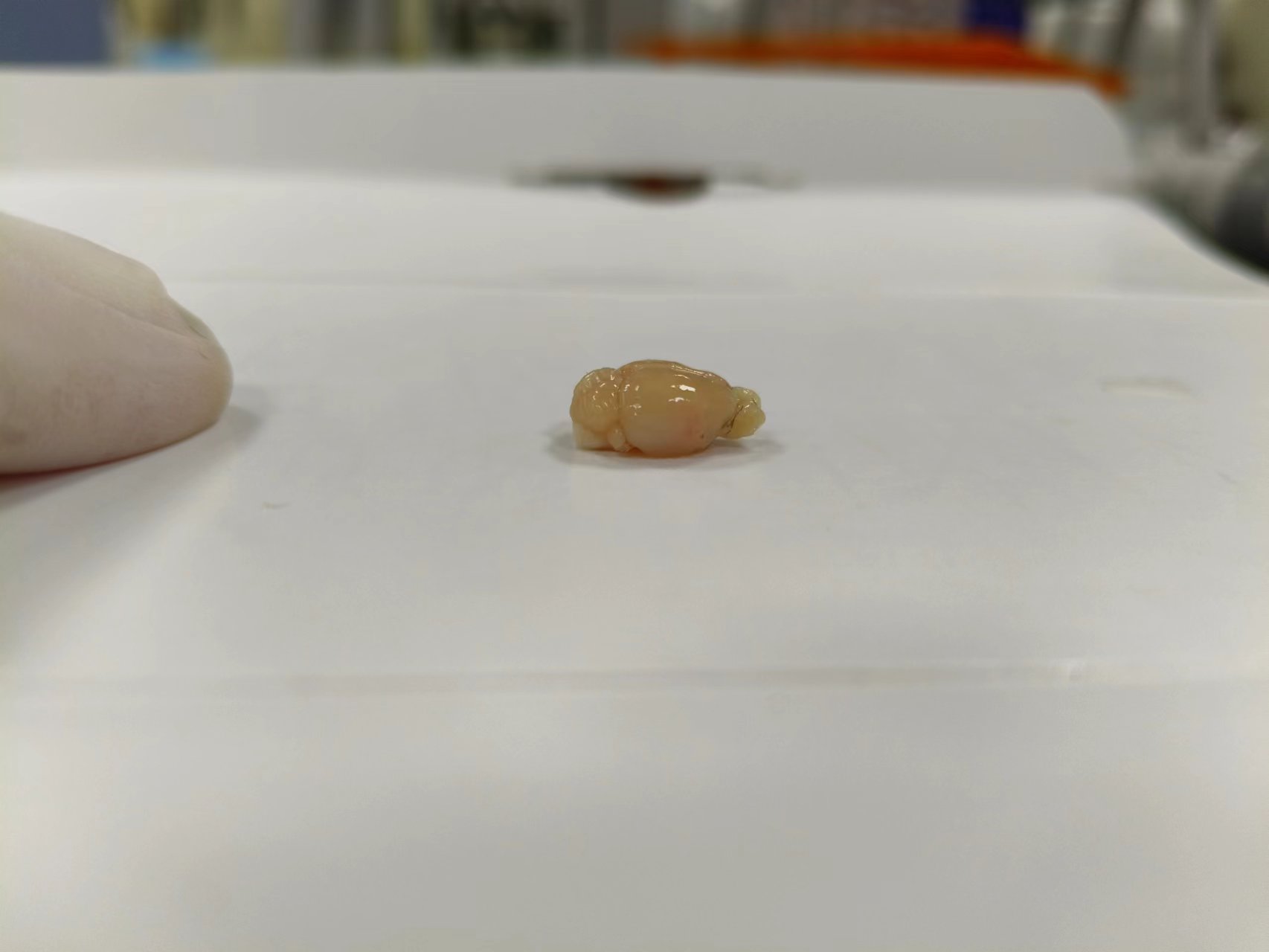

Supplement: Supplementary file 13 — Source Data for EV and Appendix figures [file 44321_2024_117_MOESM13_ESM.zip › Source Data for Expanded View and Appendix 5-23 f/Figure EV3/EV3F/5h tPA+HRG siRNA+anti-Ly6G_dorsal.jpg]

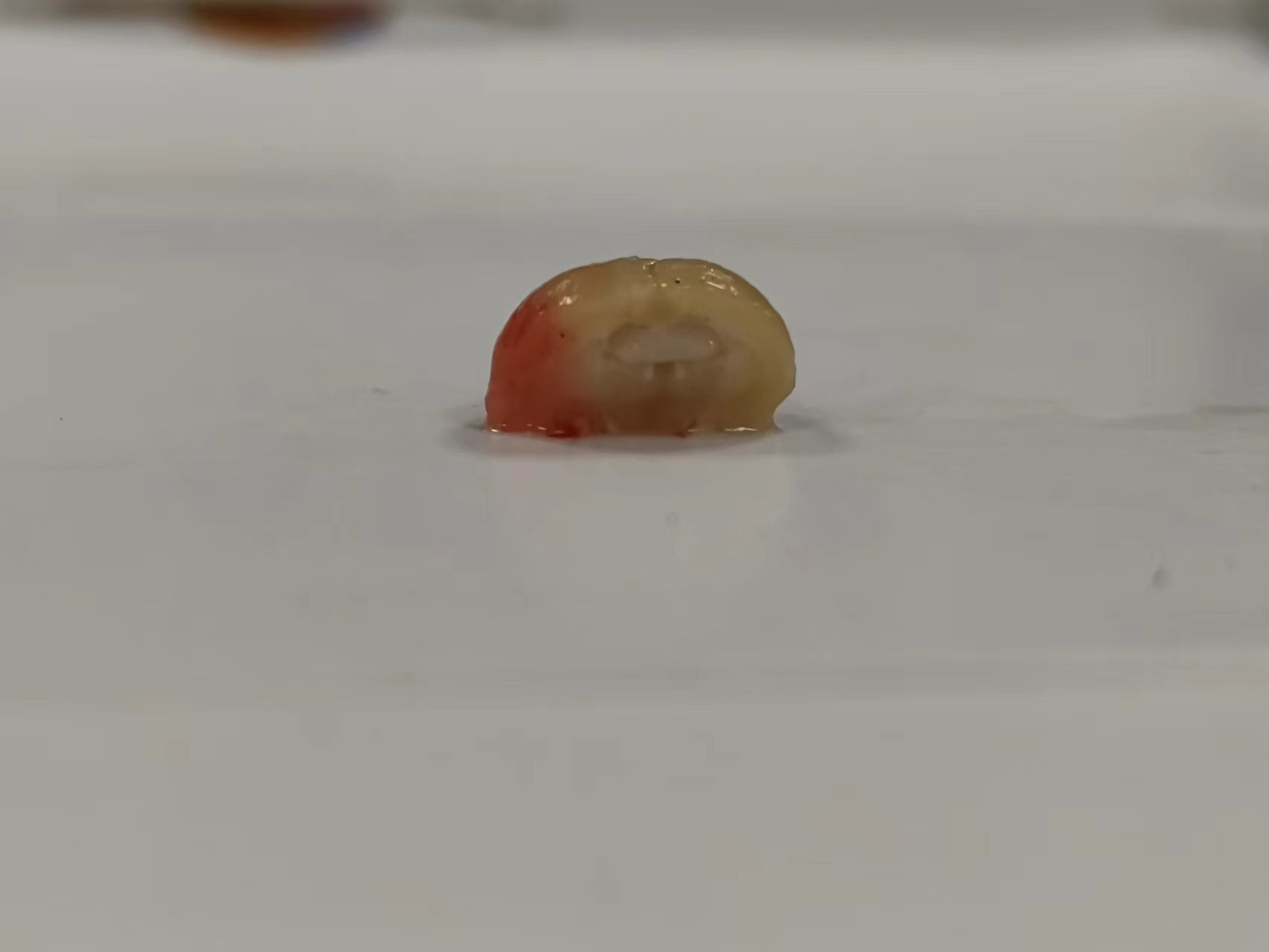

Supplement: Supplementary file 13 — Source Data for EV and Appendix figures [file 44321_2024_117_MOESM13_ESM.zip › Source Data for Expanded View and Appendix 5-23 f/Figure EV3/EV3F/5h tPA+HRG siRNA_coronal.jpg]

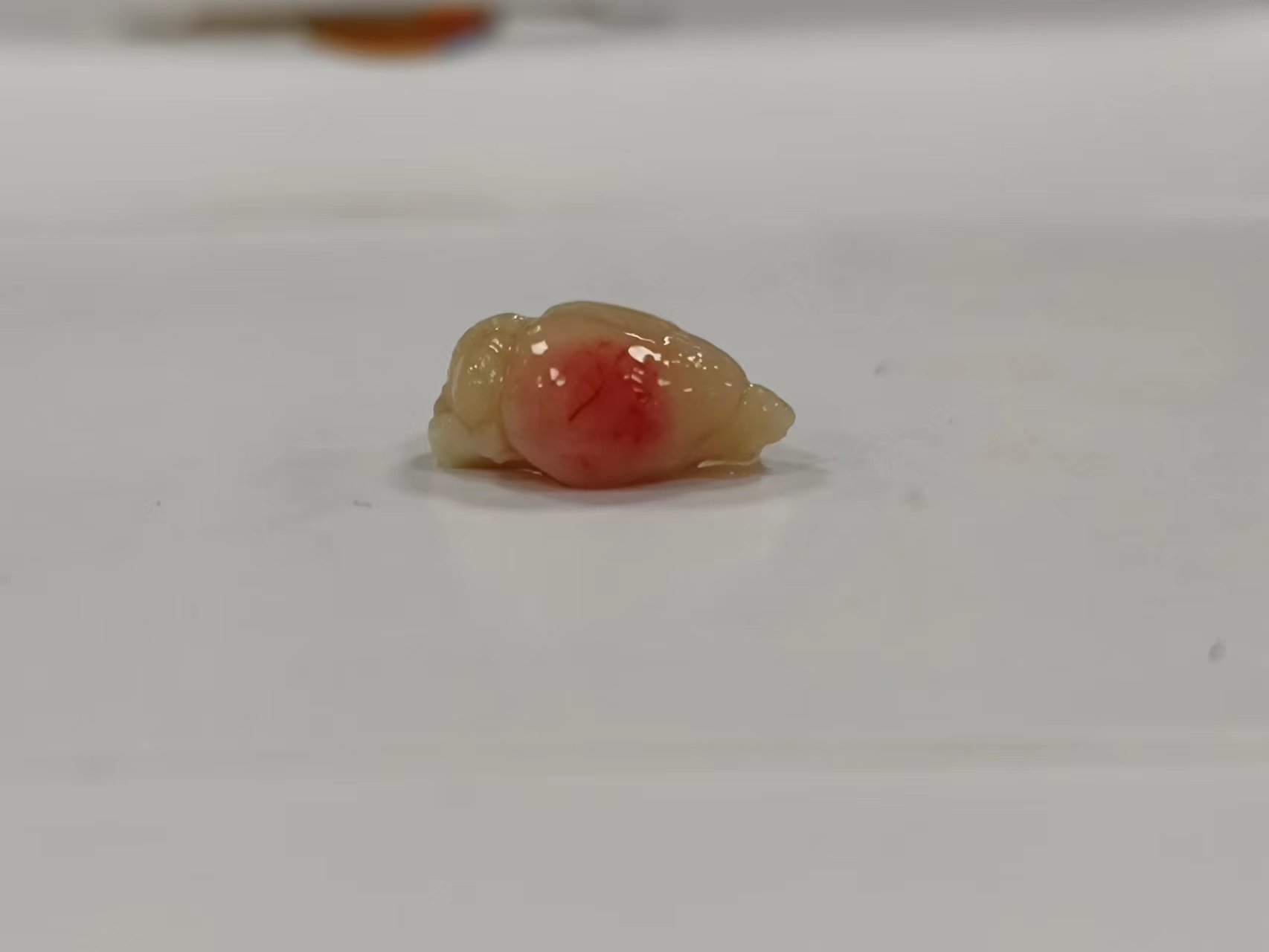

Supplement: Supplementary file 13 — Source Data for EV and Appendix figures [file 44321_2024_117_MOESM13_ESM.zip › Source Data for Expanded View and Appendix 5-23 f/Figure EV3/EV3F/5h tPA+HRG siRNA_dorsal.jpg]

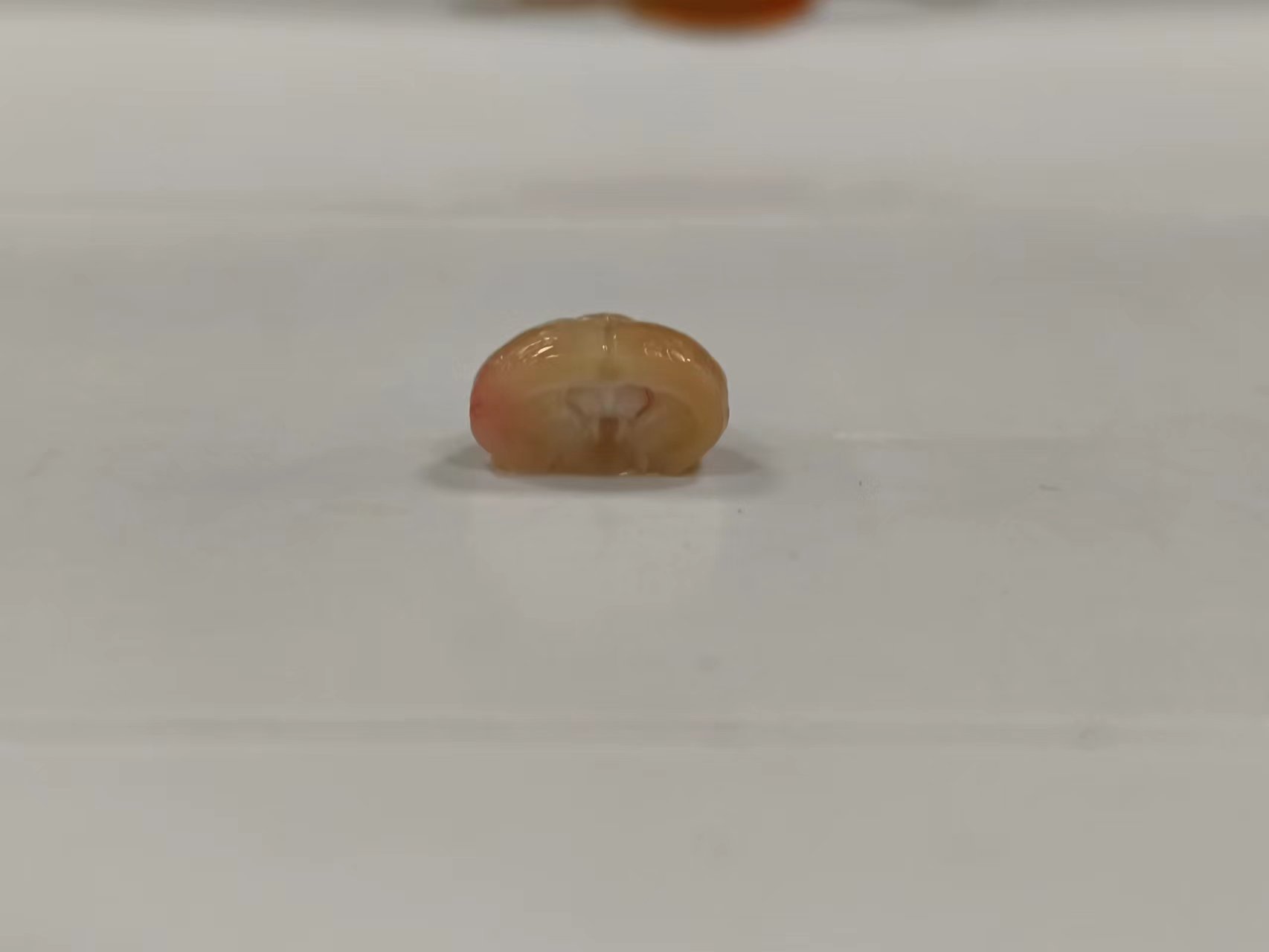

Supplement: Supplementary file 13 — Source Data for EV and Appendix figures [file 44321_2024_117_MOESM13_ESM.zip › Source Data for Expanded View and Appendix 5-23 f/Figure EV3/EV3F/5h tPA+HRG_coronal.jpg]

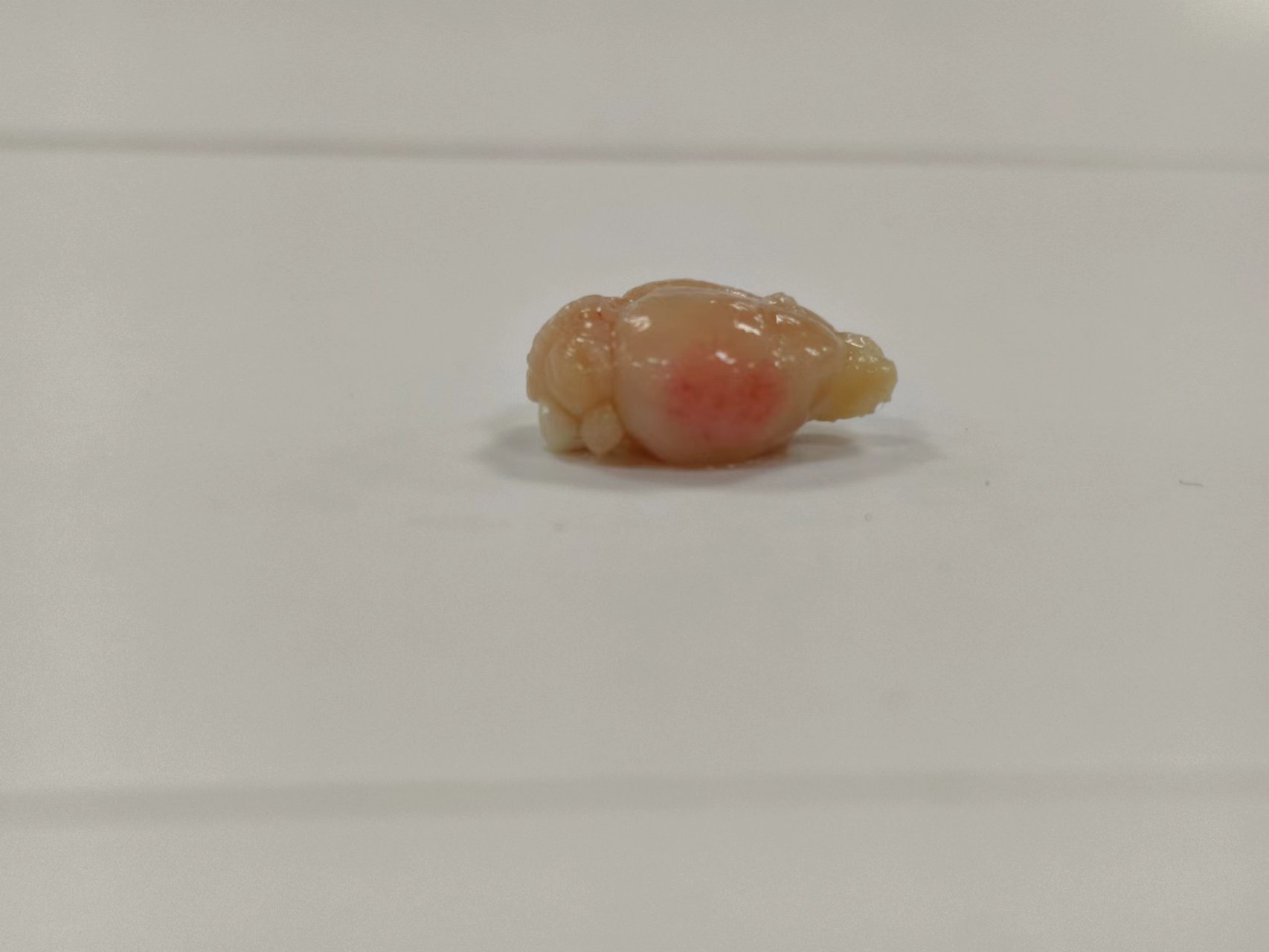

Supplement: Supplementary file 13 — Source Data for EV and Appendix figures [file 44321_2024_117_MOESM13_ESM.zip › Source Data for Expanded View and Appendix 5-23 f/Figure EV3/EV3F/5h tPA+HRG_dorsal.jpg]

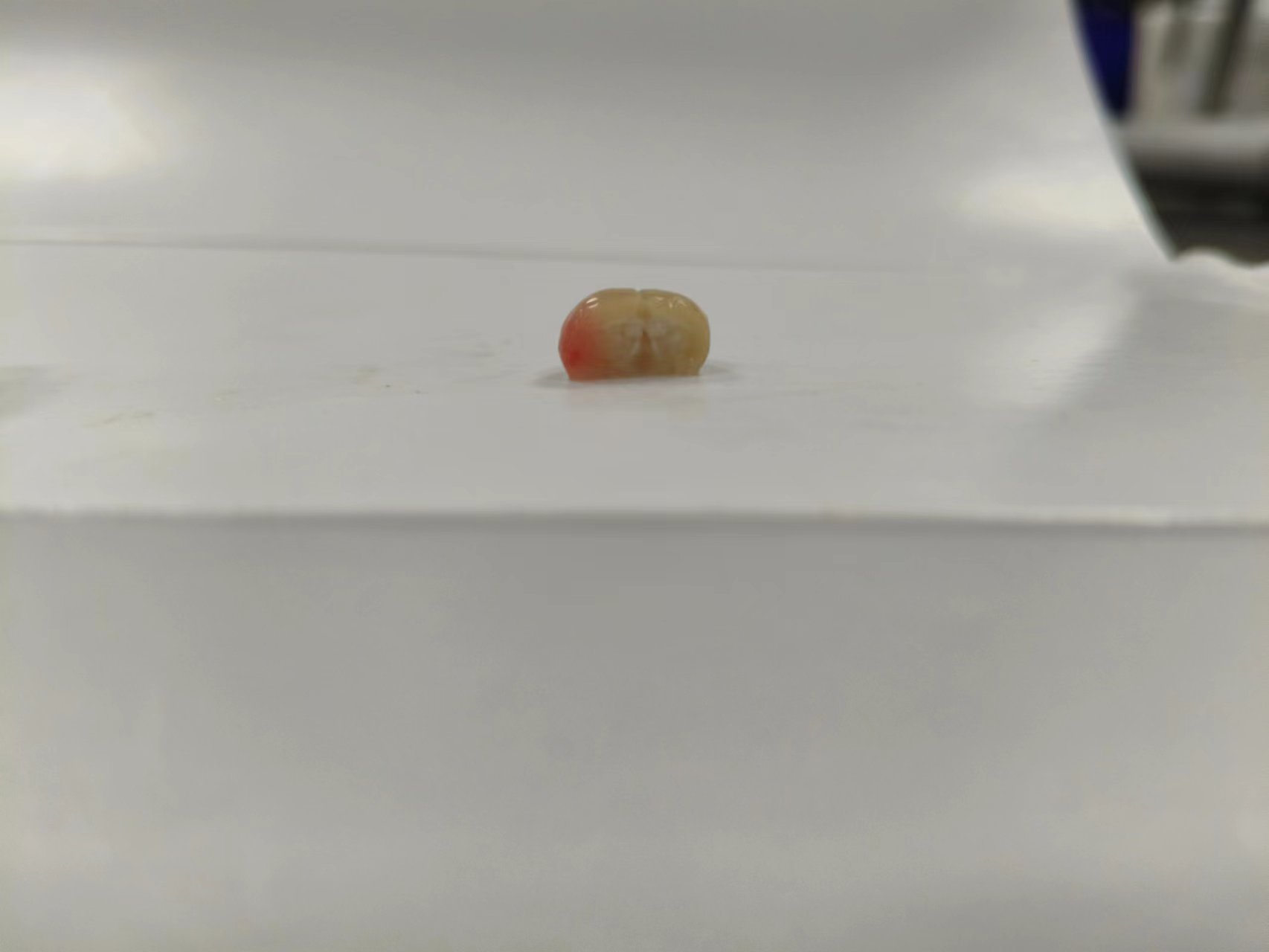

Supplement: Supplementary file 13 — Source Data for EV and Appendix figures [file 44321_2024_117_MOESM13_ESM.zip › Source Data for Expanded View and Appendix 5-23 f/Figure EV3/EV3F/5h tPA_coronal.jpg]

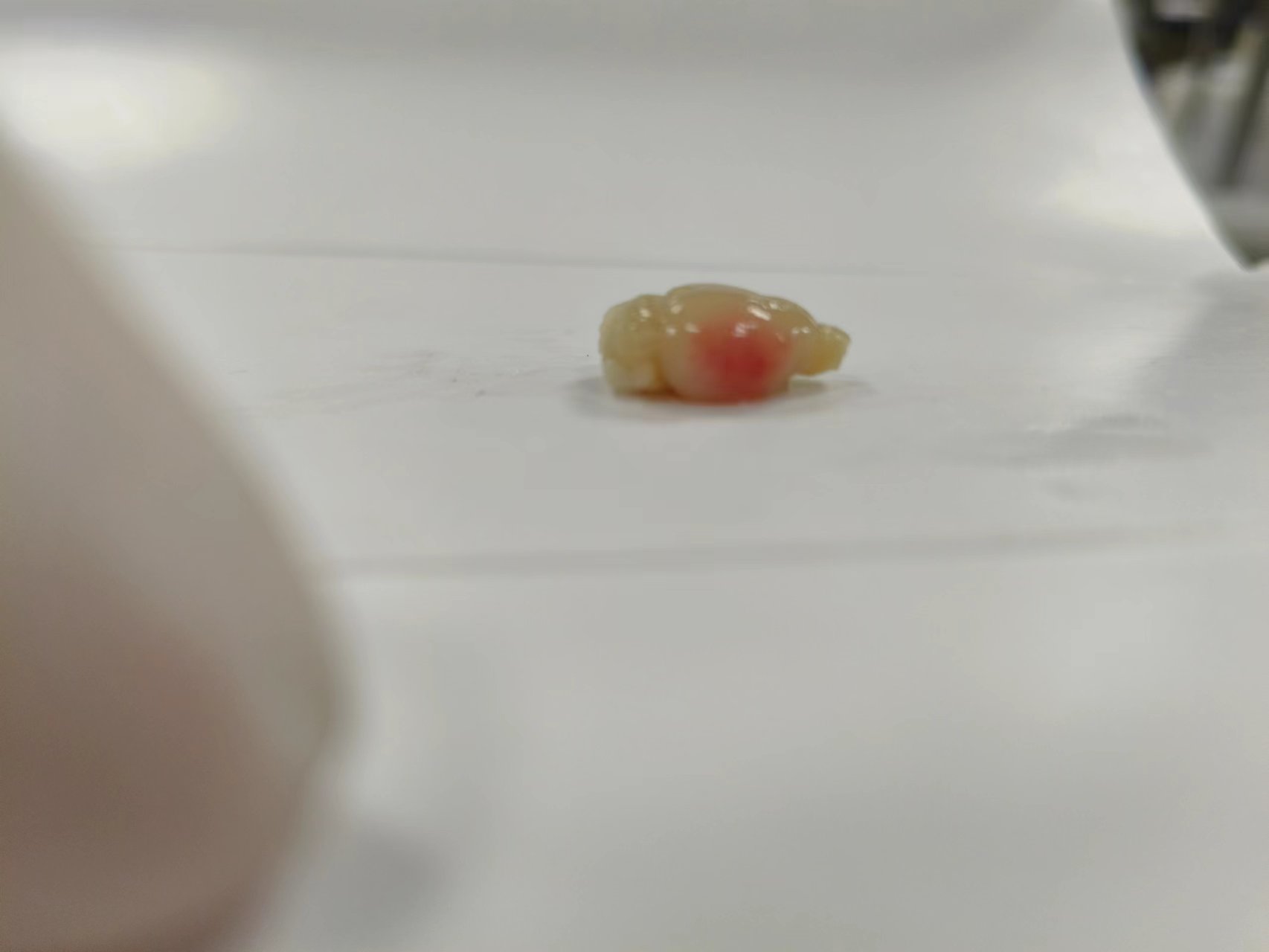

Supplement: Supplementary file 13 — Source Data for EV and Appendix figures [file 44321_2024_117_MOESM13_ESM.zip › Source Data for Expanded View and Appendix 5-23 f/Figure EV3/EV3F/5h tPA_dorsal.jpg]
