# Supplementary material for: Histidine-rich glycoprotein modulates neutrophils and thrombolysis-associated hemorrhagic transformation
Source: EMBO Mol Med. 2024 Aug 15;16(9):10. doi: 10.1038/s44321-024-00117-y (PMC11393346; doi:10.1038/s44321-024-00117-y)
Supplement: Supplementary file 14 — Expanded View Figures [file 44321_2024_117_MOESM14_ESM.pdf]

Expanded View Figures

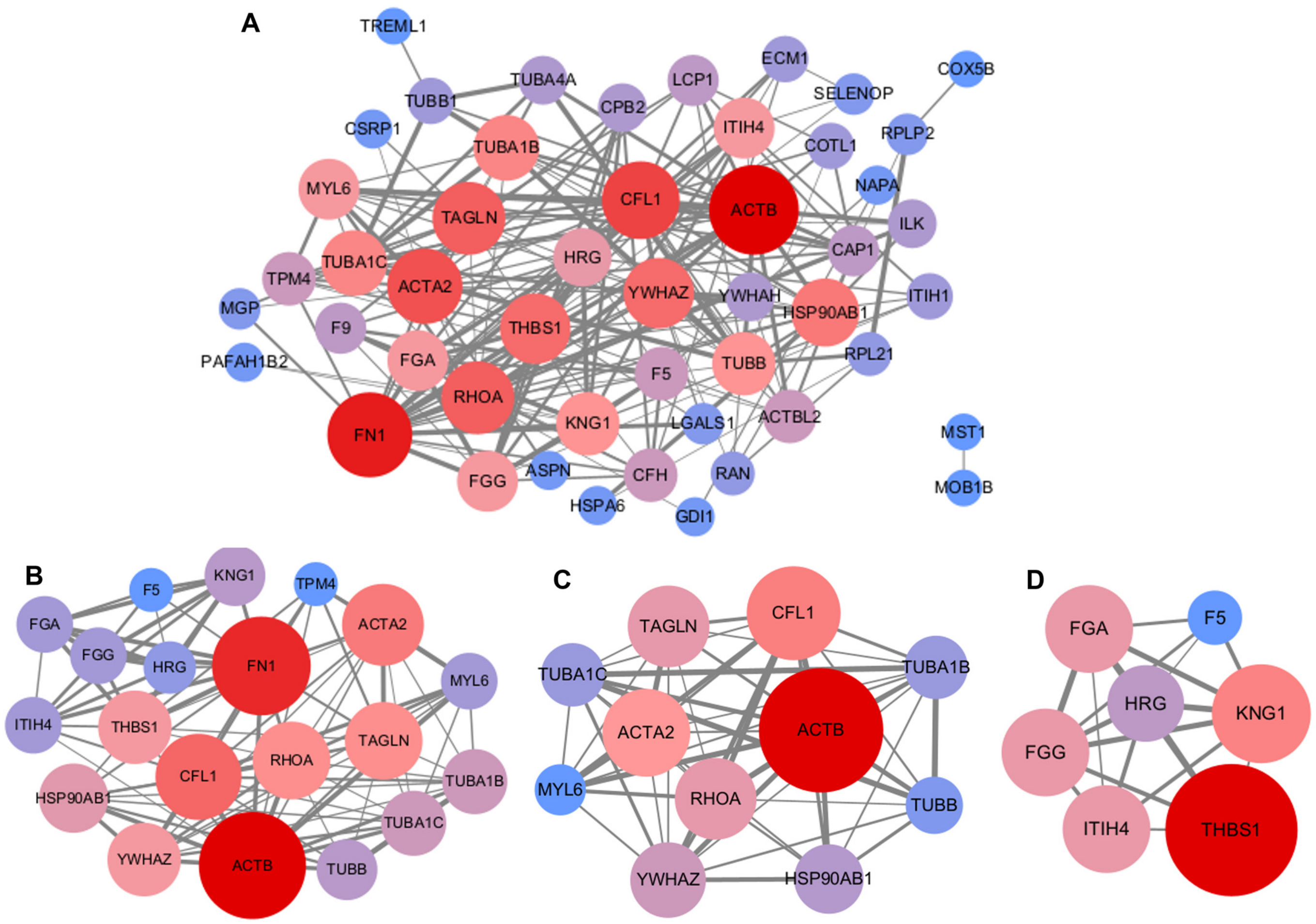

Figure EV1. Protein-protein interaction (PPI) network analysis of differentially expressed proteins (DEPs).

(**A**) PPI network of plasma DEPs between individuals after tPA vs. before and after tPA treatment. The PPI numbers of the DEPs are indicated by node size and color. Edge thickness indicates the degree of interaction between DEPs. (**B**) Top 20 hub genes identified using the CytoHubba plugin. (**C**, **D**) The top two significant cluster networks identified using the MCODE plugin.

 © The Author(s)

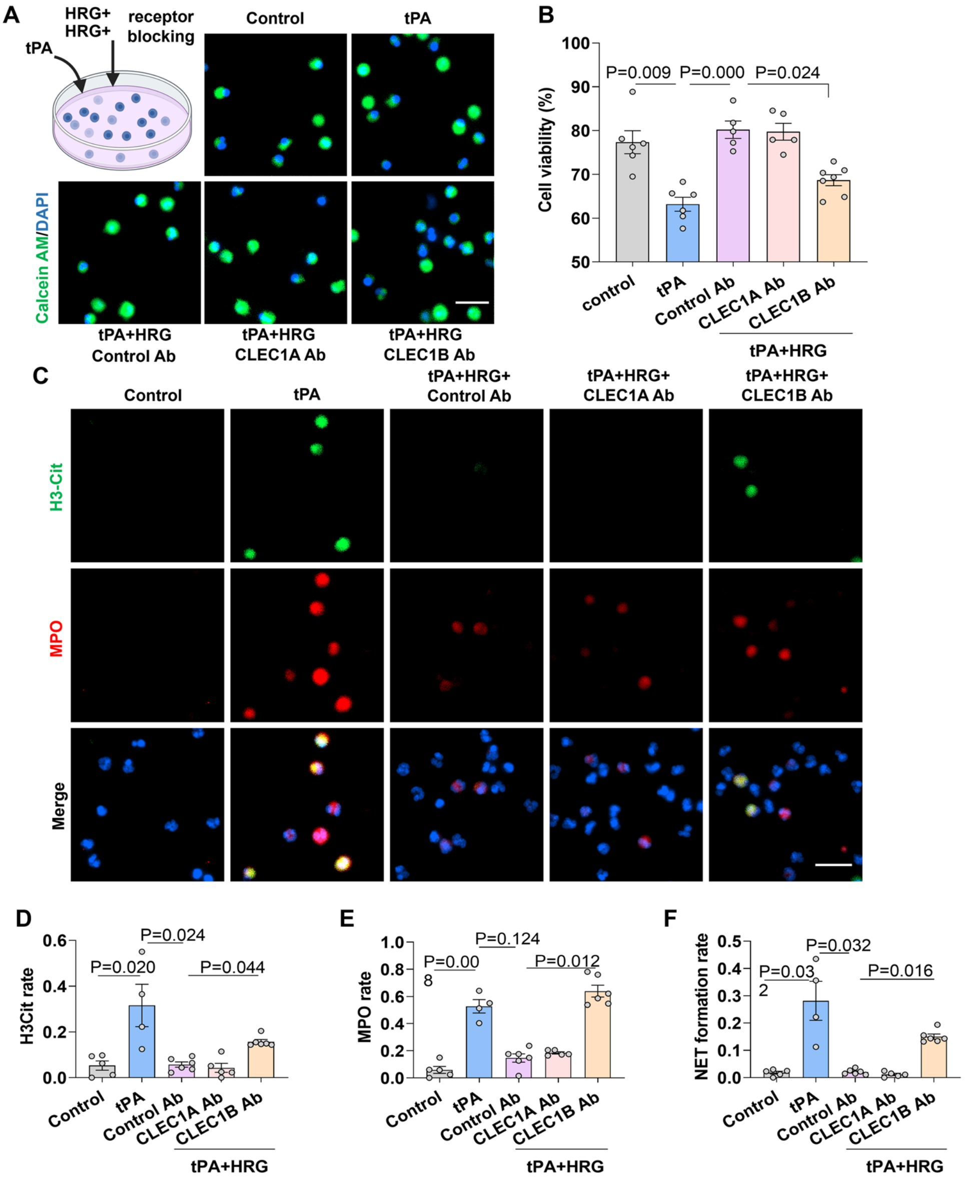

Figure EV2. Effect of CLEC1A and CLEC1B blockade on the mortality rate and NETosis of neutrophils in response to tPA and HRG treatment in vitro.

(**A**) Neutrophils were labeled with calcein-AM (living cell, green) and Hoechst 33342 (nuclei, blue). Neutrophils were cultured under the following conditions: vehicle, tPA, tPA + HRG + Control Ab, tPA + HRG + blocking antibodies against CLEC1A and CLEC1B. After 18 h of culture, the viability of neutrophils was observed and calculated under a fluorescent microscope. Typical results from five independent experiments are shown. Scale bar = 20 μm. (**B**) Statistical analysis of the cell viability is shown (n = 5–7). (**C**) Neutrophils were incubated for 2.5 h and co-stained with H3Cit (green), MPO (red), and DAPI (blue). Merged images indicate NET formation. Scale bar = 20 μm. (**D–F**) Statistical analysis and comparison of H3Cit, MPO, and NET formation rates in different experimental groups (n = 4–6). Data information: Data are presented as mean ± SEM. Two-tailed Kruskal–Wallis H test is used in (**B**, **D–F**). Source data are available online for this figure.

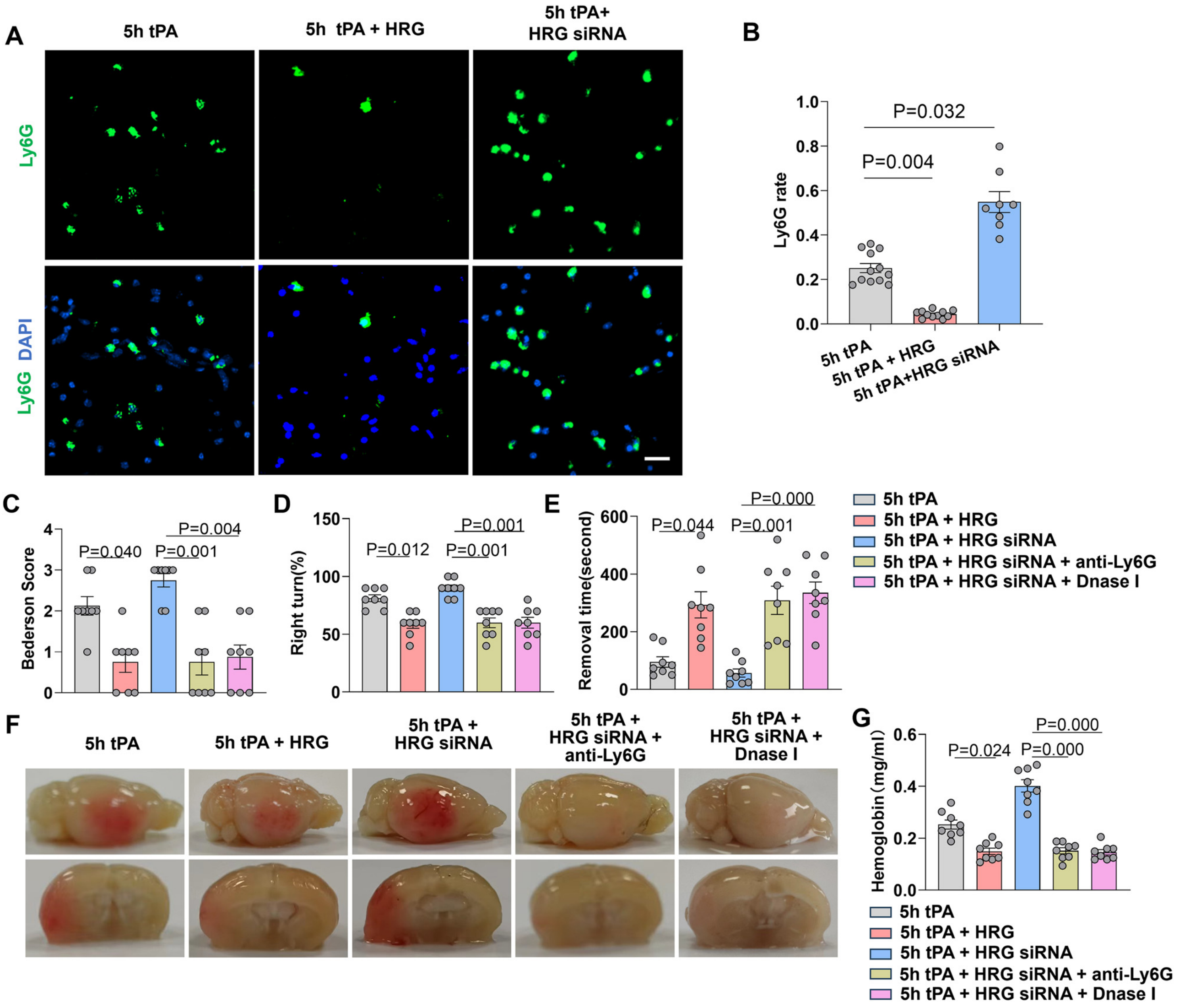

Figure EV3. Endogenous HRG depletion exacerbates hemorrhagic transformation and neurological deficits, which are rescued by neutrophil depletion or NETosis inhibition.

(**A**) Representative images of immunofluorescence staining of infiltrating neutrophils (Ly6G, green) in brain lesions. Scale bar = 20 μm. (**B**) The statistical analysis of infiltrating neutrophils (Ly6G rate) from the following experimental groups: tPA (5 h) ($n = 12$), tPA (5 h) + HRG ($n = 11$), and tPA (5 h) + HRG siRNA groups ($n = 8$). The data are also partially used in Fig. 6H. (**C–E**) Neurological deficits were measured by assessing the Bederson score (**C**), corner test (**D**) and adhesive removal test (**E**) in mice treated with tPA (5 h), tPA (5 h) + HRG, tPA (5 h) + HRG siRNA, tPA (5 h) + HRG siRNA + anti-Ly6G, and tPA (5 h) + HRG siRNA + Dnase I ($n = 8$ per group). (**F**) Representative images of the dorsal surface and a coronal section showing cerebral hemorrhage 24 h after stroke. (**G**) Quantification of cerebral hemorrhage by spectrophotometric hemoglobin assay ($n = 8$). Data information: Data are presented as mean ± SEM. Two-tailed Kruskal–Wallis H test is used in (**B–E**, **G**). Source data are available online for this figure.

 © The Author(s)

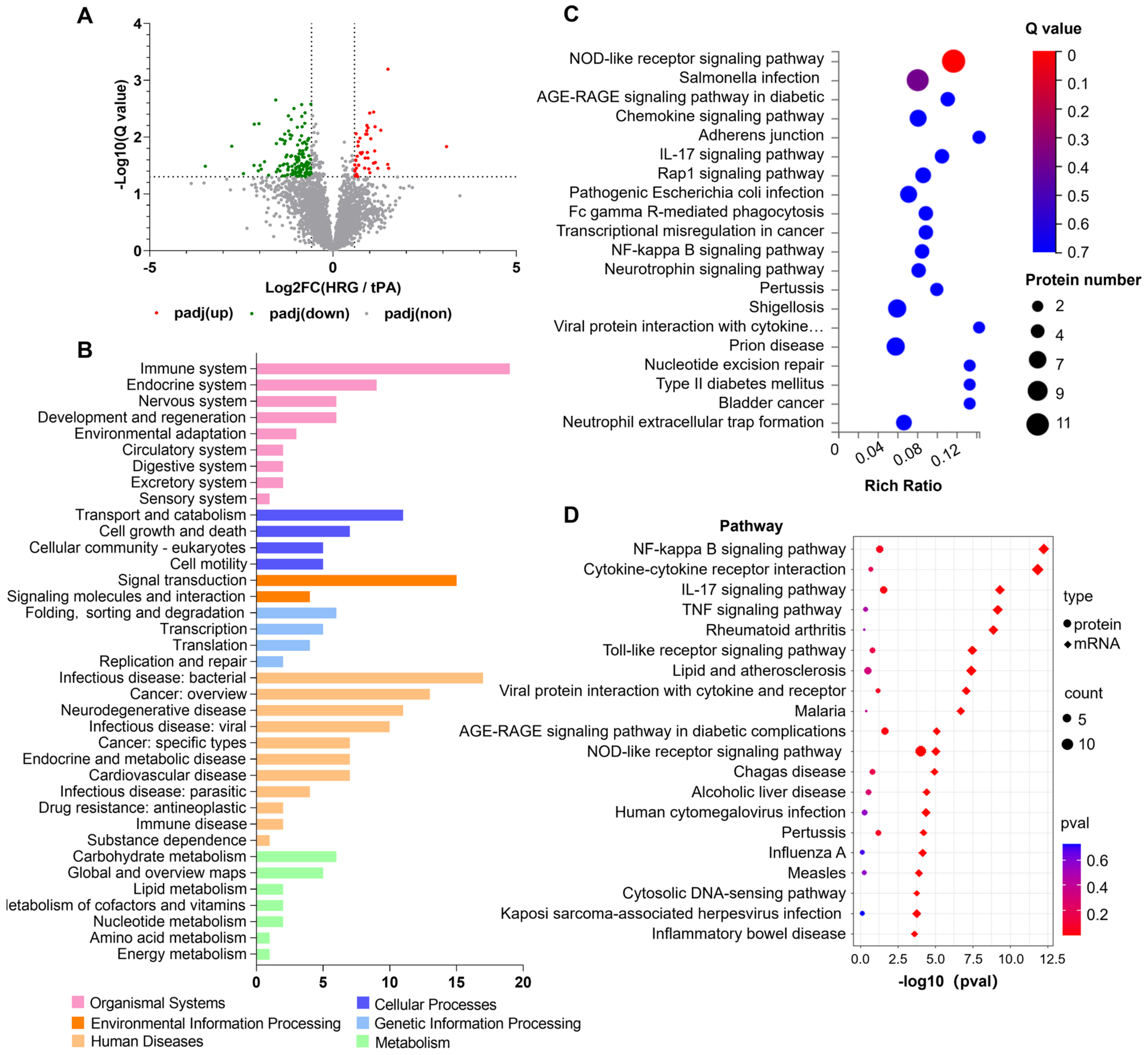

Figure EV4. Proteomic analysis and correlated differentially expressed genes and proteins of neutrophilia under HRG treatment.

(**A**) The protein profiles of neutrophils under tPA + HRG vs. tPA treatments were analyzed by the data-independent acquisition (DIA) mode ($n = 6$ per group). DEPs between two groups were identified as the padj <0.05 and fold change >1.5. The volcano plots are shown. (**B**) The downregulated DEPs were analyzed by KEGG classification in neutrophils with tPA + HRG vs. tPA treatment groups. (**C**) Bubble plot of KEGG pathway analysis of downregulated DEPs. Rich ratio represents the ratio of the number of target proteins vs. all proteins included in each pathway. Dot size represents the relative number of enriched proteins. The color of the dot represents the size of the Q-value (padj). (**D**) KEGG pathway enrichment analysis of correlated (Cor)-DEGs-DEPs. The enrichment of the proteomics is represented by circles, while the enrichment of the transcriptomics is represented by diamonds. Dot size represents the relative number of enriched proteins. The color of the dot represents the size of the Q-value (padj). For statistical methods, we performed hypergeometric test and Benjamini-Hochberg correction for Q-value (padj). We performed KEGG functional enrichment for DEGs and DEPs by clusterProfiler R package (3.8.1).

© The Author(s)

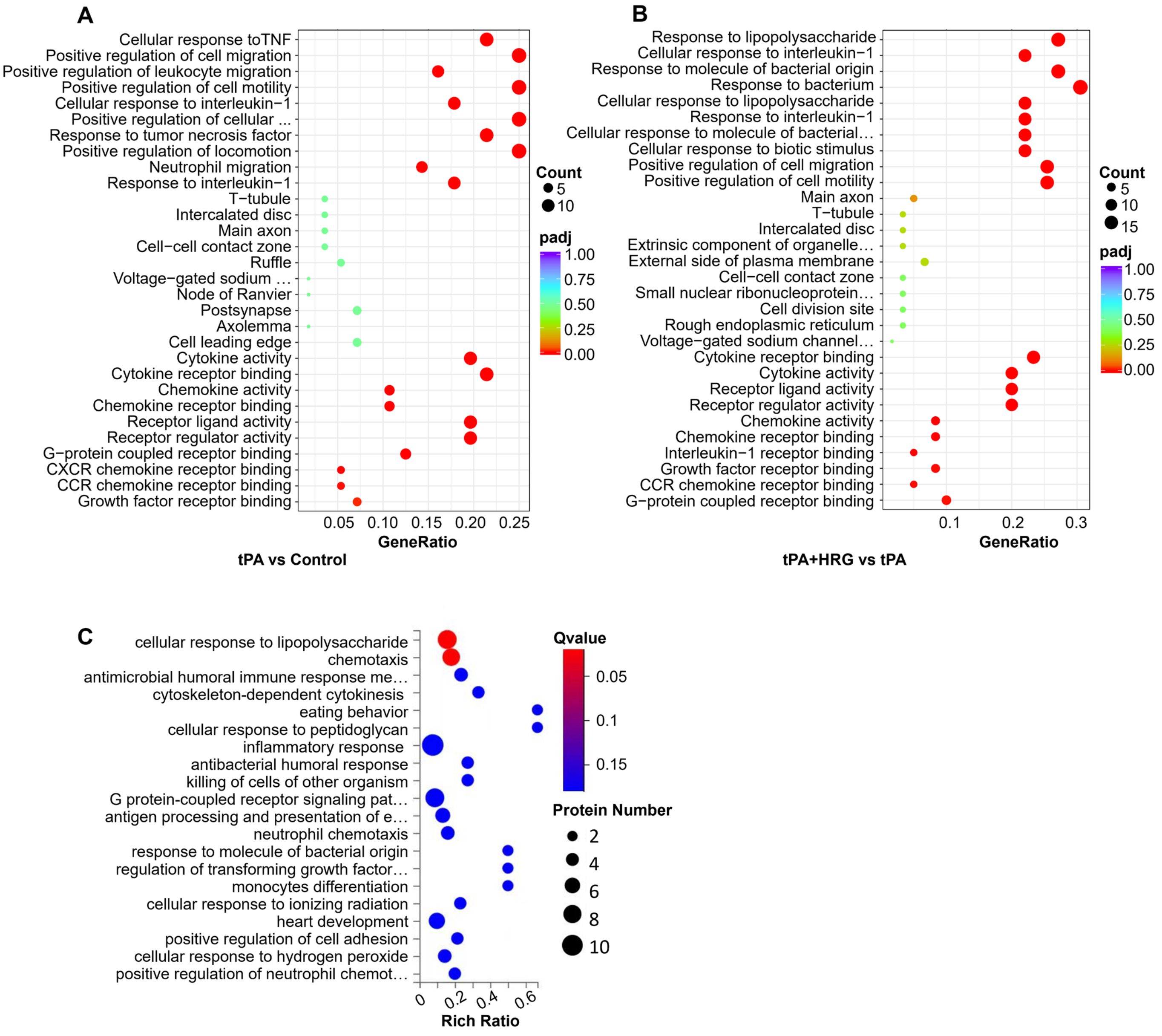

Figure EV5. Gene Ontology (GO) analysis following RNA and proteomic sequencing of purified neutrophils with recombinant tissue plasminogen activator (tPA) and histidine-rich glycoprotein (HRG) treatment.

(**A**) Functional GO enrichment analysis of the tPA vs. control group based on RNA sequencing. Enriched biological processes included cellular responses to inflammation, cell migration, and leukocyte migration. The most enriched molecular functions were cytokine activity, cytokine receptor binding, and chemokine activity. There was no significant enrichment in the cell components. We performed GO enrichment for DEGs by clusterProfiler R package (3.8.1) and we used hypergeometric test and Benjamini-Hochberg (BH) correction for padj. (**B**) Functional GO enrichment for the tPA + HRG vs. the tPA group based on RNA sequencing. The enriched biological processes mainly included the response to infectious stimulation, cellular response to IL-1, cell migration, and cell motility. Cytokine receptor binding, cytokine activity, and receptor ligand activity were significantly enriched in molecular functions. There was no significant enrichment in the cell components. We performed GO enrichment for DEGs by clusterProfiler R package (3.8.1) and we used hypergeometric test and BH correction for padj. (**C**) Functional GO enrichment for the tPA + HRG vs. the tPA group based on proteomic sequencing. The enriched biological processes mainly included the response to infectious stimulation and chemotaxis. The annotation function of the proteins was analyzed by GO using the online DAVID software, and we used hypergeometric test and BH correction for Q-value (padj).

 © The Author(s)
